# A Novel Class of Multi‐substituted Diaryl Scaffold Derivatives Inhibit Glioblastoma Progression by Targeting CD155

**DOI:** 10.1002/advs.202506688

**Published:** 2025-06-10

**Authors:** Yong‐jian Wang, Ting Sun, Si‐tu Xue, Zhong‐di Cai, Hong Yi, Miao Lv, Shi‐bo Kou, Rui Liu, Xiao‐zhong Peng, Zhuo‐rong Li

**Affiliations:** ^1^ State Key Laboratory of Bioactive Substance and Function of Natural Medicines Institute of Medicinal Biotechnology Chinese Academy of Medical Sciences and Peking Union Medical College Beijing 100050 China; ^2^ Institute of Basic Medical Sciences Chinese Academy of Medical Sciences and Peking Union Medical College Beijing 100005 China

**Keywords:** glioblastoma stem cells, multi‐substituted diaryl derivatives, poliovirus receptor cell adhesion molecule (CD155), tumor immune microenvironment

## Abstract

Glioblastoma (GBM) is the most formidable malignancy in the brain, characterized by a significant resistance to treatment. The immune targeting of glioblastoma stem cells (GSCs) holds great promise. In this study, structural modifications of the lead compound clofoctol is conducted and structure–activity relationship analyses are performed against GBM, yielding a novel blood‐brain barrier‐permeable compound, **B7**, featuring a pivotal multi‐substituted diaryl scaffold. **B7** demonstrates potent anti‐GBM effects, significantly inhibiting GSC proliferation, migration, and invasion. Notably, **B7** inhibits tumor progression, specifically bolstered natural killer (NK) cell‐mediated cytotoxicity, and mitigates the immunosuppressive microenvironment in intracranial xenograft mice implanted with GBM cells and GSCs, as well as in cocultures of GSCs and NK‐92 cells. Mechanistically, these anti‐GBM effects of **B7** are abolished by overexpression of poliovirus receptor cell adhesion molecule (CD155), both *in vitro* and *in vivo*. Further exploration reveals that **B7** targets CD155 via interaction at five crucial binding sites, namely, L47, L108, L142, M110, and V115 residues. These interactions collectively contribute to the hydrophobic interaction energies within the **B7**‐CD155 complex, modulating the CD155/T cell immunoreceptor with Ig and ITIM domains/CD226 axis to reshape the NK cell‐mediated tumor immune microenvironment. In conclusion, this study establishes the therapeutic potential of **B7** for glioma, synergistically targeting GSC biology and NK cell immunity for the treatment of GBM.

## Graphical abstract

Structural modification of clofoctol yielded a novel blood‐brain barrier‐permeable compound, **B7**, featuring a multi‐substituted diaryl scaffold. **B7** demonstrates potent anti‐glioblastoma activity by suppressing glioblastoma stem cells proliferation, migration, and invasion through interactions with five key residues of poliovirus receptor cell adhesion molecule (CD155), which enhancing natural killer cell‐mediated cytotoxicity and reshaping the tumor immune microenvironment *in vitro* and *in vivo*.

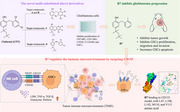

## 1 Introduction

Glioblastoma (GBM) is recognized as the most aggressive form of glioma, accounting for a concerning 48.6% of all malignant primary central nervous system cancers. It is characterized by a dismal median survival duration of merely eight months and a grim 5‐year survival rate of less than 10%.^[^ ^1^ ^]^ Despite standard therapeutic protocol combining maximal safe surgical resection, radiation, and chemotherapy, median survival rarely exceeds 15 months.^[^ ^2^, ^3^, ^4^ ^]^ The scarcity of clinically effective pharmacological options for GBM underscores the urgent need for innovative therapeutic strategies to combat this devastating disease.

The therapeutic challenges in GBM largely stem from three interlinked barriers, including glioblastoma stem cell (GSC)‐driven heterogeneity, an immunosuppressive tumor immune microenvironment (TIME), and limited blood–brain barrier (BBB) permeability.^[^ ^5^, ^6^, ^7^ ^]^ Central to this triad, GSCs are highly effective in tumor initiation, self‐renewal, and resistance to conventional therapies such as radiotherapy and temozolomide (**TMZ**).^[^ ^8^ ^]^ Accumulating evidence suggests GSCs as critical mediators of angiogenesis,^[^ ^9^ ^]^ invasion,^[^ ^10^, ^11^ ^]^ and immune escape,^[^ ^12^, ^13^ ^]^ all of which are strongly linked to unfavorable outcomes for GBM patients. Emerging therapeutic agents targeting GSCs, including ACT001^[^ ^14^ ^]^ in clinical trials, RIPGBM,^[^ ^15^ ^]^ and DP‐38003^[^ ^16^ ^]^ in preclinical stages, show selective efficacy in disrupting GSC survival pathways. These highlight the potential of GSC‐directed therapies to overcome current therapeutic limitations.

GSCs possess significant immunosuppressive properties and can shape a suppressive TIME through the dysregulated expression of key molecules,^[^ ^17^, ^18^ ^]^ including defective antigen presentation via reduced major histocompatibility complex class‐I/II (MHC‐I/II), diminished natural killer group 2 member D ligands (NKG2DL), and secretion of immunosuppressive cytokines, chemokines, growth factors, and enzymes. Novel therapeutic approaches are emerging to counteract these evasion strategies, exemplified by arming G207 viruses with a Bispecific T‐cell engager targeting NKG2DL on both GBM cells and GSCs, thereby providing a combined strategy with conventional therapies to eliminate treatment‐resistant GSCs.^[^ ^19^ ^]^ Furthermore, GSCs protect themselves against immune cell assaults by upregulating the expression of immune checkpoint ligands, where immune checkpoint inhibitors (ICIs) have succeeded in a broad spectrum of cancer immunotherapy protocols.^[^ ^20^, ^21^ ^]^ In preclinical investigations, programmed cell death 1 (PD‐1)/programmed cell death 1 ligand 1 (PD‐L1) blockade has shown promising results, significantly inhibiting tumor progression and extending survival in syngeneic GBM *in vivo* models.^[^ ^22^ ^]^ Combined treatment using dual PD‐1 and hepatitis A virus cellular receptor 2 antibodies alongside local radiation therapy has increased survival in mice with orthotopic brain tumors.^[^ ^23^ ^]^ Translational efforts are actively investigating PD‐1/PD‐L1 checkpoint inhibitors for GBM treatment in clinical trials, either as monotherapy or in combination with radiotherapy, surgery, or **TMZ**.^[^ ^24^, ^25^, ^26^ ^]^ Notably, a recent clinical case demonstrated remarkable efficacy using neoadjuvant triple‐ICI therapy prior to surgery, achieving 17 months of relapse‐free survival in advanced GBM.^[^ ^27^ ^]^ Emerging immunotherapeutic targets, such as poliovirus receptor cell adhesion molecule (CD155), T cell immunoreceptor with Ig and ITIM domains (TIGIT), CD226, cytotoxic T‐lymphocyte associated protein 4, lymphocyte activating 3, and MHC‐related pathways,^[^ ^28^, ^29^, ^30^, ^31^, ^32^ ^]^ hold great promise for GBM treatment. Increasing evidence suggests GSCs as master regulators of TIME biology, dynamically manipulating immune cell recruitment, polarization, and functional states.^[^ ^33^ ^]^ While tumor‐antagonizing immune cells, such as effector T cells and natural killer (NK) cells, initially mediate an antitumor response through persistent antigen presentation and inflammatory signaling, their activity becomes subverted via checkpoint‐mediated crosstalk, ultimately facilitating immune escape and tumor progression.^[^ ^34^ ^]^ Thus, pursuing effective therapeutic strategies that disrupt the GSC‐driven immunosuppressive network in the TIME may offer a promising avenue for future benefits for GBM patients.

Drug repositioning, the strategic repurposing of approved or investigational pharmaceuticals for novel therapeutic indications, offers a promising path to accelerate oncological drug discovery. Clofoctol (**CFT**), a clinical antimicrobial agent with a well‐established safety profile due to extensive clinical applications, has shown efficacy in rectal treatments for Gram‐positive bacterial infections.^[^ ^35^ ^]^ **CFT** has been repositioned as a potential therapeutic option for tumors, such as oral cancers,^[^ ^36^ ^]^ GBM,^[^ ^37^ ^]^ and prostate cancer.^[^ ^38^ ^]^ Due to its small molecular size and hydrophobic characteristics, **CFT** exhibits high permeability, enhancing its ability to cross the BBB. Regarding GBM, recent studies by the Peng laboratory have discovered that **CFT** selectively inhibits the proliferation of GSCs.^[^ ^37^ ^]^ Mechanically, **CFT** acts on the RNA‐binding protein Upstream of N‐ras (UNR), stabilizing the interaction with Kruppel‐like factor 13 (KLF13) mRNA. This enhanced interaction stimulates the upregulation of KLF13 expression, ultimately triggering apoptosis in GSCs. Beyond this primary mechanism, **CFT** exhibits multimodal activities, including interactions of the KLF13/UNR, cell division cycle 7/DBF4 zinc finger kinase complex, replication and translation processes in the SARS‐CoV‐2 life cycle, and production of inflammatory cytokines.^[^ ^36^, ^39^ ^]^ However, crucial insights into the structure–activity relationship (SAR) governing the inhibitory effect of **CFT** on GBM remain to be elucidated. In addition, whether optimized derivatives of **CFT** exhibit fresh mechanisms of action against GBM warrants further exploration.

In the present study, we strategically designed a novel series of **CFT** derivatives through scaffold‐oriented molecular optimization, preserving multi‐substituted diaryl framework while incorporating distinct substitution patterns to enhance target engagement. Through systematic evaluation of anti‐glioma activity, biosafety, and SAR analysis, we identified **B7** (2‐(2‐methylbenzyl)‐4‐(2,4,4‐trimethylpentan‐2‐yl)phenol) as a BBB‐permeable lead candidate that demonstrated significant anti‐GBM effects with dual‐mode anti‐GSC mechanisms that directly suppressed GSCs while concurrently activated NK cell‐mediated immunity via the CD155/TIGIT/CD226 immune checkpoint axis within the TIME, mechanically distinct from its parent compound **CFT**. Molecular dynamics simulation combined with experimental verification revealed five key CD155 residues (L47, L108, M110, V115, and L142) mediating **B7** binding, remodeling immunosuppressive TIME into an NK cell‐activated state, combining GSC inhibition with innate immune potentiation. Our findings pioneer multi‐substituted diaryl scaffold derivatives that synergistically target GSC biology and NK cell immunity, providing critical insights into the CD155‐mediated stemness regulation for GBM treatment.

## 2 Results

### 2.1 Design of Derivatives and Chemical Synthesis

**Figure 1:**
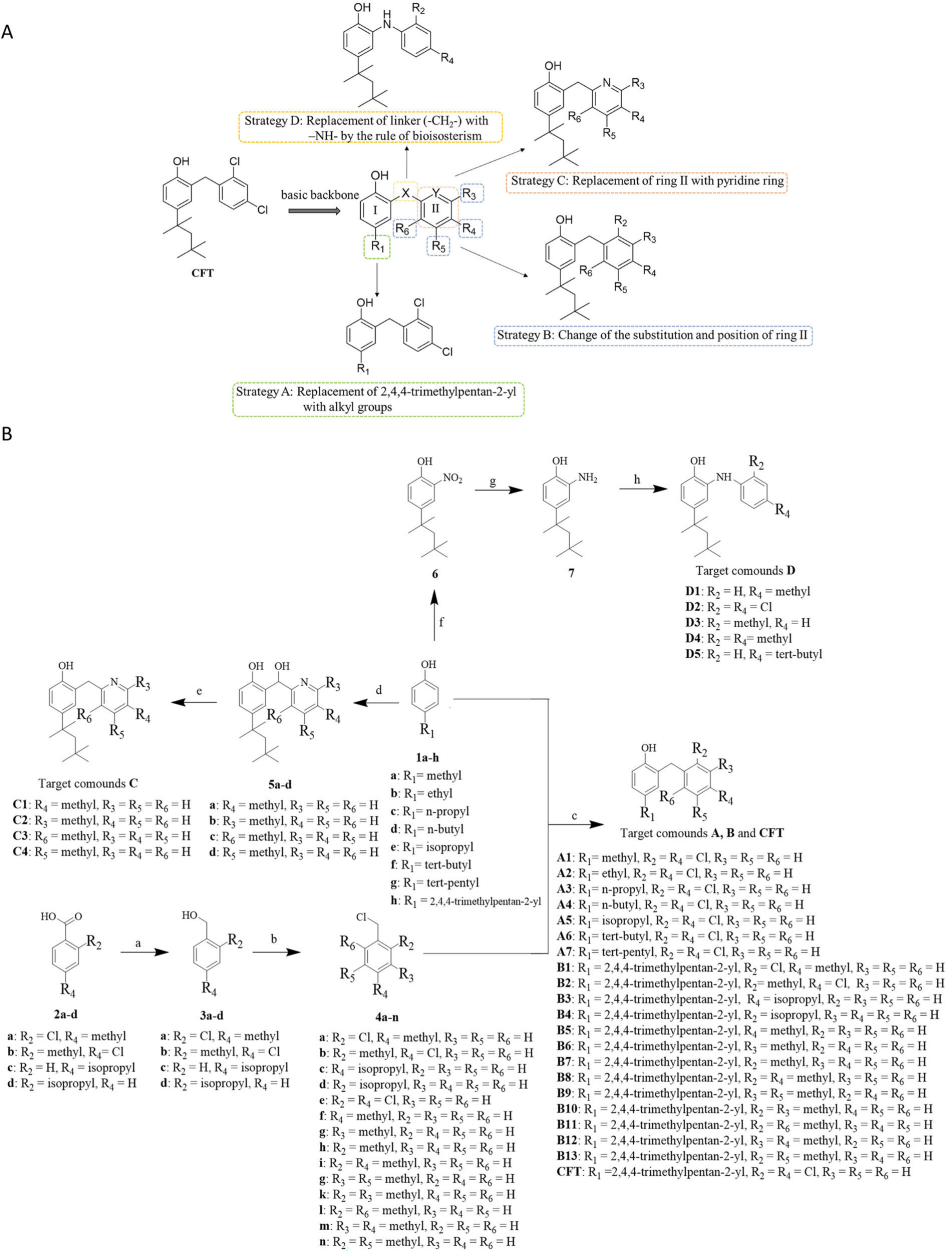
Illustrative representation of the design concept and chemical synthesis route map for the derivatives. A) Strategies employed in derivative design. B) Synthesis methods utilized for target compounds.

**CFT** is the lead compound demonstrating promising anti‐glioma efficacy, providing a modifiable basic backbone of the diaryl scaffold structure. Based on this scaffold, four distinct modification strategies were systematically designed to investigate the SARs (**Figure** **1**A). The main modification strategies are as follows. i) Strategy A: Systematically optimizing the length of the 2,4,4‐trimethylpentan‐2‐yl side chain attached to Ring I to identify the optimal length while evaluating the effect of branched aliphatic chain on biological activity. ii) Strategy B: Modulating the electronic and steric properties of substituents on Ring II through strategic positioning and functional group manipulation. iii) Strategy C: Replacing Ring II with a pyridine ring to investigate the impact of nitrogen atom introduction, thereby exploring the consequences of replacing a phenyl moiety with a π‐deficient heterocycle. (iv) Strategy D: Altering the linker connecting Ring I and II via bioisosteric transformations, evaluating classical isosteres (CH₂→NH) to modulate conformational flexibility and electronic properties while preserving the critical distance between aromatic pharmacophores.

This comprehensive approach resulted in the synthesis of 30 diaryl scaffold derivatives, comprising 7 under Strategy A (A series), 13 under Strategy B (B series), 4 under Strategy C (C series), 5 under Strategy D (D series), alongside the lead compound, **CFT** itself. The synthetic route is shown in Figure 1B.

### 2.2 *In Vitro* Activity Screening and SAR Analysis of the Derivatives

To establish an *in vitro* screening platform for potential anti‐glioma drugs, we evaluated 29 derivatives and the lead **CFT** against four human GBM cell lines (U87‐MG, U251‐MG, LN229, and U118‐MG) using MTS assay. Cells were treated with compounds at different concentrations for 72 h to determine IC_50_ values. **TMZ**, the clinical first‐line chemotherapy agent for glioma, and carmustine, a nitrosourea alkylating agent recommended by the U.S. Food and Drug Administration (FDA) for GBM treatment, were used as positive controls. The anti‐glioma effects of the derivatives were directly compared with **TMZ**, carmustine, and **CFT**.

Overall, derivatives of the B series exhibited the most significant inhibitory activity against all four tested GBM cells, followed by the D, C, and A series (**Table** **1**). Compounds **B7** and **B8,** along with the lead **CFT**, demonstrated remarkable inhibitory effects. Specifically, **B7** showed an IC_50_ ranging from 3.43 to 14.60 µm, exhibiting an activity 2–3 times higher than **TMZ** (IC_50_ = 10.20–34.40 µm) and 2–5 times more effective than carmustine (IC_50_ = 17.45–29.00 µm), equivalent to that of **CFT** (IC_50_ = 4.94–11.88 µm). Against LN229 cells, **B7** displayed the most potent anti‐proliferative activity with an IC_50_ of 3.43 µm, marginally better than **CFT** (IC_50_ = 4.94 µm). **B8**, with IC_50_ values between 6.56 and 14.43 µm, also showed remarkable anti‐glioma activities, 2–4 times higher than **TMZ** and 2–3 times more potent than carmustine, comparable to **CFT**. **B8** was the most effective against U251‐MG (IC_50_ = 7.02 µm) and U87‐MG (IC_50_ = 9.14 µm) cells, slightly better than the effects of **CFT** (9.06 and 10.91 µm).

**Table 1:** Proliferation inhibitory rate of the derivatives against four glioblastoma cells.

Compound	IC_50_ ^a)^ [µm]			
	U251‐MG	U87‐MG	U118‐MG	LN229
**A1**	–	–	93.09 ± 6.17	135.63 ± 11.30
**A2**	–	–	87.00 ± 8.12	116.33 ± 0.82
**A3**	–	–	104.60 ± 2.96	144.27 ± 0.69
**A4**	–	–	57.15 ± 12.83	141.07 ± 22.44
**A5**	–	–	100.79 ± 4.90	329.73 ± 12.15
**A6**	–	–	89.53 ± 13.77	104.07 ± 16.50
**A7**	–	–	90.84 ± 16.22	147.27 ± 26.01
**B1**	–	59.93 ± 13.05	96.53 ± 7.33	133.43 ± 16.14
**B2**	102.71 ± 12.38	46.68 ± 4.72	88.20 ± 7.28	125.67 ± 18.30
**B3**	50.79 ± 7.08	39.86 ± 6.59	84.00 ± 8.89	76.59 ± 7.00
**B4**	41.02 ± 5.33	39.21 ± 0.58	67.73 ± 3.32	123.30 ± 1.42
**B5**	45.50 ± 1.40	32.80 ± 5.79	85.62 ± 9.87	68.64 ± 11.38
**B6**	31.94 ± 0.72	71.59 ± 17.23	87.21 ± 4.25	129.63 ± 31.10
**B7**	9.68 ± 0.18	13.21 ± 0.59	14.60 ± 0.61	3.43 ± 0.10
**B8**	7.02 ± 0.90	9.14 ± 0.87	14.43 ± 0.87	6.56 ± 0.03
**B9**	47.58 ± 4.93	25.14 ± 0.32	89.06 ± 5.57	96.42 ± 1.13
**B10**	41.22 ± 0.99	31.72 ± 2.82	91.72 ± 6.21	85.58 ± 4.33
**B11**	29.19 ± 0.52	24.70 ± 0.56	70.18 ± 6.51	104.37 ± 11.75
**B12**	82.24 ± 5.69	28.10 ± 2.29	105.01 ± 6.93	149.73 ± 19.42
**B13**	65.53 ± 8.57	23.06 ± 1.04	117.00 ± 12.13	103.13 ± 0.49
**C1**	120.05 ± 5.85	45.82 ± 1.53	166.47 ± 9.62	148.67 ± 31.72
**C2**	177.75 ± 18.85	44.44 ± 2.02	203.03 ± 4.51	–
**C3**	83.08 ± 7.05	42.55 ± 0.31	100.99 ± 5.76	–
**C4**	91.56 ± 5.20	36.62 ± 2.31	224.83 ± 28.95	–
**D1**	62.46 ± 6.64	26.98 ± 1.81	305.90 ± 22.39	118.17 ± 3.84
**D2**	101.12 ± 4.13	22.63 ± 2.50	365.23 ± 30.77	243.9 ± 62.09
**D3**	65.04 ± 5.24	30.16 ± 3.69	135.87 ± 8.46	111.10 ± 5.91
**D4**	100.21 ± 14.45	27.66 ± 2.45	118.10 ± 9.41	100.44 ± 20.18
**D5**	37.36 ± 0.74	23.51 ± 1.27	110.44 ± 12.95	158.63 ± 28.67
**CFT**	9.06 ± 3.36	10.91 ± 0.44	11.88 ± 3.90	4.94 ± 0.67
**TMZ**	25.93 ± 1.05	15.79 ± 0.59	34.40 ± 0.08	10.20 ± 1.70
Carmustine	19.24 ± 1.39	29.00 ± 0.32	23.01 ± 2.34	17.45 ± 8.90

^a)^ IC_50_ values represented the concentration of the compounds that inhibited cell growth by 50% compared to control. The results were expressed as mean ± SD, *n* = 3. The symbol “‐” indicated that none of the tested concentrations of the compound resulted in more than 50% inhibition of cell proliferation.

The A series, which focused on modifying the length of the 2,4,4‐trimethylpentan‐2‐yl side chain on Ring I, generally exhibited weak inhibitory activity across all tested cell lines, with IC_50_ values consistently above 50 µm. From **A1** to **A4**, the R_1_ side chain of Ring I gradually lengthens, with **A4** showing the marginal inhibition against U118‐MG cells (57.15 µm). The activity was not enhanced by shortening the carbon chain and increasing the number of branch chains in **A5**–**A7**. The loss of activity against U251‐MG and U87‐MG cells suggests that structural modification at this position critically compromise anti‐glioma effects.

The B series, which involved modulating substituents and their positions on Ring II, exhibited a wide range of activity, with **B7** and **B8** demonstrating the strongest inhibitory effects, comparable to **CFT**. Methyl groups were substituted for chlorine in **CFT** to obtain **B1** and **B2**, with IC_50_ values of 59.93 and 46.68 µm (against U87‐MG cells), respectively. These results suggest that methyl substitution retains partial activity. **B5**, **B6**, and **B7** had different methyl substitution positions, with **B7** exhibiting outstanding inhibitory activity against all four cell lines. The IC_50_ values of **B5** ranged from 32.80 to 85.62 µm, and those of **B6** from 31.94 to 129.63 µm. Substitution at the R_2_ position proved more effective than at R_4_ and R_3_. Increasing alkyl carbons led to **B3** and **B4**, with IC_50_ values of 39.86–84.00 and 39.12–123.30 µm, respectively, indicating that the isopropyl group retained some activity. **B8**–**B13** featured two methyl groups at different locations on Ring II, with **B8** demonstrating the best activity against the four cell lines. Methyl substitution at both R_2_ and R_4_ positions yielded the most effective results, highlighting the significance of substitution position.

In the C series, Ring II was replaced with a pyridine ring, showing certain inhibitory activity against U87‐MG cells. The IC_50_ values of **C1**–**C4** for U87‐MG cells ranged from 36.62 to 45.82 µm. Among them, **C4** had the best methyl‐substitution activity (R_5_), but was still significantly weaker than **CFT**. This suggests that the introduction of nitrogen atoms into the ring leads to a significant decrease in biological activity.

In the D series, **D1**–**D5** changed the linking group between Ring I and II, exhibiting certain inhibitory activity on U87‐MG cells. The IC_50_ values ranged from 22.63 to 30.16 µm, with **D2** demonstrating the best inhibitory effect on U87‐MG cells. **D5** also showed strong activity (IC_50_ = 23.51 µm). The R_4_ in Ring II of **D5** was an alkyl substituted tert‐butyl group, potentially contributing to its superior activity.

The key SARs for these derivatives are as follows: i) Diminution of the side chain length considerably weakened its inhibitory activity against the four GBM cells. Notably, the length and branching of the side chains in Ring I considerably affected the activity, with the presence of a unique 2,4,4‐trimethylpentan‐2‐yl chain crucial for activity maintenance. ii) Introducing methyl substitutions was beneficial for the activity. Substituting the chlorine atom in Ring II with a monomethyl group yielded **B7**, which is markedly potent in inhibiting all targeted cell types. Strategic positioning methyl on Ring II revealed R_2_ superior to R_4_ and R_3_, while optimal anti‐glioma effects arose from dimethyl‐substituted positions at R_2_ and R_4_. Although alkyl substitution showed beneficial potential, the exact position of substitution influenced its effectiveness. iii) Replacing Ring II with a pyridine ring is not recommended based on our present findings. iv) Modification by substituting ‐CH_2_‐ with ‐NH‐ significantly reduced inhibitory activity against U251‐MG, U118‐MG, and LN229 cells, while exhibiting minimal impact on U87‐MG cells, with activities either stable or slightly declined. Overall, **B7** emerged as the most potent derivative due to the presence of the 2,4,4‐trimethylpentan‐2‐yl side chain and optimized R_2_‐methyl substitution on Ring II. These findings suggest that optimizing alkyl substitution may effectively elevate biological activity while preserving the essential structural framework for potency, establishing **B7** as the most promising derivative in this series.

### 2.3 Toxicity Evaluation and ADMET Prediction of Synthesized Compounds

Comprehensive cytotoxicity assessment was evaluated across five vital *in vitro* models: THLE‐2 (hepatic), H9c2(2‐1) (cardiac), HT22 (neuronal), Vero (renal), and HEK‐293 (embryonic) cells (Tables S1–S5, Supporting Information). All compound series exhibited low cytotoxicity in Vero and HEK‐293 cells, indicating minimal nephrotoxic potential. The B series derivatives demonstrated higher hepatotoxicity and neurotoxicity compared to the A, C, and D series. Notably, **B7** exhibited markedly reduced cytotoxicity across all tested cell lines compared to both **CFT** and **B8**, with cytotoxic profiles comparable to the control drugs, **TMZ** and carmustine.

Given the critical requirement for BBB penetration in glioma therapeutics, the capacity of **CFT**, **B7**, and **B8** was predicted through ADMET (absorption, distribution, metabolism, excretion, and toxicity) using Discovery Studio 3.0 (Table S6, Supporting Information). The results were presented as a rating scale, revealing very low solubility and a plasma protein binding rate of ≥90% for these three, with potential actions as CYP2D6 enzyme inhibitors. Despite the suboptimal absorption levels observed for **CFT**, **B7**, and **B8**, **B7** was better than its counterparts. **B7** was predicted to have extremely high BBB permeability, whereas **B8** fell outside the confidence elliptic interval, making its permeability undefined. Subsequent *in vivo* penetration validation experiments through liquid chromatography analysis for **B7** and **B8** showed that **B7** achieved rapid enrichment in the brain after 5 min of injection, with less distribution observed in the heart, liver, spleen, lung, and kidney tissues (Figure S1, Supporting Information). Conversely, **B8** demonstrated poor BBB permeability (Figure S2, Supporting Information), consistent with the predicted ADMET profiles, which prompted further studies to focus on **B7**. Acute toxicity assessment confirmed the safety of **B7** at doses not exceeding 100 mg kg^−1^, with treated mice maintaining normal behavior and activities (Table S7, Supporting Information). Considering its lower toxicity profile and superior BBB permeability, **B7** was selected for further investigation of its anti‐glioma effects and exploration of the underlying mechanism.

### 2.4 The Effect of Compound **B7** against Glioma *In Vivo*

**Figure 2:**
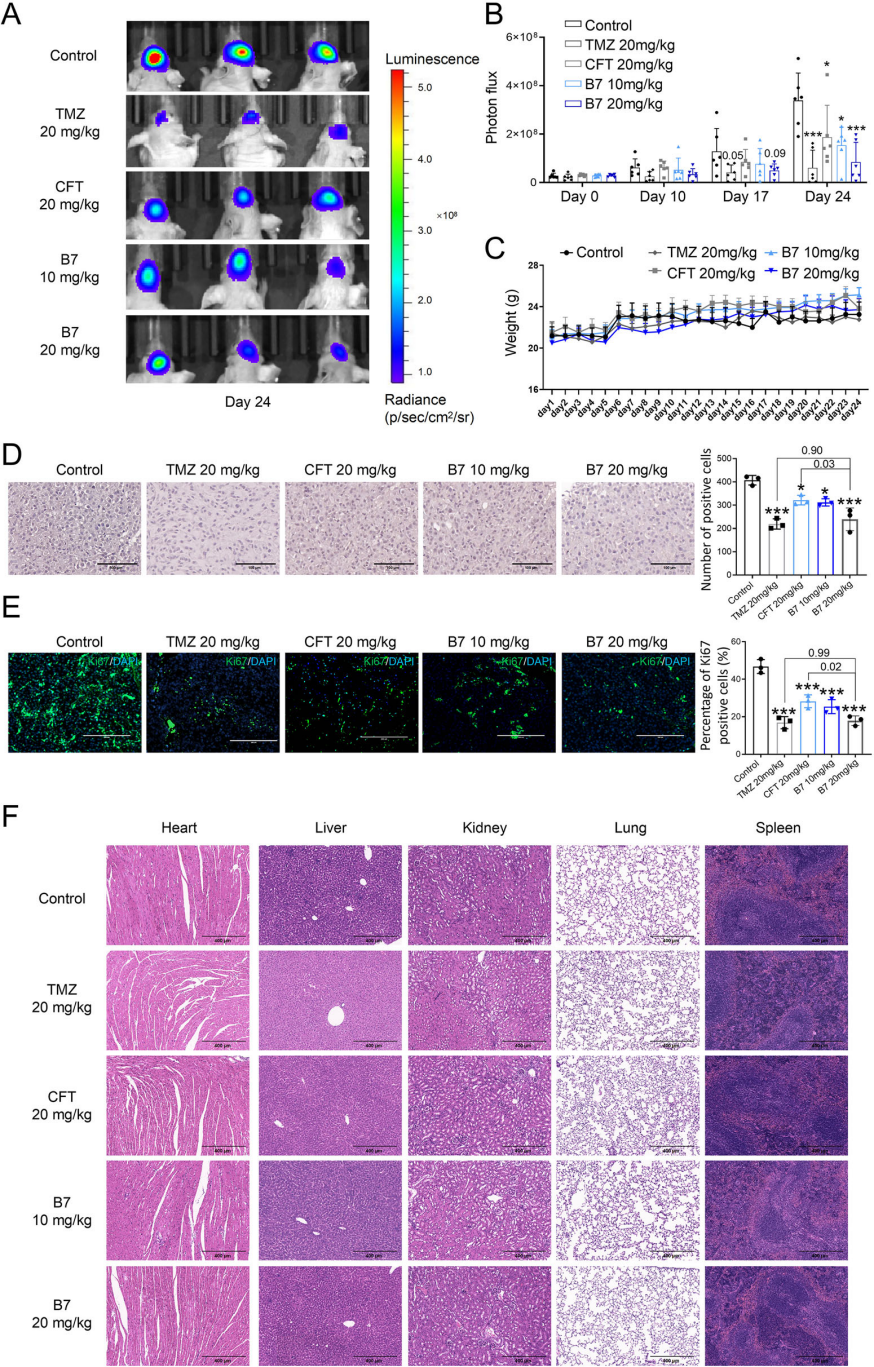
The activity of compound **B7** against glioma *in vivo*. A) Representative bioluminescent images of nude mice, *n* = 3. B) Quantitative analysis of photon flux of nude mice, *n* = 6. C) Body weight changes of nude mice, *n* = 6. D) Representative H&E staining images of tumor tissues in the brain of nude mice, *n* = 3, bar: 100 µm. E) Representative immunohistochemical staining images for Ki67 in tumor tissues of nude mice, *n* = 3, bar: 200 µm. F) Representative H&E staining images of heart, liver, kidney, lung, and spleen tissues of nude mice, bar: 400 µm. One‐way ANOVA, followed by Tukey's post hoc test, was used for multiple group comparisons to determine differences between groups. The results were presented as mean ± SD. ^*^ *P* < 0.05, ^**^ *P* < 0.01, ^***^ *P* < 0.001 versus Control.

The effects of **B7** on attenuating tumor progression *in vivo* were examined using an intracranial xenograft model with a fluorescence‐labeled human brain GBM cell line (U‐87 MG‐Luc2) implanted into the frontal cortex of BALB/c nude mice. Quantitative photon flux analysis of six mice per group showed that 10 and 20 mg kg^−1^ **B7** significantly inhibited tumor growth after 24 days of treatment (**Figure** **2**A,B, both *P* < 0.05 vs Control). Specifically, the tumor inhibition effect of **B7** at 20 mg kg^−1^ was superior to that of **CFT** at 20 mg kg^−1^ (*P* = 0.033, Figure 2B), also matching the efficacy of **TMZ** at 20 mg kg^−1^ (*P* = 0.527, Figure 2B). No significant fluctuations in body weight were observed in these mice throughout the experiment (Figure 2C).

Histological evaluation using hematoxylin and eosin (H&E) staining further corroborated the efficacy of **B7** in mitigating tumor progression, with doses of 10 and 20 mg kg^−1^ exhibiting beneficial therapeutic effects (Figure 2D, both *P* < 0.05 vs Control). Consistent findings were observed in the immunofluorescence staining of Ki67, demonstrating that **B7** effectively suppressed Ki67 expression, thereby decelerating tumor proliferation (Figure 2E, both *P* < 0.001 vs Control). Moreover, the 20 mg kg^−1^ **B7** group exhibited a more pronounced inhibitory effect on tumor‐associated histological changes compared to 20 mg kg^−1^ **CF** **T** (*P* = 0.03, Figure 2D; *P* = 0.02, Figure 2E), showing promise in reaching the anti‐GBM efficacy comparable to **TMZ** (*P* = 0.90, Figure 2D; *P* = 0.99, Figure 2E). Notably, **B7** demonstrated no toxicity toward nude mice, as evidenced by the absence of notable histological damage in vital organs (heart, liver, kidney, lung, and spleen) upon H&E staining (Figure 2F). Furthermore, no significant alterations were detected in serum biochemical markers, including aspartate aminotransferase (AST), alanine aminotransferase (ALT), blood urea nitrogen (BUN), creatine kinase (CK), creatinine (CRE), and lactate dehydrogenase (LDH), indicating a favorable safety profile of **B7** (Figure S3, Supporting Information).

### 2.5 Compound **B7** Targets the Functionality of GSCs

**Figure 3:**
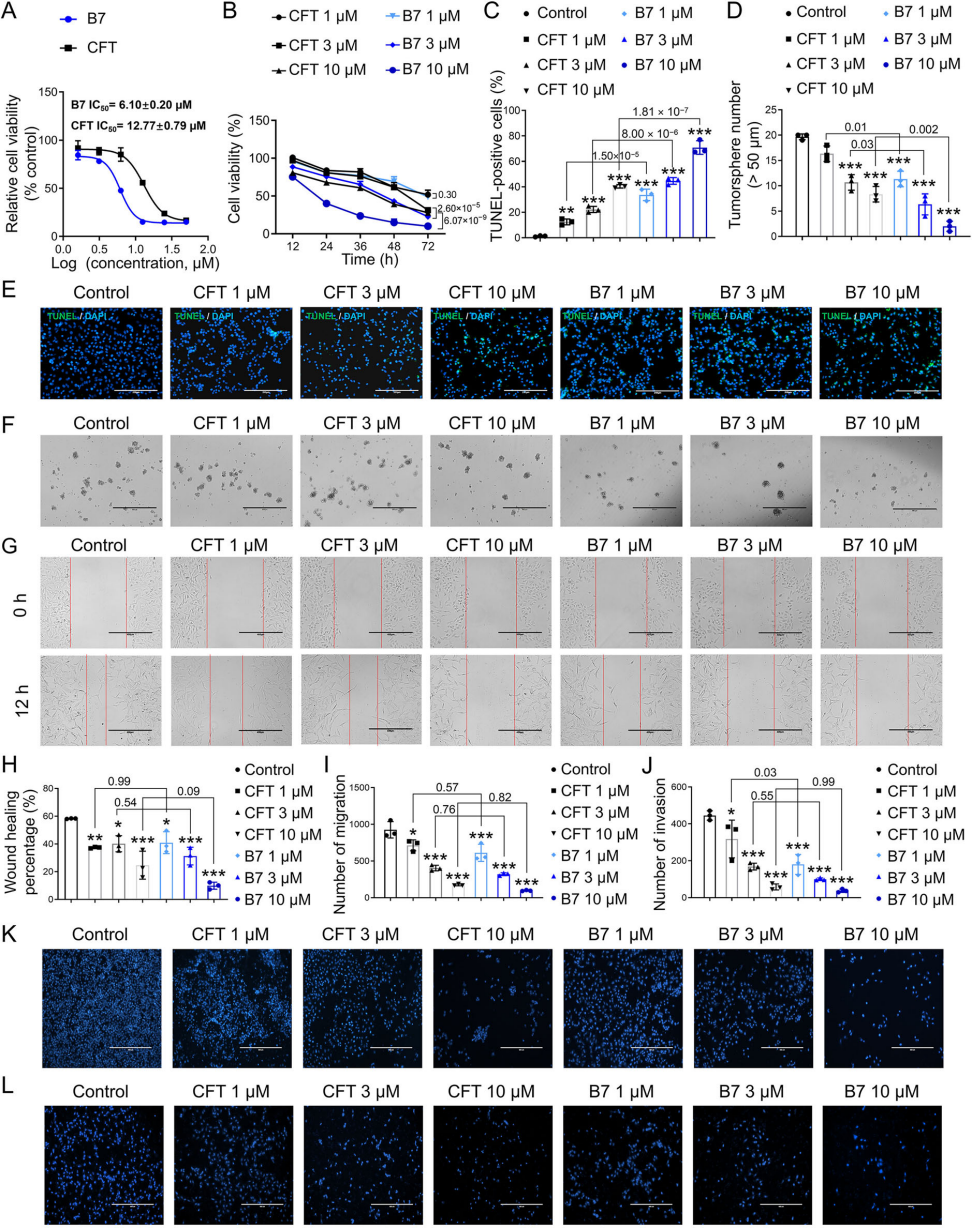
Effects of **B7** on the viability and functionality of GSCs. A) Effects of **B7** and **CFT** on the viability of GSCs and IC_50_ values. B) GSC viability at 12–72 h. C,E) Quantitative analysis (C) and representative images (E) for the evaluation of GSC apoptosis using TUNEL staining, bar: 200 µm. D,F) Quantitative analysis (D) and representative images (F) of the GSC sphere formation assays, bar: 400 µm. G,H) Representative images (G) and quantification (H) for the effects of **B7** and **CFT** on GSC migration in scratch assays, bar: 400 µm. I,K) Quantification (I) and representative images (K) of transwell migration assays, bar: 400 µm. J,L) Quantification (J) and representative images (L) of transwell invasion assays, bar: 400 µm. Comparisons among multiple groups were analyzed using one‐way ANOVA, followed by Tukey's *post hoc* test. The results were presented as mean ± SD, *n* = 3. ^*^ *P* < 0.05, ^**^ *P* < 0.01, ^***^ *P* < 0.001 versus Control.

As crucial mediators of gliomagenesis, drug resistance, and recurrence,^[^ ^40^ ^]^ GSCs represent pivotal targets for intervention. **B7** displayed potent anti‐GSC activity, characterized by its multimodal properties. Specifically, **B7** significantly inhibited the proliferation of GSCs, as evidenced by an IC_50_ value of 6.10 ± 0.20 µm, nearly halving that of **CFT** at 12.77 ± 0.79 µm (**Figure** **3**A). Continuous assessments of GSC survival rates over 12–72 h revealed that **B7** dose‐dependently inhibited GSC activity at concentrations of 1, 3, and 10 µm (Figure 3B). Notably, **B7** exhibited significantly greater potency than **CFT** at concentrations of 3 and 10 µm (*P* = 2.60 × 10^−5^ and 6.07 × 10^−9^, Figure 3B). Meanwhile, our findings from the TdT‐mediated dUTP Nick‐End Labeling (TUNEL) staining experiments demonstrated a dose‐dependent proapoptotic effect of **B7** on GSCs (Figure 3C,E, all *P* < 0.001 vs Control). Sphere formation, a crucial feature of GSCs, was significantly reduced in GSCs following **B7** treatment (1, 3, and 10 µm) (Figure 3D,F, all *P* < 0.001 vs Control). Consistently, **B7** outperformed **CFT** in inducing apoptosis and reducing the sphere‐forming ability of GSCs (*P* = 1.50 × 10^−5^, 8.00 × 10^−6^, and 1.81 × 10^−7^ for 1, 3, and 10 µm, Figure 3C,E; *P* = 0.01, 0.03, and 0.002 for 1, 3, and 10 µm, Figure 3D,F). Scratch and transwell migration assays were performed to assess the GSC migration potential, and the results showed that different concentrations of **B7** reduced the wound healing percentage and the number of migrating GSCs compared to the control group (Figure 3G–I,K, all *P* < 0.05 vs Control). **B7** at 10 µm exhibited the greatest inhibitory effect. Invasion assay additionally showed that **B7** significantly diminished the invasion capability of GSCs (Figure 3J,L, all *P* < 0.001 vs Control), equivalent to **CFT** (*P* = 0.03, 0.55, and 0.99 for 1, 3, and 10 µm, Figure 3J,L). Collectively, **B7** exhibited robust and inhibitory effects against GSCs, effectively targeting their core functions, outperforming **CFT** in inhibiting proliferation, inducing apoptosis, and blocking sphere formation, while matching its anti‐migration and anti‐invasive efficacies.

### 2.6 CD155 Serves as the Target for Compound **B7** to Inhibit GSCs

**Figure 4:**
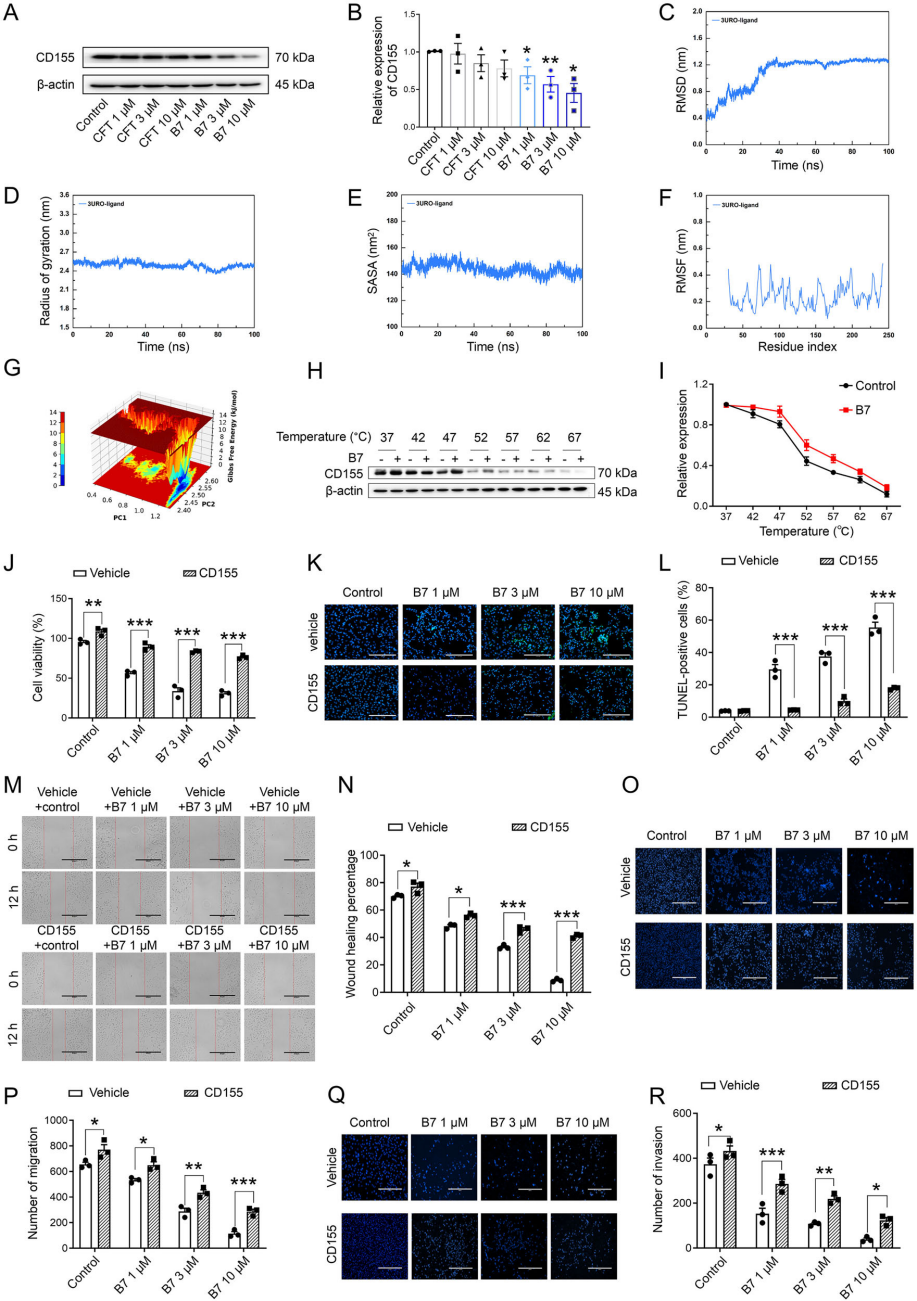
CD155 serves as the target for compound **B7** to inhibit GSCs. A,B) Representative images (A) and quantitative analysis (B) of Western blotting analysis for CD155 protein expression. C–G) Molecular dynamics (MD) simulation analysis of **B7**‐CD155 complex, including the root mean square deviation (RMSD) (C), radius of gyration (Rg) (D), solvent‐accessible surface area (SASA) (E), root mean square fluctuation (RMSF) (F), and free energy landscape (FEL) (G). H,I) Representative images (H) and fitting curve (I) for the evaluation of **B7**‐CD155 binding efficiency using Cellular Thermal Shift Assay (CETSA). J) The cell viability of GSCs. K,L) Representative images (K) and quantification (L) of TUNEL staining, bar: 200 µm. M,N) Representative images (M) and quantification (N) of the scratch assay, bar: 200 µm. O,P) Representative images (O) and quantification (P) of transwell migration assay, bar: 400 µm. Q,R) Representative images (Q) and quantification (R) of transwell invasion assay, bar: 400 µm. Comparisons across multiple groups were analyzed using one‐way ANOVA and Tukey's *post hoc* test in panel B. Two‐tailed Student's *t*‐test was employed for two‐group comparisons in panels J, L, N, P, and R. The results were expressed as mean ± SD, *n* = 3. ^*^ *P* < 0.05, ^**^ *P* < 0.01, ^***^ *P* < 0.001 vs Control or corresponding vehicle.

In previous studies, KLF13 was identified as a potential target of **CFT** through transcriptional upregulation.^[^ ^37^ ^]^ Nonetheless, our present study reveals that KLF13 is not a pivotal target of **B7**, a new derivative of **CFT**, and that the mRNA expression of KLF13 remains unaltered by **B7** against GSCs, in clear contrast to the effects on KLF13 expression observed in **CFT** (Figure S4A, Supporting Information). Based on the active functional groups of **B7**, compound target prediction combined with Kyoto Encyclopedia of Genes and Genomes (KEGG) and Gene Ontology (GO) analyses revealed that **B7** may exert anti‐GBM effects by modulating key immune regulatory processes, including leukocyte activation, lymphocyte activation, leukocyte migration, and immune response activation (Figure S5A, Supporting Information). Specifically, **B7** engaged in histone kinase activity, phosphotransferase activity, and stem cell factor receptor activity at dendrites, postsynapses, synaptic membranes, proteasome core complexes, and receptor complexes (Figure S5B,C, Supporting Information). KEGG pathway enrichment identified significant associations with cancer‐related pathways, PD‐L1 expression and PD‐1 checkpoint pathways in cancer, apoptosis, chemokine signaling pathway, B cell receptor signaling pathway, Toll‐like receptor signaling pathway, T cell receptor signaling pathway, and NK cell mediated cytotoxicity (Figure S5D, Supporting Information). These findings suggest that **B7** may reprogram the TIME to counteract GBM progression, which is critically dependent on immune evasion mechanisms of GSCs. Notably, GBM progression is driven by the ability of GSCs to evade immune surveillance through the expression of checkpoint inhibitors (e.g., PD‐L1, PD‐L2, B7‐H3, V‐set domain containing T cell activation inhibitor 1 (B7‐H4), V‐set immunoregulatory receptor (VISTA), Human endogenous retrovirus‐H Long repeat‐associating 2 (HHLA2), CD47, CD80, and CD86) and the downregulation of antigen‐presenting molecules (e.g., CD155, nectin cell adhesion molecule 2 (CD112), nectin cell adhesion molecule 3 (CD113), Galectin‐3, Galectin‐9, MHC class molecules, CD40, V‐set and immunoglobulin domain containing 4 (VSIG4), selectin P ligand (PSGL‐1), OX40 ligand (OX40L), inducible T cell costimulator (ICOS), NK cell cytotoxicity receptor 3 ligand 1 (NCR3LG1), and tumor necrosis factor superfamily member 9 (CD137L)).^[^ ^41^, ^42^, ^43^, ^44^ ^]^ Therefore, through a focused targeting and comprehensive screening of these GSC‐intrinsic immunomodulatory factors (Figure S4B–X, Supporting Information), CD155, an oncogenic antigen overexpressed in GBM that facilitates migration and invasiveness,^[^ ^45^ ^]^ was identified to be suppressed by **B7**, thereby differentiating **B7** from the anti‐glioma target of **CFT** (**Figure** **4**A,B; Figure S4B, Supporting Information, all *P* < 0.05 vs Control).

To elucidate the dynamic properties of the **B7**‐CD155 interplay, comprehensive 100 ns molecular dynamics (MD) simulations were performed. The stability of the **B7**‐CD155 complex was evaluated by analyzing various parameters, such as root mean square deviation (RMSD), root mean square fluctuation (RMSF), radius of gyration (Rg), and solvent‐accessible surface area (SASA). The RMSD analysis yielded an average poststabilization value of 1.242 ± 0.029 nm, signifying minimal fluctuations and robust overall stability of the protein‐ligand complex throughout the simulation (Figure 4C). The Rg, indicative of protein folding and unfolding, exhibited an average of 2.492 ± 0.046 nm with minimal fluctuations, underscoring the compact and stable nature of the complex (Figure 4D). The SASA remained steady with minor variations, averaging 144.004 ± 4.008 nm^2^, highlighting structural integrity (Figure 4E). The RMSF reflecting amino acid residue flexibility was low, indicating stability across the protein atoms during simulation (Figure 4F). These findings collectively demonstrate the high stability of the **B7**‐CD155 complex.

This stability was further validated by the free energy landscape (FEL), which depicted a single, smooth energy cluster at the lowest potential energy level, indicative of potent and stable interactions. The FEL visually corroborated this stability with deep purple/blue spots corresponding to optimal structural configurations (Figure 4G). Further cellular thermal shift assay (CETSA) assays confirmed the robust binding between **B7** and CD155 (Figure 4H,I).

To explore whether **B7** exerted antitumor effects by inhibiting CD155, the impact of **B7** on viability, migration, and invasion was assessed after overexpression of CD155 in GSCs. The results showed that CD155 overexpression abolished the ability of **B7** to inhibit GSC viability and promote apoptosis (Figure 4J–L, all *P* < 0.01 vs corresponding vehicle). Consistently, in scratch and transwell migration assays, the inhibitory effect of **B7** on GSC migration was diminished under conditions of CD155 overexpression (Figure 4M–P, all *P* < 0.05 vs corresponding vehicle). Analogously, the restraint of **B7** on GSC invasion was attenuated upon CD155 overexpression (Figure 4Q,R, all *P* < 0.05 vs corresponding vehicle). Overall, these findings highlight the pivotal role of **B7** in targeting CD155 to impede GBM progression.

### 2.7 Compound **B7** Enhances the Cytotoxicity of NK‐92 Cells by Targeting CD155 in a Coculture Model

**Figure 5:**
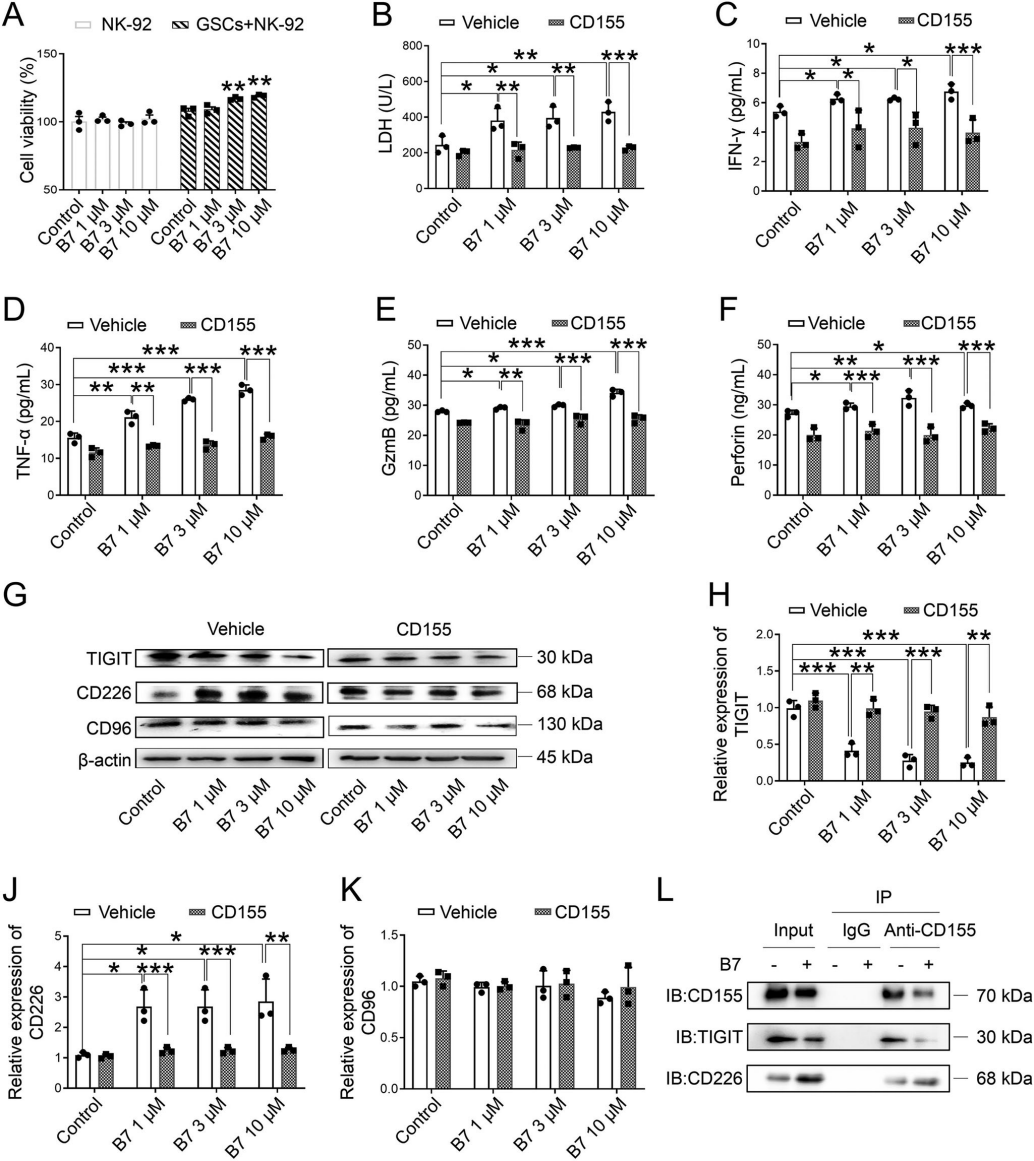
Effects of **B7** on the cytotoxicity of NK‐92 cells in a coculture environment. A) Viability of NK‐92 cells cultured alone and within the coculture environment with GSCs. B–F) Levels of lactate dehydrogenase (LDH) (B), interferon‐gamma (IFN‐γ) (C), tumor necrosis factor‐alpha (TNF‐α) (D), granzyme B (GzmB) (E), and perforin (F) released from NK‐92 cells cocultured with GSCs. G–K) Representative images (G) and quantification of TIGIT (H), CD226 (J), and CD96 (K) protein expression using Western blotting analysis. L) Co‐immunoprecipitation (co‐IP) analysis of the interaction of CD155 with TIGIT and CD226. One‐way ANOVA followed by Tukey's post hoc test was employed to analyze group differences. The results were expressed as mean ± SD, *n* = 3. ^*^ *P* < 0.05, ^**^ *P* < 0.01, ^***^ *P* < 0.001 vs Control or corresponding vehicle.

In the glioma TIME, NK cells have emerged as promising therapeutic effectors against GBM heterogeneity, attributable to their nonspecific cytolysis activities and their role in orchestrating tumor immune responses by releasing a spectrum of cytokines.^[^ ^46^ ^]^ Building on evidence suggesting that NK cells may target cancer stem cells,^[^ ^47^ ^]^ a coculture system of GSCs and NK‐92 cells was established to investigate the immunomodulatory effects of **B7** in the glioma TIME. Initial viability assessment revealed that **B7** treatment did not significantly affect NK‐92 cells in monoculture but markedly enhanced their survival when cocultured with GSCs (**Figure** **5**A, both *P* < 0.01 vs Control).

To evaluate **B7** on NK cell cytotoxicity in the TIME, LDH release, interferon‐gamma (IFN‐γ), tumor necrosis factor‐alpha (TNF‐α), granzyme B (GzmB), and perforin levels were measured. **B7** treatment significantly elevated the levels of LDH, IFN‐γ, TNF‐α, GzmB, and perforin, thereby augmenting the cytotoxic activities of NK‐92 cells (Figure 5B–F, all *P* < 0.05 vs Control). CD155 is overexpressed in tumors and involved in tumor immune escape by binding to receptors such as CD226, CD96, and TIGIT, thereby influencing the behavior of immune cells, including NK cells.^[^ ^48^, ^49^, ^50^ ^]^ Under CD155 overexpression, these cytokine secretions regulated by **B7** were significantly reduced (Figure 5B–F, all *P* < 0.05 vs corresponding vehicle).

Further Western blot analysis demonstrated that **B7** treatment reduced TIGIT expression and increased CD226 expression (Figure 5G–J, all *P* < 0.05 vs Control), but these alterations were reversed by CD155 overexpression (Figure 5G–J, all *P* < 0.01 vs corresponding vehicle). Meanwhile, neither **B7** treatment nor CD155 overexpression significantly altered CD96 expression (Figure 5G,K). Protein interaction assays revealed that **B7** treatment weakened the binding affinity of CD155‐TIGIT and strengthened the binding of CD155‐CD226 (Figure 5L). Consequently, these findings underscore that **B7** enhances the cytotoxicity of NK‐92 cells by targeting CD155‐mediated immune checkpoint interactions in a coculture system.

### 2.8 Compound **B7** Exhibits Antitumor Effects and Modulates the Immune Microenvironment via CD155 Inhibition in GSCs

**Figure 6:**
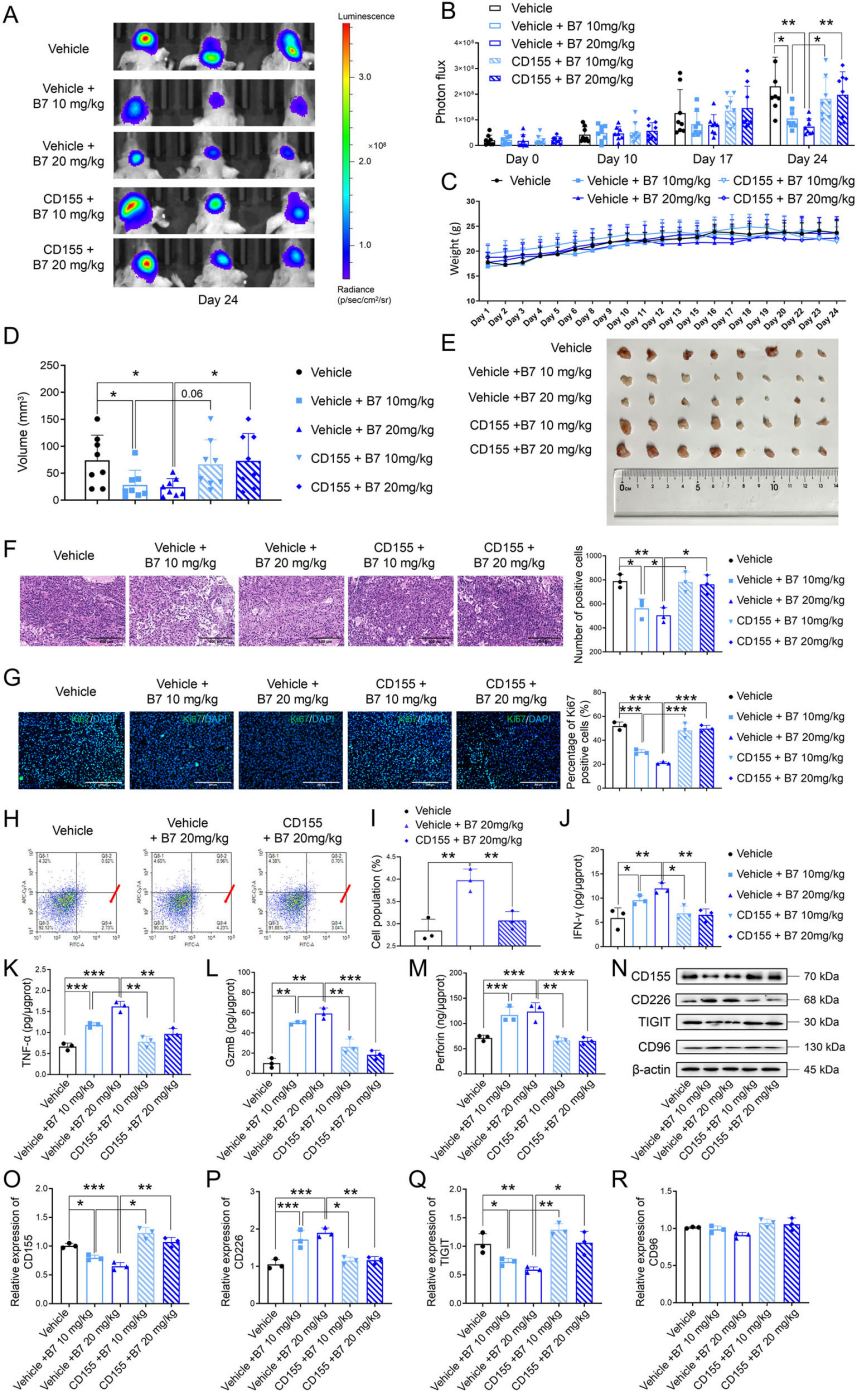
*In vivo* effects of **B7** on tumor progression and regulation of the immune microenvironment associated with CD155. A) Representative bioluminescent images of nude mice, *n* = 3. B) quantification of photon flux of nude mice intracranially implanted with GSCs‐Luc2 cotransfected with either the empty vehicle or CD155 overexpression plasmid, *n* = 8. C) Body weight changes of these nude mice, *n* = 8. D,E) Quantification of tumor volume (D) and tumor images (E) in the nude mouse brains, *n* = 8. F) H&E staining and quantification of tumor tissues, *n* = 3, bar: 400 µm. G) Immunohistochemical staining and quantification for Ki67 in tumor tissues of nude mice, *n* = 3, bar: 200 µm. H,I) Representative flow cytometry images (H) and quantification (I) of CD3^−^/CD49b^+^ NK cells in different groups of tumor tissues. J–M) The IFN‐γ levels (J), TNF‐α (K), GzmB (L), and perforin (M) levels in tumor tissues of nude mice, *n* = 3. N–R) Representative images (N) and quantification of CD155 (O), CD226 (P), TIGIT (Q), and CD96 (R) protein expression using Western blotting analysis, *n* = 3. One‐way ANOVA and Tukey's post hoc test were used to discern intergroup differences. The results were presented as mean ± SD. ^*^ *P* < 0.05, ^**^ *P* < 0.01, ^***^ *P* < 0.001 versus corresponding vehicle.

To further validate the antitumor mechanism of **B7** *in vivo*, a GBM xenograft model with CD155 genetic modification was established. Fluorescently labeled GSCs (GSCs‐Luc2) transfected with either the vehicle or CD155 overexpression plasmid were intracranially implanted into BALB/c nude mice. Photometric flux quantification revealed that **B7**, at the doses of 10 and 20 mg kg^−1^, significantly inhibited tumor growth (**Figure** **6**A,B, both *P* < 0.05 vs vehicle); however, this effect was reversed by CD155 overexpression (Figure 6A,B, both *P* < 0.05 vs corresponding vehicle). These mice showed stable body weights through the experimental period, suggesting favorable tolerability of **B7** (Figure 6C). Excised tumors consistently corroborated the significant tumor‐suppressing activity of **B7** (Figure 6D,E, both *P* < 0.05 vs vehicle), which was attenuated by CD155 overexpression (Figure 6D,E, *P* = 0.06 vs vehicle + **B7** 10 mg kg^−1^, *P* < 0.05 vs vehicle + **B7** 20 mg kg^−1^). H&E staining demonstrated that overexpressed CD155 hindered the efficacy of **B7** in controlling tumor progression (Figure 6F, all *P* < 0.05 vs corresponding vehicle). Parallel immunofluorescence analysis for Ki67 confirmed similar findings, with **B7**‐mediated inhibition of tumor proliferation being suppressed by CD155 (Figure 6G, all *P* < 0.001 vs corresponding vehicle).

Building on our prior *in vitro* experiments of **B7**‐induced NK cell recruitment by targeting CD155 in GSCs, we evaluated NK cell infiltration in tumor tissues. Flow cytometry revealed a significant increase in the number of CD3^−^/CD49b^+^ NK cells following **B7** treatment, whereas CD155 overexpression under **B7** treatment resulted in a significant decrease in this effect (Figure 6H,I, both *P* < 0.01 vs corresponding vehicle). Notably, **B7** did not alter the populations of T cells, B cells, macrophages, and monocytes with or without CD155 overexpression (Figure S6, Supporting Information), suggesting that **B7** exerts its therapeutic effects through a selective modulation of NK cell recruitment in the glioma TIME.

ELISA analysis of tumor tissues revealed that **B7** significantly enhanced IFN‐γ, TNF‐α, GzmB, and perforin levels (Figure 6J–M, *P* < 0.05 vs vehicle), whereas CD155 overexpression diminished this immunostimulatory functionality of **B7** (Figure 6J–M, all *P* < 0.05 vs corresponding vehicle). Western blot assessment illustrated the effects of CD155 on **B7** modulation of CD155, CD226, and TIGIT protein expression in tissues. Although **B7** reduced CD155 and TIGIT protein expression and increased CD226 protein levels (Figure 6N–Q, all *P* < 0.05 vs vehicle), these effects were reversed by CD155 overexpression (Figure 6N–Q, all *P* < 0.05 vs corresponding vehicle). CD96 expression remained unchanged in tissues under **B7** treatment or CD155 overexpression (Figure 6N,R). These results are consistent with prior *in vitro* studies involving GSCs and NK‐92 cell coculture. Collectively, in the GSCs‐Luc2 nude mouse xenograft model, **B7** exhibited robust antitumor activities and immunostimulatory effects, primarily through CD155 inhibition.

### 2.9 Specific Amino Acid Mutations in CD155 Affect the Affinity Binding to **B7**

**Figure 7:**
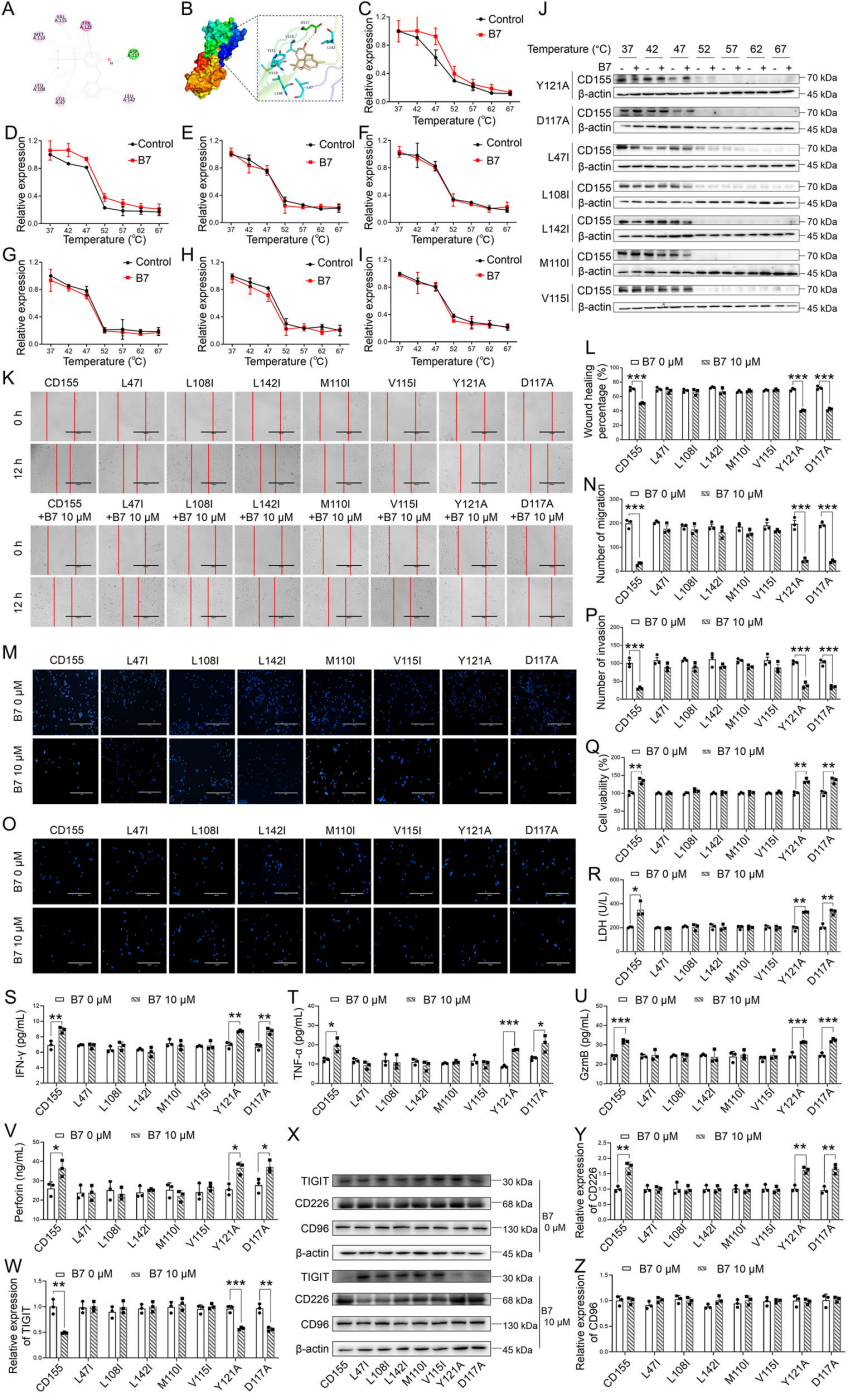
Mutations in CD155 for specific binding to **B7**. A) Key amino acids in the **B7**‐CD155 complex using molecular dynamics analysis. B) Schematic diagram of the **B7**‐CD155 complex and the locations of key amino acids. C–J) Fitting curve of Y121A (C), D117A (D), L47I (E), L108I (F), L142I (G), M110I (H), and V115I (I) and representative Western blotting images (J) demonstrating **B7**‐CD155 binding efficiency using CETSA. K,L) Representative images (K) and quantification (L) of the effects of mutations on CD155 function and the interaction between **B7** and CD155 in scratch assays, bar: 400 µm. M,N) Representative images (M) and quantification (N) of the influence of mutations on migration of **B7** in transwell migration assays, bar: 400 µm. O,P) Representative images (O) and quantification (P) of the influence of mutations on invasion of **B7** in transwell invasion assays, bar: 400 µm. Q) Viability of NK‐92 cells within the coculture environment with GSCs overexpressing CD155 WT and mutant plasmids. R–V) Levels of LDH (R), IFN‐γ (S), TNF‐α (T), GzmB (U), and perforin (V) released from NK‐92 cells cocultured with GSCs overexpressing CD155 WT and mutant plasmids. W–Z) Representative images (X) and quantification of TIGIT (W), CD226 (Y), and CD96 (Z) protein expression using Western blotting analysis. One‐way ANOVA, followed by Tukey's post hoc test, was used for multiple group comparisons. Two‐tailed Student's *t*‐test was employed for two‐group comparisons. The results were presented as mean ± SD, *n* = 3. ^*^ *P* < 0.05, ^**^ *P* < 0.01, ^***^ *P* < 0.001 versus corresponding control.

To elucidate the functional amino acid binding sites mediating the interaction between **B7** and CD155, MD simulations were employed to determine the optimal conformation of the **B7**‐CD155 complex (**Figure** **7**A,B). The molecular mechanics/Poisson–Boltzmann surface area scheme showed the biophysical basis of the molecular recognition of **B7** with CD155. The hydrophobic interaction energy, comprising the intermolecular van der Waals (Δ*G*_vdw_ = −104.573 kJ mol^−1^) and nonpolar solvation energy (Δ*G*_np_ = −10.351 kJ mol^−1^), displayed the largest binding energy, suggesting they are the major driving force for the **B7**‐CD155 complex (Table S8, Supporting Information). According to the conformation of the **B7** within the CD155 pocket, the residues of Leu47, Leu108, Met110, Val115, Asp117, Tyr121, and Leu142 were identified as crucial residues for **B7** stabilization inside the pocket of CD155. To experimentally verify their involvement in the **B7**‐CD155 complex stabilization, a series of CD155 protein expression plasmids harboring amino acid mutations at these sites were generated. Specifically, Leu (L) was mutated to Ile (I) [L47I, L108I, and L142I]; Met (M) and Val (V) were mutated to Ile (I) [M110I and V115I]; and Tyr (Y) and Asp (D) were mutated to Ala (A) [Y121A, and D117A].

In the CETSA assay, the Y121A and D117A mutations demonstrated robust stability with **B7** (Figure 7C,D,J), whereas the L47I, L108I, L142I, M110I, and V115I mutations did not (Figure 7E–J). Functional characterization through cellular scratch, migration, and invasion assays revealed that none of the mutations (L47I, L108I, L142I, M110I, V115I, Y121A, and D117A) impaired the ability of CD155 to promote migration and invasion of GSCs (Figure 7C–H). Importantly, upon **B7** treatment, the Y121A and D117A mutations retained migration and invasion promotion capabilities comparable to wild‐type CD155, suggesting a minimal impact on **B7** functionality by these amino acid substitutions (Figure 7K–P, *P* < 0.001 vs corresponding control). In contrast, the **B7**‐mediated inhibitory effect on GSC migration and invasion remained largely unaffected in the mutant groups carrying L47I, L108I, L142I, M110I, and V115I mutations, as compared to the wild‐type (Figure 7K–P).

Furthermore, GSCs overexpressing wild‐type and mutant CD155 plasmids were cocultured with NK‐92 cells, respectively. Wild‐type CD155, as well as the Y121A and D117A mutant groups, enhanced NK‐92 cytotoxicity after **B7** treatment, as evidenced by improved NK‐92 cell viability, increased LDH release, and elevated IFN‐γ, TNF‐α, GzmB, and perforin levels (Figure 7Q–V, *P* < 0.05 vs corresponding control). Meanwhile, **B7** treatment reduced the expression of TIGIT and increased the expression of CD226 in overexpressing wild‐type, Y121A, and D117A mutant groups (Figure 7W–Y, *P* < 0.01 vs corresponding control), with no significant effect on CD96 expression (Figure 7X,Z). Whereas, L47I, L108I, L142I, M110I, and V115I mutations did not affect **B7**‐mediated NK‐92 cytotoxicity (Figure 7Q–Z). In addition, all mutations (L47I, L108I, L142I, M110I, V115I, Y121A, and D117A) did not impair the effect of CD155 on NK‐92 cell activity following overexpression in GSCs (Figure 7Q–Z). These findings collectively identify L47, L108, L142, M110, and V115 residues as crucial structural elements within the **B7**‐CD155 interaction interface.

## 3 Discussion

Our study identified a novel anti‐GBM compound, namely **B7**, through rational optimization of **CFT**. As the most favorable derivates among 29 structural modified **CFT** derivatives, **B7** demonstrated remarkably anti‐GBM effects in anti‐glioma assays (IC_50_ = 3.43–14.60 µm) and showed selective inhibition of GSC proliferation (IC_50_ = 6.10 ± 0.20 µm), migration, and invasion. Notably, **B7** at 10 and 20 mg kg^−1^ effectively suppressed tumor growth in situ in two intracranial xenograft models involving nude mice implanted with GBM cells and GSCs. Mechanistically, extensive *in vitro* and *in vivo* studies revealed that **B7** inhibited CD155‐mediated GSC migration and invasion while simultaneously bolstering NK cell cytotoxicity and modulating the TIME via CD155/TIGIT/CD226 interactions. Specifically, L47, L108, L142, M110, and V115 residues were identified as crucial sites for **B7**’s functional binding to CD155. These findings suggest the promise of **B7** as a therapeutic agent for gliomas, with dual mechanistic actions targeting both GSCs and the TIME.

Through the analysis of SARs, we have unveiled the importance of the 2,4,4‐trimethylpentan‐2‐yl chain, the length and branching of the side chain in Ring I, and the diverse effects of the position and type of other substituents on its biological activity. Notably, the unique 2,4,4‐trimethylpentan‐2‐yl chain is crucial for supporting the anti‐glioma activity profile of these compounds. Furthermore, the introduction of methyl groups enhances activity, with the R_2_ position in Ring II identified as the optimal substitution site. In the case of dimethyl substitution, the maximal outcomes are achieved when targeting both the R_2_ and R_4_ positions concurrently. This constitutes a novel discovery that stands as an untouched field within prior studies on the anticancer potential of **CFT**.^[^ ^36^, ^37^, ^38^ ^]^

GSCs exhibit a remarkable self‐renewal capacity that orchestrates malignant glioma progression through multiple oncogenic mechanisms, including chemoresistance acquisition and metastatic dissemination. Their immunomodulatory properties foster an immunosuppressive TIME that facilitates immune evasion.^[^ ^12^ ^]^ These pathological characteristics contribute to current therapeutic resistance in eradicating GBM and represent the primary determinant of tumor recurrence. For example, **TMZ**, the first‐line chemotherapeutic agent for glioma, demonstrates limited efficacy due to GSC‐mediated chemoresistance.^[^ ^51^ ^]^ This treatment difficulty has promoted intensified investigation into GSC‐targeted therapies. In our study, **B7** inhibited diverse GBM cell growth with an excellent safety profile both *in vitro* and *in vivo*. Notably, while **CFT**, a leading drug known to selectively target GSCs,^[^ ^37^ ^]^ has been reported, **B7** demonstrated double its inhibitory potency against GSCs growth *in vitro*. At equivalent concentrations, **B7** surpassed **CFT** in promoting GSC apoptosis and suppressing their proliferation and spheroid formation properties. Meanwhile, **B7** exhibited anti‐migration and anti‐invasion capabilities toward GSCs comparable to those of **CFT**. Furthermore, with BBB penetration properties, **B7** treatment markedly inhibited GSC‐mediated tumor progression and ameliorated immunohistochemical changes in tumor tissues in the intracranial xenograft model *in vivo*. However, ADMET prediction indicates that **B7** may function as a CYP2D6 inhibitor, potentially necessitating dose adjustments in combination therapies. Nevertheless, this concern could be mitigated by **B7**’s relatively low IC_50_ values, the reassuring safety data derived from **CFT** derivatives, and the well‐established combination therapy profile of its parent compound **CFT**.^[^ ^52^, ^53^ ^]^ Regarding physicochemical optimization, the predicted low solubility and high plasma protein binding of **B7** suggest formulation strategies such as lipid–polymer hybrid nanoparticles or site‐specific pegylation to enhance its bioavailability while maintaining target engagement.

A novel finding from our study reveals that **B7** targeted immune molecule interactions based on GSC‐regulated TIME. Subsequent to the 2018 Nobel Prize in Physiology or Medicine announcement,^[^ ^54^ ^]^ which involved the development of cancer therapy by regulating the suppression of negative immune, strategies on GSCs have surfaced as a promising avenue for cancer management. Distinct from **CFT**, which exerts glioma‐inhibiting effects by regulating KLF13‐mediated epigenetic remodeling, **B7** uniquely modulates TIME through GSC‐specific immunostimulatory mechanisms. In mixed cocultures that incorporated GSCs with NK‐92 cells, **B7** increased NK cell‐mediated tumor‐killing activity by inducing GSCs apoptosis via LDH, GzmB, and perforin release. Simultaneously, **B7** bolstered pro‐inflammatory cytokines IFN‐γ and TNF‐α secretion, further promoting the anti‐GSC response. Notably, in our GSCs‐Luc2 mouse xenograft model, **B7** exhibited excellent anti‐glioma properties through selective intra‐tumoral NK cell accumulation via an immunostimulatory mechanism, making the first demonstration of the pivotal role of **B7** in targeting NK cell‐linked TIME in gliomas.

Mechanistic investigations identified CD155, a type I transmembrane glycoprotein belonging to the immunoglobulin (Ig) superfamily,^[^ ^55^ ^]^ as the crucial **B7**‐interacting molecule responsible for reversing GSC‐associated immune suppression. CD155 overexpression is a hallmark observed in multiple malignancies, including lung adenocarcinoma,^[^ ^56^ ^]^ colorectal cancer,^[^ ^57^ ^]^ pancreatic cancer,^[^ ^58^ ^]^ and high‐grade gliomas, such as GBM (Grade IV),^[^ ^45^, ^59^ ^]^ and promotes tumor migration and invasion. In our study, overexpression of CD155 attenuated the inhibitory effect of **B7** on spheroid formation, migration, and invasion of GSCs, as well as the attenuation of the immuno‐stimulatory responses. These were manifested through decreased NK cell cytotoxicity toward GSCs and dampened release of lytic and pro‐inflammatory factors. These observations were consistently replicated *in vitro* through coculturing GSCs with NK‐92 cells under conditions of CD155 overexpression. Furthermore, *in vivo* experiments using GSC‐implanted nude mice and co‐transfected with CD155 overexpression plasmid also confirmed diminished anti‐glioma effects of **B7**. These immunomodulatory mechanisms of **B7** were intertwined with the CD155/TIGIT/CD226 interaction in the TIME. CD155 functions as a key ligand for NK cell‐resident inhibitory receptor TIGIT and the activating receptor CD226, modulating NK cell‐mediated cytotoxicity and IFN‐γ production via β‐Arrestin 2/nuclear factor kappa B subunit 1 signaling pathways.^[^ ^60^ ^]^ Notably, CD155 displays differential binding affinities to these receptors, preferentially engaging with TIGIT.^[^ ^61^ ^]^ In line with protein interaction analyses, **B7** was found to interfere with CD155‐TIGIT binding while promoting its association with CD226, thereby concerting immunosuppressive signals into immune activation.

Our study explored the intricate interplay between **B7** and CD155 through the integrative applications of MD simulations, thermal stability assessments, and amino acid point mutation experiments. The MD simulations revealed a remarkably stable complex formation, with **B7** inserted into a pocket composed of seven amino acids. This unique binding architecture is characterized by a low total binding free energy between **B7** and CD155, solidifying the effectiveness of their binding. Further dissection of the free energy landscape suggests hydrophobic interactions as the primary driving force for the **B7**‐CD155 interaction, contributing a substantial −114.924 kJ mol^−1^ toward their binding affinity. By assessing the thermal stability and bioactivity of the protein, we confirmed L47, L108, L142, M110, and V115 as pivotal sites within the seven‐amino acid pocket, providing a more stable anchorage for **B7**. The N‐terminal immunoglobulin variable (IgV) domain of CD155 is crucial for binding to TIGIT and CD226.^[^ ^62^ ^]^ Within the IgV domain of CD155, the residues L47, L108, M110, and V115 exhibited a close association with **B7**, highlighting their potential significance as key determinants in the modulation of **B7** on CD155‐TIGIT and CD155‐CD226 interactions. Despite their looser affiliations as indicated by thermal stability and protein biofunction studies, Y121 and D117 cannot be entirely disregarded, as they hold supporting roles within the binding pocket. Recently, a study has revealed the functional impact of polymorphisms in the CD155 gene. The single nucleotide polymorphism rs1058402G>A, causing a mutation from Ala to Thr at residue 67 of CD155, increased the binding affinity of CD155 for CD226 and generated stronger tumor immunosuppressive responses, inhibiting small‐cell lung cancer development.^[^ ^63^ ^]^ However, in our study, the L47I, L108I, L142I, M110I, V115I, Y121A, and D117A mutations had no effect on CD155's function in glioma, suggesting that alterations at these sites mainly interfere with **B7**‐specific binding rather than with receptor basal activity.

Immunotherapeutic approaches targeting the CD155/TIGIT axis are increasingly popular, with clinical trials harnessing genetically engineered NK cells as therapeutic vectors.^[^ ^64^ ^]^ A Phase I/II clinical study enrolling 61 patients with recurrent WHO grade IV malignant glioma demonstrated higher survival rates at 24 and 36 months among those treated with recombinant, nonpathogenic polio‐rhinovirus chimera specifically designed to recognize CD155, compared to historical controls.^[^ ^65^ ^]^ Extended follow‐up showed sustained survival rates of 21% and 18% at 60 and 72 months, respectively. Correlating our present study, the pursuit of small molecule candidates that target the CD155‐linked TIME could hold promise in glioma immunotherapy.

Although our findings suggest that **B7** holds promising potential in targeting GSCs and modulating antitumor immunity, several critical knowledge gaps warrant further investigation. First, the molecular mechanisms underlying potential resistance to **B7**‐based therapies remain incompletely defined, particularly regarding intrinsic pathways in GSCs. Second, the synergistic effect of **B7** with standard‐of‐care therapies (e.g., **TMZ**, immunotherapy) requires systematic evaluation through combination treatment models. Third, comprehensive preclinical pharmacokinetic and extended toxicology studies of **B7** remain essential for clinical translation. Additionally, from a computational perspective, our current analysis of **B7**‐CD155 interactions could be enhanced through multiscale modeling approaches. Specifically, Density Functional Theory calculations could elucidate microelectronic structural dynamics during ligand–receptor binding, including electrostatic potential mapping of key binding residues and electron density redistribution patterns during complex formation, providing atomic‐level detail of **B7**’s conformational changes upon CD155 binding.

## 4 Conclusion

In summary, our study identified a novel anti‐GBM compound **B7** from the structural optimization of **CFT**. **B7** exhibited promising antitumor effects against GBM both *in vitro* and *in vivo*. Importantly, **B7** had a distinct anti‐GBM mechanism of action that targeted CD155 via interaction at five key binding interfaces within the **B7**‐CD155 complex, while simultaneously modulating the TIME through the CD155/TIGIT/CD226 axis. Collectively, our work highlights the potential of **B7** as a therapeutic agent for gliomas targeting CD155, with dual mechanisms that synergize to inhibit tumor progression and enhance antitumor immunity.

## 5 Experimental Section

### Chemistry

All reagents were purchased from J&K Scientific (Beijing, China) and Bide Pharmatech Ltd. (Shanghai, China) and utilized without further processing. All reactions were monitored using thin‐layer chromatography with an aluminum TLC plate 60F254D (Merck Millipore, USA). Biotage initiator+ was employed for the microwave reaction. Compounds were isolated and purified using Combiflash Rf+ (Teledyne Isco, USA) and Slica Flash Column (Santai Technologies, China). LC‐MS analysis was conducted using a Shimadzu LC‐MS 2020 system with an electrospray ionization source (ESI) and a single‐quadrupole mass analyzer. A Shim‐pack VP‐ODS (2.0 mm × 150 mm, 5 µm) was used, with a gradient of solvent B ranging from 10% to 90% and a flow rate of 0.5 mL min^−1^. Solvent A was composed of 0.1% formic acid in water, while solvent B consisted of 0.1% formic acid in acetonitrile. MS spectra were obtained in either negative or positive ion mode, with a scan range of m/z 100–800. High‐resolution mass spectra were performed using LTQ Orbitrap XL (Thermo Scientific, USA). HPLC analysis was performed on an Agilent 1260 using XDB‐C18 (4.6 mm × 150 mm, 5 µm) or an Agilent 1200 using EC‐C18 (4.6 mm × 150 mm, 4 µm) columns. The mobile phases comprised acetonitrile and water, with a flow rate of 0.8 mL min^−1^. The purity of all target compounds, as determined by analytical HPLC, was greater than 95%. ^1^H NMR and ^13^C NMR spectra were recorded in CDCl_3_ or DMSO‐*d*_6_ (Cambridge Isotope Laboratories, USA) using a Bruker‐400 MHz, Bruker‐500 MHz or Bruker‐600 MHz (Bruker Bioscience, USA) spectrometer.

Detailed synthesis schemes and characterization data of compound structures were described in the Supporting Information (Methods section).

### Cell Culture

The following cell lines were acquired from various sources. The human GBM cell lines: LN229, U118‐MG, U251‐MG, and U87‐MG, along with NK‐92 cells, normal hepatocyte epithelial cell THLE‐2, and mouse hippocampal neuronal cell HT22, were obtained from Procell Life Science & Technology Co., Ltd (Wuhan, China). U‐87 MG‐Luc2, rat cardiomyocytes H9c2(2‐1), African green monkey kidney cell Vero, and human embryo kidney cell HEK‐293 were obtained from the American Type Culture Collection (ATCC). The human GSCs were obtained from QINGQI (Shanghai) BIOTECHNOLOGY DEVELOPMENT Co., LTD. For cultivation, the U251‐MG, U87‐MG, LN229, U118‐MG, GSCs, HT22, H9c2(2‐1), and HEK‐293 cells were grown in Dulbecco's modified Eagle medium (DMEM) (Procell, China) containing 10% fetal bovine serum (FBS) (Gibco, USA). Vero cells were cultured in MEM medium supplemented with 10% FBS. NK‐92 and THLE‐2 cells were cultured in NK‐92 and THLE‐2 Specific Culture Medium (CM‐0530 and CM‐0833, Procell, China) at 37 °C with 5% CO_2_. All cells were maintained in monolayer culture at 37 °C under a humidified atmosphere containing 5% CO_2_.

### Animals and Xenograft Models of Gliomas

Five‐week‐old male BALB/c nude mice (Zhishan Health Medical Research Institute Co., LTD, Beijing, China) were used to determine the *in vivo* drug effects of **B7**. For intracranial xenograft models, 1 × 10^5^ U‐87 MG‐Luc2 cells and GSCs transferred with empty vehicle or CD155 overexpression plasmid were infused into the frontal cortex (coordinates: 1 mm rostral to bregma, 1.5 mm lateral to the midline, and 2.7 mm deep) using a stereotaxic apparatus (RWD Life Science Co., Ltd., China). In the U‐87 MG‐Luc2 xenograft model, mice were randomized into five groups: control, 10 mg kg^−1^ **B7**, 20 mg kg^−1^ **B7**, 20 mg kg^−1^ **CFT**, and 20 mg kg^−1^ **TMZ**. **B7**, **CFT**, and **TMZ** (purchased from MedChemExpress, HY‐17364) were dissolved in a solution comprising 10% Polyoxyl 15 Hydroxystearate (HS‐15) + 90% normal saline. 10% HS‐15+ 90% normal saline served as a control. In the GSCs xenograft models, there were five groups: vehicle, vehicle + 10 mg kg^−1^ **B7**, vehicle + 20 mg kg^−1^ **B7**, CD155 + 10 mg kg^−1^ **B7,** and CD155 + 20 mg kg^−1^ **B7**. The mice received a total of 21 intravenous doses of the compounds or control once daily, 6 days per week. Then the photon flux value of the bioluminescence signal was observed and recorded using the IVIS SpectrumCT *In Vivo* Imaging System (PerkinElmer, USA). To determine the drug toxicity of **B7**, eight‐week‐old male C57BL/6 mice (Zhishan Health Medical Research Institute Co., LTD, Beijing, China) were injected with a single tail vein dose and monitored for over one week. **B7** was administered at doses of 0, 40, 80, 100, 120, and 200 mg kg^−1^, with each group containing two female and one male mouse. The Experimental Animal Ethical Inspection of the Institute of Medicinal Biotechnology authorized the animal experiments (No. IMB‐20230424D_6_01, IMB‐20240415D_6_01, and IMB‐20250113D_6_01).

### Cell Viability Assay

Following the 72 h treatment of cells with compounds or PBS, the MTS (Promega, USA) mixture was used for colorimetry.^[^ ^64^, ^65^, ^66^, ^67^, ^68^ ^]^ In brief, cells from each well were incubated with 90 µL basal DMEM and 10 µL MTS working fluid at 37 °C for 2 h. The absorbance was read at 490 nm using a multifunctional microplate photometer (TECAN, Switzerland). The concentration of compounds that reduced cell proliferation by 50% (IC_50_) was calculated.

### Assessment of BBB Permeability

To evaluate the *in vivo* BBB permeability of compounds **B7** and **B8**, C57BL/6 mice underwent a single tail vein injection with 20 mg kg^−1^ **B7** and **B8**. Five minutes postinjection, tissues from blood, brain, kidney, liver, heart, spleen, and lung were collected. Tissue samples were homogenized in methanol and centrifuged at 10 000 × *g* for 10 min to obtain the supernatant. Compound preparations involved dissolving **B7** and **B8** in methanol at 1 mg mL^−1^, followed by dilutions (**B7**: 0.1 mg mL^−1^, 50 000 ng mL^−1^, 40 000 ng mL^−1^, 20 000 ng mL^−1^, 10 000 ng mL^−1^; **B8**: 40 000 ng mL^−1^, 20 000 ng mL^−1^). The Agilent 1200 HPLC system was equipped with a C18 column (150 mm × 4.6 mm, 4 µm), a mobile phase of 90% acetonitrile and 10% water (isocratic elution, 0.8 mL min^−1^ flow rate), and a detection wavelength of 230 nm to determine the retention times of **B7** and **B8**. The presence or absence of **B7** and **B8** peaks in the plasma and tissue samples was determined by comparing the chromatograms to those of blank control samples.

### TUNEL Assay

GSCs were seeded onto coverslips and cultured for 24 h before exposure to **B7** for 48 h. After fixation and washing, 0.1% Triton X‐100 in PBS was used to enhance the membrane permeability of GSCs. The GSCs were washed thrice with PBS and incubated with the TUNEL reaction mixture (Wuhan Pricella Biotechnology Co., Ltd., Wuhan, China) in a light‐shielded environment for 2 h. Following three additional washes with PBS, a DAPI staining solution was added to label the GSC nuclei. After mounting the coverslips with an antifluorescent quencher, images of TUNEL‐positive cells (green fluorescence) and nuclei (blue fluorescence) were captured using an EVOS FL Imaging System (Thermo Fisher Scientific, USA). The apoptotic content was quantified by counting the proportion of these positive cells to the total number of cells.

### Cell Transfections

The pcDNA3.1 constructs harboring CD155 WT and various CD155 mutants (L47I, L108I, L142I, M110I, V115I, Y121A, and D117A) were synthesized by Sangon Biotech (Shanghai, China). GSCs were transfected with the vehicle, CD155 WT, and mutant plasmids using Lipofectamine (Invitrogen, USA). Briefly, one day before transfection, 1 × 10^5^ cells per 24‐well were plated in 1 mL of DMEM+10% FBS medium to achieve 60–70% confluence at the time of transfection. Plasmids were diluted in DMEM without serum and gently mixed. The Lipofectamine was combined with the diluted DNA and left to stand for 20 min at room temperature. Subsequently, GSCs were cultured in a standard incubator for 24 h.

### Tumorsphere Formation Assay

GSCs (5000 cells per well) were seeded on 24‐well ultralow‐attachment plates (Corning, USA) and maintained in serum‐free DMEM/F12 containing 20 ng mL^−1^ EGF, 2% B27, and 20 ng mL^−1^ FGF for a duration of 7 days. On day 7 postdrug treatment, the tumorspheres were recorded and counted, specifically focusing on those with a diameter of at least 50 µm.

### Scratch Assay

The migration capacity of GSCs was assessed using the scratch assay. 2 × 10^5^ cells were plated into a 6‐well plate and allowed to grow until they reached confluence. A scratch wound was created using a 10 µL plastic pipette tip and any detached cells were removed by washing with medium. Then the cells were incubated in a serum‐free DMEM supplemented with or without **B7** for 24 h. The calculation of the wound healing area was performed as outlined below: wound healing area (%) = (*A*_0_–*A*_12_)/*A*_0_ × 100%, where *A*_0_ signals the original area and *A*_12_ signals the area 12 h post‐treatment.

### Transwell Migration and Invasion Assays

GSCs were suspended and seeded into the upper chambers of transwell plates. For invasion assays, the upper chambers were coated with Matrigel (BD Biosciences, USA). DMEM supplemented with compounds was added to both the upper and lower chambers. After 12 h of incubation, GSCs remaining in the upper chamber were removed using swabs, and the transwell plates were washed three times with DMEM. After fixation, the migrated and invaded cells were visualized and photographed under a light microscopy.

### RNA Isolation and qRT‐PCR Analysis

RNA was isolated from GSCs employing TRIzol reagent (CWBIO, China), adhering to the instructions. mRNA reverse transcription was performed using HiscriptIV RT SuperMix (Vazyme Biotech, Nanjing, China), followed by qRT‐PCR using ChamQ Universal SYBR qPCR Master Mix (Vazyme Biotech, Nanjing, China). The relative expression of mRNA was determined using the 2^−ΔΔCT^ method and normalized to GAPDH mRNA. The primer sequences for qRT‐PCR are listed in Table S9 of the Supporting Information.

### Western Blot

Western blot analysis was conducted to evaluate the expression levels of CD155, TIGIT, CD226, and CD96 in tissues and cells, according to previously described protocols.^[^ ^69^ ^]^ In brief, protein extraction was carried out using RIPA lysis buffer (CWBIO, China). Identical quantities of protein were fractionated via 10% sodium dodecyl sulfate‐polyacrylamide gel electrophoresis and transferred onto polyvinylidene fluoride membranes (Millipore, MA, USA) for antibody hybridization. The primary antibodies used included Anti‐CD155 (Rabbit mAb ab205304, 1:1000, Abcam), Anti‐TIGIT (Rabbit mAb NO.20350, 1:1000, ABclonal), Anti‐CD226 (Rabbit mAb NO.A23200, 1:1000, ABclonal), Anti‐CD96 (Rabbit mAb NO.A20547, 1:1000, ABclonal), Anti‐β‐actin (Mouse mAb #3700, 1:1000, Cell Signaling Technology (CST)), and the secondary antibodies were HRP‐conjugated goat anti‐rabbit IgG and anti‐mouse IgG (1:10 000, Gene‐Protein Link). The gray intensity of the images was analyzed using the Fusion‐FX6 Imaging System (Vilber Lourmat, France), with β‐actin levels serving as an endogenous control.

### Flow Cytometry

Tumor samples were thawed and resuspended in Cell Staining Buffer (Biolegend, CA, USA, 420201). For surface staining, cell suspensions were blocked with TruStain FcX PLUS (anti‐mouse CD16/32) antibody (Biolegend, 156604) for 10 min on ice and then stained with fluorescently labeled anti‐mouse antibodies for 45 min in Cell Staining Buffer on ice. The antibodies used included APC/Cyanine7 anti‐mouse CD3 antibody (Biolegend, 100221), FITC anti‐mouse CD49b (pan‐NK cells) antibody (Biolegend, 108905), APC anti‐mouse/human CD11b antibody (Biolegend, 101211), PE anti‐mouse/human CD45R/B220 antibody (Biolegend, 103207), and PE/Cyanine7 anti‐mouse F4/80 antibody (Biolegend, 123113). After staining, cells were washed with Cell Staining Buffer. Flow cytometry analysis was performed using NasoCyte 2060R flow cytometer (Agilent Technologies, USA). Data were analyzed using Novoexpress software (version 1.4.1, Agilent Technologies, USA).

### Cellular Thermal Shift Assay

GSCs were pretreated with **B7** or PBS for 24 h, then collected and suspended in PBS containing protease and phosphatase inhibitors. The cell suspension was divided into seven aliquots and subjected to heating at 37, 42, 47, 52, 57, 62, and 67 °C for 3 min, followed by three freeze–thaw cycles using liquid nitrogen. The supernatant was obtained by centrifuging at 12000 × *g* for 10 min at 4 °C. Subsequently, the protein levels under different temperatures and drug treatment conditions were assessed using Western blot analysis.

### A Coculture System of GSCs and NK‐92 Cells

Five thousand transfected GSCs were plated in the upper chamber, and 2 × 10^5^ NK‐92 cells were plated in the lower chamber in a 6‐well transwell culture plate. 2 mL of DMEM containing **B7** (1, 3, and 10 µm) was added to each well, ensuring uniform distribution of DMEM in both the upper and lower chambers. Subsequent experiments were performed after an additional 72 hours of incubation.

### Molecular Dynamics

The MD simulations were conducted utilizing the GROMACS 2022.1 program, equipped with the Amber14sb force field, maintained under conditions of constant temperature and pressure, while applying periodic boundary conditions. The molecules were positioned within a cubic box measuring 8.0 nm × 8.0 nm × 8.0 nm. The Amber14sb all‐atom force field was applied, alongside the TIP3P water model to solubilize both the protein and small molecule complexes. The visualization of results was achieved using the built‐in tools of GROMACS and the VMD (Visual Molecular Dynamics) software.

### H&E and Immunohistochemistry Staining

After fixation, dehydration, and paraffin embedding, the fresh tissues were sliced to a thickness of 5 µm. Sections were stained with hematoxylin and eosin (G1076, Servicebio, Wuhan, China). For immunohistochemical staining, tissue sections were hybridized with Anti‐Ki67 (Mouse mAb #P46013, 1:1000, CST) and Goat Anti‐mouse IgG‐Alexa Fluor 488 (1:200, Gene‐Protein Link). Nuclei were labeled with DAPI. The images were scanned using the panoramic MIDI digital section scanner (3DHISTECH, Budapest, Hungary) and analyzed using ImageJ software (version 1.8.0).

### Co‐Immunoprecipitation (Co‐IP)

The Pierce Classic Magnetic IP/Co‐IP Kit (88804, Thermo Scientific) was employed to perform the co‐IP experiments. Cells treated with or without **B7** were lysed using the provided lysis buffer and then centrifuged to obtain the supernatant. Subsequently, the supernatant was incubated with an Anti‐CD155 antibody (Rabbit mAb ab205304, 1:1000, Abcam) at 4 °C for 8 h. Following incubation, the immune complexes were allowed to bind to Protein A/G magnetic beads by gentle mixing at room temperature for 1 h. The beads underwent two washes with IP Lysis/Wash Buffer and one wash with distilled water. After eluting the complexes, Western blotting was performed to detect and quantify the protein levels of TIGIT and CD226.

### Bioinformatics Analysis

Based on the structural features of **B7**, the SwissTargetPrediction (http://swisstargetprediction.ch/) and Prediction (https://prediction.charite.de/subpages/target_prediction.php) databases were used to obtain the targets of **B7**’s active functional groups. GO and KEGG analyses were performed using the Metascape platform (https://metascape.org/), with statistical significance defined as *P* <0.01. The significant KEGG pathways and GO terms were presented as bubble charts using the bioinformatics platform (http://www.bioinformatics.com.cn).

### Statistical Analysis

Data plotting and analysis were executed using GraphPad Prism software (GraphPad Inc., La Jolla, CA, USA). Data normality was assessed using the Shapiro–Wilk normality test. For multigroup comparisons, statistical significance was determined using one‐way ANOVA with Tukey's post hoc test. For comparisons between two groups, unpaired Student's *t*‐tests were employed. For molecular biological experiments, each group consisted of three replicates. For *in vivo* experiments, each group comprised 6–8 mice. Results were presented as mean ± standard deviation (SD), with statistical significance considered at *P* < 0.05.

## Conflict of Interest

The authors declare no conflict of interest.

## Author Contributions

Y.W., T.S., and Z.C. conducted the experiment execution, data collection, and original draft. S.X., H.Y., and M.L. assisted in the chemical synthesis of experiments. S.K. assisted in the discovery studio. Z.L. established the project, directed the overall design, and provided funding support. R.L. directed the biological experiment design and provided funding support. X.P. contributed to per‐guidance. All authors have approved the final version of the manuscript.

## Supporting information



Supporting Information

## Data Availability

The data that support the findings of this study are available from the corresponding author upon reasonable request.
