# Supplementary material for: A Novel Class of Multi‐substituted Diaryl Scaffold Derivatives Inhibit Glioblastoma Progression by Targeting CD155
Source: Adv Sci (Weinh). 2025 Jun 10;12(32):e06688. doi: 10.1002/advs.202506688 (PMC12407320; doi:10.1002/advs.202506688)
Supplement: Supplementary file 1 — Supporting Information [file ADVS-12-e06688-s001.docx]

**Supplementary Information**

**A novel class of multi-substituted diaryl scaffold derivatives inhibit glioblastoma progression by targeting CD155**

*Yong-jian Wang, Ting Sun, Si-tu Xue, Zhong-di Cai, Hong Yi, Miao Lv, Shi-bo Kou, Rui Liu, ^*^ Xiao-zhong Peng, ^*^and Zhuo-rong Li^*^*

Y Wang, T Sun, S Xue, Z Cai, H Yi, M Lv, S Kou, R Liu, Z Li

State Key Laboratory of Bioactive Substance and Function of Natural Medicines, Institute of Medicinal Biotechnology, Chinese Academy of Medical Sciences & Peking Union Medical College, Beijing 100050, China

E-mail: [lizhuorong@imb.pumc.edu.cn](mailto:lizhuorong@imb.pumc.edu.cn); [liurui@imb.pumc.edu.cn](mailto:liurui@imb.pumc.edu.cn)

X Peng

Institute of Basic Medical Sciences, Chinese Academy of Medical Sciences & Peking Union Medical College, Beijing 100005, China

1. mail: pengxiaozhong@pumc.edu.cn

**Contents of Supplementary Information**

**[Table S1](#_Toc193446285)**[. Cell viability of compounds against THLE-2 cells 3](#_Toc193446285)

**[Table S2](#_Toc193446287)**[. Cell viability of compounds against H9c2(2-1) cells 3](#_Toc193446287)

**[Table S3](#_Toc193446288)**[. Cell viability of compounds against HT22 cells 4](#_Toc193446288)

**[Table S4](#_Toc193446289)**[. Cell viability of compounds against Vero cells 5](#_Toc193446289)

**[Table S5](#_Toc193446290)**[. Cell viability of compounds against HEK-293 cells 6](#_Toc193446290)

**[Table S6](#_Toc193446291)**[. ADMET prediction results of compounds 7](#_Toc193446291)

**[Table S7](#_Toc193446292)**[. Safety test of compound](#_Toc193446292) **[B7](#_Toc193446292)** *[in vivo](#_Toc193446292)* [7](#_Toc193446292)

**[Table S8.](#_Toc193446293)** [Binding energies of](#_Toc193446293) **[B7](#_Toc193446293)**[-CD155 complexes 8](#_Toc193446293)

**[Table S9.](#_Toc193446294)** [Sequences for qRT-PCR analysis 8](#_Toc193446294)

**[Figure S1.](#_Toc193446295)** [Qualitative detection of compound](#_Toc193446295) **[B7](#_Toc193446295)** [in the blood, brain, heart, liver, spleen, lung, and kidney tissues following](#_Toc193446295) *[in vivo](#_Toc193446295)* [administration. 10](#_Toc193446295)

**[Figure S2.](#_Toc193446296)** [Assessment of the blood-brain barrier penetration of compound](#_Toc193446296) **[B8](#_Toc193446296)**[. 11](#_Toc193446296)

**[Figure S3](#_Toc193446297)**[. Levels of aspartate aminotransferase (AST), alanine aminotransferase (ALT), lactate dehydrogenase (LDH), blood urea nitrogen (BUN), creatinine (CRE), and creatine kinase (CK) in the serum of mice. 11](#_Toc193446297)

**[Figure S4.](#_Toc193446298)** [CD155 serves as the target for compound](#_Toc193446298) **[B7](#_Toc193446298)**[. 14](#_Toc193446298)

**[Figure S5.](#_Toc193446299)** [Network pharmacological prediction for the treatment of glioma with](#_Toc193446299) **[B7](#_Toc193446299)**[. 14](#_Toc193446299)

**[Figure S6](#_Toc193446300)**[. Effect of](#_Toc193446300) **[B7](#_Toc193446300)** [treatment and CD155 modulation on T-cell, B-cell, macrophage, and monocyte populations in tumor tissues. 15](#_Toc193446300)

**[Figure S7.](#_Toc193446301)** [Original pictures of Western blotting analyses. 18](#_Toc193446301)

**[Supplementary Methods](#_Toc193446302)** [18](#_Toc193446302)

**[1. General procedure for preparation of phenyl compounds 3a-d](#_Toc193446303)** [18](#_Toc193446303)

**[2. General procedure for preparation of 4a-d](#_Toc193446304)** [19](#_Toc193446304)

**[3. General procedure for preparation of A1-A7, B1-B13 and CFT](#_Toc193446305)** [20](#_Toc193446305)

**[4. General procedure for preparation of 5a-d](#_Toc193446306)** [26](#_Toc193446306)

**[5. General Procedure for preparation of C1-C4](#_Toc193446307)** [27](#_Toc193446307)

**[6. The synthesis procedure for preparation of 6](#_Toc193446308)** [28](#_Toc193446308)

**[7. The synthesis procedure for preparation of 7](#_Toc193446309)** [29](#_Toc193446309)

**[8. General procedure for preparation of D1-D5.](#_Toc193446310)** [29](#_Toc193446310)

**[Figure S8-S51.](#_Toc193446311)** ^[1](#_Toc193446311)^[H NMR, MS,](#_Toc193446311) ^[13](#_Toc193446311)^[C NMR, HRMS and HPLC data of compound. 32](#_Toc193446311)

**Table S1**. Cell viability of compounds against THLE-2 cells

| **Compound** | **Cell viability** (%) | | | | |
| --- | --- | --- | --- | --- | --- |
|  | **6.25** µM | **12.5** µM | **25** µM | **50** µM | **100** µM |
| **A1** | 98.47 ± 1.03 | 103.93 ± 1.79 | 117.65 ± 2.19 | 115.10 ± 0.96 | 117.16 ± 2.13 |
| **A2** | 102.21 ± 4.93 | 107.95 ± 2.75 | 111.51 ± 1.78 | 75.20 ± 47.20 | 84.22 ± 12.02 |
| **A3** | 108.35 ± 14.86 | 109.37 ± 5.29 | 110.80 ± 1.02 | 99.58 ± 18.77 | 97.18 ± 16.54 |
| **A4** | 98.76 ± 2.89 | 94.12 ± 2.74 | 95.61 ± 3.18 | 92.69 ± 1.76 | 95.32 ± 3.35 |
| **A5** | 95.06 ± 1.44 | 99.65 ± 5.71 | 94.86 ± 5.35 | 91.63 ± 4.99 | 92.66 ± 3.09 |
| **A6** | 93.21 ± 3.35 | 89.05 ± 2.79 | 101.33 ± 3.19 | 99.87 ± 9.61 | 96.81 ± 0.60 |
| **A7** | 100.09 ± 4.92 | 86.10 ± 4.93 | 98.13 ± 10.65 | 99.11 ± 1.28 | 100.76 ± 1.08 |
| **B1** | 96.95 ± 3.46 | 98.76 ± 6.85 | 100.75 ± 3.97 | 105.56 ± 3.80 | 98.77 ± 2.14 |
| **B2** | 104.58 ± 3.71 | 103.67 ± 2.23 | 99.06 ± 4.70 | 101.66 ± 3.47 | 100.59 ± 2.85 |
| **B3** | 97.27 ± 2.75 | 106.42 ± 7.44 | 102.06 ± 8.66 | 96.63 ± 6.78 | 94.16 ± 5.98 |
| **B4** | 100.95 ± 6.44 | 100.53 ± 6.14 | 87.59 ± 4.36 | 91.05 ± 8.58 | 91.28 ± 7.13 |
| **B5** | 99.94 ± 3.09 | 93.86 ± 4.95 | 99.29 ± 2.92 | 100.50 ± 6.37 | 36.16 ± 6.75 |
| **B6** | 99.28 ± 5.84 | 111.71 ± 13.54 | 101.30 ± 9.56 | 87.89 ± 6.51 | 9.67 ± 1.12 |
| **B7** | 99.95 ± 2.76 | 103.80 ± 2.24 | 99.97 ± 6.21 | 61.41 ± 1.68 | 8.46 ± 0.16 |
| **B8** | 101.56 ± 5.90 | 103.01 ± 3.71 | 103.24 ± 5.39 | 29.35 ± 4.19 | 8.38 ± 0.04 |
| **B9** | 103.21 ± 0.95 | 99.02 ± 4.92 | 101.66 ± 6.74 | 105.46 ± 7.18 | 96.40 ± 3.89 |
| **B10** | 98.90 ± 5.85 | 108.02 ± 4.29 | 96.75 ± 4.09 | 55.04 ± 2.13 | 10.10 ± 0.81 |
| **B11** | 100.91 ± 12.23 | 89.84 ± 14.11 | 95.50 ± 17.95 | 95.11 ± 15.61 | 102.88 ± 12.12 |
| **B12** | 101.14 ± 19.20 | 106.82 ± 18.00 | 108.37 ± 6.50 | 98.06 ± 1.81 | 69.40 ± 13.96 |
| **B13** | 110.35 ± 13.67 | 92.22 ± 7.74 | 88.23 ± 7.10 | 80.91 ± 2.32 | 87.50 ± 13.57 |
| **C1** | 92.54 ± 10.30 | 93.89 ± 15.84 | 84.12 ± 0.99 | 86.10 ± 14.45 | 54.70 ± 9.63 |
| **C2** | 101.21 ± 7.68 | 95.39 ± 10.07 | 79.97 ± 10.05 | 86.53 ± 5.47 | 64.68 ± 7.59 |
| **C3** | 96.70 ± 3.58 | 95.50 ± 5.62 | 92.19 ± 6.58 | 85.75 ± 9.89 | 67.53 ± 10.89 |
| **C4** | 97.26 ± 1.98 | 95.26 ± 7.45 | 95.73 ± 1.58 | 51.48 ± 5.51 | 54.75 ± 1.58 |
| **D1** | 104.95 ± 3.37 | 104.55 ± 1.95 | 68.19 ± 2.58 | 64.72 ± 3.91 | 54.20 ± 8.00 |
| **D2** | 94.12 ± 7.24 | 103.85 ± 1.71 | 98.64 ± 11.82 | 98.82 ± 3.42 | 90.32 ± 5.70 |
| **D3** | 101.17 ± 11.19 | 98.99 ± 9.10 | 95.93 ± 7.95 | 94.33 ± 2.62 | 80.52 ± 3.65 |
| **D4** | 98.16 ± 3.32 | 105.06 ± 1.15 | 101.97 ± 5.69 | 103.80 ± 3.84 | 99.51 ± 6.32 |
| **D5** | 102.58 ± 1.46 | 94.60 ± 5.34 | 90.87 ± 6.20 | 87.18 ± 4.91 | 86.17 ± 8.00 |
| **CFT** | 109.88 ± 4.13 | 106.66 ± 4.19 | 99.62 ± 7.77 | 37.24 ± 2.65 | 15.63 ± 5.66 |
| **TMZ** | 101.97 ± 2.99 | 118.62 ± 0.53 | 113.53 ± 0.89 | 109.71 ± 3.37 | 104.50 ± 3.59 |
| Carmustine | 97.08 ± 2.44 | 108.83 ± 0.96 | 100.43 ± 2.09 | 108.50 ± 3.84 | 100.85 ± 1.02 |

**Table S2**. Cell viability of compounds against H9c2(2-1) cells

| **Compound** | **Cell viability (%)** | | | | |
| --- | --- | --- | --- | --- | --- |
|  | **6.25 µM** | **12.5 µM** | **25 µM** | **50 µM** | **100 µM** |
| **A1** | 91.40 ± 6.01 | 83.35 ± 6.95 | 81.71 ± 6.44 | 74.89 ± 2.93 | 64.71 ± 18.10 |
| **A2** | 99.72 ± 0.75 | 98.44 ± 0.76 | 91.58 ± 1.19 | 90.69 ± 1.85 | 87.89 ± 1.23 |
| **A3** | 85.11 ± 0.08 | 86.57 ± 0.78 | 82.36 ± 3.49 | 77.83 ± 0.66 | 77.77 ± 0.18 |
| **A4** | 95.82 ± 1.67 | 91.02 ± 3.78 | 89.47 ± 0.17 | 86.23 ± 1.23 | 78.89 ± 0.43 |
| **A5** | 85.78 ± 1.32 | 79.98 ± 1.46 | 84.71 ± 2.93 | 85.64 ± 2.24 | 81.91 ± 2.04 |
| **A6** | 101.03 ± 4.15 | 99.97 ± 1.40 | 95.16 ± 0.35 | 91.46 ± 0.35 | 89.11 ± 1.05 |
| **A7** | 109.68 ± 0.55 | 99.27 ± 2.60 | 100.44 ± 0.79 | 101.32 ± 2.54 | 95.00 ± 1.07 |
| **B1** | 104.94 ± 1.03 | 94.66 ± 2.71 | 93.45 ± 0.83 | 89.54 ± 3.87 | 89.67 ± 3.28 |
| **B2** | 105.12 ± 1.95 | 89.77 ± 3.47 | 95.30 ± 1.53 | 91.53 ± 1.88 | 90.78 ± 4.74 |
| **B3** | 99.38 ± 1.66 | 95.05 ± 2.51 | 96.67 ± 1.11 | 91.80 ± 0.22 | 92.02 ± 0.92 |
| **B4** | 105.00 ± 0.28 | 105.98 ± 1.33 | 101.23 ± 0.11 | 93.20 ± 0.88 | 89.72 ± 0.33 |
| **B5** | 95.71 ± 0.09 | 85.02 ± 1.81 | 88.23 ± 2.39 | 86.53 ± 1.21 | 85.19 ± 0.33 |
| **B6** | 99.35 ± 0.18 | 78.66 ± 3.24 | 87.31 ± 0.83 | 83.66 ± 0.77 | 86.44 ± 1.46 |
| **B7** | 100.35 ± 1.51 | 91.17 ± 0.94 | 91.80 ± 1.72 | 87.00 ± 1.36 | 68.98 ± 0.46 |
| **B8** | 100.96 ± 0.18 | 87.39 ± 3.01 | 82.22 ± 9.85 | 68.96 ± 1.77 | 42.18 ± 2.31 |
| **B9** | 92.07 ± 4.26 | 82.28 ± 2.18 | 82.20 ± 0.56 | 84.85 ± 2.16 | 82.93 ± 0.74 |
| **B10** | 98.45 ± 1.69 | 92.44 ± 1.25 | 82.95 ± 0.58 | 84.57 ± 0.46 | 75.34 ± 0.35 |
| **B11** | 100.39 ± 2.64 | 90.86 ± 0.46 | 86.49 ± 3.33 | 83.88 ± 0.42 | 77.87 ± 2.90 |
| **B12** | 96.93 ± 3.92 | 97.75 ± 1.15 | 97.40 ± 1.09 | 96.16 ± 2.60 | 90.93 ± 3.39 |
| **B13** | 98.92 ± 1.07 | 100.05 ± 0.50 | 96.32 ± 2.74 | 90.30 ± 1.76 | 90.14 ± 1.47 |
| **C1** | 104.87 ± 1.80 | 100.87 ± 0.59 | 97.33 ± 2.07 | 92.24 ± 1.17 | 76.43 ± 0.88 |
| **C2** | 100.35 ± 2.16 | 94.75 ± 0.96 | 90.86 ± 0.38 | 82.01 ± 1.03 | 72.40 ± 1.30 |
| **C3** | 96.79 ± 1.01 | 95.17 ± 0.04 | 91.12 ± 0.44 | 94.07 ± 1.13 | 89.62 ± 1.36 |
| **C4** | 98.71 ± 1.81 | 95.67 ± 0.94 | 91.27 ± 0.11 | 88.92 ± 1.99 | 68.80 ± 0.74 |
| **D1** | 97.25 ± 5.10 | 94.45 ± 0.35 | 92.76 ± 0.59 | 89.94 ± 1.23 | 88.45 ± 0.79 |
| **D2** | 94.07 ± 1.40 | 93.64 ± 2.01 | 88.16 ± 2.73 | 89.74 ± 2.87 | 85.27 ± 2.03 |
| **D3** | 95.87 ± 2.17 | 91.65 ± 0.57 | 85.90 ± 2.28 | 86.60 ± 0.06 | 81.55 ± 2.45 |
| **D4** | 100.05 ± 1.16 | 96.32 ± 1.03 | 92.83 ± 1.27 | 86.26 ± 4.52 | 87.64 ± 1.42 |
| **D5** | 100.36 ± 1.82 | 92.38 ± 0.20 | 93.55 ± 1.42 | 90.68 ± 2.54 | 87.44 ± 1.11 |
| **CFT** | 93.76 ± 0.80 | 90.83 ± 1.82 | 82.90 ± 5.84 | 62.44 ± 2.83 | 46.27 ± 2.33 |
| **TMZ** | 96.17 ± 3.15 | 95.62 ± 1.14 | 88.72 ± 2.24 | 89.31 ± 0.13 | 87.26 ± 1.94 |
| Carmustine | 96.47 ± 3.23 | 92.18 ± 2.76 | 88.79 ± 0.60 | 83.77 ± 2.16 | 78.02 ± 3.17 |

**Table S3**. Cell viability of compounds against HT22 cells

| **Compound** | **Cell viability (%)** | | | | |
| --- | --- | --- | --- | --- | --- |
|  | **6.25 µM** | **12.5 µM** | **25 µM** | **50 µM** | **100 µM** |
| **A1** | 91.18 ± 0.82 | 99.16 ± 5.33 | 109.12 ± 4.25 | 111.95 ± 5.40 | 86.43 ± 9.14 |
| **A2** | 88.49 ± 9.39 | 96.88 ± 3.00 | 100.56 ± 3.03 | 56.93 ± 14.29 | 52.17 ± 6.20 |
| **A3** | 97.25 ± 3.35 | 97.66 ± 1.70 | 95.39 ± 5.24 | 94.83 ± 4.94 | 45.53 ± 5.80 |
| **A4** | 102.11 ± 4.84 | 102.48 ± 3.74 | 97.48 ± 4.03 | 105.17 ± 7.76 | 97.69 ± 5.47 |
| **A5** | 96.21 ± 7.83 | 105.80 ± 6.54 | 106.97 ± 12.68 | 114.23 ± 6.54 | 96.26 ± 5.37 |
| **A6** | 101.67 ± 4.86 | 97.67 ± 1.54 | 91.52 ± 6.62 | 99.88 ± 5.92 | 98.65 ± 4.23 |
| **A7** | 94.89 ± 7.05 | 92.42 ± 3.08 | 107.09 ± 1.32 | 107.36 ± 7.69 | 96.61 ± 2.32 |
| **B1** | 97.38 ± 9.37 | 94.30 ± 5.34 | 91.43 ± 1.67 | 98.43 ± 4.33 | 107.95 ± 6.39 |
| **B2** | 108.18 ± 7.93 | 116.48 ± 2.32 | 109.77 ± 4.97 | 107.40 ± 4.24 | 101.00 ± 4.91 |
| **B3** | 102.22 ± 8.75 | 94.67 ± 7.92 | 93.74 ± 10.10 | 96.42 ± 6.41 | 100.48 ± 6.97 |
| **B4** | 107.50 ± 4.22 | 113.07 ± 3.98 | 95.14 ± 2.92 | 89.31 ± 4.92 | 88.34 ± 5.37 |
| **B5** | 91.39 ± 6.25 | 135.42 ± 11.13 | 94.51 ± 9.97 | 75.46 ± 7.74 | 73.56 ± 9.65 |
| **B6** | 92.87 ± 7.89 | 94.86 ± 2.52 | 86.48 ± 2.35 | 73.39 ± 9.29 | 42.63 ± 2.45 |
| **B7** | 106.46 ± 3.19 | 97.73 ± 8.47 | 76.78 ± 9.14 | 82.58 ± 4.16 | 79.94 ± 1.71 |
| **B8** | 80.14 ± 1.21 | 70.80 ± 5.26 | 46.37 ± 3.52 | 41.02 ± 0.77 | 43.64 ± 2.37 |
| **B9** | 96.42 ± 4.78 | 97.73 ± 4.50 | 99.65 ± 5.09 | 87.42 ± 2.41 | 83.33 ± 4.33 |
| **B10** | 95.45 ± 1.08 | 84.53 ± 5.86 | 78.41 ± 2.09 | 84.41 ± 1.86 | 39.09 ± 0.82 |
| **B11** | 96.39 ± 9.22 | 92.69 ± 12.75 | 87.59 ± 2.49 | 84.87 ± 11.78 | 88.08 ± 2.63 |
| **B12** | 82.94 ± 7.47 | 77.44 ± 7.55 | 79.64 ± 1.11 | 77.64 ± 3.97 | 82.03 ± 2.16 |
| **B13** | 96.45 ± 1.62 | 83.70 ± 4.51 | 82.88 ± 5.96 | 86.85 ± 9.79 | 72.20 ± 5.41 |
| **C1** | 101.29 ± 13.03 | 73.51 ± 6.32 | 71.00 ± 1.73 | 68.47 ± 2.96 | 65.96 ± 10.37 |
| **C2** | 98.93 ± 3.38 | 91.24 ± 11.90 | 91.36 ± 6.44 | 98.92 ± 1.20 | 98.59 ± 2.74 |
| **C3** | 98.50 ± 4.95 | 98.92 ± 7.31 | 100.54 ± 7.14 | 100.93 ± 4.36 | 92.81 ± 8.71 |
| **C4** | 102.50 ± 5.52 | 101.53 ± 6.45 | 84.91 ± 8.00 | 83.32 ± 8.88 | 71.09 ± 9.30 |
| **D1** | 96.41 ± 1.70 | 90.52 ± 10.56 | 80.73 ± 6.93 | 74.43 ± 6.49 | 71.40 ± 5.43 |
| **D2** | 109.27 ± 3.64 | 104.44 ± 7.43 | 96.07 ± 9.55 | 92.06 ± 3.60 | 76.48 ± 2.54 |
| **D3** | 91.51 ± 15.47 | 86.84 ± 8.03 | 95.35 ± 7.68 | 99.57 ± 9.63 | 96.13 ± 1.16 |
| **D4** | 96.70 ± 4.07 | 95.43 ± 8.15 | 87.78 ± 6.64 | 83.62 ± 4.50 | 79.15 ± 7.27 |
| **D5** | 99.18 ± 6.81 | 89.24 ± 1.55 | 92.19 ± 2.53 | 82.75 ± 4.18 | 71.28 ± 5.17 |
| **CFT** | 88.85 ± 2.67 | 86.43 ± 2.19 | 65.27 ± 4.08 | 62.33 ± 0.51 | 57.52 ± 2.24 |
| **TMZ** | 98.03 ± 4.19 | 102.15 ± 5.67 | 106.25 ± 8.38 | 99.87 ± 7.79 | 96.59 ± 1.16 |
| Carmustine | 98.00 ± 3.22 | 99.04 ± 3.42 | 100.16 ± 3.20 | 94.13 ± 3.78 | 91.06 ± 1.28 |

**Table S4**. Cell viability of compounds against Vero cells

| **Compound** | **Cell viability (%)** | | | | |
| --- | --- | --- | --- | --- | --- |
|  | **6.25 µM** | **12.5 µM** | **25 µM** | **50 µM** | **100 µM** |
| **A1** | 100.15 ± 6.06 | 99.18 ± 1.22 | 82.02 ± 0.59 | 89.40 ± 0.77 | 80.29 ± 2.39 |
| **A2** | 90.04 ± 2.10 | 92.09 ± 4.96 | 89.21 ± 2.57 | 89.86 ± 6.18 | 82.84 ± 2.00 |
| **A3** | 87.95 ± 5.85 | 78.20 ± 13.49 | 74.85 ± 2.98 | 67.72 ± 3.72 | 75.96 ± 2.75 |
| **A4** | 90.01 ± 7.81 | 83.54 ± 5.78 | 81.38 ± 2.25 | 78.27 ± 3.43 | 83.28 ± 0.47 |
| **A5** | 92.90 ± 8.27 | 66.85 ± 0.50 | 73.49 ± 3.62 | 69.00 ± 3.29 | 59.46 ± 1.79 |
| **A6** | 76.74 ± 3.96 | 68.59 ± 1.36 | 74.93 ± 2.50 | 70.69 ± 0.80 | 70.77 ± 3.39 |
| **A7** | 103.42 ± 3.01 | 95.27 ± 0.40 | 97.49 ± 1.26 | 93.45 ± 1.27 | 95.47 ± 2.80 |
| **B1** | 85.99 ± 5.76 | 88.23 ± 1.32 | 93.97 ± 6.13 | 86.33 ± 2.59 | 91.80 ± 1.20 |
| **B2** | 100.81 ± 0.11 | 87.82 ± 6.12 | 86.50 ± 0.86 | 92.36 ± 0.73 | 92.23 ± 1.60 |
| **B3** | 89.77 ± 7.14 | 89.34 ± 1.37 | 87.23 ± 1.38 | 89.66 ± 2.21 | 95.38 ± 4.48 |
| **B4** | 96.34 ± 0.94 | 88.91 ± 5.21 | 81.00 ± 1.13 | 89.27 ± 0.53 | 90.88 ± 3.94 |
| **B5** | 82.32 ± 0.65 | 89.18 ± 2.77 | 92.32 ± 1.18 | 92.16 ± 0.19 | 97.53 ± 0.80 |
| **B6** | 97.69 ± 6.01 | 86.99 ± 0.33 | 89.52 ± 2.14 | 91.84 ± 3.35 | 94.15 ± 0.76 |
| **B7** | 85.35 ± 4.08 | 93.25 ± 2.02 | 89.49 ± 2.45 | 87.29 ± 0.85 | 83.29 ± 1.33 |
| **B8** | 96.50 ± 1.90 | 81.90 ± 2.43 | 83.41 ± 0.54 | 88.28 ± 9.70 | 58.61 ± 4.97 |
| **B9** | 80.78 ± 3.25 | 78.29 ± 0.01 | 74.42 ± 1.16 | 74.81 ± 1.70 | 82.26 ± 2.02 |
| **B10** | 82.53 ± 1.09 | 81.03 ± 2.30 | 77.87 ± 0.51 | 86.28 ± 5.50 | 82.89 ± 1.91 |
| **B11** | 79.77 ± 1.04 | 79.45 ± 2.40 | 79.04 ± 0.01 | 80.56 ± 1.89 | 83.13 ± 0.69 |
| **B12** | 98.43 ± 0.01 | 91.65 ± 0.67 | 89.58 ± 1.95 | 92.59 ± 0.62 | 93.35 ± 4.45 |
| **B13** | 89.16 ± 0.51 | 95.38 ± 4.55 | 93.48 ± 1.66 | 93.83 ± 0.46 | 94.90 ± 4.48 |
| **C1** | 100.58 ± 1.52 | 109.71 ± 7.32 | 93.37 ± 1.00 | 95.92 ± 1.15 | 95.92 ± 3.31 |
| **C2** | 77.57 ± 1.38 | 87.52 ± 5.74 | 87.18 ± 1.96 | 98.95 ± 1.68 | 98.68 ± 7.96 |
| **C3** | 91.10 ± 2.35 | 91.06 ± 0.52 | 89.93 ± 2.54 | 86.28 ± 2.53 | 79.41 ± 0.70 |
| **C4** | 86.93 ± 2.39 | 84.28 ± 3.15 | 88.25 ± 2.59 | 91.34 ± 1.19 | 91.89 ± 2.49 |
| **D1** | 94.60 ± 2.86 | 85.31 ± 3.34 | 96.76 ± 3.69 | 90.52 ± 1.09 | 87.00 ± 0.29 |
| **D2** | 99.09 ± 5.48 | 98.98 ± 4.65 | 84.12 ± 1.78 | 89.36 ± 1.75 | 88.66 ± 7.23 |
| **D3** | 90.10 ± 0.66 | 90.45 ± 4.75 | 84.57 ± 2.00 | 82.86 ± 5.48 | 89.81 ± 3.30 |
| **D4** | 88.34 ± 2.31 | 80.12 ± 4.73 | 73.80 ± 0.99 | 80.50 ± 7.77 | 76.49 ± 3.47 |
| **D5** | 87.57 ± 5.81 | 81.48 ± 1.85 | 78.99 ± 0.31 | 80.41 ± 0.50 | 79.29 ± 3.08 |
| **CFT** | 100.32 ± 1.76 | 97.95 ± 3.48 | 96.22 ± 1.07 | 83.42 ± 2.50 | 49.21 ± 3.50 |
| **TMZ** | 98.51 ± 2.18 | 96.79 ± 1.59 | 99.44 ± 2.37 | 93.51 ± 4.26 | 73.21 ± 7.37 |
| Carmustine | 99.62 ± 0.95 | 98.21 ± 3.39 | 96.08 ± 5.57 | 89.00 ± 7.00 | 63.77 ± 11.71 |

**Table S5**. Cell viability of compounds against HEK-293 cells

| **Compound** | **Cell viability (%)** | | | | |
| --- | --- | --- | --- | --- | --- |
|  | **6.25 µM** | **12.5 µM** | **25 µM** | **50 µM** | **100 µM** |
| **A1** | 101.39 ± 1.42 | 98.08 ± 7.82 | 92.35 ± 6.79 | 82.61 ± 7.29 | 76.78 ± 7.56 |
| **A2** | 96.09 ± 0.30 | 92.36 ± 0.45 | 90.18 ± 3.78 | 92.61 ± 6.02 | 70.14 ± 1.27 |
| **A3** | 97.62 ± 4.39 | 97.74 ± 1.45 | 94.91 ± 3.13 | 88.10 ± 1.14 | 80.74 ± 3.36 |
| **A4** | 95.40 ± 3.07 | 93.30 ± 2.20 | 92.90 ± 0.79 | 71.85 ± 0.90 | 71.89 ± 1.19 |
| **A5** | 98.87 ± 2.59 | 96.59 ± 2.82 | 94.44 ± 2.54 | 83.85 ± 1.41 | 71.17 ± 1.90 |
| **A6** | 96.96 ± 0.17 | 94.01 ± 1.38 | 94.05 ± 1.10 | 84.53 ± 2.13 | 75.68 ± 3.94 |
| **A7** | 99.23 ± 1.81 | 101.85 ± 4.98 | 108.52 ± 0.45 | 95.51 ± 1.96 | 87.97 ± 2.68 |
| **B1** | 105.38 ± 5.26 | 98.91 ± 4.51 | 97.05 ± 4.33 | 86.51 ± 1.06 | 78.59 ± 3.09 |
| **B2** | 96.80 ± 1.51 | 99.27 ± 1.35 | 102.04 ± 3.63 | 88.95 ± 6.31 | 74.35 ± 3.45 |
| **B3** | 111.73 ± 4.18 | 102.63 ± 0.40 | 96.39 ± 2.30 | 92.18 ± 3.60 | 88.87 ± 0.64 |
| **B4** | 106.40 ± 1.14 | 104.88 ± 2.80 | 106.99 ± 1.61 | 81.80 ± 4.30 | 74.64 ± 6.08 |
| **B5** | 101.45 ± 1.16 | 98.62 ± 2.22 | 95.73 ± 6.57 | 77.77 ± 2.93 | 73.29 ± 3.45 |
| **B6** | 100.84 ± 0.65 | 98.14 ± 2.87 | 97.64 ± 0.11 | 101.80 ± 2.36 | 90.91 ± 1.69 |
| **B7** | 99.40 ± 6.41 | 94.51 ± 1.82 | 93.67 ± 1.47 | 86.83 ± 1.80 | 81.16 ± 0.83 |
| **B8** | 92.56 ± 0.99 | 90.78 ± 0.59 | 86.80 ± 2.37 | 87.62 ± 0.02 | 70.15 ± 1.43 |
| **B9** | 99.62 ± 2.85 | 93.00 ± 1.57 | 97.11 ± 0.36 | 89.93 ± 0.46 | 86.73 ± 0.72 |
| **B10** | 105.26 ± 2.11 | 104.88 ± 3.99 | 96.65 ± 7.50 | 102.08 ± 1.43 | 98.85 ± 0.77 |
| **B11** | 102.41 ± 2.04 | 95.60 ± 1.62 | 99.79 ± 0.67 | 97.30 ± 2.17 | 97.32 ± 1.93 |
| **B12** | 97.13 ± 3.22 | 96.75 ± 3.51 | 94.22 ± 3.15 | 89.68 ± 5.60 | 73.15 ± 2.37 |
| **B13** | 104.86 ± 1.64 | 100.22 ± 0.05 | 100.85 ± 3.04 | 87.02 ± 4.67 | 81.02 ± 2.79 |
| **C1** | 93.78 ± 1.84 | 93.84 ± 1.27 | 92.20 ± 0.94 | 90.27 ± 0.25 | 82.27 ± 1.56 |
| **C2** | 96.73 ± 4.55 | 94.79 ± 2.07 | 88.96 ± 0.54 | 78.65 ± 5.48 | 70.90 ± 1.93 |
| **C3** | 103.13 ± 2.00 | 97.33 ± 0.51 | 94.37 ± 0.36 | 92.82 ± 0.01 | 87.52 ± 0.64 |
| **C4** | 106.54 ± 2.04 | 95.60 ± 2.90 | 85.62 ± 1.83 | 82.80 ± 1.06 | 70.96 ± 0.70 |
| **D1** | 100.80 ± 0.75 | 99.21 ± 3.18 | 97.83 ± 0.31 | 82.05 ± 1.36 | 77.84 ± 3.43 |
| **D2** | 96.71 ± 5.08 | 95.81 ± 1.29 | 93.56 ± 3.70 | 81.05 ± 3.83 | 67.77 ± 0.96 |
| **D3** | 95.48 ± 1.96 | 95.81 ± 6.67 | 92.66 ± 2.23 | 85.64 ± 4.54 | 74.68 ± 4.53 |
| **D4** | 103.80 ± 5.52 | 97.99 ± 1.85 | 94.57 ± 4.59 | 87.05 ± 4.43 | 84.80 ± 2.93 |
| **D5** | 104.37 ± 1.94 | 100.93 ± 3.04 | 98.21 ± 0.48 | 93.21 ± 3.85 | 95.33 ± 0.45 |
| **CFT** | 108.72 ± 1.29 | 98.93 ± 0.77 | 91.50 ± 1.24 | 81.02 ± 1.22 | 65.57 ± 3.70 |
| **TMZ** | 102.16 ± 1.90 | 97.80 ± 1.12 | 94.11 ± 0.88 | 92.78 ± 1.70 | 87.03 ± 3.15 |
| Carmustine | 105.35 ± 2.43 | 99.45 ± 2.56 | 97.42 ± 1.43 | 93.65 ± 1.17 | 88.33 ± 1.65 |

**Table S6**. ADMET prediction results of compounds

| Compound | Solubility  Level^a)^ | BBB  Leve^b)^ | Absorption  Level^c)^ | PPB  Prediction^d)^ | CYP2D6  Prediction^e)^ |
| --- | --- | --- | --- | --- | --- |
| **B7** | 1 | 0 | 2 | TRUE | TRUE |
| **B8** | 1 | 4 | 3 | TRUE | TRUE |
| **CFT** | 1 | 4 | 3 | TRUE | TRUE |

^a)^The solubility level was graded on a scale of 0–6, where 0 represented extremely low solubility, 1 denoted very low solubility, 2 indicated low solubility, 3 signified good solubility, 4 represented optimal solubility, 5 represented too soluble solubility, and 6 served as a warning for molecules with one or more unknown AlogP98 types; ^b)^The blood-brain barrier (BBB) level was graded on a scale of 0–5, where 0 indicated very high penetrant, 1 presented high penetrant, 2 stood for medium penetrant, 3 donated low penetrant, 4 meant undefined (outside 99% confidence ellipse), and 5 indicated a warning for molecules with one or more unknown AlogP98 types; ^c)^The absorption level was rated on a scale of 0–3, where 0 indicated good absorption, 1 represented moderate absorption, 2 denoted low absorption, and 3 meant very low absorption; ^d)^The classification of whether a compound was highly bound (>= 90% bound) to plasma proteins, represented by TRUE, indicating high binding (>= 90% bound); ^e)^The classification of whether a compound was a CYP2D6 inhibitor, represented by TRUE, denoting CYP2D6 inhibitor.

**Table S7**. Safety test of compound **B7** *in vivo*

| **Dose** | **Number** | **Results** |
| --- | --- | --- |
| 0 mg/kg | 3 （2♂，1♀） | Behavior and activities are normal |
| 40 mg/kg |  | Behavior and activities are normal |
| 80 mg/kg |  | Behavior and activities are normal |
| 100 mg/kg |  | Behavior and activities are normal |
| 120 mg/kg |  | One mouse died |
| 200 mg/kg |  | Three mice died |

**Table S8.** Binding energies of **B7**-CD155 complexes

| Item | Energy(kJ/mol) | Delta |
| --- | --- | --- |
| ΔG_vdw_(kJ/mol) | -104.573 | 8.824 |
| ΔG_ele_(kJ/mol) | -0.550 | 0.495 |
| ΔG_PB_(kJ/mol) | 64.304 | 3.896 |
| ΔG_np_(kJ/mol) | -10.351 | 2.472 |
| **ΔG_bind_(kJ/mol)** | **-51.170** | **6.191** |

ΔG_vdw_: the intermolecular van der Waals; ΔG_ele_: electrostatic interactions; ΔG_PB_: polar solvation free energy; ΔG_np_: nonpolar solvation energy; ΔG_bind_: the total binding free energy.

**Table S9.** Sequences for qRT-PCR analysis

| Name | Sequences |
| --- | --- |
| PD-L1 | FORWARD: AAGGTGGAGCGTCATCGTCATC  REVERSE: CCCTCGGCTACAGTCAATCTCATC |
| PD-L2 | FORWARD: AGAGGGAAGTGAACAGTGCT  REVERSE: TAGGCTCCAGAGGTGAGTCC |
| CD112 | FORWARD: TGGAGGCGGCAAAGCACAAC  REVERSE: GGGAAGAGCAGCAGCAGCAG |
| CD155 | FORWARD: GCGGAGCTGCGGAATGCC  REVERSE: GCGGGAACGTGACGAACAGG |
| CD96 | FORWARD: ATGGTCGGTGGAGGATAATGGAAC  REVERSE: TGGAGAGAGGTGGAGTCTGTAGTC |
| HLA-A | FORWARD: GCTGTTCTAAAGTCCGCACG  REVERSE: CTTGGTGATCTGAGCTGCCA |
| HLA-B | FORWARD: CACACAGATCTACAAGGCCCA  REVERSE: CACACAGATCTACAAGGCCCA |
| Galectin-9 | FORWARD: TGGAGACAATGGCGACTTTACCC  REVERSE: CAGATGGATATGGCAGGTGTAGGTC |
| Galectin-3 | FORWARD: GTCCGGAGCCAGCCAAC  REVERSE: AGGCCATCCTTGAGGGTTTG |
| CD80 | FORWARD: ATGACAGGCACAATAGAAACAACGG  REVERSE: GTGGTGGAGGAGAGGTGACATTG |
| KLF13 | FORWARD: TCACAGACCCACGGTTTTCC  REVERSE: TGGAGACACATCCTTTGCCC |
| CD113 | FORWARD: GGAGCAGGTTGGATGGACAA  REVERSE: TTGTAGGCTGAAATGTAGATGACT |
| B7-H3 | FORWARD: CTCACAGGAAGATGCTGCGT  REVERSE: CTCTGGGGTGTGATGGTGAC |
| B7-H4 | FORWARD: GTGCTCCCTGATTCCTCCAG  REVERSE: GGTTAGCATTCCCCTTGCCT |
| VISR | FORWARD: TAGTCCAGCCTGGGCATTTG  REVERSE: AGCATTGTGGTGACCAGAGG |
| HHLA2 | FORWARD: AGAACTGGATGCCCTTGTGG  REVERSE: GCCAGGACAGAGAAAGTCCC |
| CD40 | FORWARD: CAGTCGGCTTCTTCTCCAATGTG REVERSE: TCTTGTTTGTGCCTGCCTGTTG |
| VSIG4 | FORWARD: AAGCAACATCTACAGTGAAGCAGTC REVERSE: ATGATGAGGATGATGGCAAAGACAG |
| PSGL-1 | FORWARD: TCCTCCTGTTGCTGATCCTACTG REVERSE: CCAAGGCTTTCTCGGCTTCATC |
| OX40L | FORWARD: AGAAGGTCAGGTCTGTCAACTCC REVERSE: AGGTATTGTCAGTGGTCACATTCAAG |
| ICOS | FORWARD: ACTCTGCGATCTCACTAAGACAAAAG REVERSE: AGAGACACTGTTGTTGGATAACTGAG |
| NCR3LG1 | FORWARD: GCCTTCTTTGCCTTGCGAGAC REVERSE: ATGGACTTGCCTGATGTAACTGTTAG |
| CD137L | FORWARD: GAATACGCCTCTGACGCTTCAC REVERSE: CAGCAGCAGCAGCAGCAG |
| CD47 | FORWARD: TAACCTCCTTCGTCATTGCCATATTG REVERSE: AATACAGAGACTCAGTCCAACCACAG |
| GAPDH | FORWARD: CACCCACTCCTCCACCTTTGAC  REVERSE: GTCCACCACCCTGTTGCTGTAG |





**Figure S1.** Qualitative detection of compound **B7** in the blood, brain, heart, liver, spleen, lung, and kidney tissues following *in vivo* administration. Liquid chromatography analysis demonstrates the presence of **B7** in the blood (A), brain (B), heart (C), liver (D), spleen (E), lung (F), and kidney (G) tissues at 5 min after a single intravenous tail injection of 20 mg/kg of **B7** in mice.





**Figure S2.** Assessment of the blood-brain barrier penetration of compound **B8**. Liquid chromatography analysis demonstrates the presence of **B8** in the blood (A) and brain (B) at 5 min after a single intravenous tail injection of 20 mg/kg of **B8** in mice.





**Figure S3.** Levels of aspartate aminotransferase (AST), alanine aminotransferase (ALT), lactate dehydrogenase (LDH), blood urea nitrogen (BUN), creatinine (CRE), and creatine kinase (CK) in the serum of mice. Multiple group comparisons were conducted using one-way ANOVA, followed by Tukey’s *post hoc* test for inter-group difference analysis. The results are expressed as mean ±SD, *n*=5.





**Figure S4.** CD155 serves as the target for compound **B7**. The mRNA expression levels of KLF13 (A), CD155 (B), CD112 (C), CD113 (D), PD-L1 (E), PD-L2 (F), CD80 (G), CD86 (H), HLA-A (I), HLA-B (G), Galectin-3 (K), Galectin-9 (L), B7-H3 (M), B7-H4 (N), VISTA (O), HHLA2 (P), OX40L (Q), VSIG4 (R), CD40 (S), PSGL-1 (T), CD137L (U), ICOS (V), NCR3LG1 (W), and CD47 (X) were detected using qRT-PCR. One-way ANOVA and Tukey’s *post hoc* test were employed to compare differences between multiple groups. The results are expressed as mean ±SD, *n*=3. ^*^*P* < 0.05, ^***^*P* < 0.001 *vs.* control.





**Figure S5.** Network pharmacological prediction for the treatment of glioma with **B7**. A–C) GO enrichment analysis of the targets’ biological processes (A), molecular functions (B), and cellular components (C). D) KEGG pathway enrichment analysis.





**Figure S6.** Effects of **B7** treatment and CD155 modulation on T-cell, B-cell, macrophage, and monocyte populations in tumor tissues. Panel A presents representative flow cytometry images, while Panel B provides quantitative analyses of CD45R⁺, F4/80⁺, and CD11b⁺ cell populations in different tumor tissue groups. Multiple group comparisons were performed using one-way ANOVA, followed by Tukey’s *post hoc* test for inter-group difference analysis. The results were presented as mean ± SD. *n*=3.











**Figure S7.** Original pictures of Western blotting analyses.

**Supplementary Methods**

**1. General procedure for preparation of phenyl compounds 3a-d**

Appropriately substituted benzoic acid compounds **2a**-**d** (1 equiv) were dissolved in anhydrous tetrahydrofuran (25 mL) and stirred at 0 °C for 30 min under a N_2_ atmosphere. LiAlH_4_ (2 equiv) was added drop by drop in anhydrous THF (10 mL) and then stirred for 2 h at room temperature. The mixture was quenched by the addition of ice-water, 5% NaOH was added to form a white precipitate, which was filtered through diatomite. It was extracted with ethyl acetate (25 mL × 3). The merged organic layer was dried over anhydrous Na_2_SO_4_, concentrated under reduced pressure, and purified over silica gel to give the target compounds.

(2-chloro-4-methylphenyl)methanol (**3a**). Slight yellow soild; mp 42.5-45.1 °C; yield: 86%. ^1^H NMR (600 MHz, Chloroform-*d*) δ (ppm): 7.32 (d, *J* = 7.7 Hz, 1H), 7.18 (d, *J* = 1.7 Hz, 1H), 7.08 – 7.05 (m, 1H), 4.71 (s, 2H), 2.32 (s, 3H). ESI-MS calcd for C_8_H_10_ClO [M + H] ^+^ 157, found 157. (Figure S8)

(4-chloro-2-methylphenyl)methanol (**3b**). White solid; mp 55.5-58.2 °C; yield: 87%. ^1^H NMR (600 MHz, Chloroform-*d*) δ (ppm): 7.28 (dd, *J* = 8.8, 3.1 Hz, 1H), 7.18 – 7.15 (m, 2H), 4.65 (s, 2H), 2.32 (s, 3H). ESI-MS calcd for C_8_H_10_ClO [M + H] ^+^ 157, found 157. (Figure S9)

(4-isopropylphenyl)methanol (**3c**). Colorless [liquid](javascript:;); yield: 83%. ^1^H NMR (600 MHz, Chloroform-*d*) δ (ppm): 7.30 (d, *J* = 8.4 Hz, 2H), 7.24 (d, *J* = 7.2 Hz, 2H), 4.65 (s, 2H), 2.92 (h, *J* = 6.9 Hz, 1H), 1.27 (d, *J* = 7.0 Hz, 6H). ESI-MS calcd for C_10_H_15_O [M + H] ^+^ 151, found 151. (Figure S10)

(2-isopropylphenyl)methanol (**3d**). Colorless [oil](javascript:;); yield: 85%. ^1^H NMR (500 MHz, DMSO-*d*_6_) δ (ppm): 7.33 (d, *J* = 7.6 Hz, 1H), 7.27 (d, *J* = 7.6 Hz, 1H), 7.22 (t, *J* = 7.4 Hz, 1H), 7.13 (t, *J* = 7.3 Hz, 1H), 5.01 (t, *J* = 5.4 Hz, 1H), 4.55 (d, *J* = 5.4 Hz, 2H), 3.17 (h, *J* = 6.9 Hz, 1H), 1.18 (d, *J* = 6.9 Hz, 6H). ESI-MS calcd for C_10_H_15_O [M + H] ^+^ 151, found 151. (Figure S11)

**2. General procedure for preparation of 4a-d**

The compounds **3a**-**d** (1 equiv) were dissolved in anhydrous methylene chloride (25 ml) under a N_2_ atmosphere. SOCl_2_ (2 equiv) was added drop by drop under 0 °C, and then the mixture was raised to room temperature for 1.5 h. The reaction was quenched by the addition of ice-water and extracted with ethyl acetate (25mL × 3). The merged organic layer was dried over anhydrous Na_2_SO_4_, concentrated under reduced pressure, and purified over silica gel to give the target compounds.

2-chloro-1-(chloromethyl)-4-methylbenzene (**4a**). Colorless [liquid](javascript:;); yield: 82%. ^1^H NMR (400 MHz, DMSO-*d*_6_) δ (ppm): 7.48 (d, *J* = 7.7 Hz, 1H), 7.34 (s, 1H), 7.20 – 7.15 (m, 1H), 4.78 (s, 2H), 2.31 (s, 3H). ESI-MS calcd for C_8_H_9_Cl_2_ [M + H] ^+^ 175, found 175. (Figure S12)

4-chloro-1-(chloromethyl)-2-methylbenzene (**4b**). Colorless [liquid](javascript:;); yield: 85%. ^1^H NMR (400 MHz, DMSO-*d*_6_) δ (ppm): 7.42 (d, *J* = 8.2 Hz, 1H), 7.33 (d, *J* = 2.3 Hz, 1H), 7.26 (dd, *J* = 8.2, 2.3 Hz, 1H), 4.78 (s, 2H), 2.38 (s, 3H). ESI-MS calcd for C_8_H_9_Cl_2_ [M + H] ^+^ 175, found 175. (Figure S13)

1-(chloromethyl)-4-isopropylbenzene (**4c**). Slight yellow [liquid](javascript:;); yield: 86%. ^1^H NMR (600 MHz, Chloroform-*d*) δ (ppm): 7.32 (d, *J* = 8.1 Hz, 2H), 7.23 (d, *J* = 8.1 Hz, 2H), 4.58 (s, 2H), 2.92 (hept, *J* = 6.9 Hz, 1H), 1.26 (d, *J* = 6.9 Hz, 6H). ESI-MS calcd for C_10_H_14_Cl [M + H] ^+^ 169, found 169. (Figure S14)

1-(chloromethyl)-2-isopropylbenzene (**4d**). Colorless [oil](javascript:;); yield: 85%. ^1^H NMR (500 MHz, DMSO-*d*_6_) δ (ppm): 7.38 – 7.31 (m, 3H), 7.17 (td, *J* = 7.2, 1.9 Hz, 1H), 4.81 (s, 2H), 3.27 (h, *J* = 6.8 Hz, 1H), 1.22 (d, *J* = 6.8 Hz, 6H). ESI-MS calcd for C_10_H_14_Cl [M + H] ^+^ 169, found 169. (Figure S15)

**3. General procedure for preparation of A1-A7, B1-B13 and CFT**

Appropriately substituted phenol compounds **1a-h** (1 equiv) were dissolved in anhydrous chloroform (25 mL), and appropriately substituted benzyl chloride **4a-n** (1 equiv) and zinc chloride (0.2 equiv) were added. The mixture was stirred, heated and reflux for 16 h under a N_2_ atmosphere. The reaction was quenched by adding water and extracted with dichloromethane (25 mL × 3). The obtained organic layer was dried over anhydrous Na_2_SO_4_, concentrated under reduced pressure, and purified over silica gel to give the target compounds.

2-(2,4-dichlorobenzyl)-4-(2,4,4-trimethylpentan-2-yl)phenol (**CFT**). White solid; mp 77.8-79.9 °C; yield: 31%. ^1^H NMR (400 MHz, Chloroform-*d*) δ (ppm): 7.40 (d, *J* = 2.2 Hz, 1H), 7.15 – 7.10 (m, 2H), 7.07 (d, *J* = 2.4 Hz, 1H), 6.98 (d, *J* = 8.3 Hz, 1H), 6.70 (d, *J* = 8.3 Hz, 1H), 4.51 (s, 1H), 4.03 (s, 2H), 1.65 (s, 2H), 1.30 (s, 6H), 0.69 (s, 9H). ^13^C NMR (101 MHz, CDCl_3_) δ (ppm): 151.31, 142.86, 136.87, 134.86, 132.55, 131.25, 129.33, 129.18, 127.12, 125.86, 123.97, 114.99, 57.11, 38.07, 33.52, 32.44, 31.89, 31.81. ESI-HRMS calcd for C_21_H_30_ONCl_2_ [M + NH_4_]^+^ 382.16990, found 382.16925. HPLC: t_R_ = 5.85 min, normalization method purity 99.03 %. (Figure S16)

2-(2,4-dichlorobenzyl)-4-methylphenol (**A1**). White solid; mp 79.5-81.1 °C; yield: 31%. ^1^H NMR (600 MHz, Chloroform-*d*) δ (ppm): 7.40 (d, *J* = 2.1 Hz, 1H), 7.14 (dd, *J* = 8.3, 2.2 Hz, 1H), 7.03 (d, *J* = 8.3 Hz, 1H), 6.94 (dd, *J* = 8.1, 2.2 Hz, 1H), 6.85 (d, *J* = 2.2 Hz, 1H), 6.69 (d, *J* = 8.1 Hz, 1H), 4.57 (s, 1H), 4.01 (s, 2H), 2.24 (s, 3H). ^13^C NMR (151 MHz, CDCl_3_) δ (ppm): 151.43, 136.70, 134.87, 132.64, 131.61, 131.45, 130.49, 129.26, 128.61, 127.22, 124.92, 115.52, 32.96, 20.66.ESI-HRMS calcd for C_14_H_11_OCl_2_ [M - H] ^-^ 265.01815, found 265.01830. HPLC: t_R_ = 9.07 min, normalization method purity 98.98%. (Figure S17)

2-(2,4-dichlorobenzyl)-4-ethylphenol (**A2**). Slight yellow oil; yield: 35%. ^1^H NMR (600 MHz, Chloroform-*d*) δ (ppm): 7.41 (d, *J* = 2.2 Hz, 1H), 7.14 (dd, *J* = 8.3, 2.2 Hz, 1H), 7.04 (d, *J* = 8.3 Hz, 1H), 6.98 (dd, *J* = 8.1, 2.3 Hz, 1H), 6.90 (d, *J* = 2.2 Hz, 1H), 6.72 (d, *J* = 8.1 Hz, 1H), 4.72 (s, 1H), 4.03 (s, 2H), 2.56 (q, *J* = 7.6 Hz, 2H), 1.19 (t, *J* = 7.6 Hz, 3H). ^13^C NMR (151 MHz, CDCl_3_) δ (ppm): 151.60, 137.05, 136.71, 134.86, 132.59, 131.42, 130.55, 129.22, 127.37, 127.19, 124.90, 115.55, 33.09, 28.12, 15.98. ESI-HRMS calcd for C_15_H_13_OCl_2_ [M - H] ^-^ 279.03380, found 279.03360. HPLC: t_R_ = 9.50 min, normalization method purity 95.76%. (Figure S18)

2-(2,4-dichlorobenzyl)-4-propylphenol (**A3**). Slight yellow oil; yield: 27%. ^1^H NMR (400 MHz, Chloroform-*d*) δ (ppm): 7.45 (d, *J* = 2.2 Hz, 1H), 7.18 (dd, *J* = 8.3, 2.2 Hz, 1H), 7.08 (d, *J* = 8.3 Hz, 1H), 7.01 (dd, *J* = 8.1, 2.2 Hz, 1H), 6.92 (d, *J* = 2.2 Hz, 1H), 6.75 (d, *J* = 8.1 Hz, 1H), 4.74 (s, 1H), 4.07 (s, 2H), 2.54 (dd, *J* = 8.5, 6.7 Hz, 2H), 1.63 (h, *J* = 7.4 Hz, 2H), 0.97 (t, *J* = 7.3 Hz, 3H). ^13^C NMR (101 MHz, CDCl_3_) δ (ppm): 151.59, 136.70, 135.48, 134.85, 132.57, 131.39, 131.13, 129.21, 127.99, 127.18, 124.75, 115.45, 37.28, 33.10, 24.92, 13.89. ESI-HRMS calcd for C_16_H_15_OCl_2_ [M - H] ^-^ 293.04945, found 293.04929. HPLC: t_R_ = 13.24 min, normalization method purity 96.82%. (Figure S19)

4-butyl-2-(2,4-dichlorobenzyl)phenol (**A4**). Slight yellow solid; mp 49.4-51.0 °C; yield: 35%.^1^H NMR (600 MHz, Chloroform-*d*) δ (ppm): 7.40 (d, *J* = 2.2 Hz, 1H), 7.13 (dd, *J* = 8.3, 2.2 Hz, 1H), 7.03 (d, *J* = 8.3 Hz, 1H), 6.95 (dd, *J* = 8.1, 2.2 Hz, 1H), 6.87 (d, *J* = 2.2 Hz, 1H), 6.70 (d, *J* = 8.1 Hz, 1H), 4.59 (s, 1H), 4.02 (s, 2H), 2.52 – 2.46 (m, 2H), 1.53 (tt, *J* = 9.0, 6.9 Hz, 2H), 1.33 (q, *J* = 7.4 Hz, 2H), 0.91 (t, *J* = 7.4 Hz, 3H). ^13^C NMR (151 MHz, CDCl_3_) δ (ppm): 151.61, 136.74, 135.71, 134.88, 132.60, 131.41, 131.11, 129.24, 127.96, 127.20, 124.78, 115.49, 34.89, 34.01, 33.13, 22.43, 14.09.ESI-HRMS calcd for C_17_H_17_OCl_2_ [M - H] ^-^ 307.06510, found 307.06409. HPLC: t_R_ = 10.89 min, normalization method purity 99.19%. (Figure S20)

2-(2,4-dichlorobenzyl)-4-isopropylphenol (**A5**). Slight yellow oil; yield: 39%.^1^H NMR (600 MHz, Chloroform-*d*) δ (ppm): 7.40 (d, *J* = 2.2 Hz, 1H), 7.13 (dd, *J* = 8.3, 2.2 Hz, 1H), 7.03 (dd, *J* = 8.3, 0.8 Hz, 1H), 7.02 – 7.00 (m, 1H), 6.94 (d, *J* = 2.3 Hz, 1H), 6.72 (d, *J* = 8.2 Hz, 1H), 4.63 (s, 1H), 4.03 (s, 2H), 2.82 (hept, *J* = 6.9 Hz, 1H), 1.20 (d, *J* = 6.9 Hz, 6H).^13^C NMR (151 MHz, CDCl_3_) δ (ppm):151.68, 141.75, 136.73, 134.87, 132.59, 131.40, 129.32, 129.22, 127.19, 125.86, 124.76, 115.50, 33.40, 33.27, 24.34. ESI-HRMS calcd for C_16_H_15_OCl_2_ [M - H] ^-^ 293.04945, found 293.04933. HPLC: t_R_ = 9.88 min, normalization method purity 95.60%. (Figure S21)

4-(tert-butyl)-2-(2,4-dichlorobenzyl)phenol (**A6**). White solid; mp 56.1-57.7 °C; yield: 36%.^1^H NMR (600 MHz, Chloroform-*d*) δ (ppm): 7.40 (t, *J* = 2.1 Hz, 1H), 7.16 (dt, *J* = 8.3, 2.3 Hz, 1H), 7.13 (dq, *J* = 6.2, 2.1 Hz, 2H), 7.03 (dd, *J* = 8.4, 2.1 Hz, 1H), 6.72 (dd, *J* = 8.3, 2.0 Hz, 1H), 4.60 (s, 1H), 4.04 (s, 2H), 1.27 (s, 9H). ^13^C NMR (151 MHz, CDCl_3_) δ (ppm): 151.42, 144.07, 136.78, 134.84, 132.57, 131.36, 129.21, 128.39, 127.18, 124.97, 124.32, 115.18, 34.23, 33.51, 31.66. ESI-HRMS calcd for C_17_H_17_OCl_2_ [M - H] ^-^ 307.06510, found 307.06477. HPLC: t_R_ = 10.37 min, normalization method purity 98.20%. (Figure S22)

2-(2,4-dichlorobenzyl)-4-(tert-pentyl)phenol (**A7**). White solid; mp 57.9-59.4 °C; yield: 38%. ^1^H NMR (600 MHz, Chloroform-*d*) δ (ppm): 7.40 (d, *J* = 2.2 Hz, 1H), 7.11 (ddd, *J* = 16.9, 8.3, 2.3 Hz, 2H), 7.05 (d, *J* = 2.4 Hz, 1H), 7.00 (d, *J* = 8.3 Hz, 1H), 6.72 (d, *J* = 8.3 Hz, 1H), 4.56 (s, 1H), 4.03 (s, 2H), 1.57 (q, *J* = 7.4 Hz, 2H), 1.23 (s, 6H), 0.66 (t, *J* = 7.4 Hz, 3H). ^13^C NMR (151 MHz, CDCl_3_) δ (ppm): 151.34, 142.31, 136.83, 134.86, 132.55, 131.28, 129.20, 129.09, 127.17, 125.69, 124.17, 115.15, 37.43, 37.09, 33.57, 28.71, 9.26. ESI-HRMS calcd for C_18_H_19_OCl_2_ [M - H] ^-^ 321.08075, found 321.08049. HPLC: t_R_ = 12.47 min, normalization method purity 98.79%. (Figure S23)

2-(2-chloro-4-methylbenzyl)-4-(2,4,4-trimethylpentan-2-yl)phenol (**B1**). White solid; mp 64.4-67.2 °C; yield: 31%. ^1^H NMR (500 MHz, DMSO-*d*_6_) δ (ppm): 9.10 (s, 1H), 7.25 (s, 1H), 7.02 (d, *J* = 10.1 Hz, 2H), 6.97 (d, *J* = 8.0 Hz, 1H), 6.92 (d, *J* = 2.5 Hz, 1H), 6.71 (d, *J* = 8.4 Hz, 1H), 3.88 (s, 2H), 2.26 (s, 3H), 1.57 (s, 2H), 1.21 (s, 6H), 0.63 (s, 9H). ^13^C NMR (101 MHz, DMSO-*d*_6_) δ (ppm): 152.66, 139.34, 137.14, 135.23, 132.89, 130.44, 129.20, 128.04, 127.54, 124.66, 124.19, 114.19, 56.36, 37.34, 32.78, 31.93, 31.56, 31.50, 20.14. ESI-HRMS calcd for C_22_H_28_OCl [M - H] ^-^ 343.18232, found 343.18179. HPLC: t_R_ = 5.39 min, normalization method purity 98.31%. (Figure S24)

2-(4-chloro-2-methylbenzyl)-4-(2,4,4-trimethylpentan-2-yl)phenol (**B2**). White solid; mp 59.4-61.1 °C; yield: 35%. ^1^H NMR (500 MHz, DMSO-*d*_6_) δ (ppm): 9.10 (s, 1H), 7.22 (d, *J* = 2.4 Hz, 1H), 7.12 (dd, *J* = 8.1, 2.4 Hz, 1H), 7.01 (dd, *J* = 8.4, 2.5 Hz, 1H), 6.97 (d, *J* = 8.2 Hz, 1H), 6.83 (s, 1H), 6.72 (d, *J* = 8.2 Hz, 1H), 3.80 (s, 2H), 2.23 (s, 3H), 1.56 (s, 2H), 1.20 (s, 6H), 0.61 (s, 9H). ^13^C NMR (101 MHz, DMSO-*d*_6_) δ (ppm):152.63, 139.35, 138.59, 138.32, 130.67, 130.05, 129.14, 127.77, 125.27, 124.56, 124.52, 114.20, 56.34, 37.34, 32.38, 31.90, 31.55, 31.47, 18.88. ESI-HRMS calcd for C_22_H_28_OCl [M - H] ^-^ 343.18232, found 343.18171. HPLC: t_R_ = 5.19 min, normalization method purity 98.28%. (Figure S25)

2-(4-isopropylbenzyl)-4-(2,4,4-trimethylpentan-2-yl)phenol (**B3**). Colorless [oil](javascript:;); yield: 42%. ^1^H NMR (500 MHz, DMSO-*d*_6_) δ (ppm): 9.10 (s, 1H), 7.28 (d, *J* = 7.7 Hz, 1H), 7.18 (dt, *J* = 7.7, 3.8 Hz, 1H), 7.09 – 7.04 (m, 1H), 7.00 (m, 2H), 6.74 – 6.68 (m, 2H), 3.89 (s, 2H), 3.14 (p, *J* = 6.6 Hz, 1H), 1.52 (s, 2H), 1.15 (s, 6H), 1.13 (d, *J* = 6.8 Hz, 6H), 0.59 (s, 9H). ^13^C NMR (101 MHz, DMSO-*d*_6_) δ (ppm): 152.42, 145.42, 139.40, 138.87, 128.38, 128.31, 126.28, 125.82, 124.37, 114.29, 56.40, 37.36, 35.23, 33.02, 31.95, 31.58, 31.52, 23.98. ESI-HRMS calcd for C_24_H_33_O [M - H] ^-^ 337.25259, found 337.25219. HPLC: t_R_ = 5.83 min, normalization method purity 99.51%. (Figure S26)

2-(2-isopropylbenzyl)-4-(2,4,4-trimethylpentan-2-yl)phenol (**B4**). Colorless [oil](javascript:;); yield: 37%. ^1^H NMR (500 MHz, DMSO-*d*_6_) δ (ppm): 8.99 (s, 1H), 7.08 (s, 4H), 7.04 (d, *J* = 2.5 Hz, 1H), 6.98 (dd, *J* = 8.3, 2.4 Hz, 1H), 6.68 (d, *J* = 8.4 Hz, 1H), 3.80 (s, 2H), 2.81 (p, *J* = 6.9 Hz, 1H), 1.60 (s, 2H), 1.24 (s, 6H), 1.15 (d, *J* = 6.9 Hz, 6H), 0.63 (s, 9H). ^13^C NMR (101 MHz, DMSO) δ (ppm): 152.29, 146.57, 139.23, 137.29, 129.90, 127.25, 126.34, 125.91, 125.29, 124.81, 124.13, 113.98, 56.26, 37.34, 32.04, 31.88, 31.54, 31.48, 28.21, 23.63. ESI-HRMS calcd for C_24_H_33_O [M - H] ^-^ 337.25259, found 337.25219. HPLC: t_R_ = 5.67 min, normalization method purity 97.69%. (Figure S27)

2-(4-methylbenzyl)-4-(2,4,4-trimethylpentan-2-yl)phenol (**B5**). White solid; mp 46.0-47.7 °C; yield: 36%. ^1^H NMR (600 MHz, Chloroform-*d*) δ (ppm): 7.12 (t, *J* = 2.6 Hz, 1H), 7.09 (s, 5H), 6.71 (d, *J* = 8.2 Hz, 1H), 3.95 (s, 2H), 2.31 (s, 3H), 1.68 (s, 2H), 1.33 (s, 6H), 0.72 (s, 9H). ^13^C NMR (151 MHz, CDCl_3_) δ (ppm): 151.71, 142.34, 137.22, 135.82, 129.38, 129.10, 128.55, 126.11, 125.48, 115.25, 57.20, 38.05, 36.61, 32.47, 31.92, 31.84, 21.14. ESI-HRMS calcd for C_22_H_29_O [M - H] ^-^ 309.22129, found 309.22104. HPLC: t_R_ = 11.71 min, normalization method purity 99.49%. (Figure S28)

2-(3-methylbenzyl)-4-(2,4,4-trimethylpentan-2-yl)phenol (**B6**). White solid; mp 47.7-49.4 °C; yield: 33%. ^1^H NMR (600 MHz, Chloroform-*d*) δ (ppm): 7.18 (t, *J* = 7.9 Hz, 1H), 7.13 (d, *J* = 8.1 Hz, 2H), 7.03 – 6.99 (m, 3H), 6.71 (d, *J* = 7.7 Hz, 1H), 3.96 (s, 2H), 2.29 (s, 3H), 1.69 (s, 2H), 1.34 (s, 6H), 0.72 (s, 9H). ^13^C NMR (151 MHz, CDCl_3_) δ (ppm): 151.59, 142.52, 140.11, 138.36, 129.42, 129.26, 128.64, 127.21, 125.92, 125.67, 125.60, 115.32, 57.24, 38.07, 36.93, 32.49, 31.93, 31.86, 21.54. ESI-HRMS calcd for C_22_H_29_O [M - H] ^-^ 309.22129, found 309.22105. HPLC: t_R_ = 11.75 min, normalization method purity 98.03%. (Figure S29)

2-(2-methylbenzyl)-4-(2,4,4-trimethylpentan-2-yl)phenol (**B7**). Colorless oil; yield: 39%. ^1^H NMR (600 MHz, Chloroform-*d*) δ (ppm): 7.20 (dd, *J* = 7.4, 1.7 Hz, 1H), 7.17 (dd, *J* = 7.3, 1.5 Hz, 1H), 7.15 – 7.11 (m, 2H), 7.03 – 7.01 (m, 1H), 6.98 (d, *J* = 2.5 Hz, 1H), 6.73 (d, *J* = 8.3 Hz, 1H), 3.96 (s, 2H), 2.32 (s, 3H), 1.64 (s, 2H), 1.29 (s, 6H), 0.69 (s, 9H). ^13^C NMR (151 MHz, CDCl_3_) δ (ppm): 151.58, 142.58, 138.03, 136.88, 130.43, 128.96, 128.90, 126.66, 126.30, 125.38, 125.08, 115.05, 57.10, 38.05, 34.34, 32.42, 31.90, 31.84, 19.77. ESI-HRMS calcd for C_22_H_29_O [M - H] ^-^ 309.22129, found 309.22073. HPLC: t_R_ = 11.52 min, normalization method purity 99.21%. (Figure S30)

2-(2,4-dimethylbenzyl)-4-(2,4,4-trimethylpentan-2-yl)phenol (**B8**). White solid; mp 44.4-46.0 °C; yield: 35%. ^1^H NMR (600 MHz, Chloroform-*d*) δ (ppm): 7.11 (dd, *J* = 8.4, 2.5 Hz, 1H), 7.00 (d, *J* = 2.7 Hz, 2H), 6.94 – 6.91 (m, 1H), 6.89 (d, *J* = 7.7 Hz, 1H), 6.71 (d, *J* = 8.4 Hz, 1H), 3.91 (s, 2H), 2.29 (s, 3H), 2.27 (s, 3H), 1.64 (s, 2H), 1.29 (s, 6H), 0.69 (s, 9H). ^13^C NMR (151 MHz, CDCl_3_) δ (ppm): 151.71, 142.53, 136.69, 136.18, 134.77, 131.36, 129.01, 128.73, 126.95, 125.39, 125.21, 115.13, 57.12, 38.06, 34.24, 32.43, 31.90, 31.85, 21.06, 19.72. ESI-HRMS calcd for C_23_H_31_O [M - H] ^-^ 323.23694, found 323.23666. HPLC: t_R_ = 8.86 min, normalization method purity 98.93%. (Figure S31)

2-(3,5-dimethylbenzyl)-4-(2,4,4-trimethylpentan-2-yl)phenol (**B9**). Colorless oil; yield: 43%. ^1^H NMR (400 MHz, Chloroform-*d*) δ (ppm): 7.15 – 7.11 (m, 2H), 6.84 (s, 1H), 6.81 (s, 2H), 6.71 (dd, *J* = 8.0, 0.7 Hz, 1H), 3.92 (s, 2H), 2.25 (s, 6H), 1.69 (s, 2H), 1.34 (s, 6H), 0.73 (s, 9H). ^13^C NMR (101 MHz, CDCl_3_) δ (ppm): 151.65, 142.40, 139.94, 138.30, 129.29, 128.17, 126.42, 125.91, 125.60, 115.36, 57.26, 38.06, 36.93, 32.50, 31.92, 31.87, 21.41. ESI-HRMS calcd for C_23_H_32_ONa [M + Na] ^+^ 347.23454, found 347.23477. HPLC: t_R_ = 5.38 min, normalization method purity 95.07%. (Figure S32)

2-(2,3-dimethylbenzyl)-4-(2,4,4-trimethylpentan-2-yl)phenol (**B10**). Slight yellow solid; yield: 41%. ^1^H NMR (400 MHz, Chloroform-*d*) δ (ppm): 7.12 (dd, *J* = 8.4, 2.5 Hz, 1H), 7.09 – 7.01 (m, 2H), 6.97 (d, *J* = 2.4 Hz, 1H), 6.92 – 6.88 (m, 1H), 6.72 (d, *J* = 8.4 Hz, 1H), 3.98 (s, 2H), 2.31 (s, 3H), 2.19 (s, 3H), 1.63 (s, 2H), 1.29 (s, 6H), 0.68 (s, 9H). ^13^C NMR (101 MHz, CDCl_3_) δ (ppm): 151.59, 142.49, 137.77, 137.22, 135.46, 128.94, 128.54, 126.87, 125.77, 125.34, 125.31, 115.05, 57.06, 38.03, 35.12, 32.41, 31.87, 31.85, 20.81, 15.40. ESI-HRMS calcd for C_23_H_32_ONa [M + Na] ^+^ 347.23454, found 347.23502. HPLC: t_R_ = 4.96 min, normalization method purity 99.15%. (Figure S33)

2-(2,6-dimethylbenzyl)-4-(2,4,4-trimethylpentan-2-yl)phenol (**B11**). White solid; mp 59.4-61.7 °C; yield: 39%. ^1^H NMR (400 MHz, Chloroform-*d*) δ (ppm): 7.14 – 7.06 (m, 3H), 7.05 – 7.01 (m, 1H), 6.69 (d, *J* = 8.3 Hz, 1H), 6.56 – 6.54 (m, 1H), 3.98 (s, 2H), 2.22 (s, 6H), 1.48 (s, 2H), 1.16 (s, 6H), 0.58 (s, 9H). ^13^C NMR (101 MHz, CDCl_3_) δ (ppm): 151.37, 142.24, 137.43, 136.25, 128.27, 126.74, 126.57, 124.55, 124.49, 114.30, 56.81, 37.91, 32.23, 31.79, 31.76, 29.36, 20.15. ESI-HRMS calcd for C_23_H_32_ONa [M + Na] ^+^ 347.23454, found 347.23481. HPLC: t_R_ = 5.34 min, normalization method purity 98.39%. (Figure S34)

2-(3,4-dimethylbenzyl)-4-(2,4,4-trimethylpentan-2-yl)phenol (**B12**). Colorless oil; yield: 45%. ^1^H NMR (400 MHz, Chloroform-*d*) δ (ppm): 7.17 – 7.11 (m, 2H), 7.05 (d, *J* = 7.6 Hz, 1H), 6.98 – 6.92 (m, 2H), 6.71 (d, *J* = 8.3 Hz, 1H), 3.94 (s, 2H), 2.22 (s, 3H), 2.20 (s, 3H), 1.70 (s, 2H), 1.35 (s, 6H), 0.74 (s, 9H). ^13^C NMR (101 MHz, CDCl_3_) δ (ppm): 151.65, 142.42, 137.36, 136.96, 134.68, 130.03, 129.89, 129.20, 126.02, 125.91, 125.59, 115.36, 57.23, 38.06, 36.77, 32.49, 31.94, 31.87, 19.91, 19.47. ESI-HRMS calcd for C_23_H_32_ONa [M + Na] ^+^ 347.23454, found 347.23457. HPLC: t_R_ = 5.25 min, normalization method purity 97.74%. (Figure S35)

2-(2,5-dimethylbenzyl)-4-(2,4,4-trimethylpentan-2-yl)phenol (**B13**). Colorless oil; yield: 40%. ^1^H NMR (400 MHz, Chloroform-*d*) δ (ppm): 7.14 (dd, *J* = 8.4, 2.5 Hz, 1H), 7.09 (d, *J* = 7.6 Hz, 1H), 7.03 (d, *J* = 2.5 Hz, 1H), 6.99 – 6.96 (m, 1H), 6.84 – 6.81 (m, 1H), 6.74 (d, *J* = 8.3 Hz, 1H), 3.93 (s, 2H), 2.29 (s, 3H), 2.24 (s, 3H), 1.67 (s, 2H), 1.32 (s, 6H), 0.72 (s, 9H). ^13^C NMR (101 MHz, CDCl_3_) δ (ppm): 151.63, 142.42, 137.77, 135.74, 133.65, 130.33, 129.50, 129.08, 127.27, 125.40, 125.11, 115.13, 57.12, 38.02, 34.29, 32.43, 31.90, 31.86, 21.11, 19.32. ESI-HRMS calcd for C_23_H_32_ONa [M + Na] ^+^ 347.23454, found 347.23473. HPLC: t_R_ = 5.40 min, normalization method purity 98.51%. (Figure S36)

**4. General procedure for preparation of 5a-d**

**1h** (2 equiv) was dissolved in anhydrous DCM (20 mL) with [appropriate](javascript:;)ly substituted 2-pyridinecarboxaldehyde (1 equiv), MgCl_2_ (3.5 equiv), *N*,*N*-dimethylamine (2 equiv), and the mixture was stirred for 10 h at room temperature. Distilled water (30 mL) was added and extracted with dichloromethane (25 mL × 3). The merged organic layer was dried with anhydrous Na_2_SO_4_, concentrated under reduced pressure, and purified over silica gel to give the target compounds.

2-(hydroxy(5-methylpyridin-2-yl) methyl)-4-(2,4,4-trimethylpentan-2-yl)phenol (**5a**). White solid; mp 119.3-122.6 °C; yield: 73%. ^1^H NMR (600 MHz, DMSO-*d*_6_) δ (ppm): 9.65 (s, 1H), 8.31 – 8.27 (m, 1H), 7.59 (ddd, *J* = 8.0, 2.3, 0.8 Hz, 1H), 7.37 – 7.28 (m, 2H), 7.03 – 6.97 (m, 1H), 6.66 (d, *J* = 8.4 Hz, 1H), 5.91 (d, *J* = 4.6 Hz, 1H), 5.86 (d, *J* = 4.8 Hz, 1H), 2.25 (s, 3H), 1.64 – 1.55 (m, 2H), 1.25 (d, *J* = 8.3 Hz, 6H), 0.61 (s, 9H). ESI-MS calcd for C_21_H_30_NO_2_ [M + H] ^+^ 328, found 328. (Figure S37)

2-(hydroxy(6-methylpyridin-2-yl) methyl)-4-(2,4,4-trimethylpentan-2-yl)phenol (**5b**). Slight pink solid; mp 176.3-179.5 °C; yield:70 %. ^1^H NMR (600 MHz, DMSO-*d*_6_) δ (ppm): 9.96 (s, 1H), 7.68 (t, *J* = 7.7 Hz, 1H), 7.32 (d, *J* = 2.5 Hz, 1H), 7.29 (d, *J* = 7.7 Hz, 1H), 7.12 (d, *J* = 7.6 Hz, 1H), 7.03 (dd, *J* = 8.4, 2.5 Hz, 1H), 6.67 (d, *J* = 8.4 Hz, 1H), 5.91 (d, *J* = 4.8 Hz, 1H), 5.88 (d, *J* = 4.6 Hz, 1H), 2.43 (s, 3H), 1.64 – 1.57 (m, 2H), 1.25 (d, *J* = 13.3 Hz, 6H), 0.61 (s, 9H). ESI-MS calcd for C_21_H_30_NO_2_ [M + H] ^+^ 328, found 328. (Figure S38)

2-(hydroxy(3-methylpyridin-2-yl) methyl)-4-(2,4,4-trimethylpentan-2-yl)phenol (**5c**). White solid; mp 139.3-146.0 °C; yield:76%. ^1^H NMR (600 MHz, DMSO-*d*_6_) δ (ppm): 9.43 (s, 1H), 8.41 (dd, *J* = 5.0, 1.6 Hz, 1H), 7.54 (ddd, *J* = 7.6, 1.7, 0.9 Hz, 1H), 7.22 (dd, *J* = 7.5, 4.8 Hz, 1H), 7.04 – 6.99 (m, 2H), 6.67 (d, *J* = 9.1 Hz, 1H), 6.07 (d, *J* = 5.2 Hz, 1H), 5.69 (d, *J* = 5.9 Hz, 1H), 2.18 (s, 3H), 1.53 (s, 2H), 1.19 (d, *J* = 28.2 Hz, 6H), 0.53 (s, 9H). ESI-MS calcd for C_21_H_30_NO_2_ [M + H] ^+^ 328, found 328. (Figure S39)

2-(hydroxy(4-methylpyridin-2-yl) methyl)-4-(2,4,4-trimethylpentan-2-yl)phenol (**5d**). White solid; mp 142.6-146.7 °C; yield:71 %. ^1^H NMR (600 MHz, DMSO-*d*_6_) δ (ppm): 9.77 (s, 1H), 8.31 (dd, *J* = 5.0, 0.8 Hz, 1H), 7.35 (d, *J* = 2.5 Hz, 1H), 7.28 (s, 1H), 7.15 – 6.99 (m, 2H), 6.66 (d, *J* = 8.4 Hz, 1H), 5.90 (q, *J* = 4.7 Hz, 2H), 2.29 (s, 3H), 1.66 – 1.54 (m, 2H), 1.25 (d, *J* = 8.6 Hz, 6H), 0.62 (s, 9H). ESI-MS calcd for C_21_H_30_NO_2_ [M + H] ^+^ 328, found 328. (Figure S40)

**5. General Procedure for preparation of C1-C4**

Compounds **5a**-**d** were dissolved in anhydrous DCM (5 mL), TFA (2 mL) and Et_3_SiH (2 equiv) was added respectively, and then the mixture was stirred for 40 h at room temperature. Distilled water (30 mL) was added and extracted with ethyl acetate (25 mL × 3) were added. The merged organic layer was dried with anhydrous Na_2_SO_4_, concentrated under reduced pressure, and purified over silica gel to give the target compounds.

2-((5-methylpyridin-2-yl) methyl)-4-(2,4,4-trimethylpentan-2-yl)phenol (**C1**). White solid; mp 142.6-145.7 °C; yield: 43%.^1^H NMR (400 MHz, DMSO-*d*_6_) δδ (ppm): 9.59 (s, 1H), 8.30 (dt, *J* = 2.3, 0.8 Hz, 1H), 7.49 (ddd, *J* = 7.9, 2.3, 0.9 Hz, 1H), 7.09 (d, *J* = 2.5 Hz, 1H), 7.06 (dd, *J* = 7.8, 0.8 Hz, 1H), 7.03 (dd, *J* = 8.4, 2.5 Hz, 1H), 6.71 (d, *J* = 8.4 Hz, 1H), 3.97 (s, 2H), 2.24 (s, 3H), 1.61 (s, 2H), 1.25 (s, 6H), 0.64 (s, 9H). ^13^C NMR (101 MHz, DMSO-*d*_6_) δ (ppm): 158.05, 152.78, 148.54, 139.66, 137.18, 130.13, 128.37, 124.94, 124.86, 121.99, 114.88, 56.41, 38.40, 37.39, 31.97, 31.55, 31.52, 17.47. ESI-HRMS calcd for C_21_H_30_ON [M + H] ^+^ 312.23219, found 312.23224. HPLC: t_R_ = 7.69 min, normalization method purity 98.92%. (Figure S41)

2-((6-methylpyridin-2-yl) methyl)-4-(2,4,4-trimethylpentan-2-yl)phenol (**C2**). White solid; mp 129.4-132.3 °C; yield: 46%. ^1^H NMR (400 MHz, DMSO-*d*_6_) δ (ppm): 9.82 (s, 1H), 7.57 (t, *J* = 7.7 Hz, 1H), 7.11 (d, *J* = 2.5 Hz, 1H), 7.06 (d, *J* = 7.6 Hz, 1H), 7.02 (dd, *J* = 8.4, 2.5 Hz, 1H), 6.97 (d, *J* = 7.7 Hz, 1H), 6.71 (d, *J* = 8.4 Hz, 1H), 3.97 (s, 2H), 2.43 (s, 3H), 1.61 (s, 2H), 1.24 (s, 6H), 0.63 (s, 9H). ^13^C NMR (101 MHz, DMSO-*d*_6_) δ (ppm): 160.21, 156.68, 152.92, 139.70, 137.26, 128.50, 124.90, 124.83, 120.62, 119.52, 115.06, 56.41, 38.96, 37.41, 31.99, 31.59, 31.55, 23.82. ESI-HRMS calcd for C_21_H_30_ON [M + H]^+^ 312.23219, found 312.23242. HPLC: t_R_ = 7.65 min, normalization method purity 98.63%. (Figure S42)

2-((3-methylpyridin-2-yl) methyl)-4-(2,4,4-trimethylpentan-2-yl)phenol (**C3**). White solid; mp 108.9-112.3 °C.; yield: 48%. ^1^H NMR (400 MHz, DMSO-*d*_6_) δ (ppm): 9.68 (s, 1H), 8.34 (ddd, *J* = 4.9, 1.8, 0.7 Hz, 1H), 7.56 (ddd, *J* = 7.6, 1.8, 0.9 Hz, 1H), 7.17 (dd, *J* = 7.6, 4.8 Hz, 1H), 6.99 (dd, *J* = 8.4, 2.5 Hz, 1H), 6.84 (d, *J* = 2.5 Hz, 1H), 6.71 (d, *J* = 8.3 Hz, 1H), 4.03 (s, 2H), 2.25 (s, 3H), 1.52 (s, 2H), 1.18 (s, 6H), 0.57 (s, 9H). ^13^C NMR (101 MHz, DMSO-*d*_6_) δ (ppm): 158.99, 152.72, 145.88, 139.37, 138.01, 131.30, 127.36, 124.49, 124.18, 121.69, 114.75, 56.31, 37.28, 35.67, 31.84, 31.49, 31.43, 18.12. ESI-HRMS calcd for C_21_H_30_ON [M + H]^+^ 312.23219, found 312.23193. HPLC: t_R_ = 7.63 min, normalization method purity 98.98%. (Figure S43)

2-((4-methylpyridin-2-yl) methyl)-4-(2,4,4-trimethylpentan-2-yl)phenol (**C4**). White solid; mp 132.5-137.7 °C; yield: 53%. ^1^H NMR (400 MHz, DMSO-*d*_6_) δ (ppm): 9.68 (s, 1H), 8.31 (dd, *J* = 5.1, 0.8 Hz, 1H), 7.09 (d, *J* = 2.5 Hz, 1H), 7.05 – 7.01 (m, 2H), 6.97 (dd, *J* = 1.7, 0.9 Hz, 1H), 6.71 (d, *J* = 8.3 Hz, 1H), 3.97 (s, 2H), 2.21 (s, 3H), 1.61 (s, 2H), 1.25 (s, 6H), 0.64 (s, 9H). ^13^C NMR (101 MHz, DMSO-*d*_6_) δ (ppm): 160.83, 152.85, 148.17, 147.40, 139.67, 128.52, 124.98, 124.77, 123.19, 122.14, 115.01, 56.47, 38.68, 37.41, 31.99, 31.57, 31.53, 20.52. ESI-HRMS calcd for C_21_H_30_ON [M + H] ^+^ 312.23219, found 312.23233. HPLC: t_R_ = 7.61 min, normalization method purity 98.39%. (Figure S44)

**6. The synthesis procedure for preparation of 6**

2-nitro-4-(2,4,4-trimethylpentan-2-yl)phenol compounds (**6**). 4-(2,4,4-trimethylpentan-2-yl) phenol(**1h**) (2.06 g, 10 mmol) was dissolved in acetic acid (20 mL), and concentrated nitric acid (1 mL) was added drop by drop and stirred for 30min at room temperature. It was distilled under reduced pressure, distilled water (30 mL) was added, and extracted with ethyl acetate (25 mL × 3). The merged organic layer was dried with anhydrous Na_2_SO_4_, concentrated under reduced pressure, and purified by silica gel to obtain an orange oil (2.13 g, 85% yield). ^1^H NMR (600 MHz, Chloroform-*d*) δ (ppm): 10.47 (s, 1H), 8.05 (s, 1H), 7.63 (d, *J* = 8.8 Hz, 1H), 7.08 (d, *J* = 8.9 Hz, 1H), 1.73 (s, 2H), 1.37 (s, 6H), 0.74 (s, 9H). ESI-MS calcd for C_14_H_22_NO_3_ [M + H] ^+^ 252, found 252. (Figure S45)

**7. The synthesis procedure for preparation of 7**

2-amino-4-(2,4,4-trimethylpentan-2-yl)phenol compounds (**7**). Compound **6** (1255 mg, 5 mmol) was dissolved in methanol (20 mL), formate amine (630 mg, 10 mmol) and 30% Pd/C (251 mg, 20% w/w) were added and stirred for 2 h at room temperature. Pd/C was filtered, distilled under reduced pressure, distilled water (30 mL) was added, and extracted with ethyl acetate (25 mL × 3). The consolidated organic layer was dried with anhydrous Na_2_SO_4_, concentrated under reduced pressure, and purified by column chromatography to obtain a brown solid (960 mg, 87% yield). mp 121.5-124.3 °C. ^1^H NMR (400 MHz, DMSO-*d*_6_) δ (ppm): 8.65 (s, 1H), 6.64 (d, *J* = 2.3 Hz, 1H), 6.53 (d, *J* = 8.2 Hz, 1H), 6.40 (dd, *J* = 8.2, 2.4 Hz, 1H), 4.42 (s, 2H), 1.61 (s, 2H), 1.24 (s, 6H), 0.71 (s, 9H). ESI-MS calcd for C_14_H_24_NO [M + H] ^+^ 222, found 222. (Figure S46)

**8. General procedure for preparation of D1-D5**

Compound **7** (2 equiv) was dissolved in DMF (5 mL) and [appropriate](javascript:;)ly substituted iodobenzene (1 equiv), Cs_2_CO_3_ (2 equiv), CuI (0.2 equiv) were added. The resulting mixture was performed to microwave irradiation (Absorption level: normal, Power: 80W, Temperature: 100 °C) for 1 h. Distilled water (30 mL) was added and extracted with ethyl acetate (25 mL × 3). The merged organic layer was dried with anhydrous Na_2_SO_4_, concentrated under reduced pressure, and purified over silica gel to give the target compounds.

2-(p-tolylamino)-4-(2,4,4-trimethylpentan-2-yl)phenol (**D1**). Brown solid; mp 78.8-81.5 °C; yield: 56%. ^1^H NMR (400 MHz, DMSO-*d*_6_) δ (ppm): 9.12 (s, 1H), 7.12 (s, 1H), 6.97 (d, *J* = 8.1 Hz, 2H), 6.92 (s, 1H), 6.88 (d, *J* = 8.1 Hz, 2H), 6.73 (s, 2H), 2.19 (s, 3H), 1.62 (s, 2H), 1.24 (s, 6H), 0.71 (s, 9H). ^13^C NMR (101 MHz, DMSO-*d*_6_) δ (ppm): 145.42, 142.19, 139.86, 130.04, 129.27, 127.17, 118.63, 116.34, 116.05, 114.67, 56.42, 37.54, 32.01, 31.56, 31.54, 20.19. ESI-HRMS calcd for C_21_H_30_ON [M + H] ^+^ 312.23219, found 312.23223. HPLC: t_R_ = 10.50 min, normalization method purity 98.69%. (Figure S47)

2-((2,4-dichlorophenyl) amino)-4-(2,4,4-trimethylpentan-2-yl)phenol (**D2**). Brown oil; yield: 63%. ^1^H NMR (400 MHz, DMSO-*d*_6_) δ (ppm): 9.36 (s, 1H), 7.47 (d, *J* = 2.5 Hz, 1H), 7.17 (dd, *J* = 8.8, 2.5 Hz, 1H), 7.11 (d, *J* = 2.3 Hz, 1H), 6.97 (dd, *J* = 8.4, 2.4 Hz, 1H), 6.89 (s, 1H), 6.82 (d, *J* = 8.4 Hz, 1H), 6.68 (d, *J* = 8.8 Hz, 1H), 1.65 (s, 2H), 1.27 (s, 6H), 0.71 (s, 9H). ^13^C NMR (101 MHz, DMSO-*d*_6_) δ (ppm): 147.69, 140.93, 140.46, 128.49, 127.47, 127.02, 122.29, 121.43, 121.18, 120.11, 115.42, 115.30, 56.38, 37.56, 32.04, 31.57, 31.49. ESI-HRMS calcd for C_20_H_26_ONCl_2_ [M + H] ^+^ 366.13860, found 366.13847. HPLC: t_R_ = 13.35 min, normalization method purity 96.51%. (Figure S48)

2-(o-tolylamino)-4-(2,4,4-trimethylpentan-2-yl)phenol (**D3**). Brown solid; mp 75.5-78.6 °C; yield: 58%. ^1^H NMR (400 MHz, DMSO-*d*_6_) δ (ppm): 9.12 (s, 1H), 7.13 (ddd, *J* = 7.4, 1.7, 0.8 Hz, 1H), 7.01 (td, *J* = 7.7, 1.6 Hz, 1H), 6.94 (d, *J* = 2.1 Hz, 1H), 6.85 (dd, *J* = 8.1, 1.2 Hz, 1H), 6.80 – 6.72 (m, 3H), 6.23 (s, 1H), 2.21 (s, 3H), 1.61 (s, 2H), 1.23 (s, 6H), 0.71 (s, 9H). ^13^C NMR (101 MHz, DMSO-*d*_6_) δ (ppm): 145.67, 142.82, 140.08, 130.42, 130.19, 126.31, 126.24, 119.65, 119.17, 117.49, 115.74, 114.70, 56.39, 37.52, 32.00, 31.54, 17.73. ESI-HRMS calcd for C_21_H_30_ON [M + H] ^+^ 312.23219, found 312.23208. HPLC: t_R_ = 10.96 min, normalization method purity 99.46%. (Figure S49)

2-((2,4-dimethylphenyl) amino)-4-(2,4,4-trimethylpentan-2-yl)phenol (**D4**). Brown oil; yield: 61%. ^1^H NMR (400 MHz, DMSO-*d*_6_) δ (ppm): 9.11 (s, 1H), 6.99 – 6.96 (m, 1H), 6.85 (t, *J* = 1.2 Hz, 2H), 6.81 (d, *J* = 2.1 Hz, 1H), 6.74 – 6.67 (m, 2H), 6.10 (s, 1H), 2.21 (s, 3H), 2.16 (s, 3H), 1.58 (s, 2H), 1.20 (s, 6H), 0.70 (s, 9H). ^13^C NMR (101 MHz, DMSO-*d*_6_) δ (ppm): 144.56, 140.01, 139.71, 131.29, 131.16, 129.20, 127.64, 126.68, 117.85, 117.64, 115.35, 114.40, 56.39, 37.52, 31.99, 31.54, 31.52, 20.26, 17.66. ESI-HRMS calcd for C_22_H_32_ON [M + H] ^+^ 326.24784, found 326.24750. HPLC: t_R_ = 111.87 min, normalization method purity 98.23%. (Figure S50)

2-((4-(tert-butyl) phenyl) amino)-4-(2,4,4-trimethylpentan-2-yl)phenol (**D5**). Brown solid; mp 77.3-80.1 °C; yield: 56%. ^1^H NMR (400 MHz, DMSO-*d*_6_) δ (ppm): 9.10 (s, 1H), 7.19 – 7.16 (m, 2H), 7.14 (d, *J* = 1.9 Hz, 1H), 6.95 (s, 1H), 6.90 – 6.87 (m, 2H), 6.76 – 6.74 (m, 2H), 1.63 (s, 2H), 1.25 (s, 6H), 1.24 (s, 9H), 0.72 (s, 9H). ^13^C NMR (101 MHz, DMSO-*d*_6_) δ (ppm): 146.10, 142.74, 141.12, 140.37, 130.22, 125.88, 119.38, 117.38, 115.65, 115.15, 56.91, 38.02, 34.12, 32.50, 32.06, 32.02, 31.86. ESI-HRMS calcd for C_24_H_36_ON [M + H]^+^ 354.27914, found 354.27899.HPLC: t_R_ = 13.48 min, normalization method purity 99.18%. (Figure S51)

**Figure S8-S51.** ^1^H NMR, MS, ^13^C NMR, HRMS and HPLC data of compound.

**
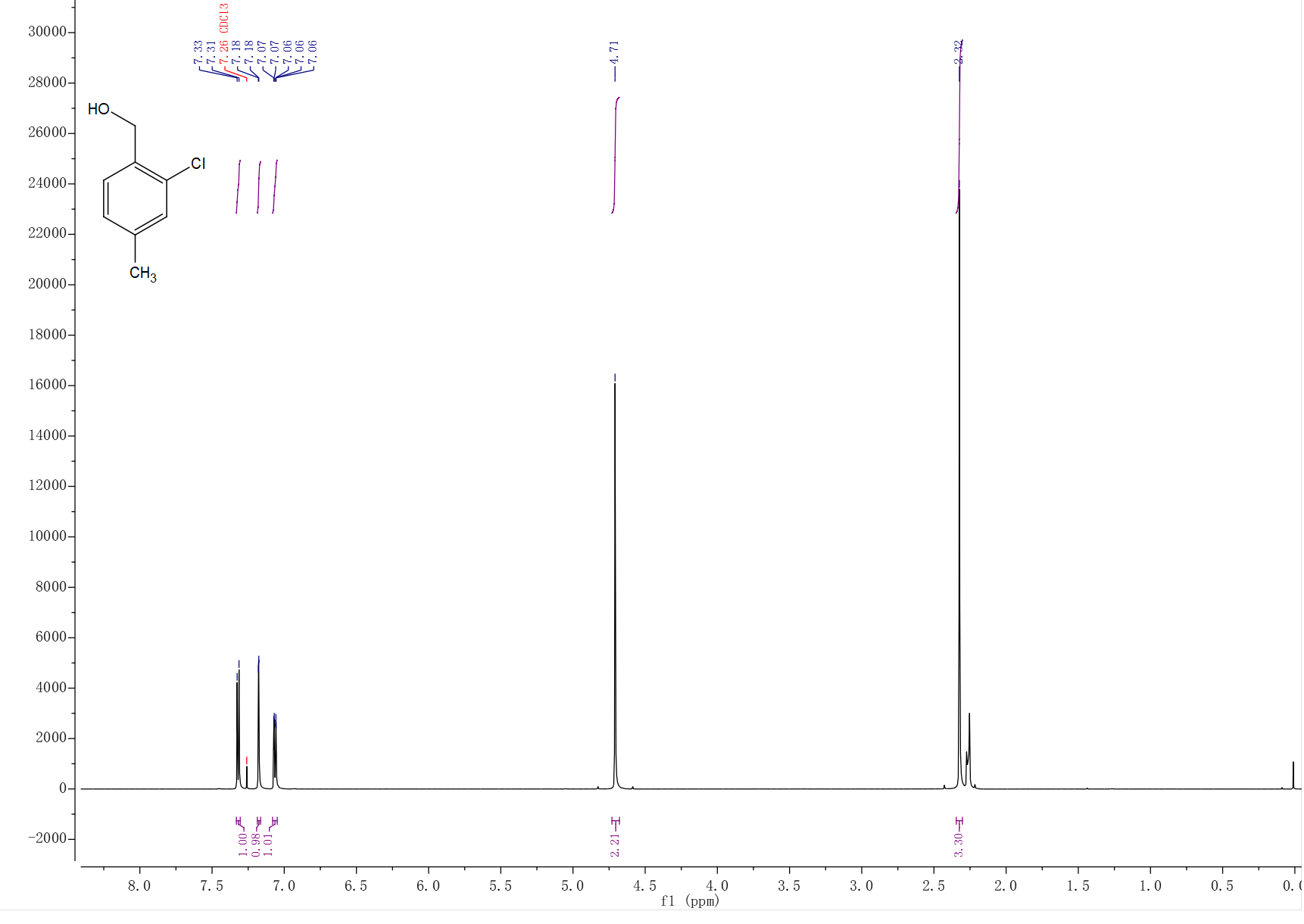
**
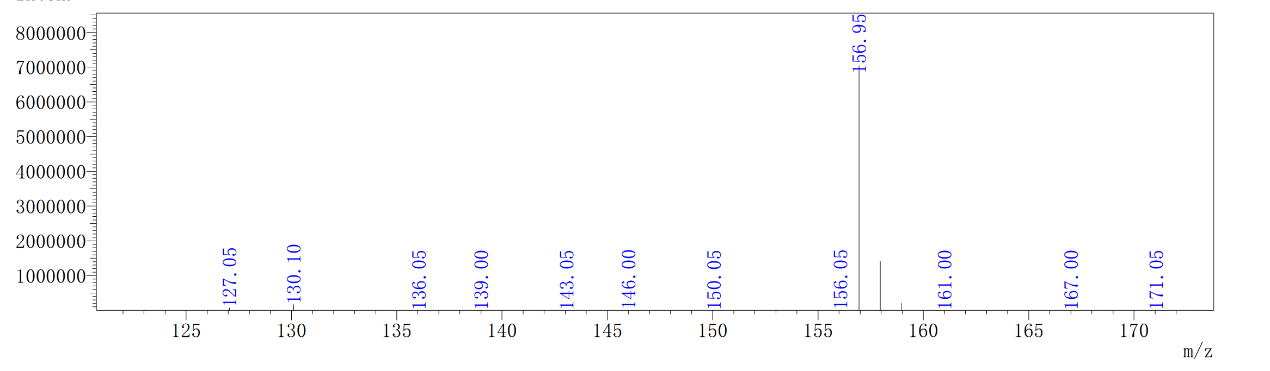
**Figure S8.** ^1^H NMR and MS data of compound **3a**.

**
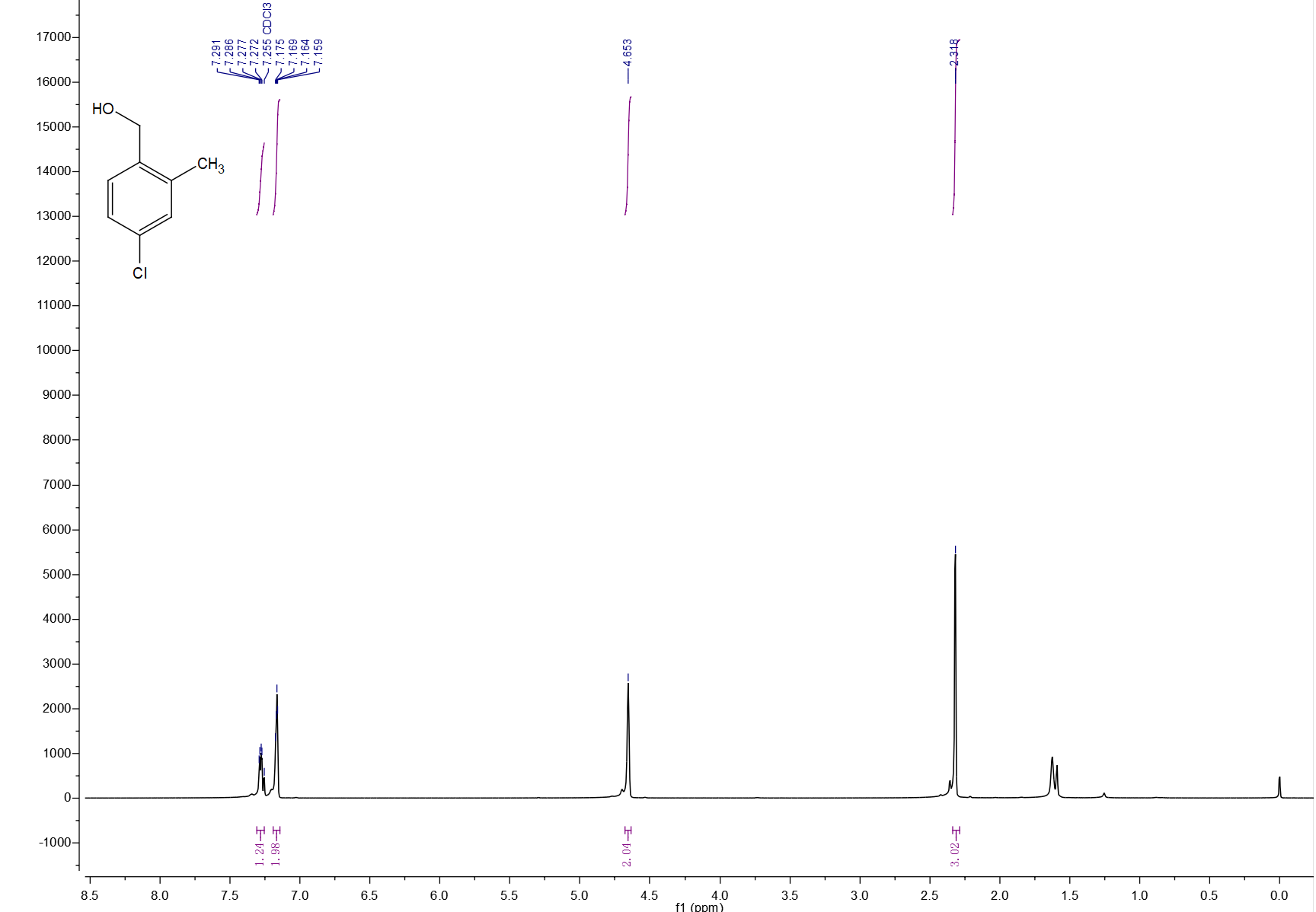
**

**
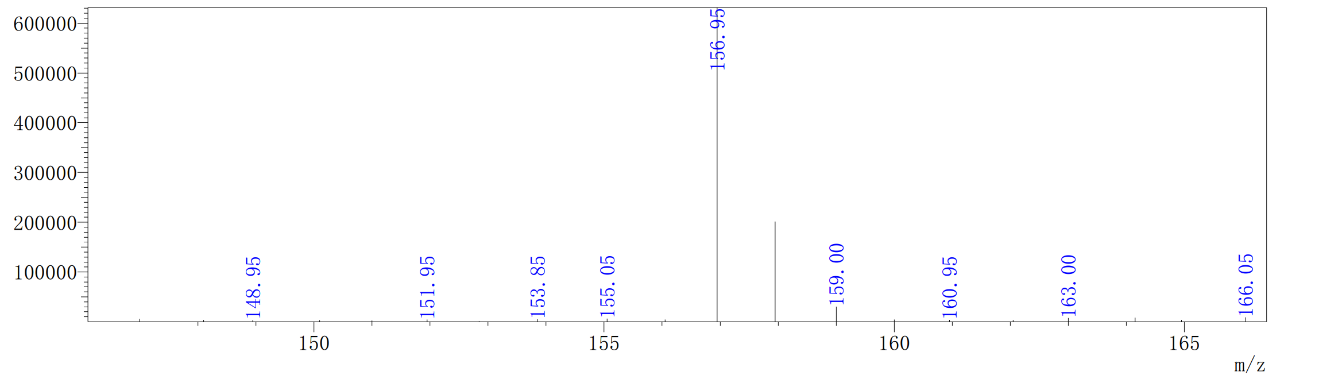
Figure S9.** ^1^H NMR and MS data of compound **3b**.

**
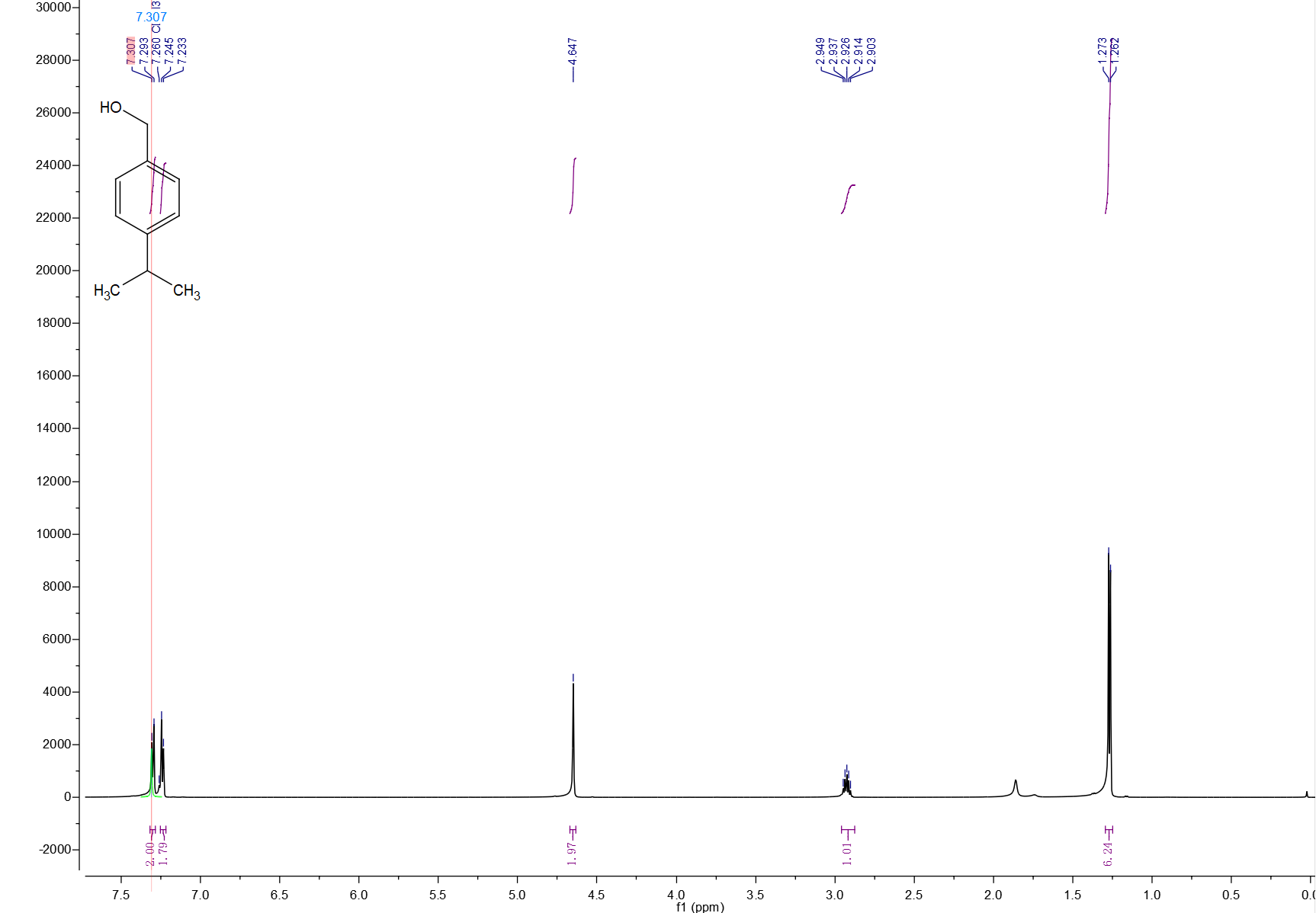
**

**
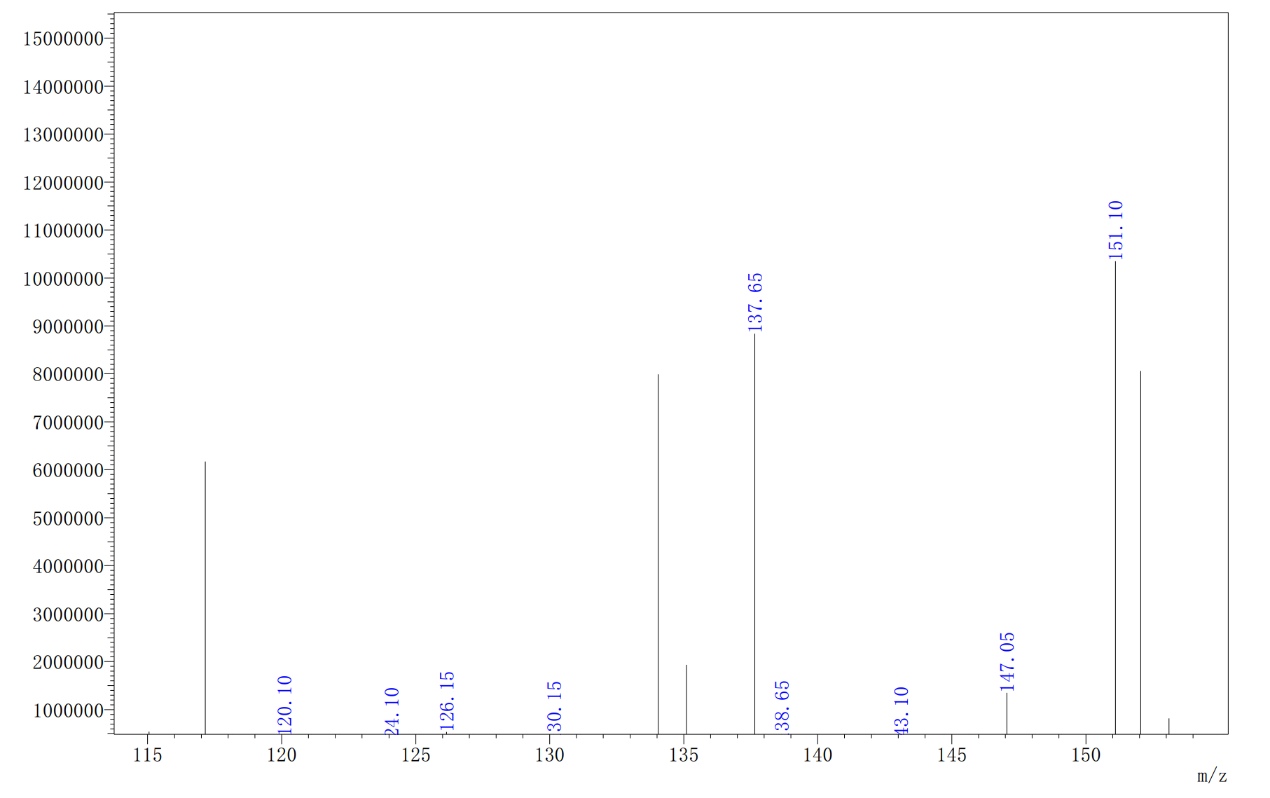
**

**Figure S10.** ^1^H NMR and MS data of compound **3c**.

**
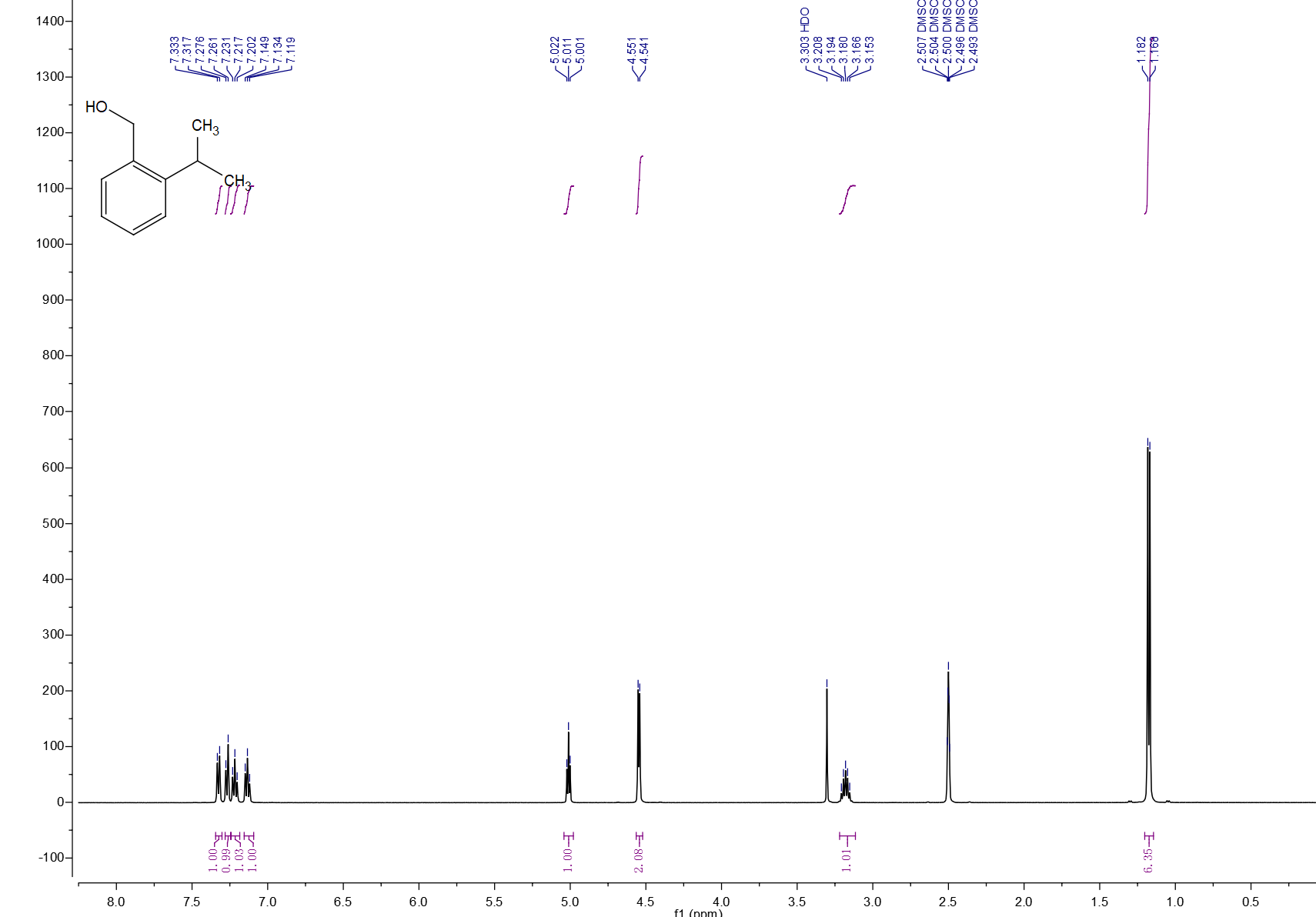

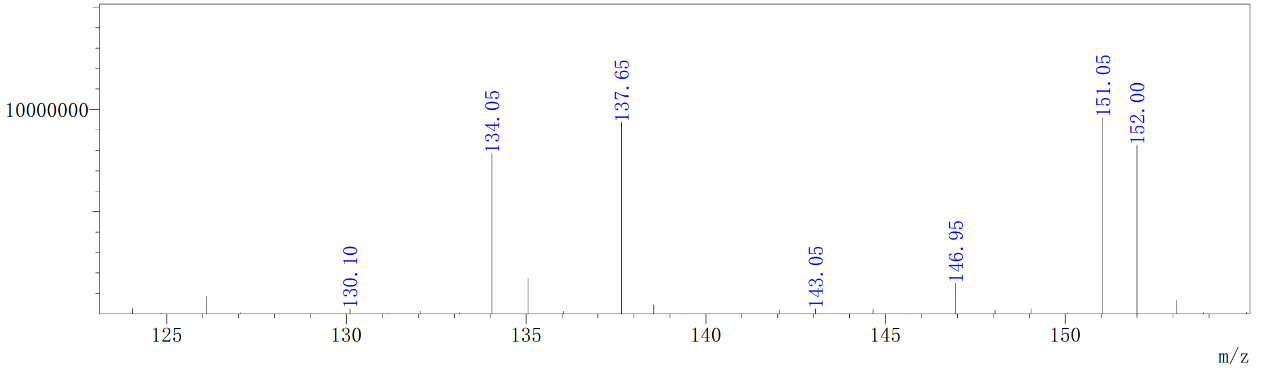
Figure S11.** ^1^H NMR and MS data of compound **3d**.


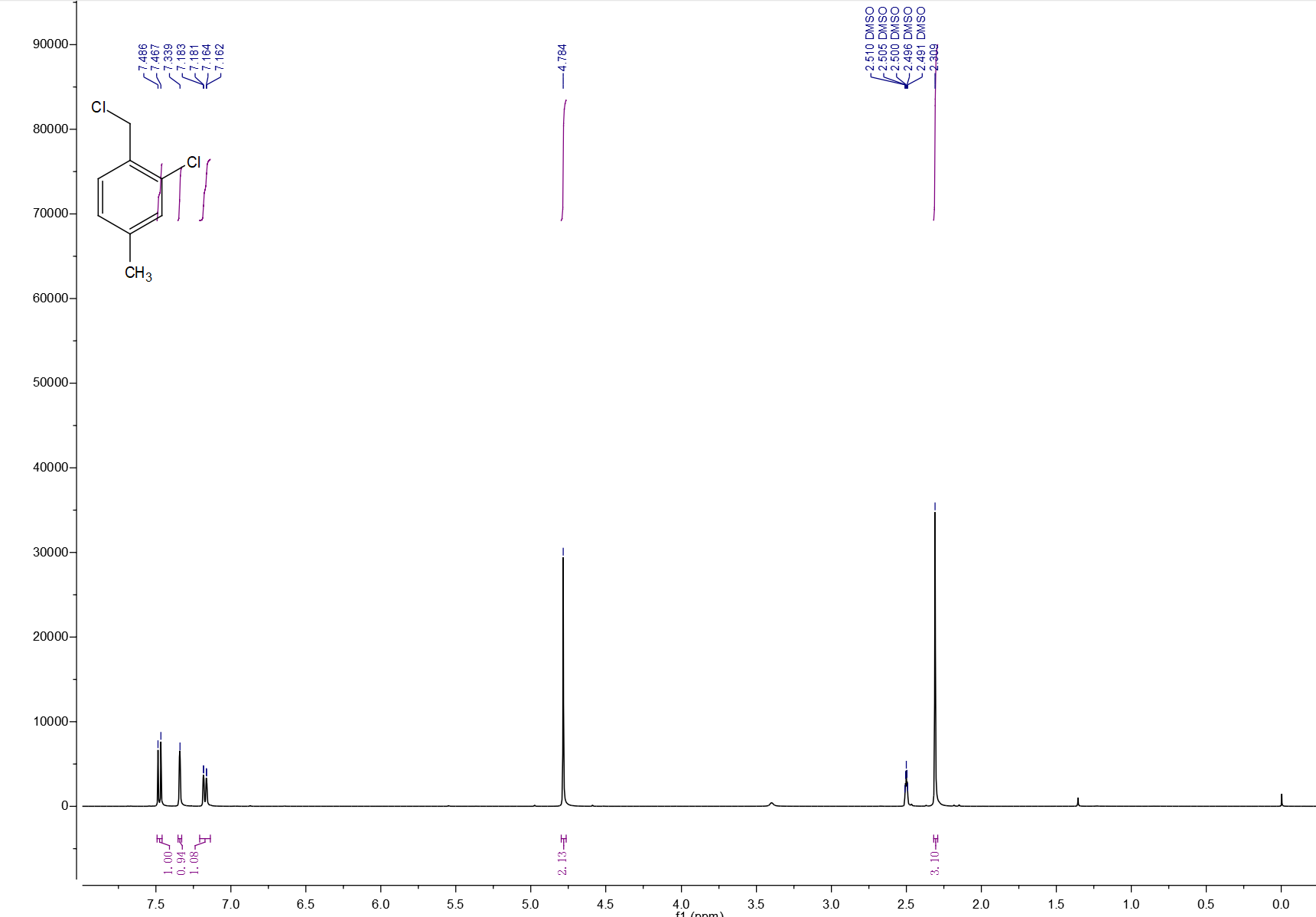

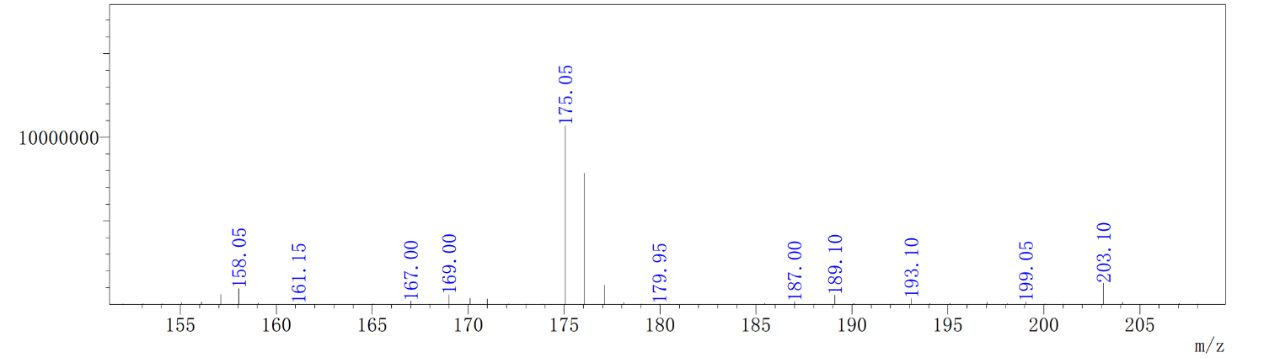


**Figure S12.** ^1^H NMR and MS data of compound **4a**.


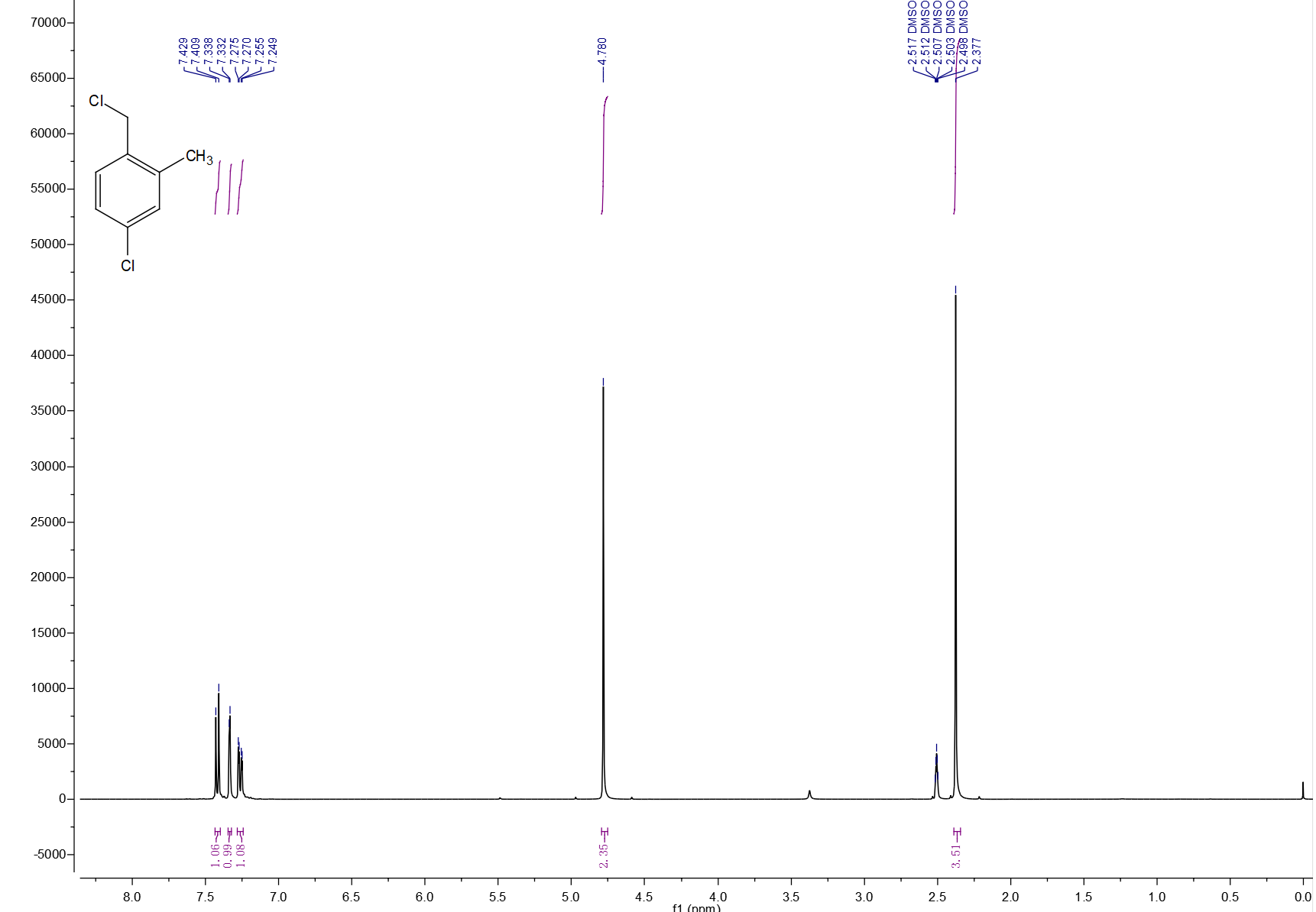


**
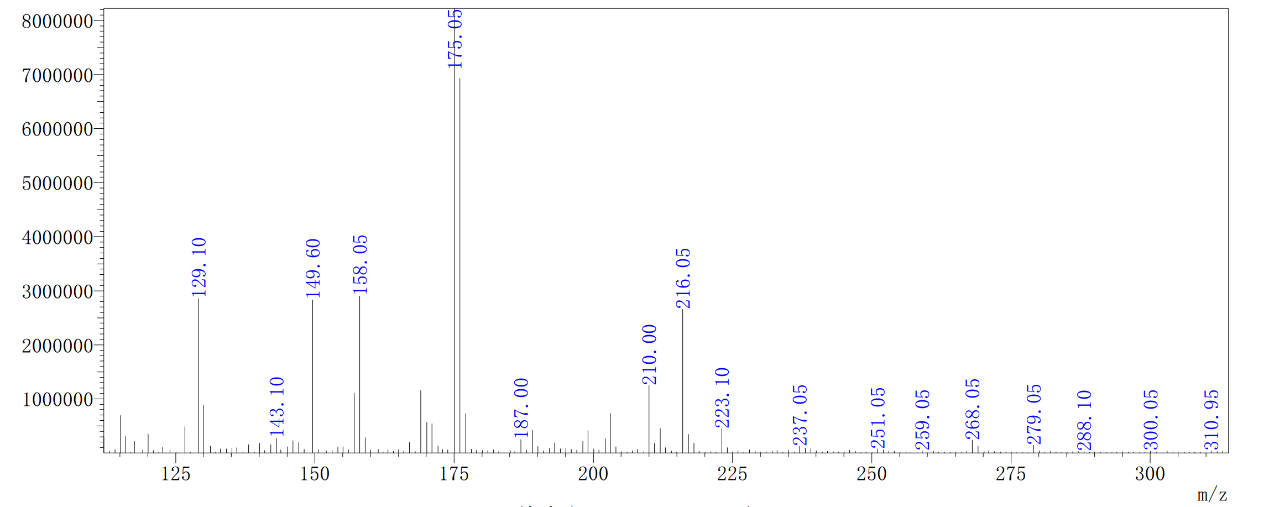
**

**Figure S13.** ^1^H NMR and MS data of compound **4b**.

**
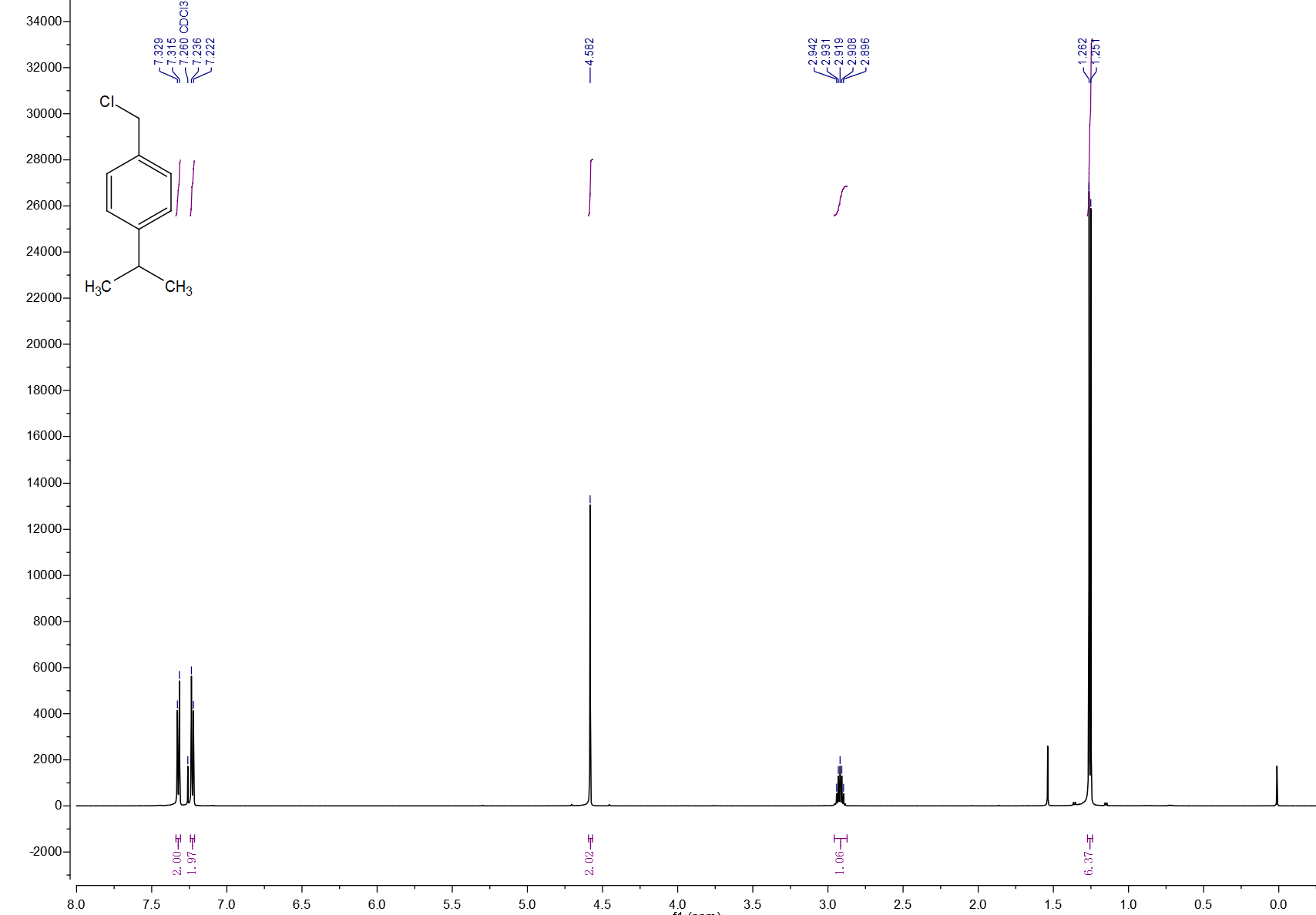
**

**
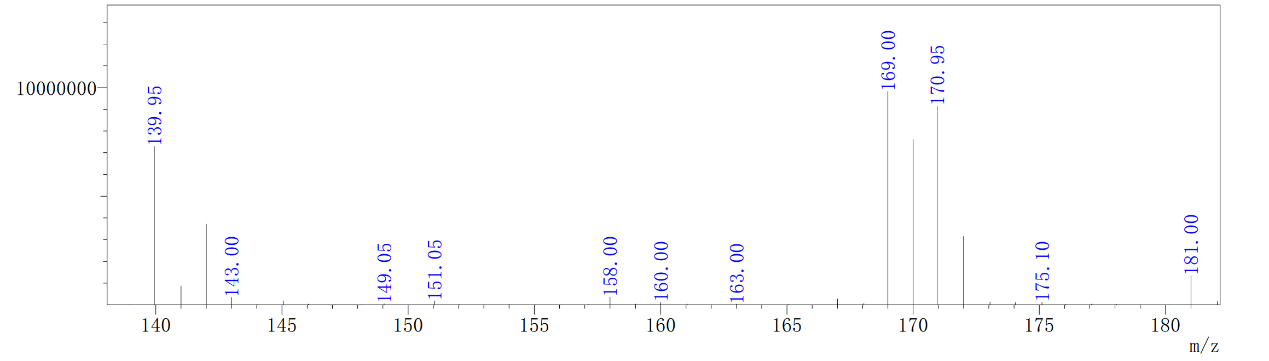
**

**Figure S14.** ^1^H NMR and MS data of compound **4c**.


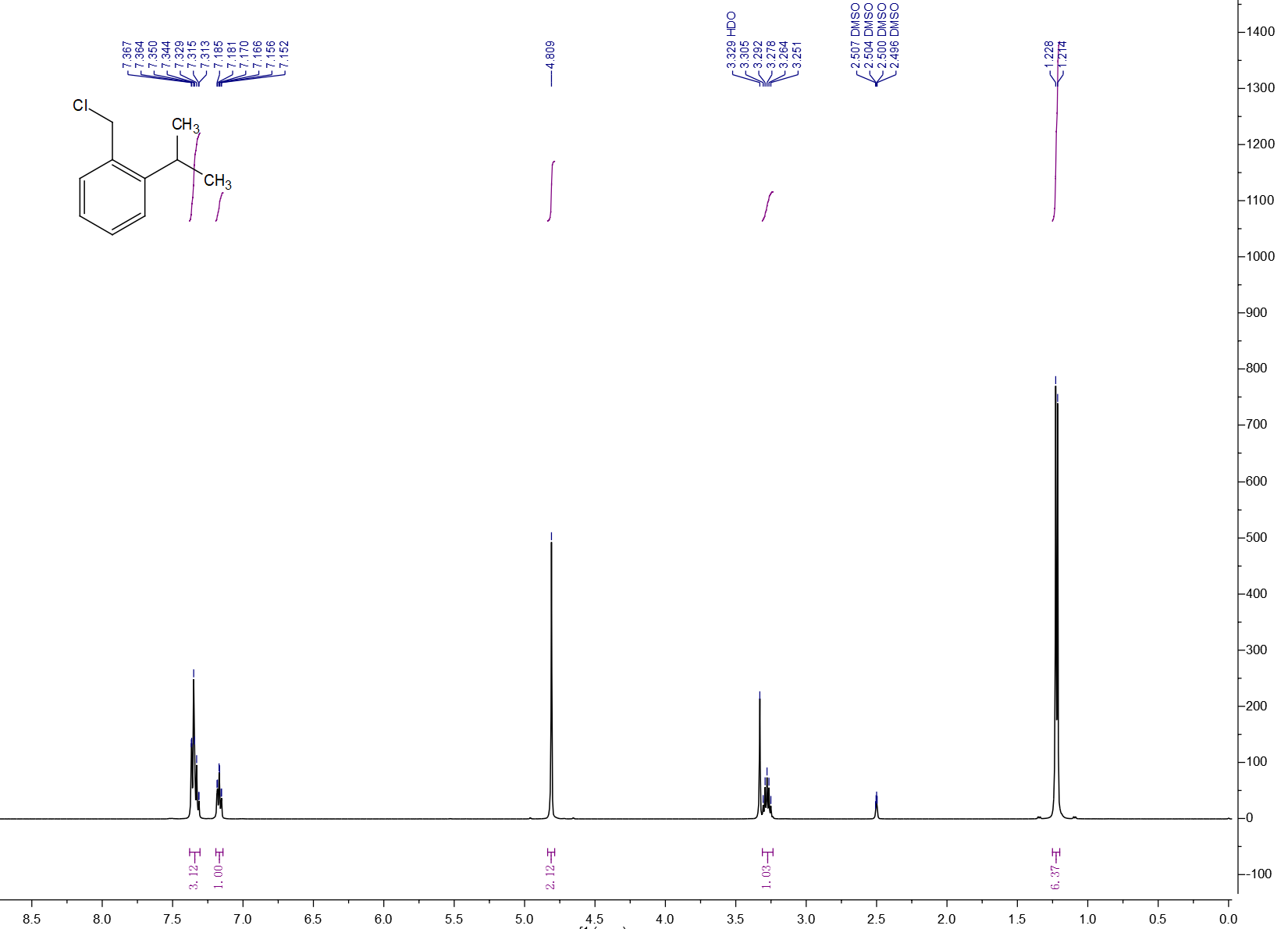


**
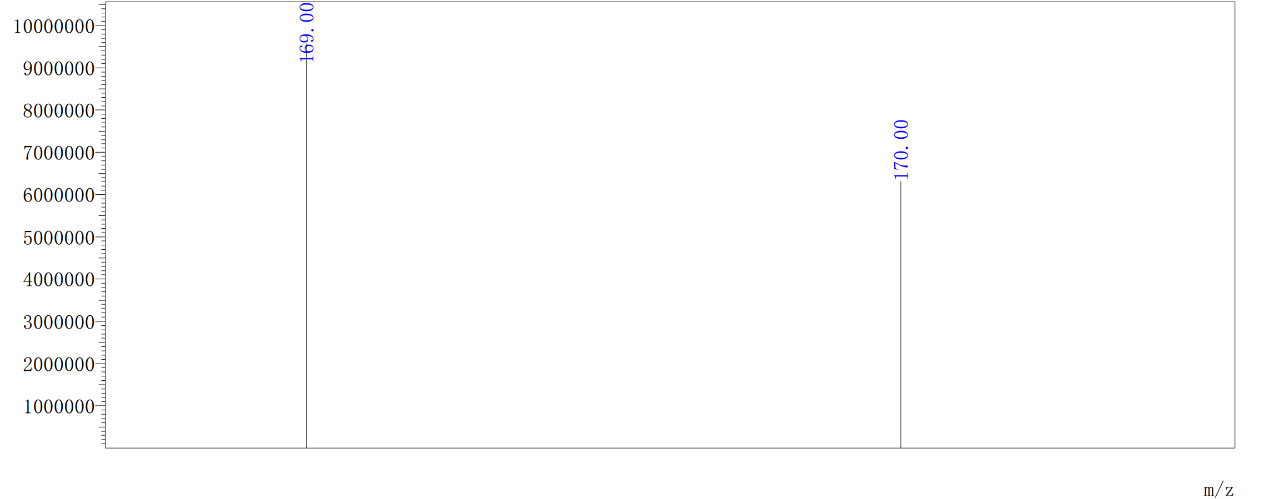
**

**Figure S15.** ^1^H NMR and MS data of compound **4d**.


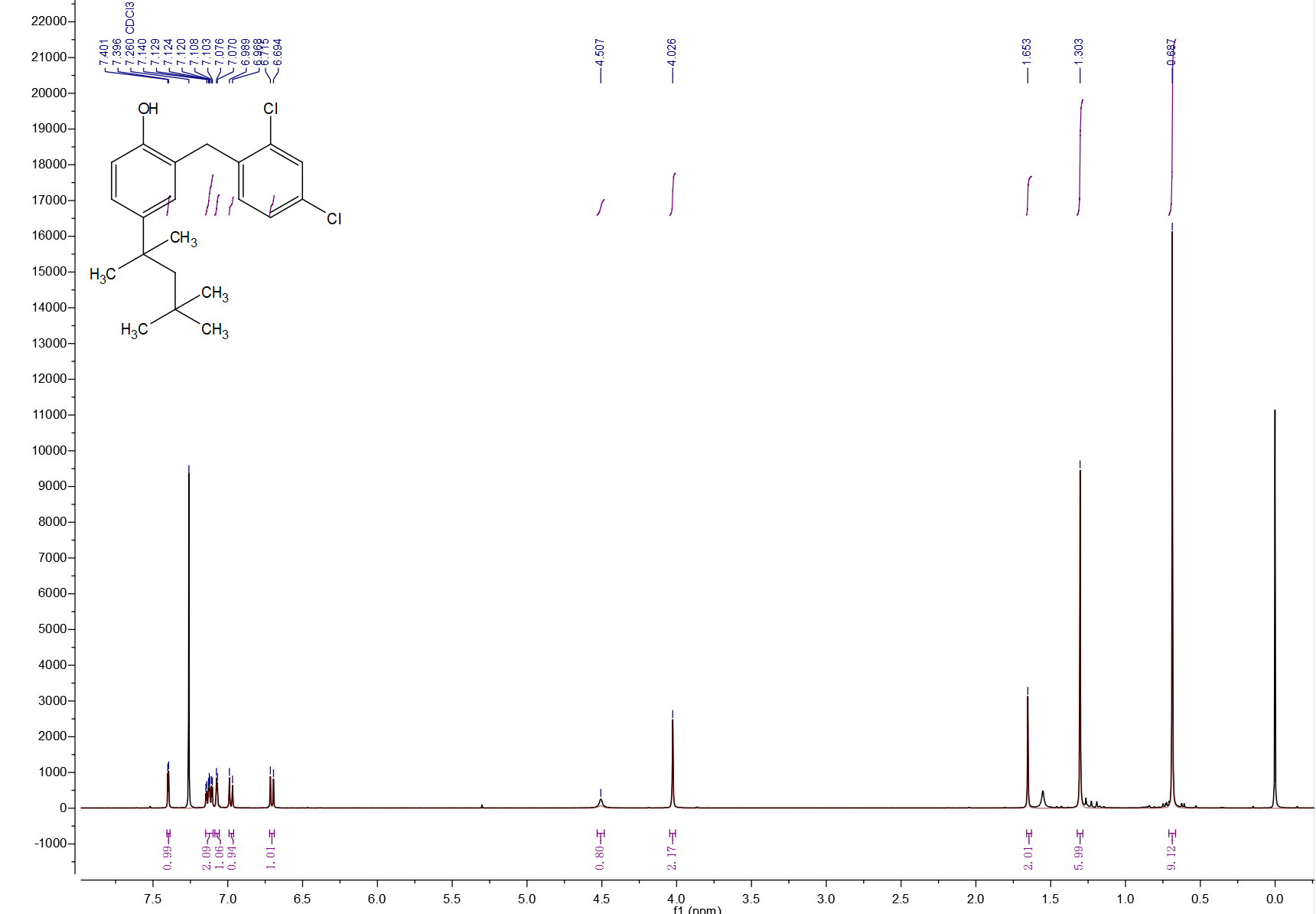


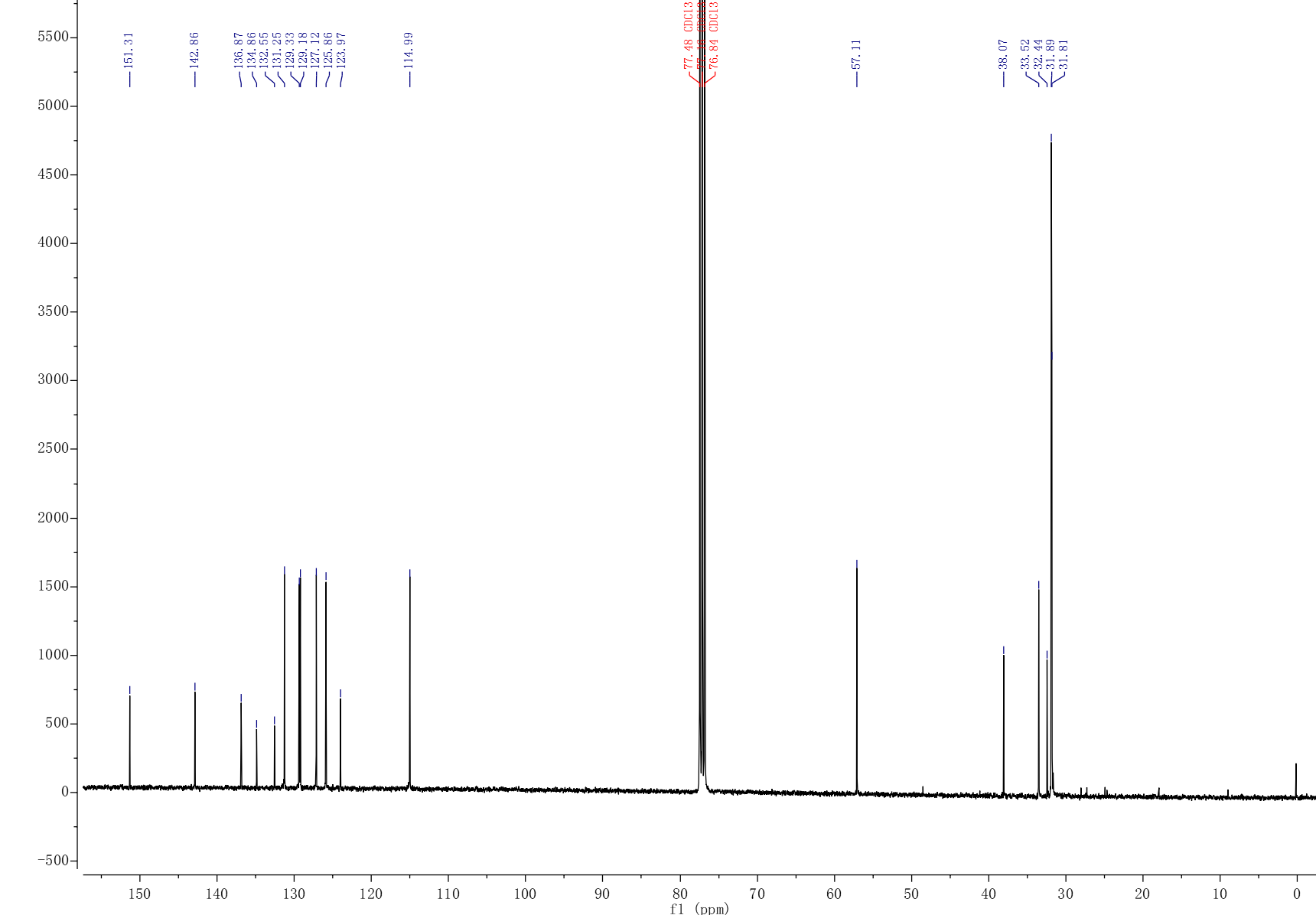


**
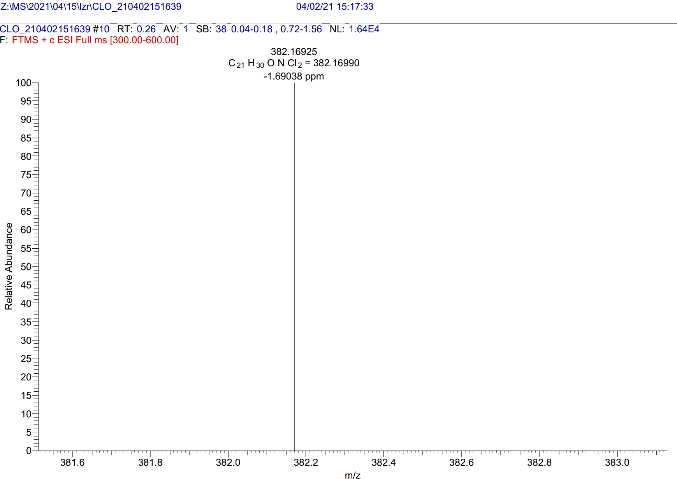
**


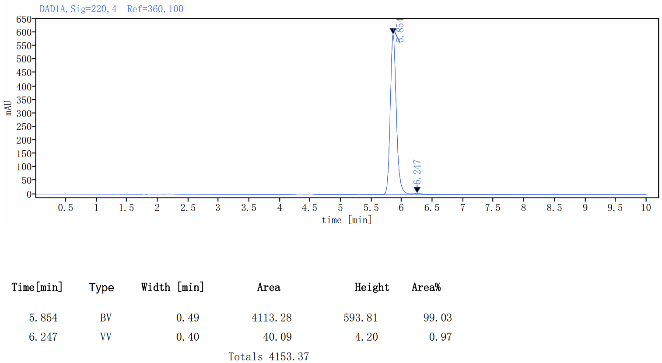


**Figure S16.** ^1^H NMR, ^13^C NMR, HRMS and HPLC data of compound **CFT**.

**
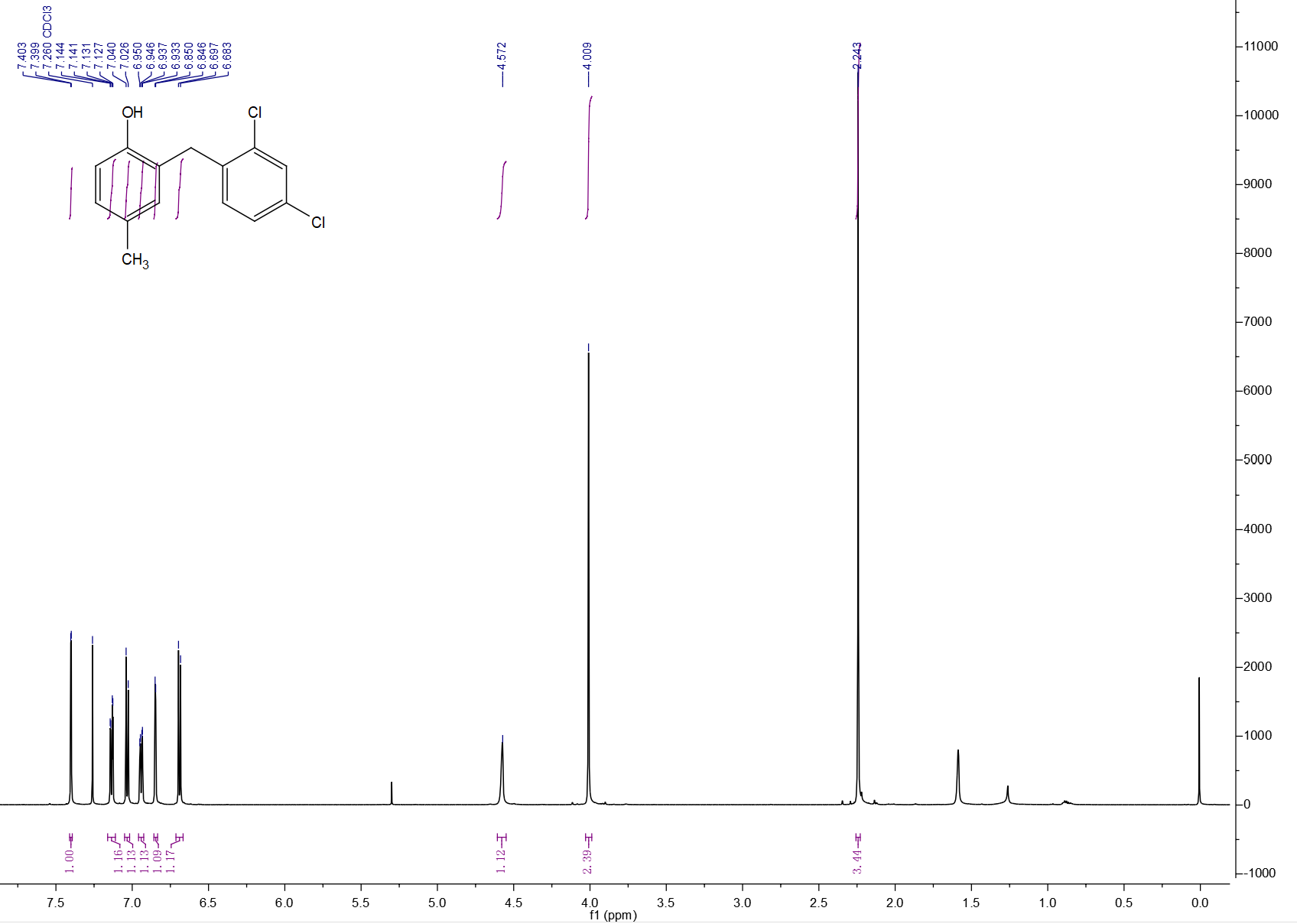
**


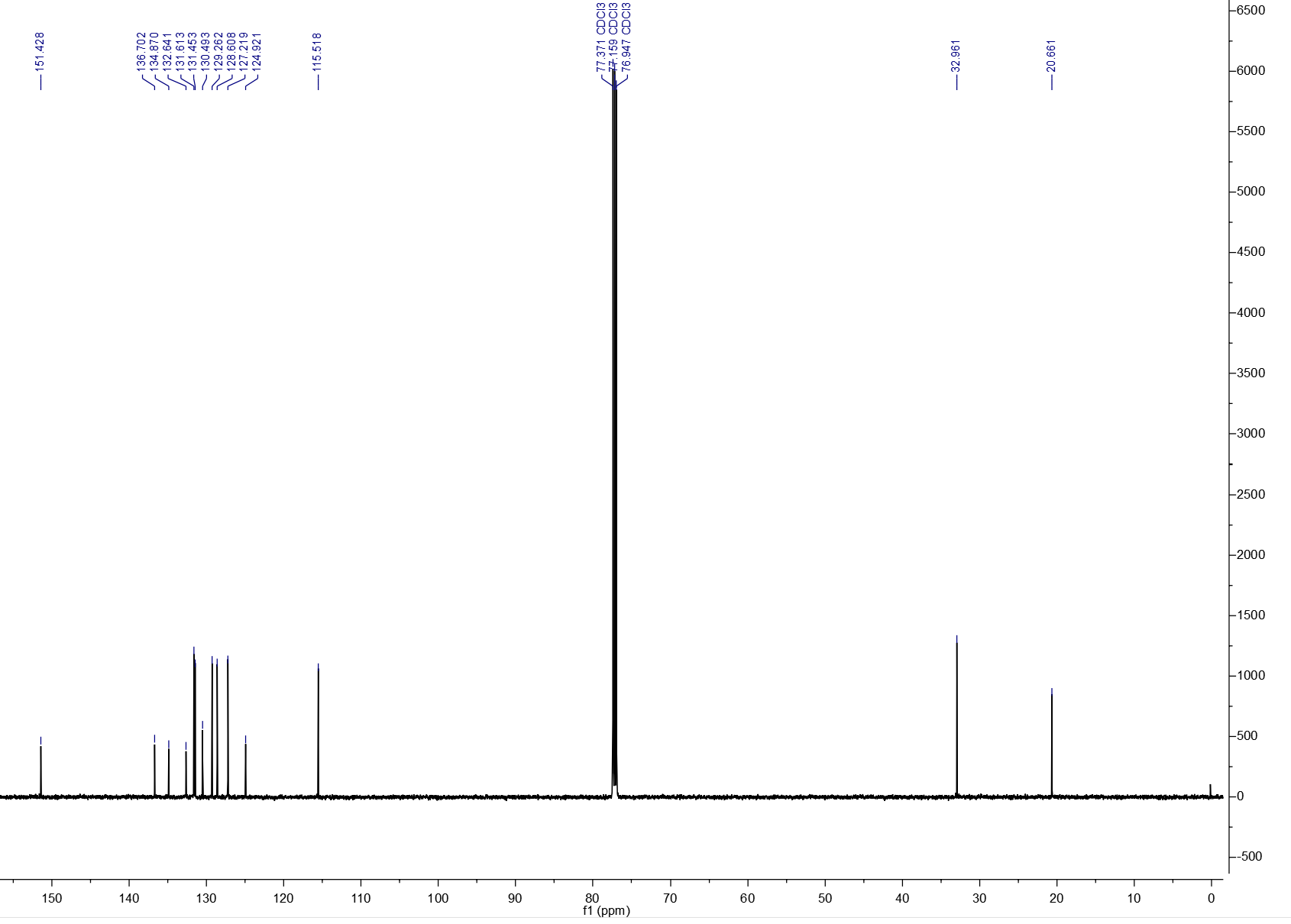


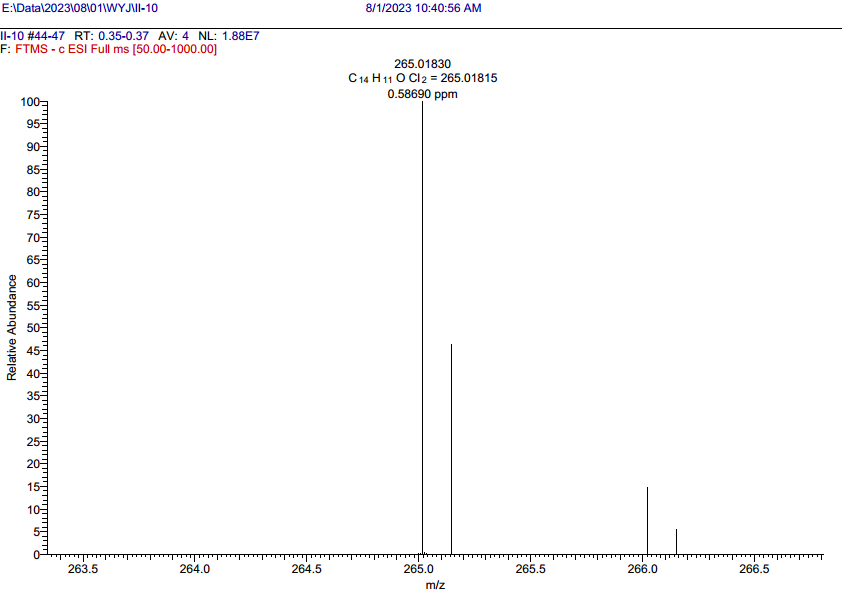


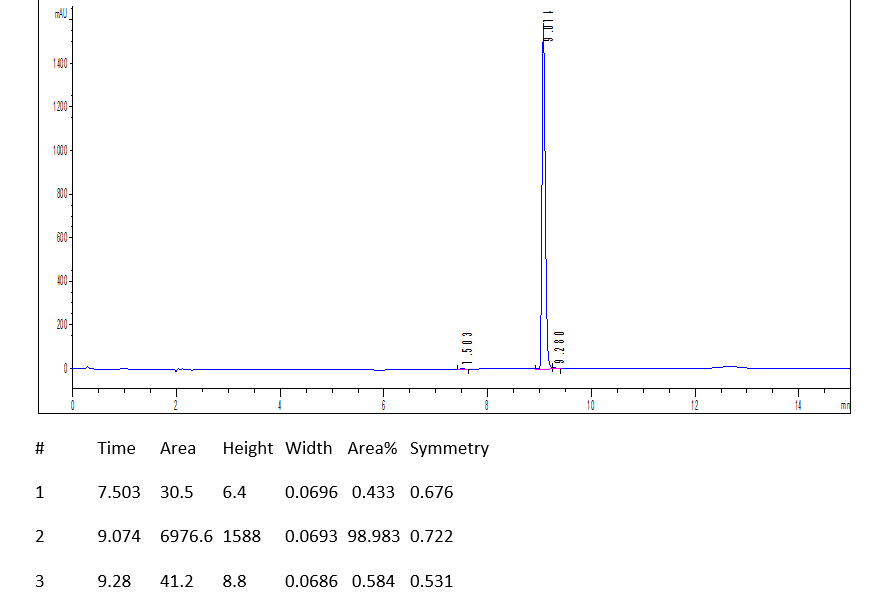


**Figure S17.** ^1^H NMR, ^13^C NMR, HRMS and HPLC data of compound **A1**.


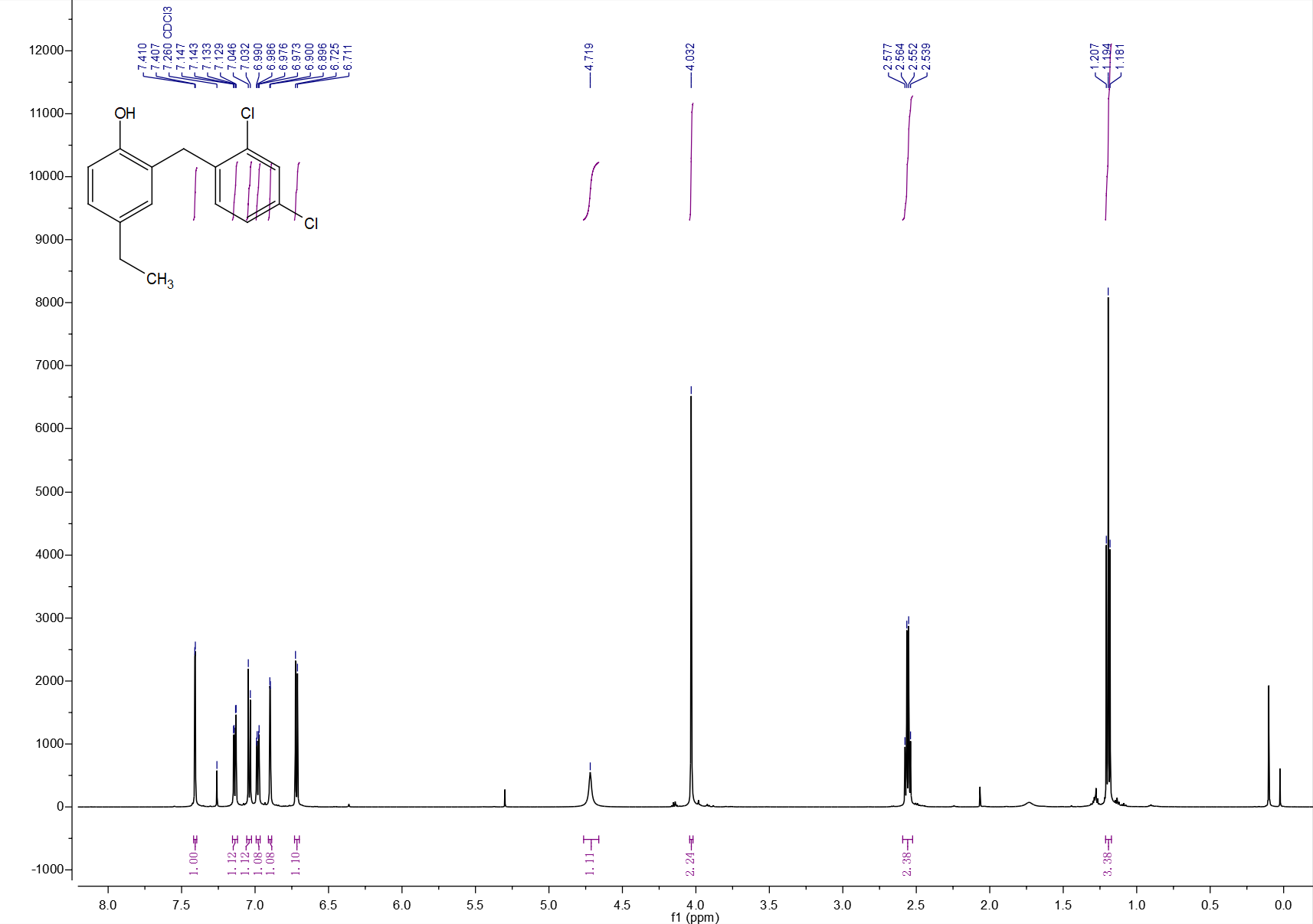


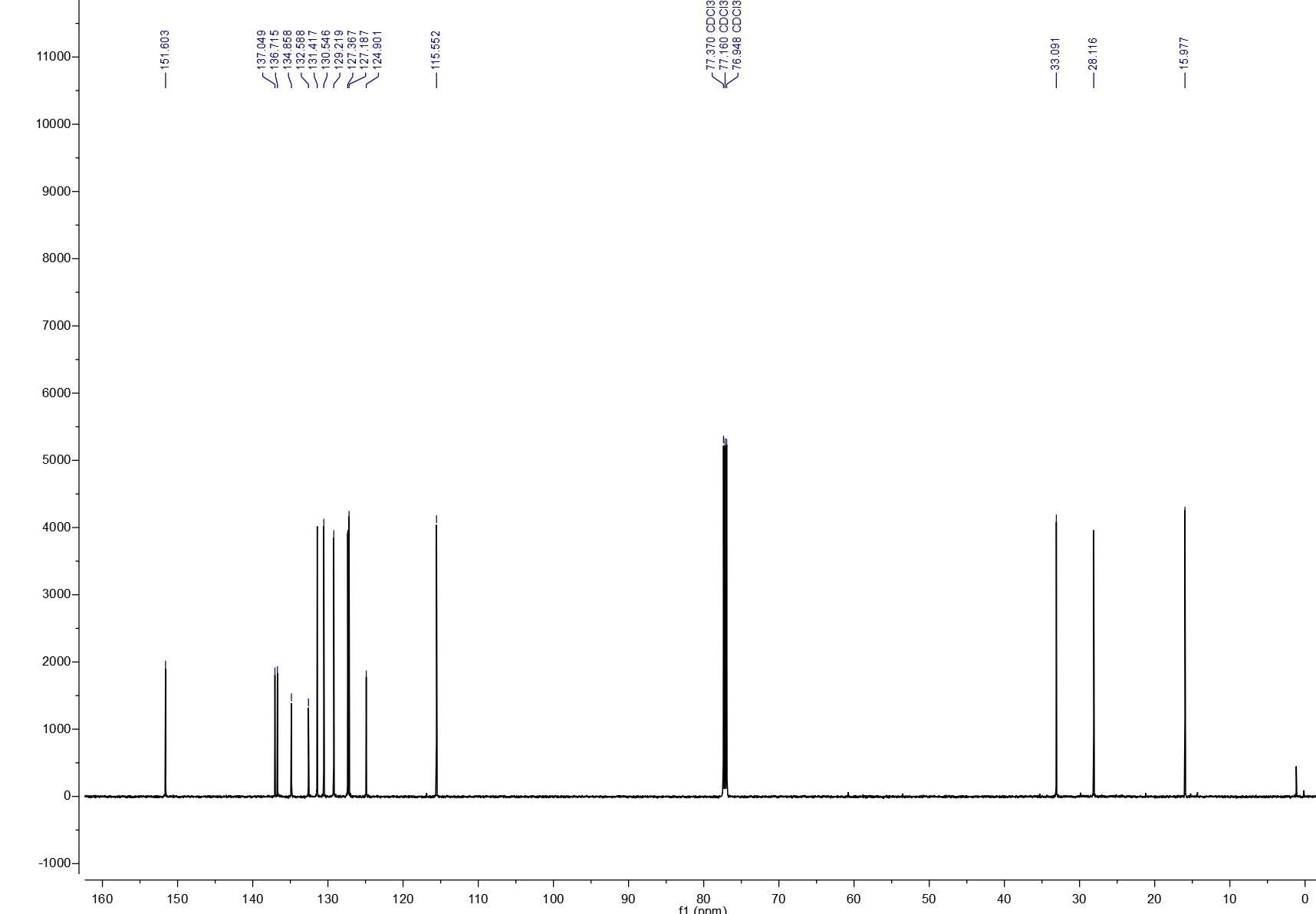


**
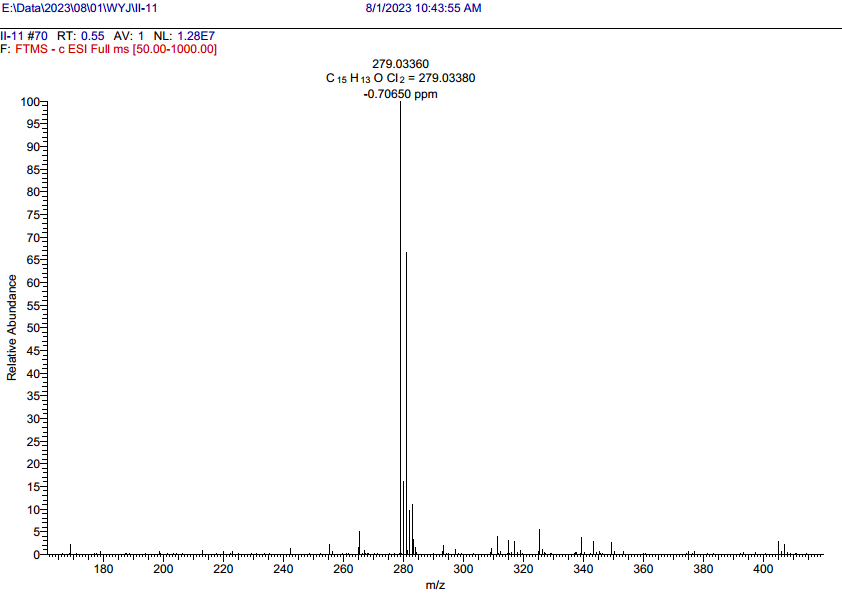
**

**
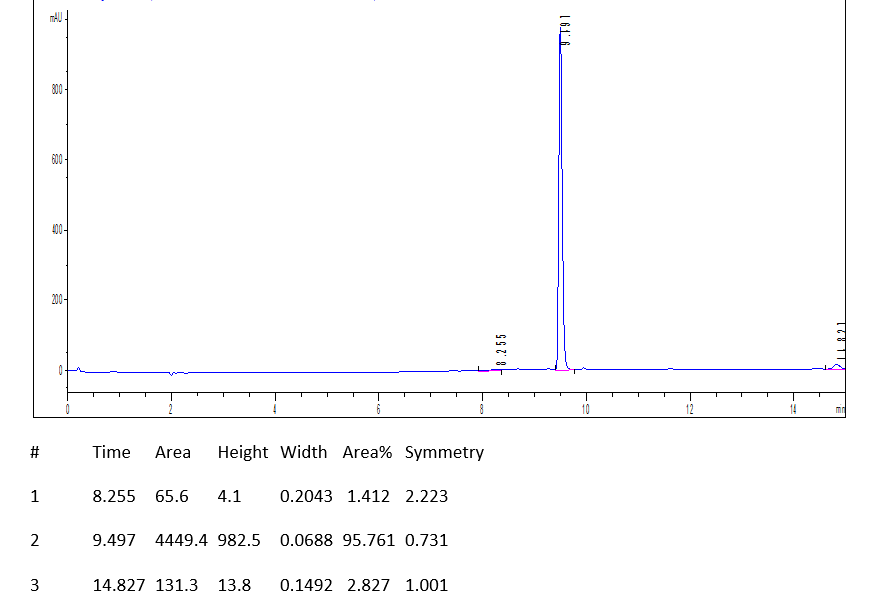
**

**Figure S18.** ^1^H NMR, ^13^C NMR, HRMS and HPLC data of compound **A2**.


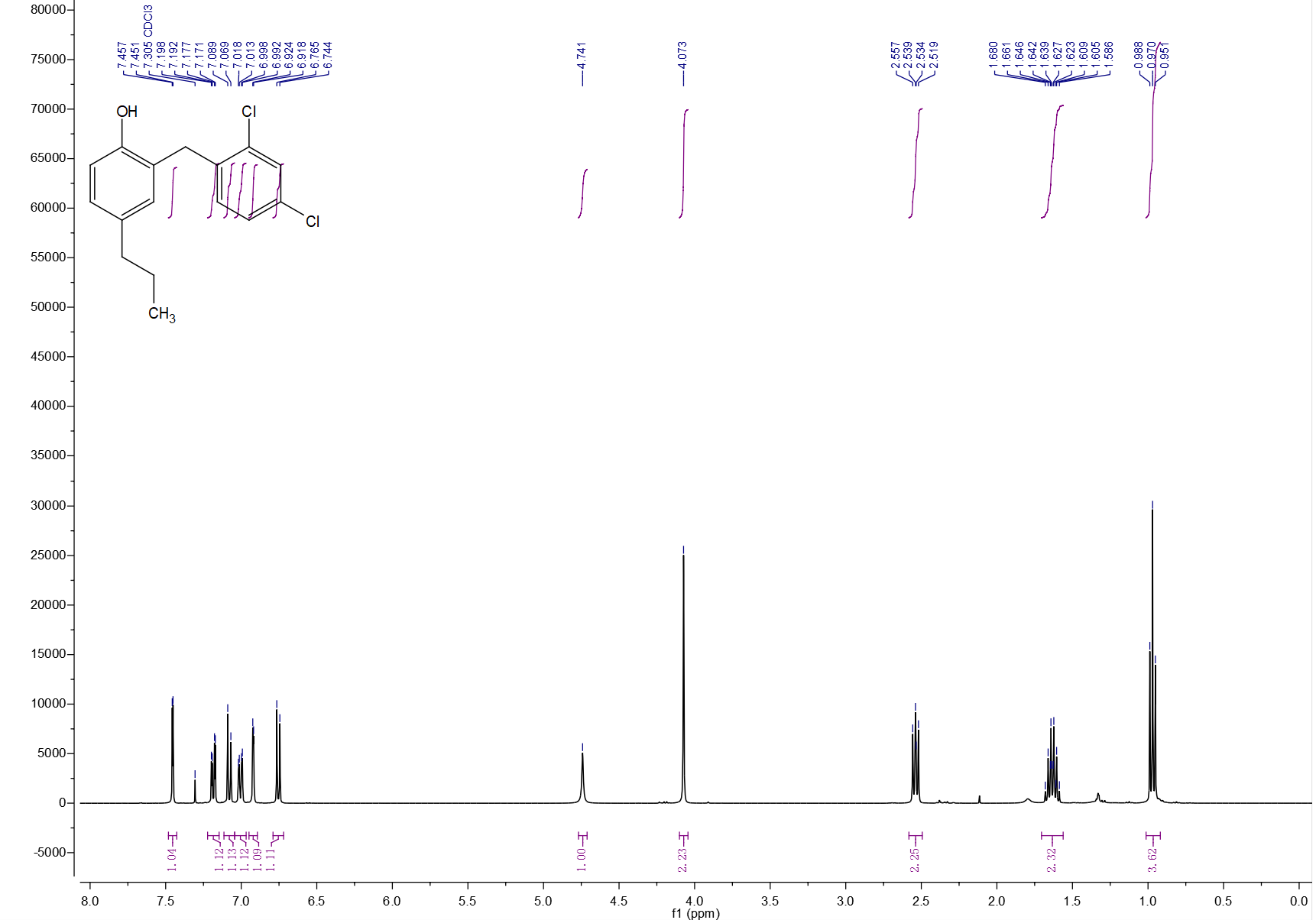


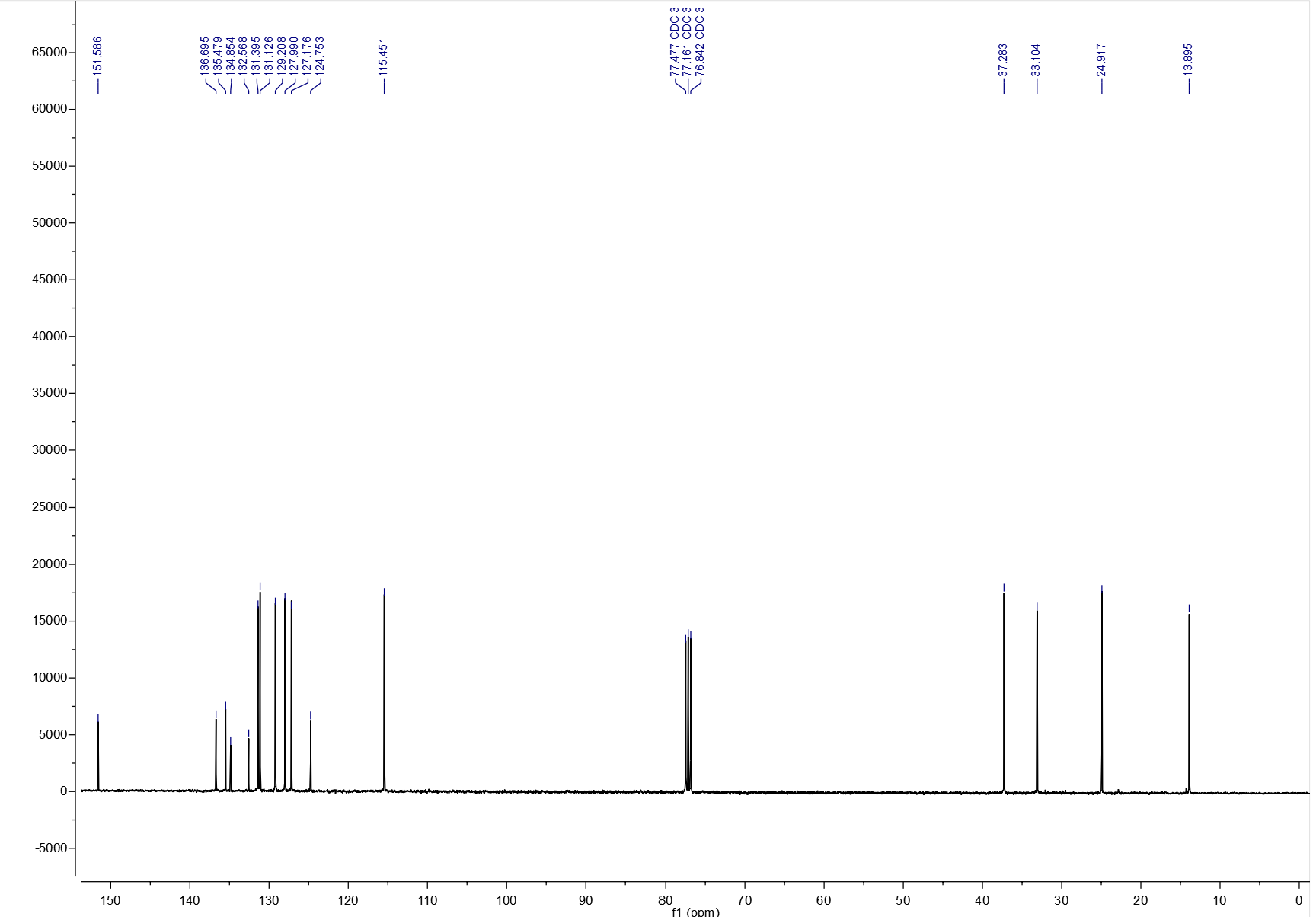


**
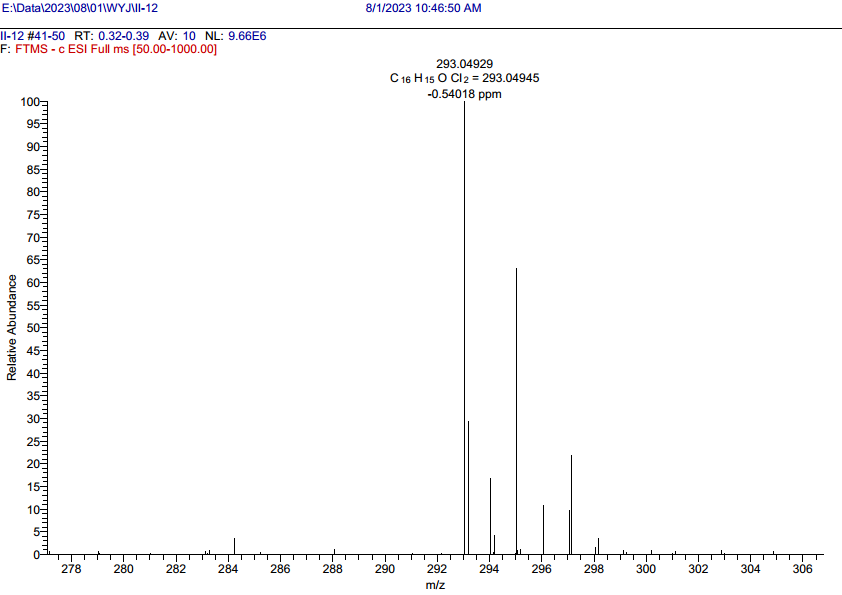
**

**
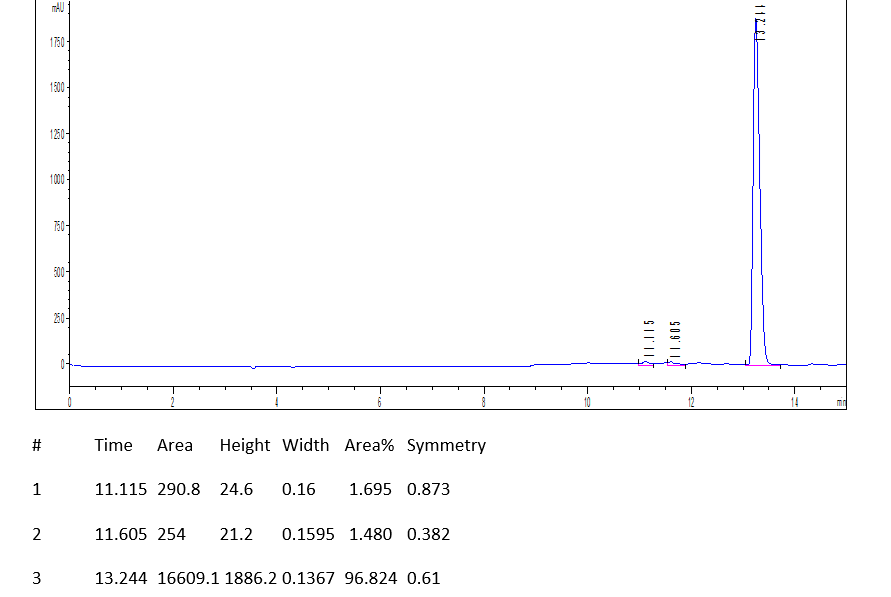
**

**Figure S19.** ^1^H NMR, ^13^C NMR, HRMS and HPLC data of compound **A3**.


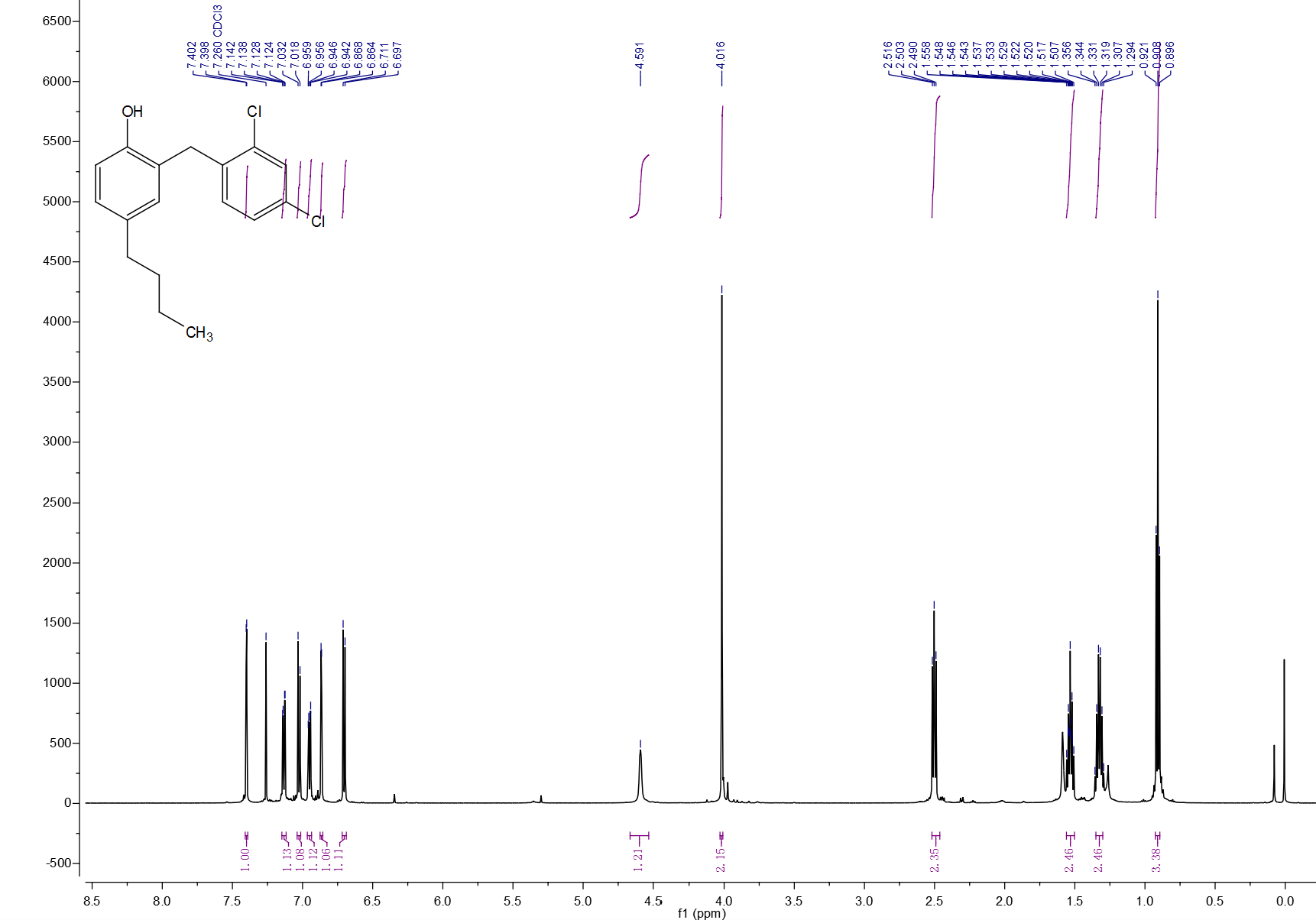


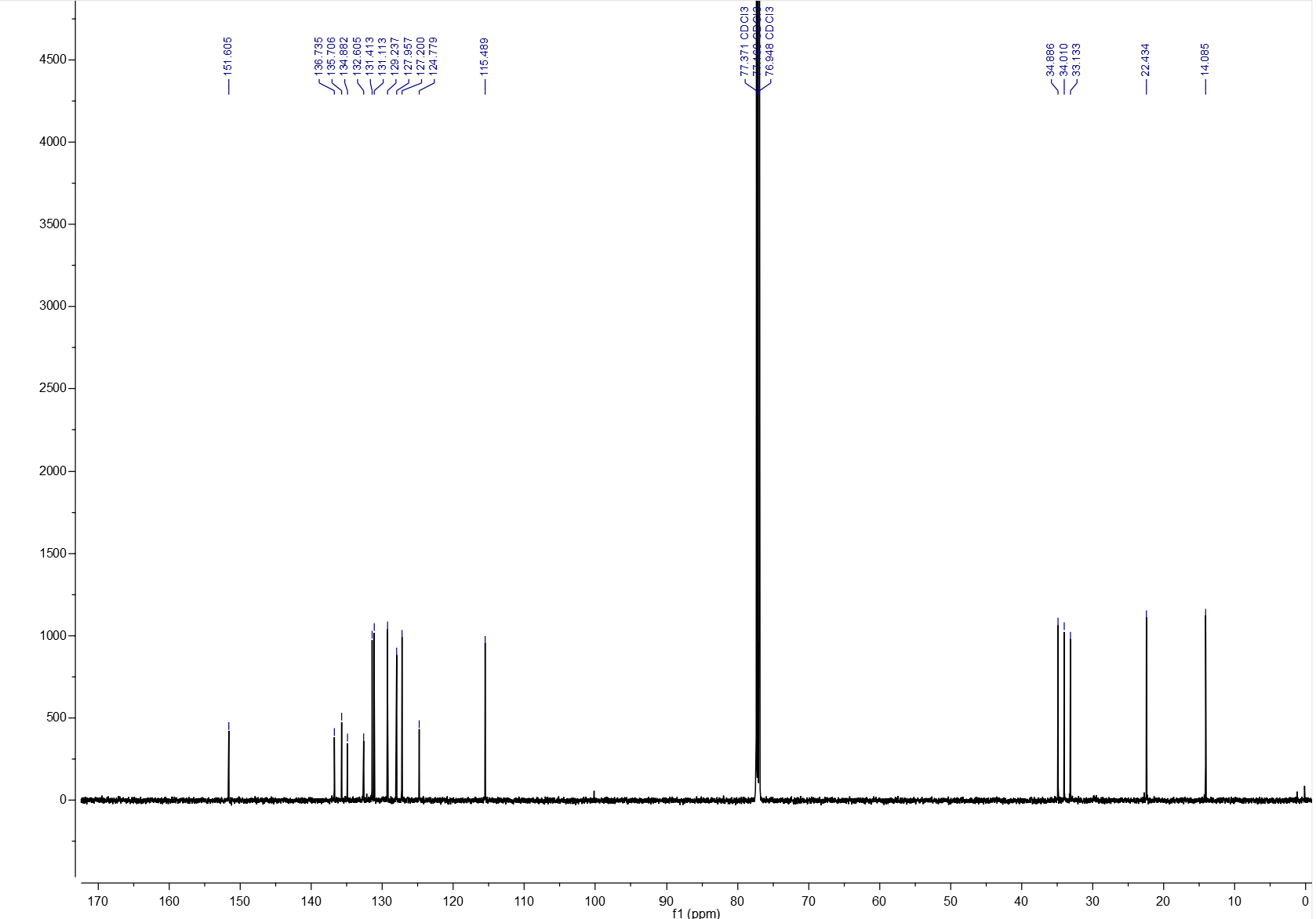


**
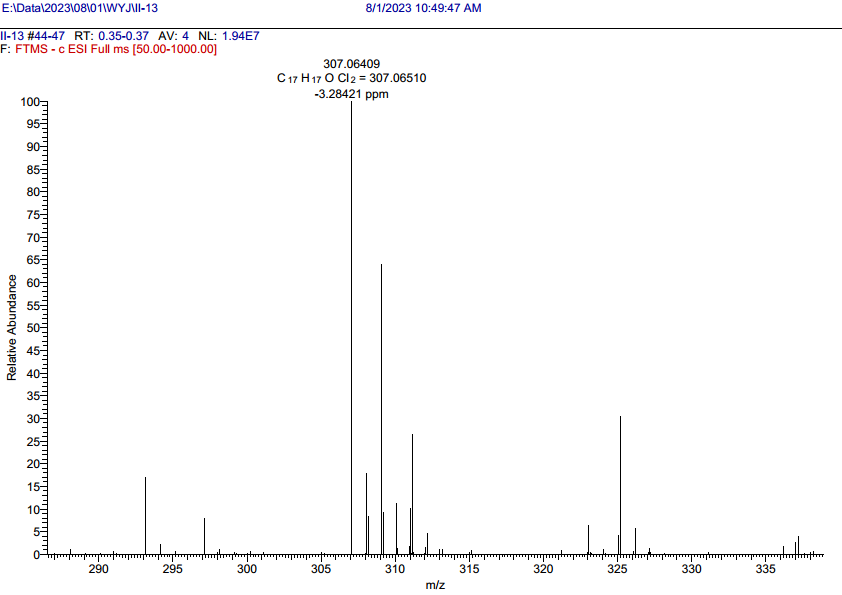
**

**
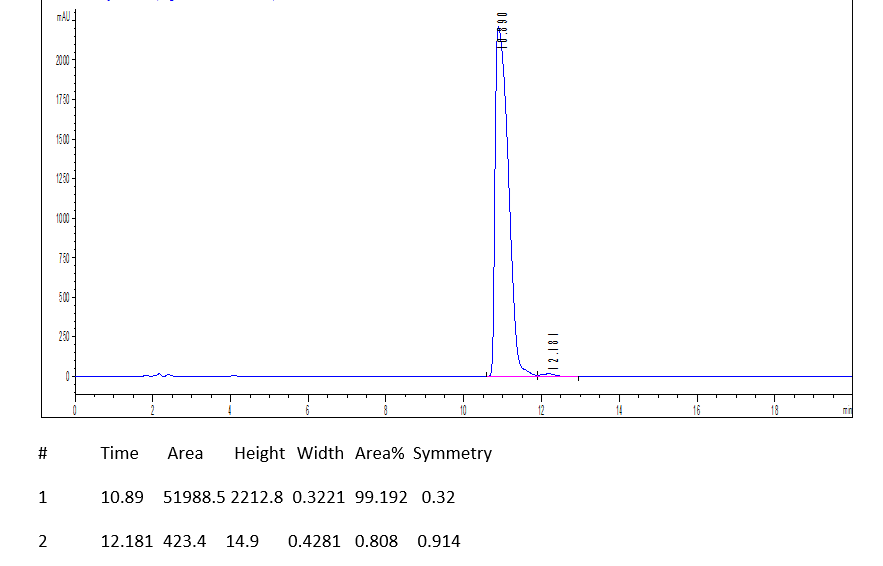
**

**Figure S20.** ^1^H NMR, ^13^C NMR, HRMS and HPLC data of compound **A4**.


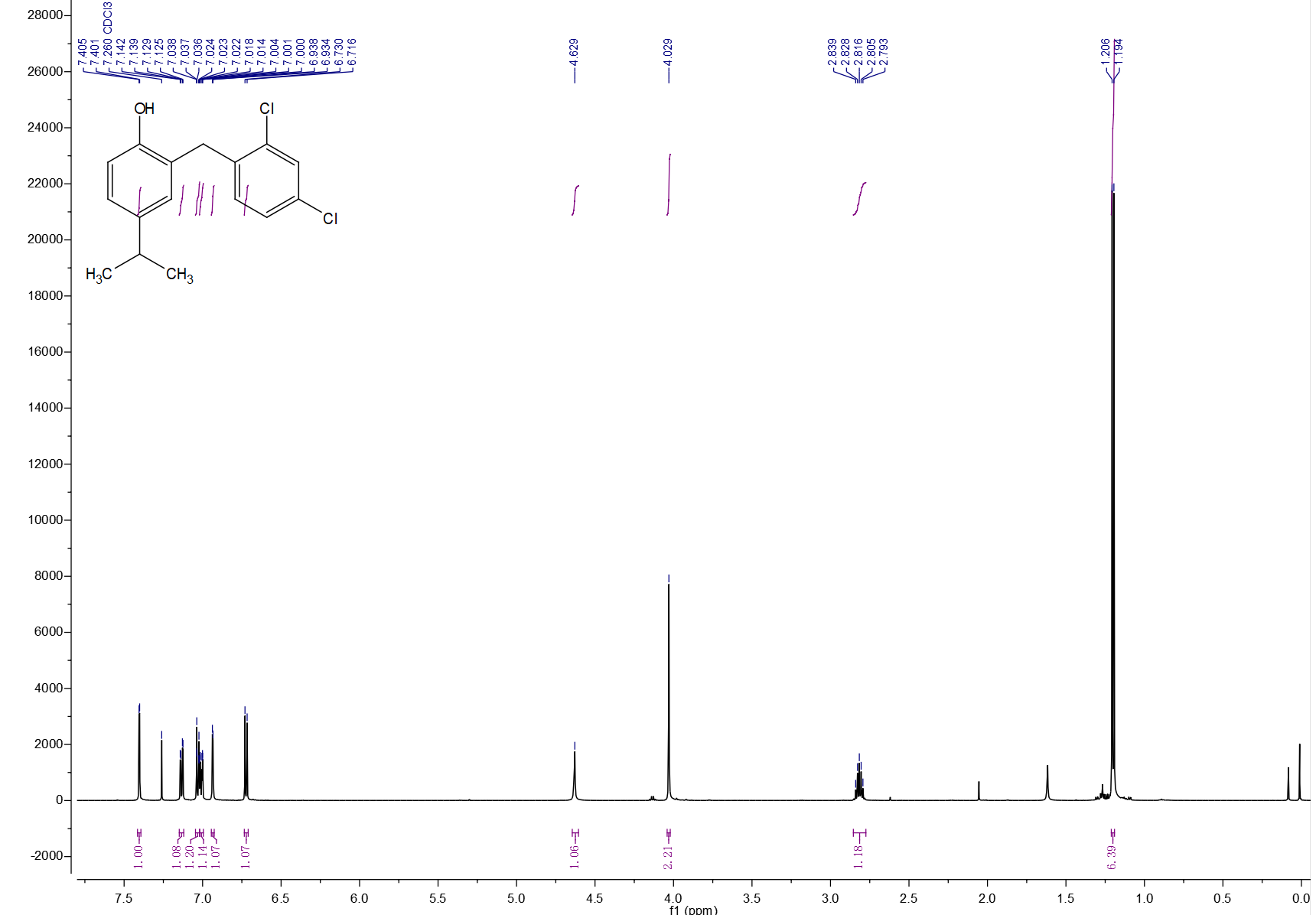


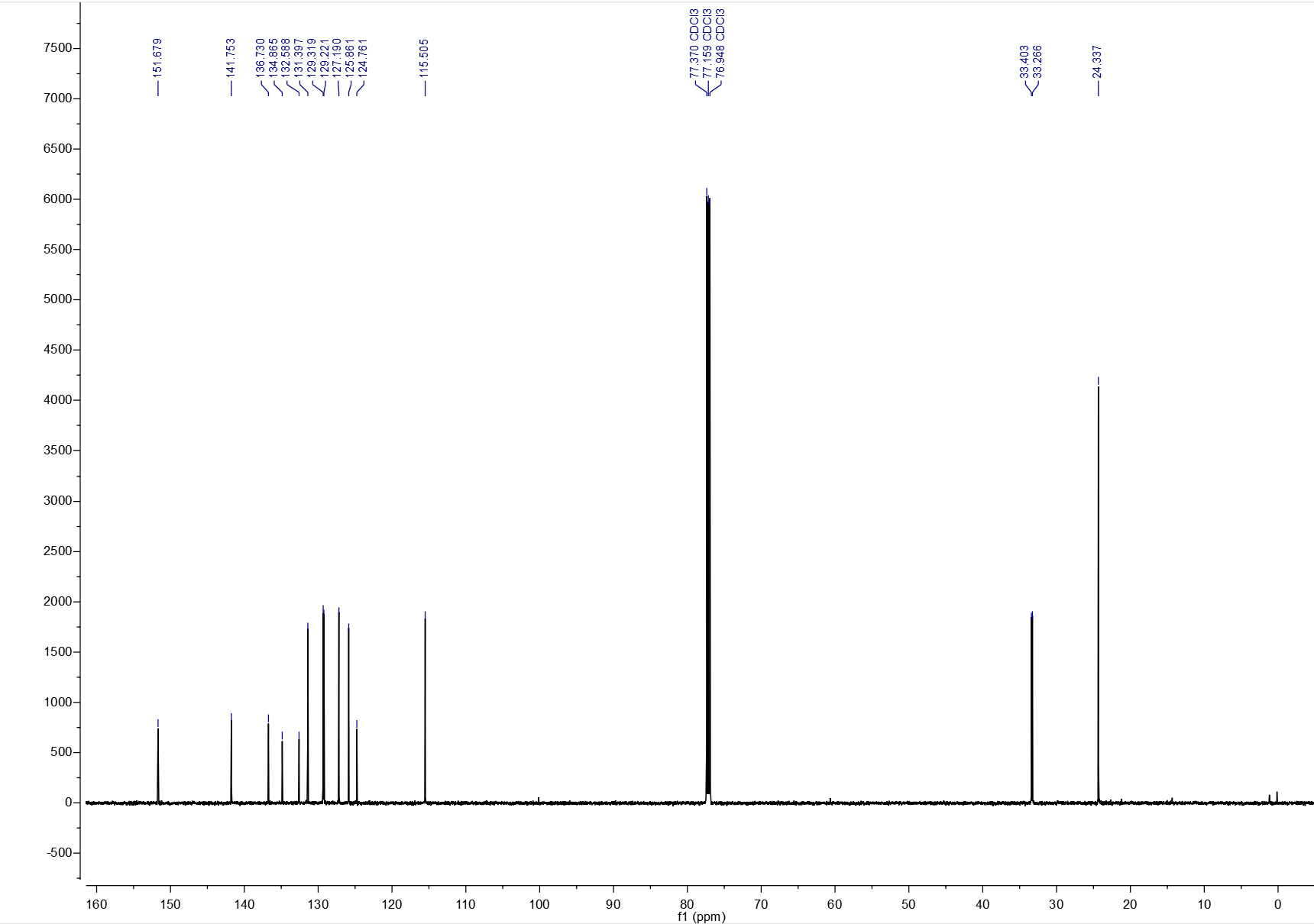


**
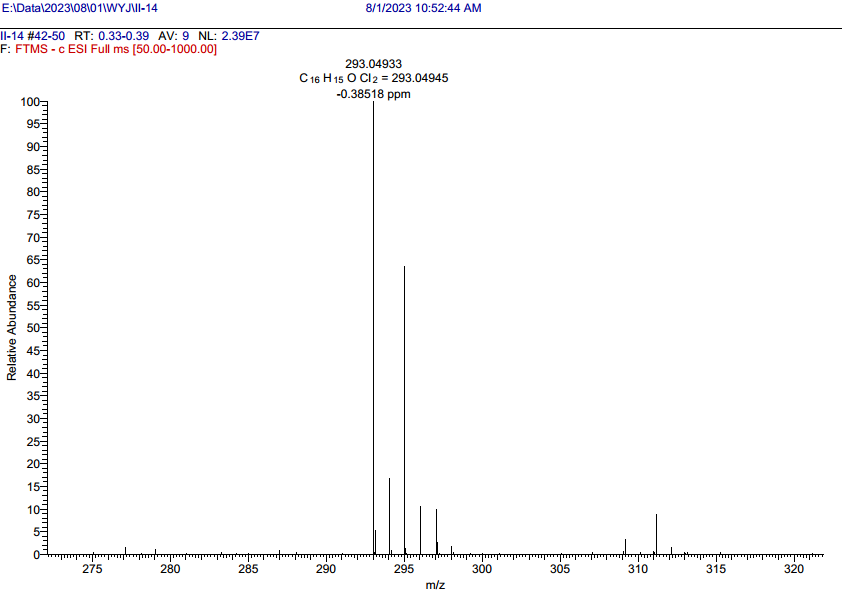
**

**
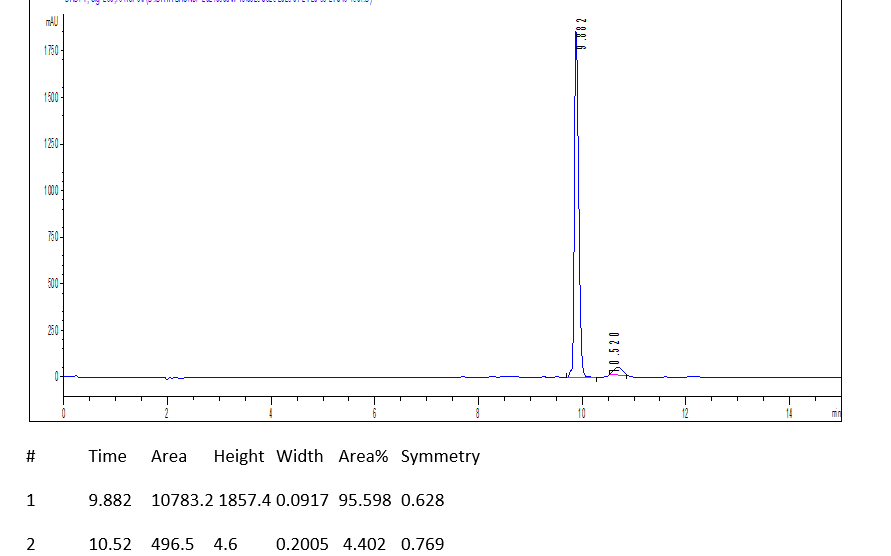
**

**Figure S21.** ^1^H NMR, ^13^C NMR, HRMS and HPLC data of compound **A5**.


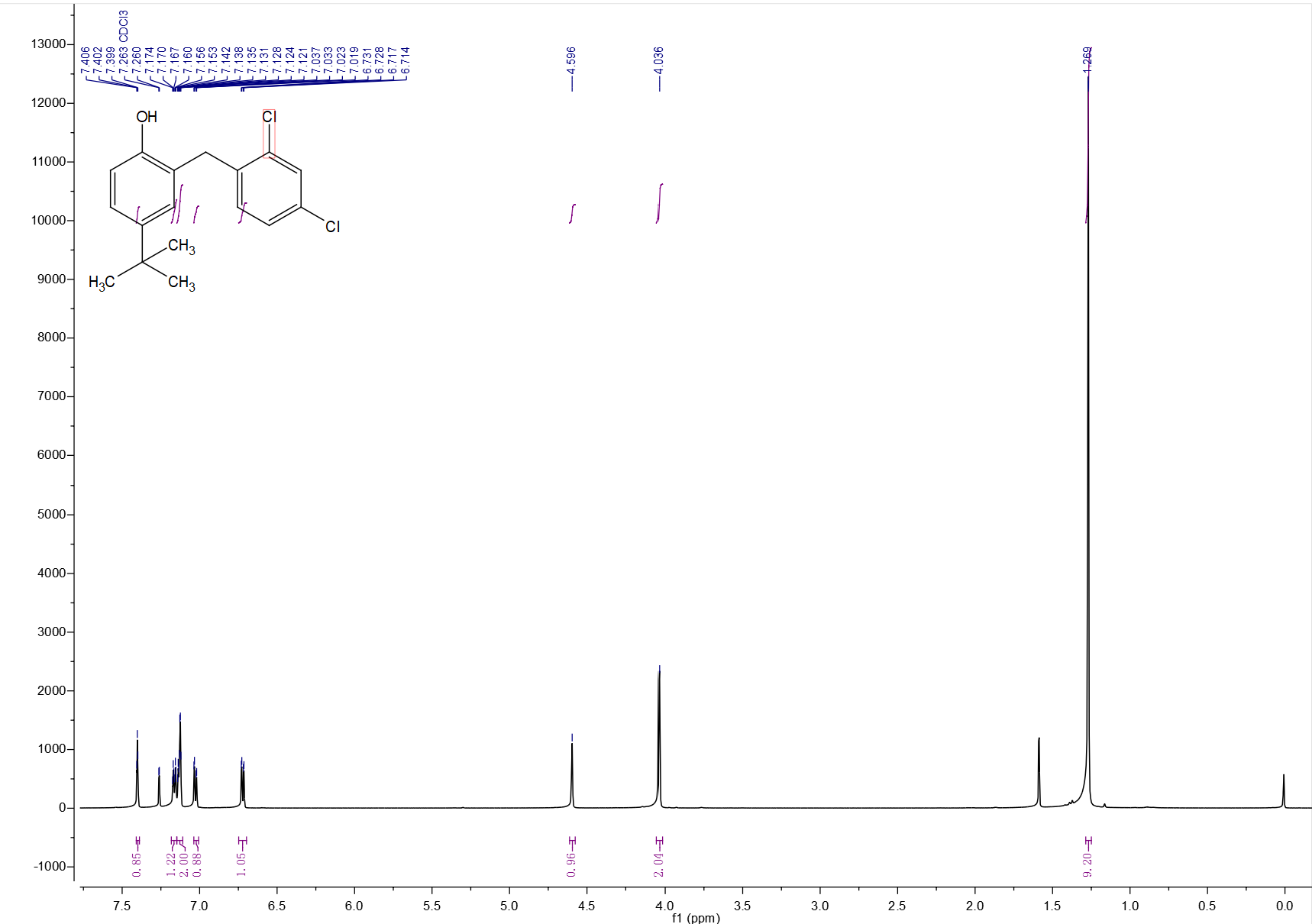


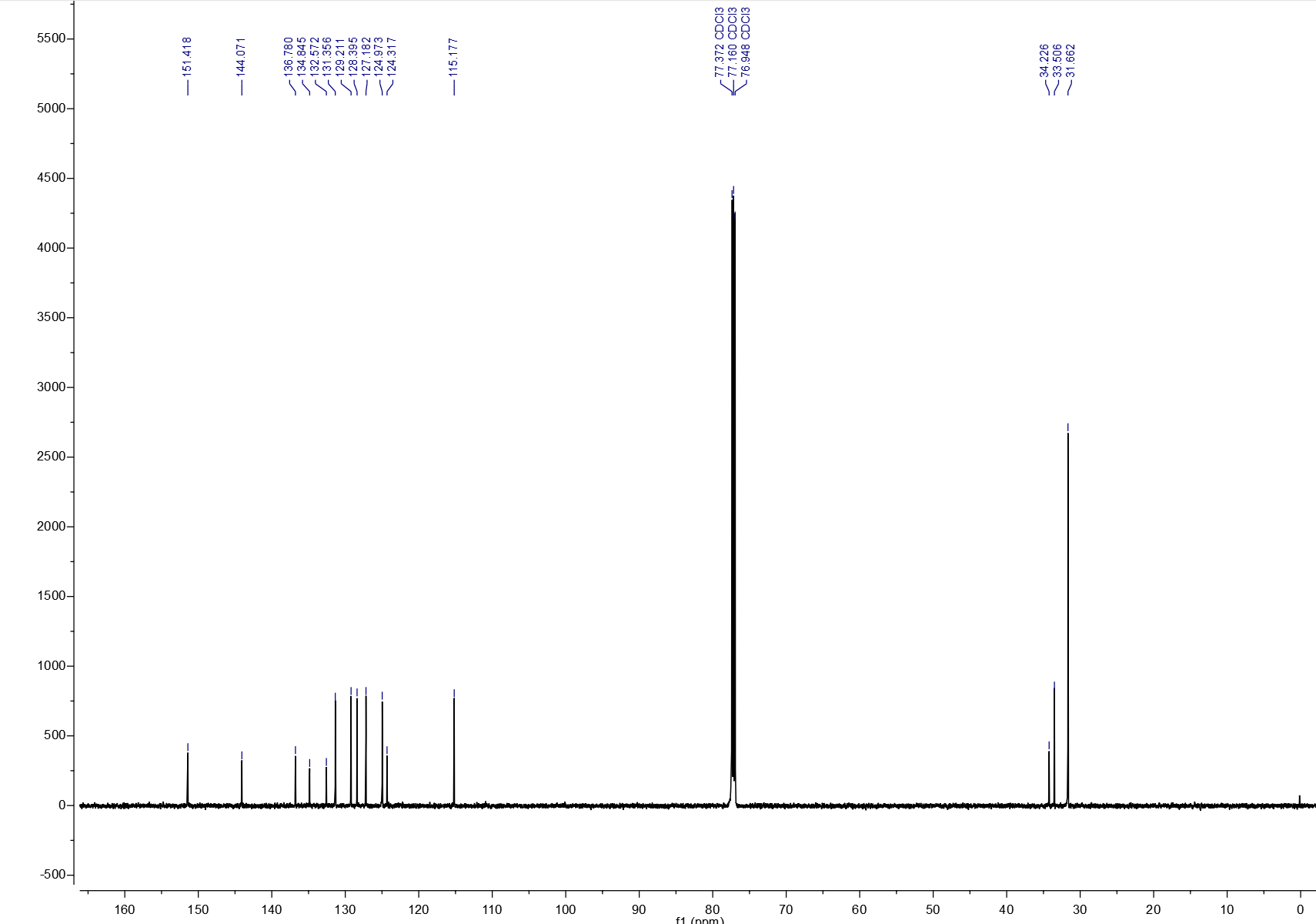


**
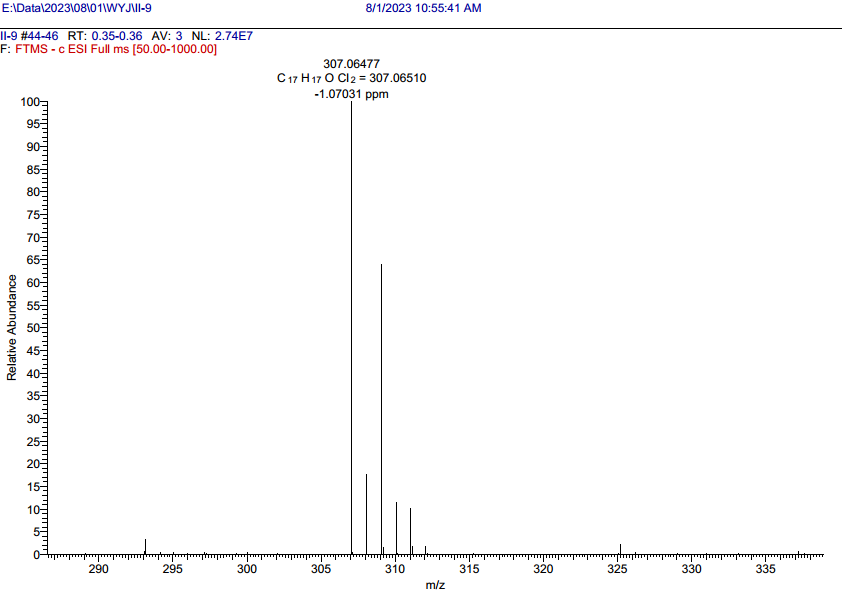
**

**
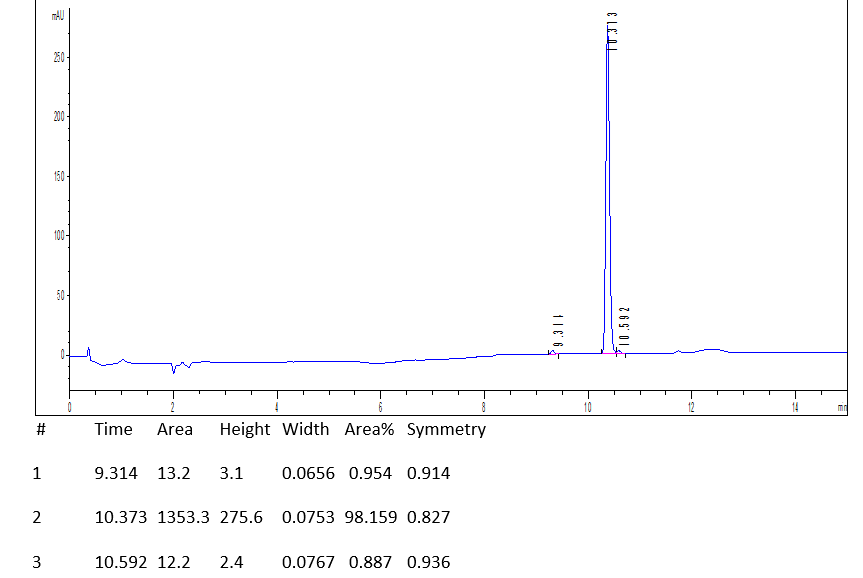
**

**Figure S22.** ^1^H NMR, ^13^C NMR, HRMS and HPLC data of compound **A6**.


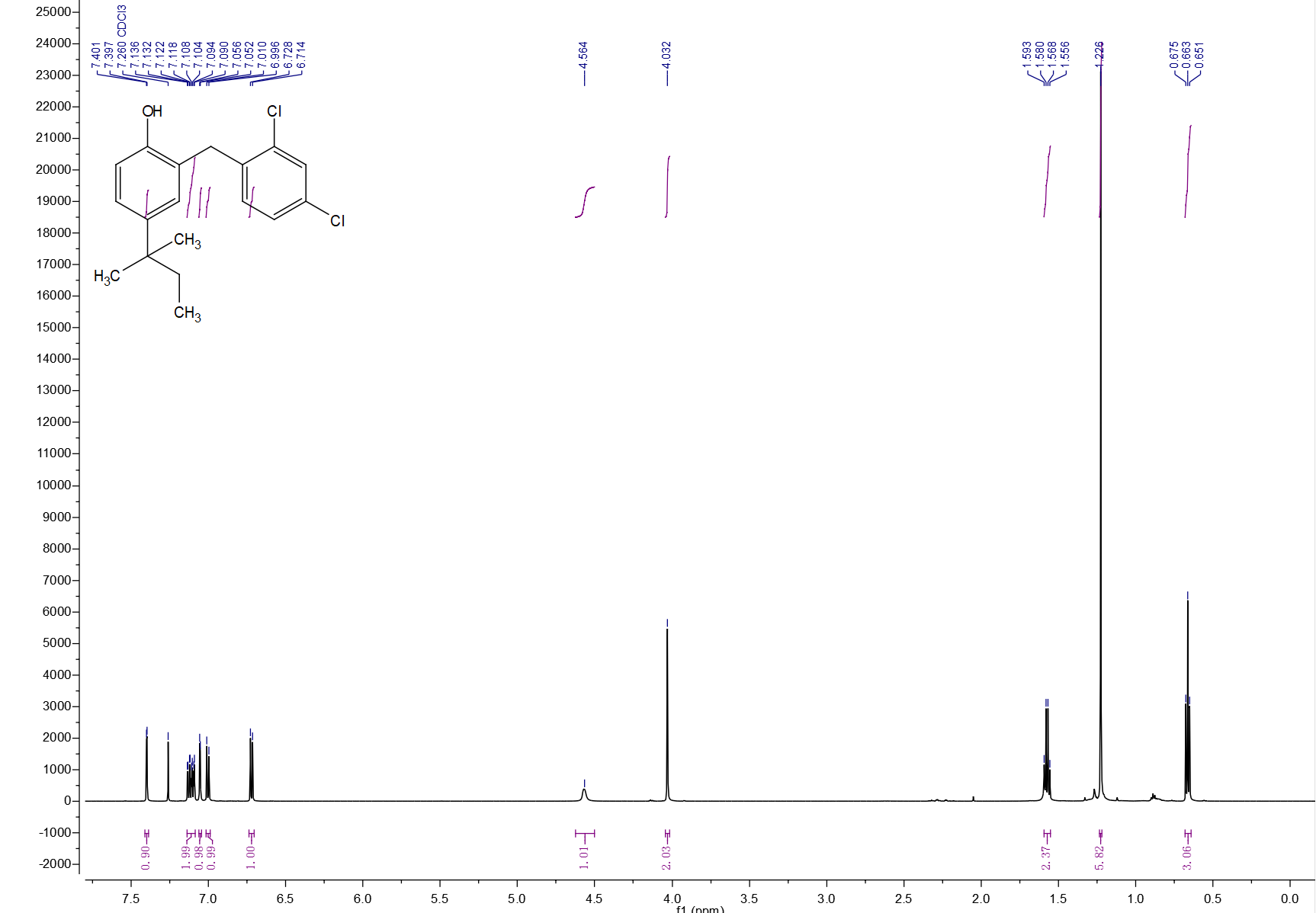


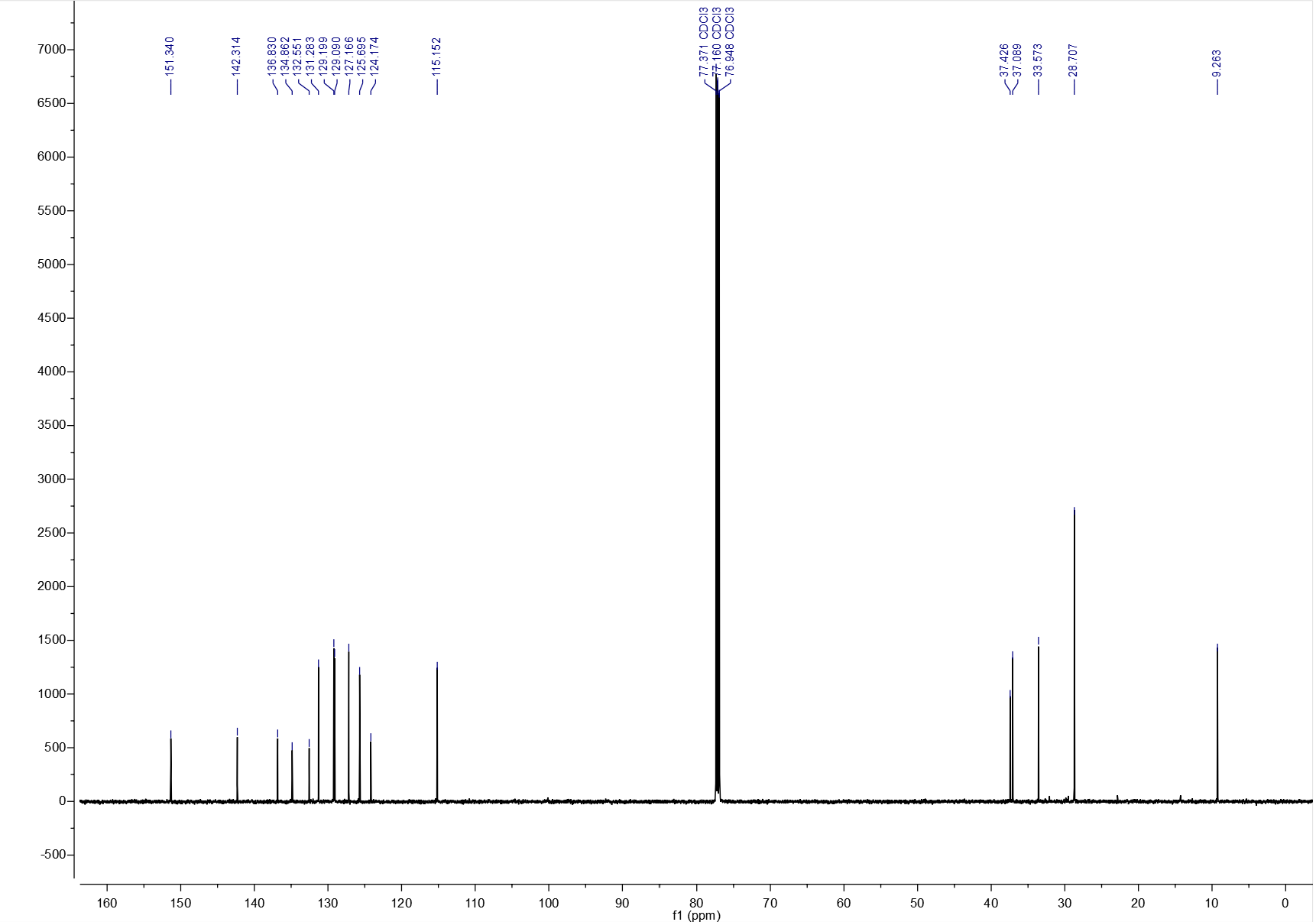


**
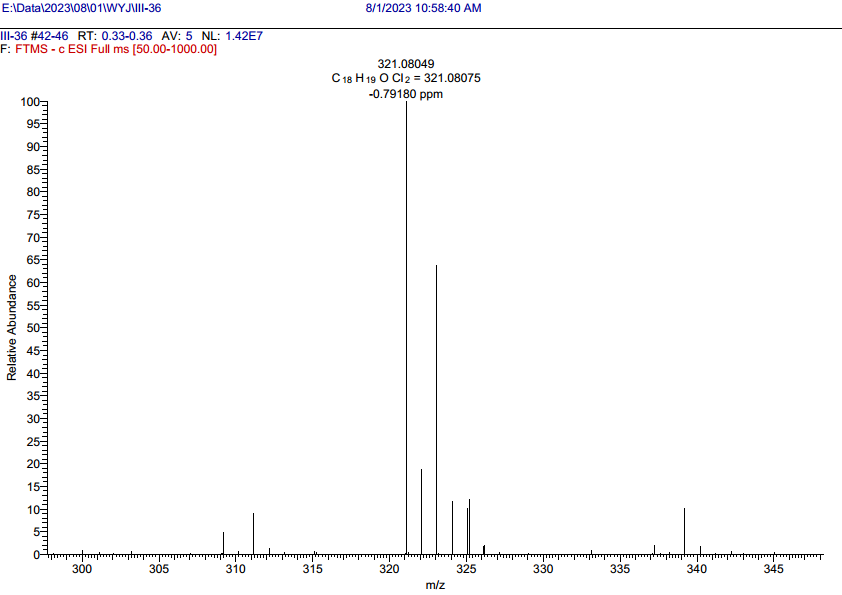
**

**
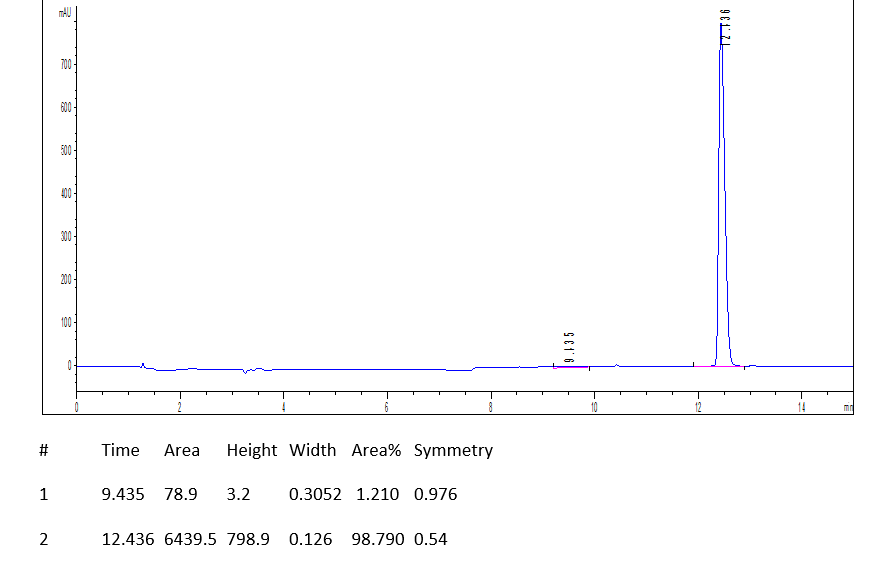
**

**Figure S23.** ^1^H NMR, ^13^C NMR, HRMS and HPLC data of compound **A7**.


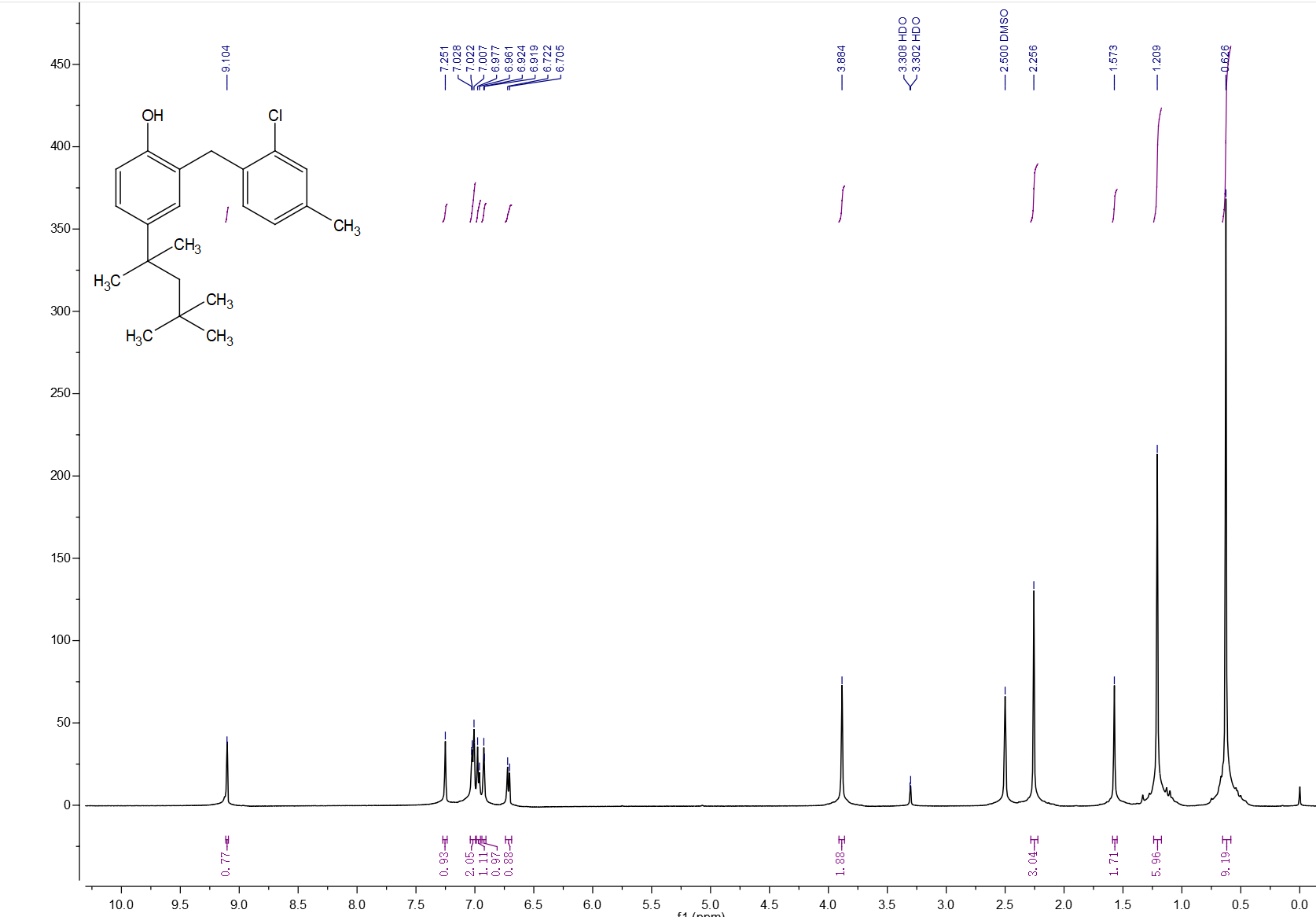


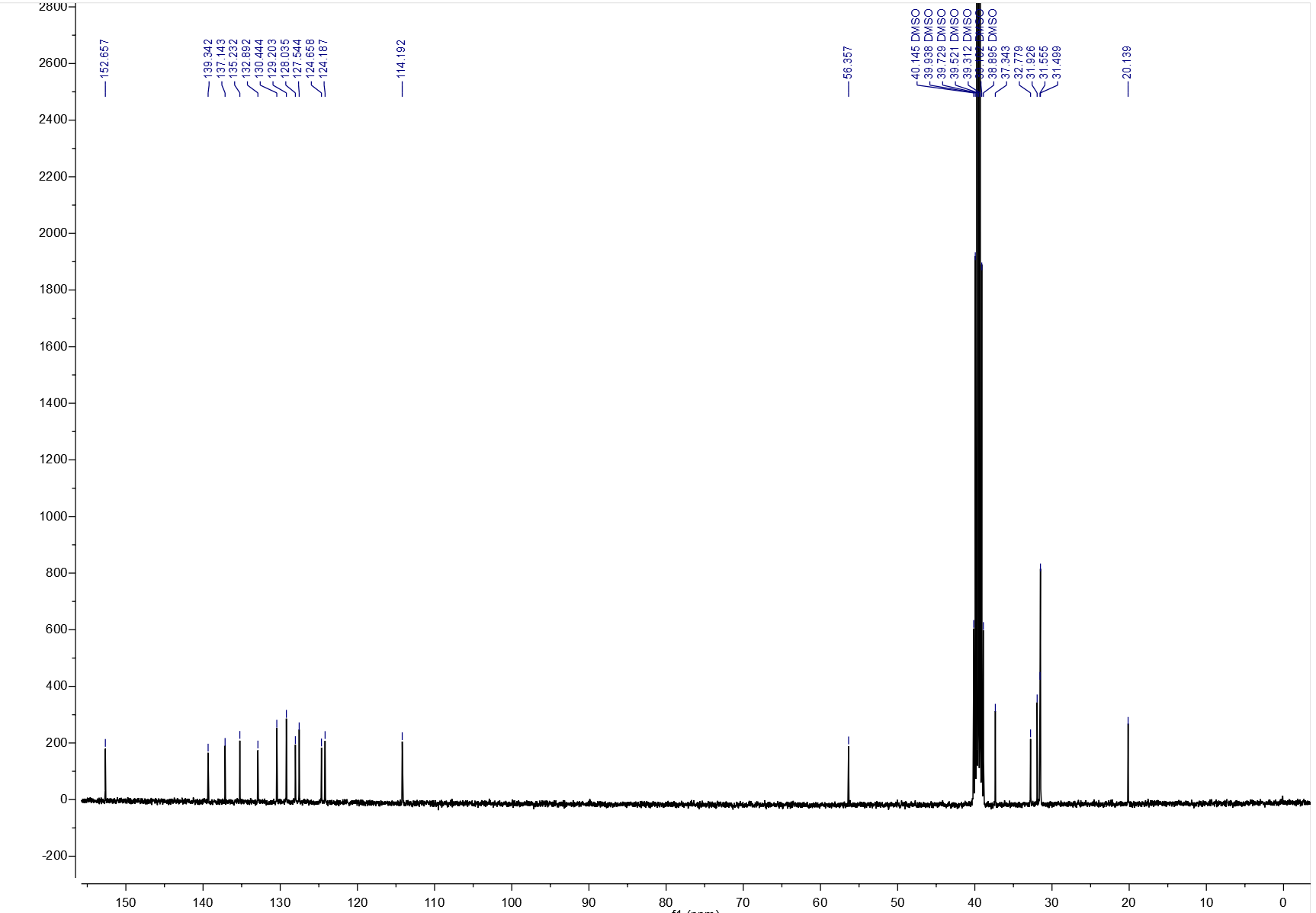


**
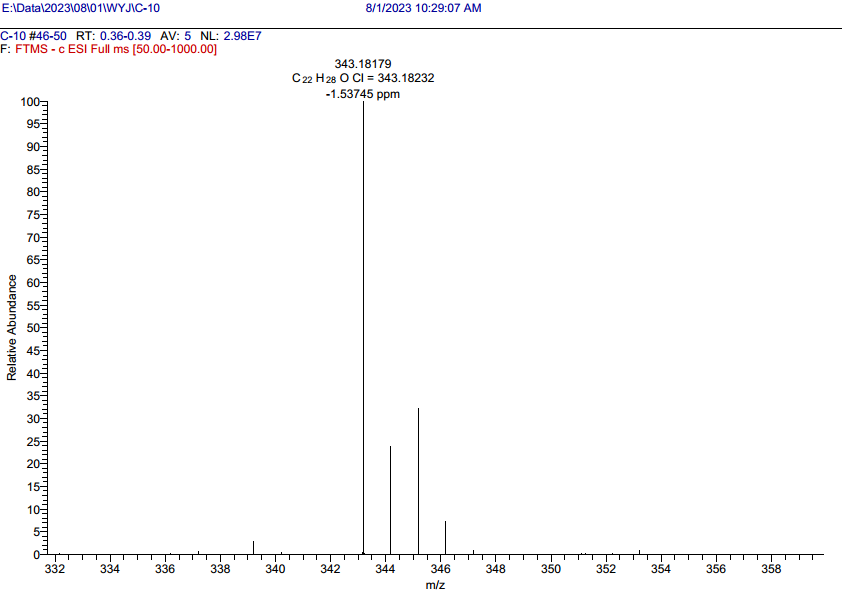
**

**
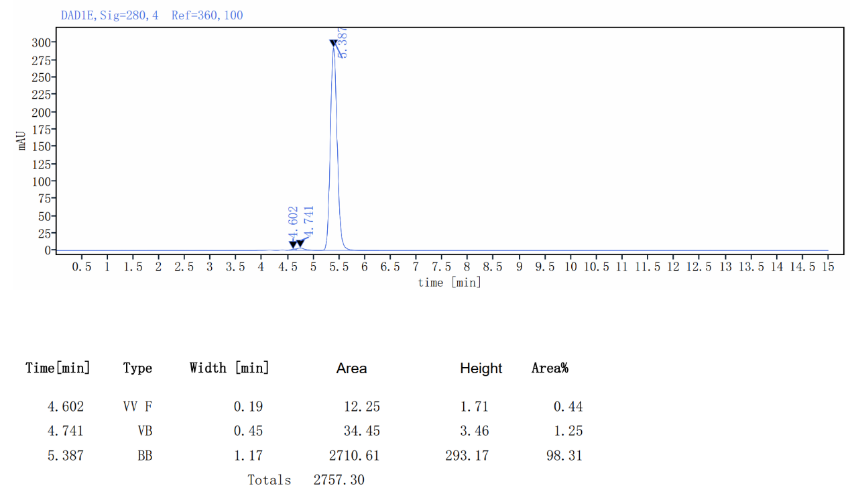
**

**Figure S24.** ^1^H NMR, ^13^C NMR, HRMS and HPLC data of compound **B1**.


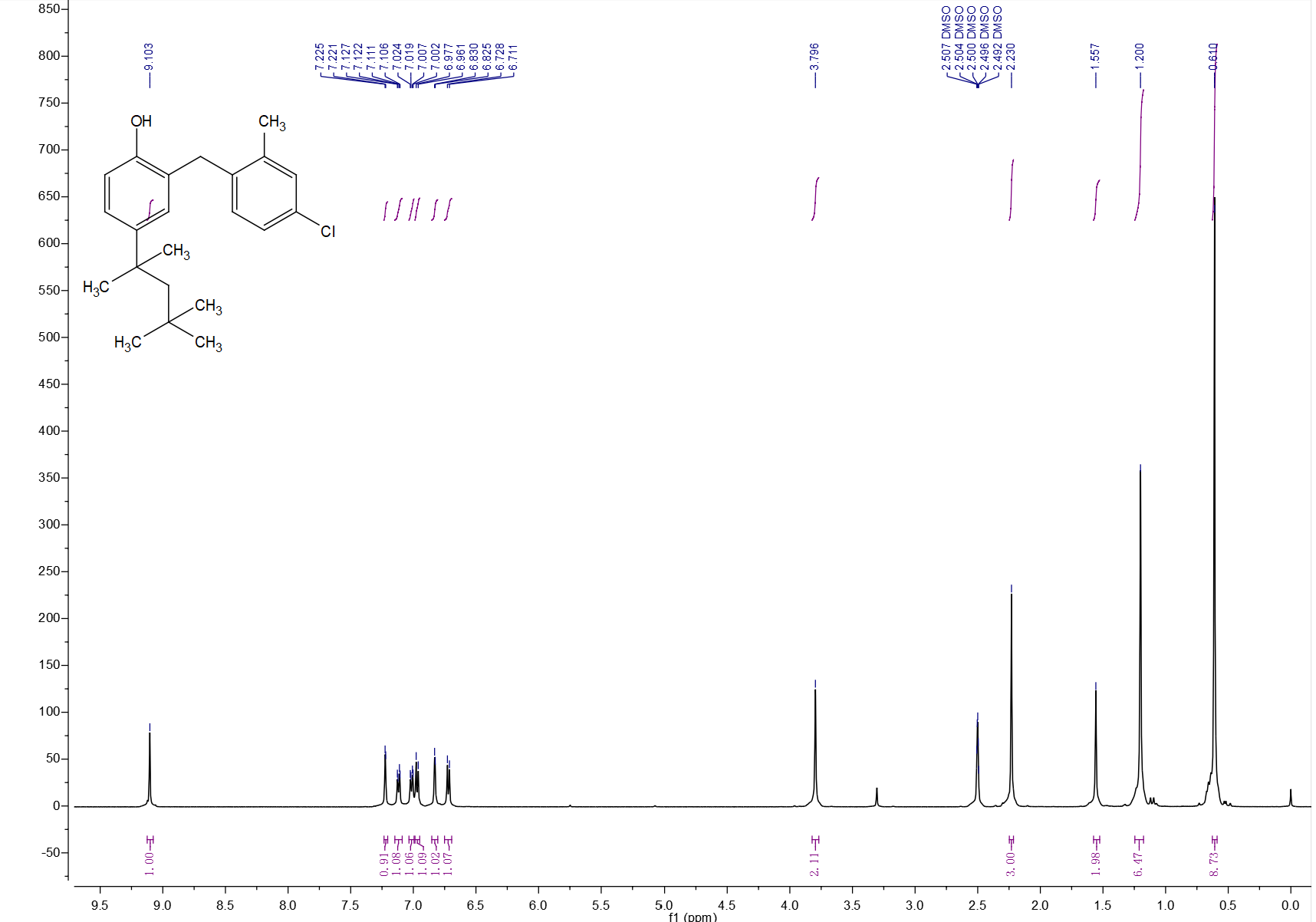


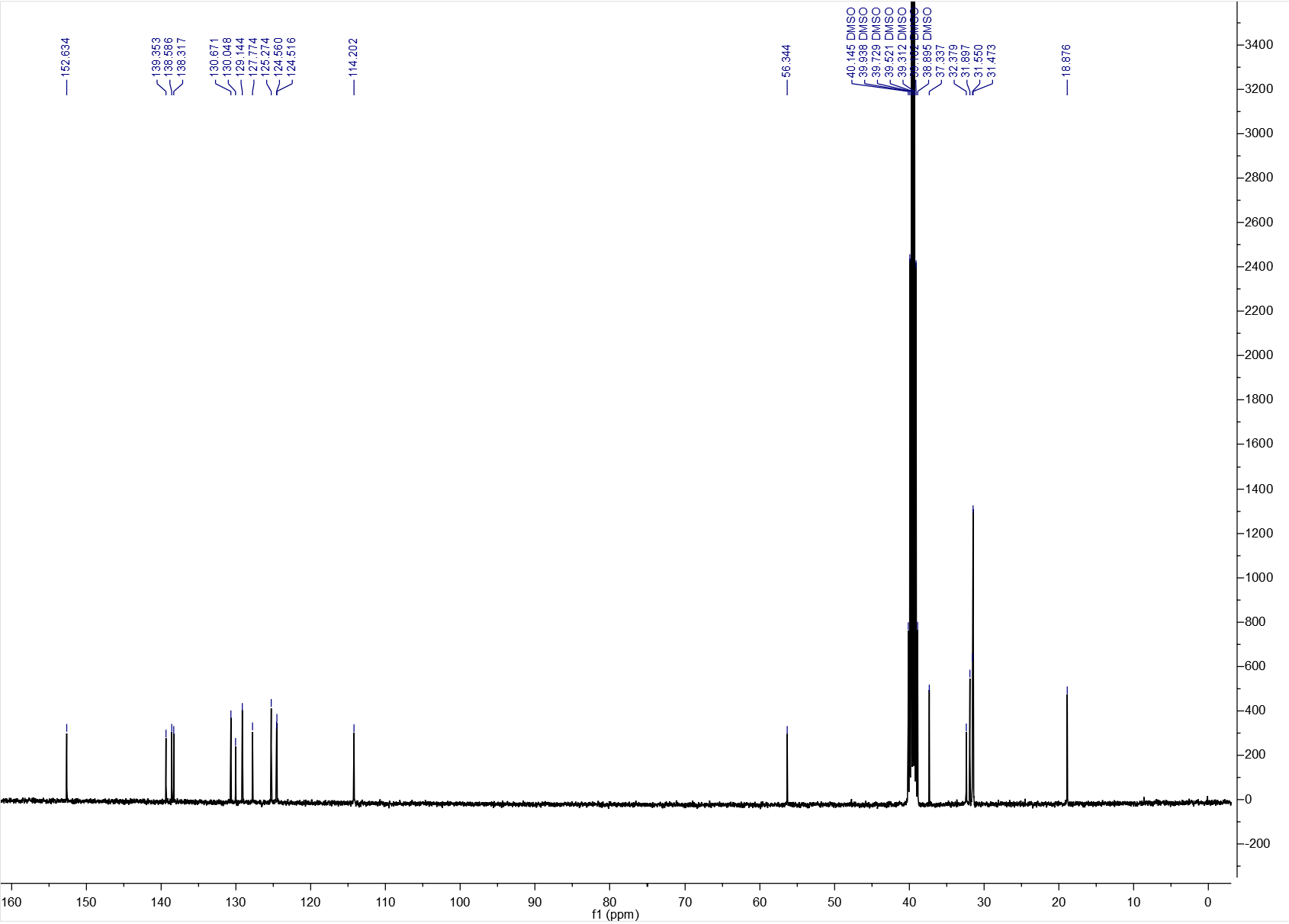


**
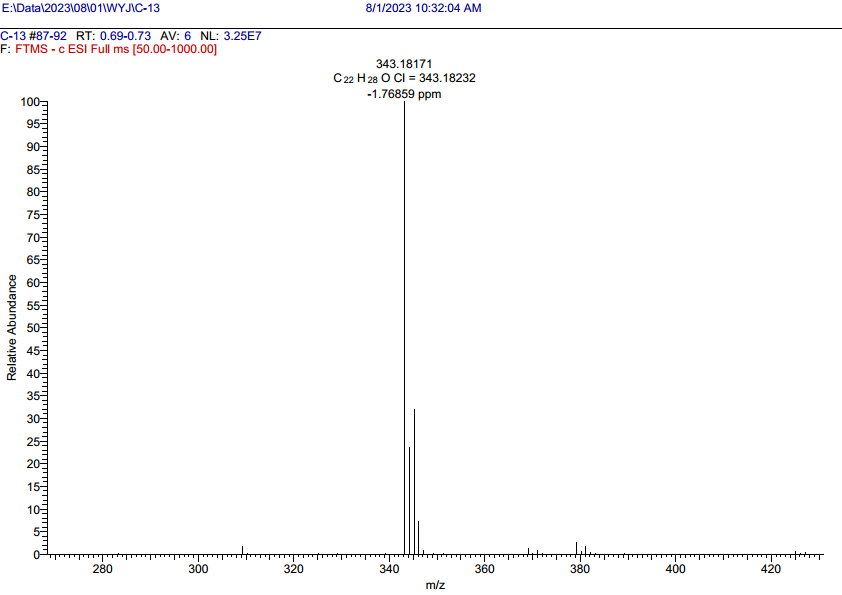
**

**
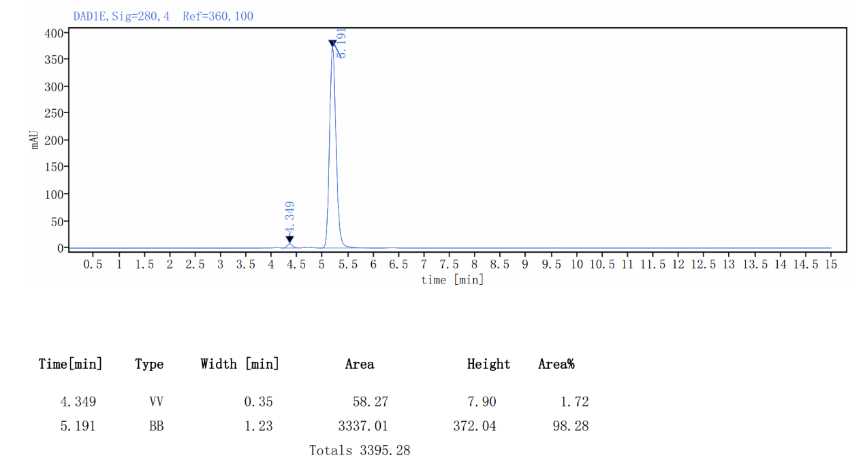
**

**Figure S25.** ^1^H NMR, ^13^C NMR, HRMS and HPLC data of compound **B2**.


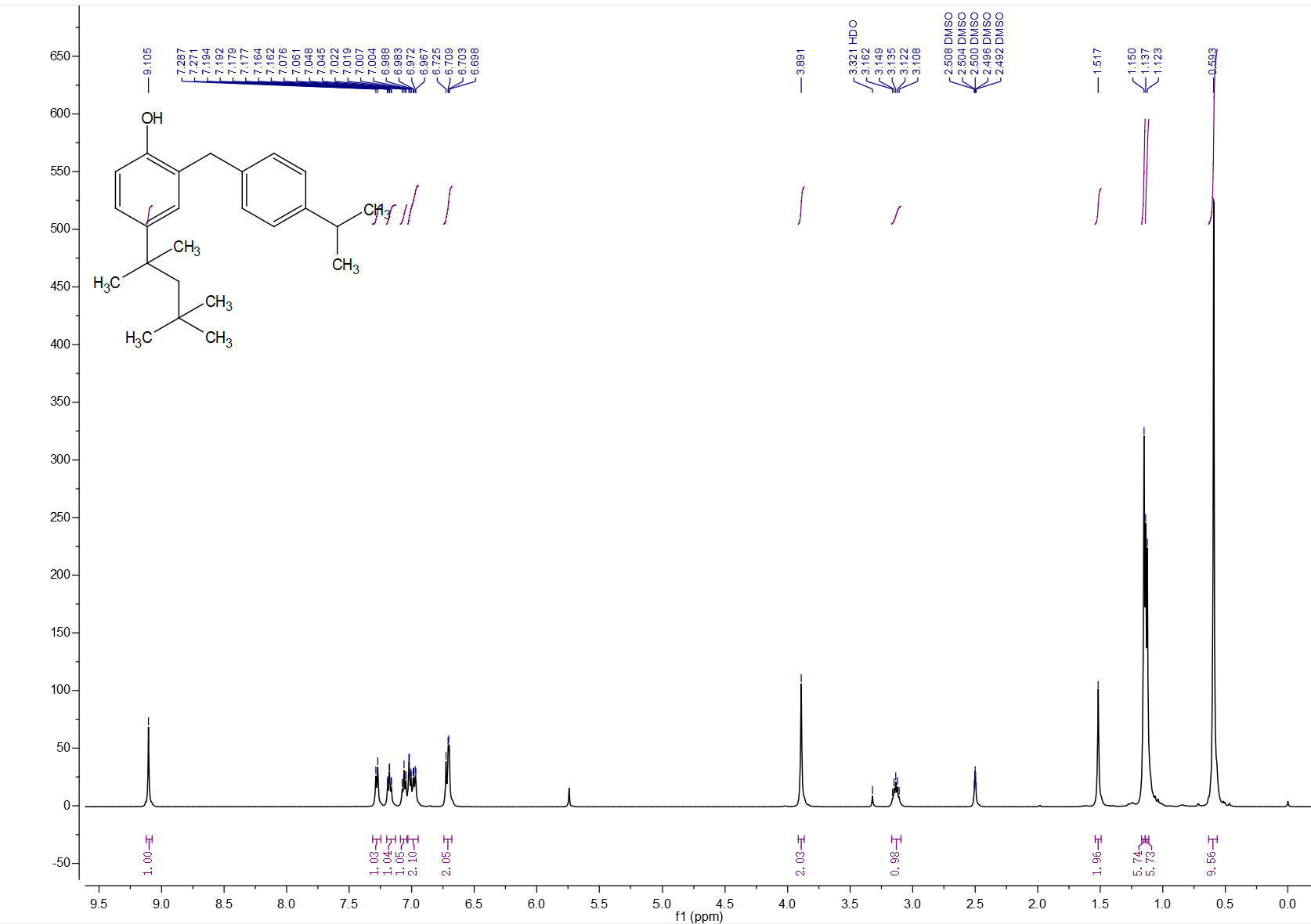


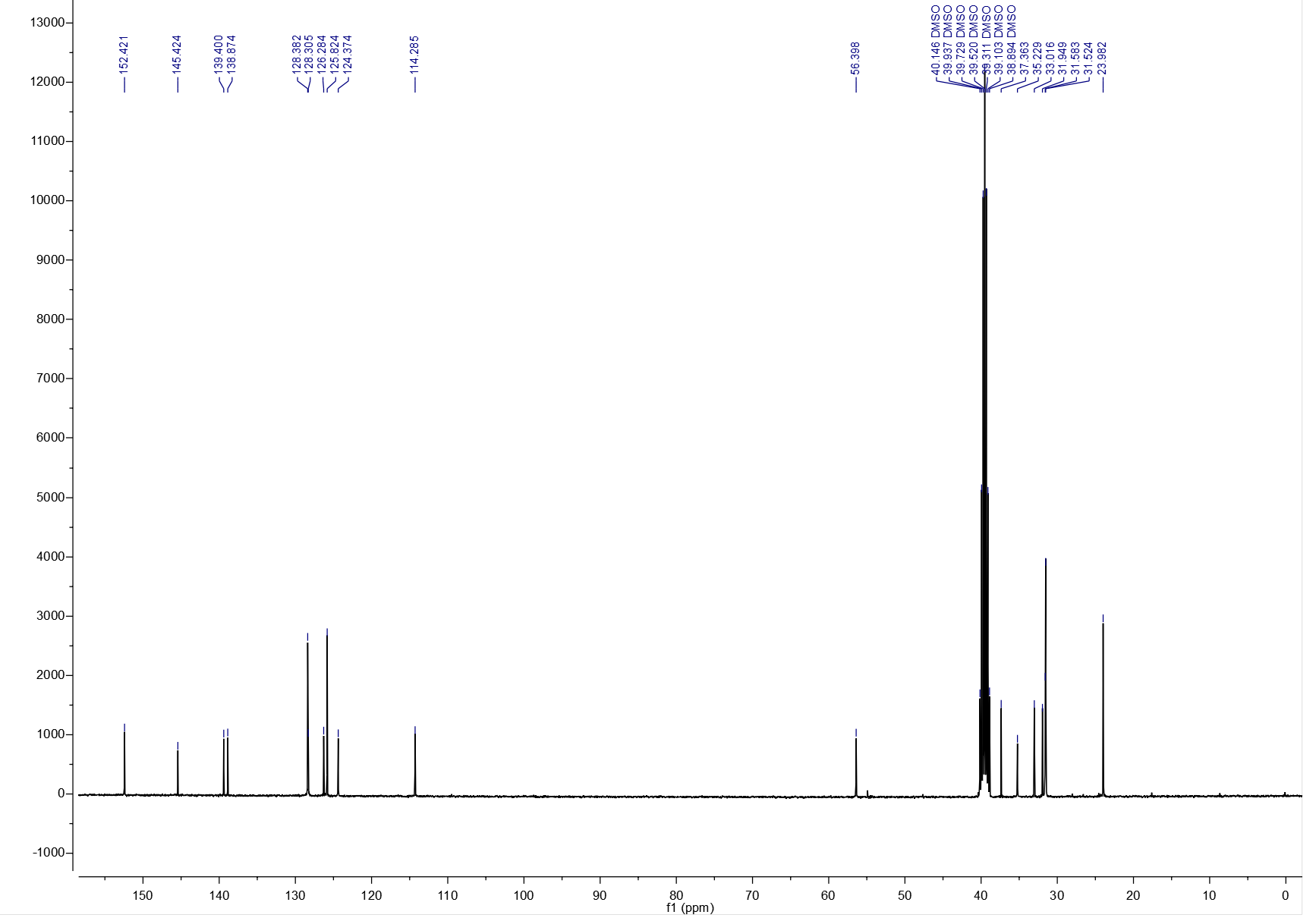


**
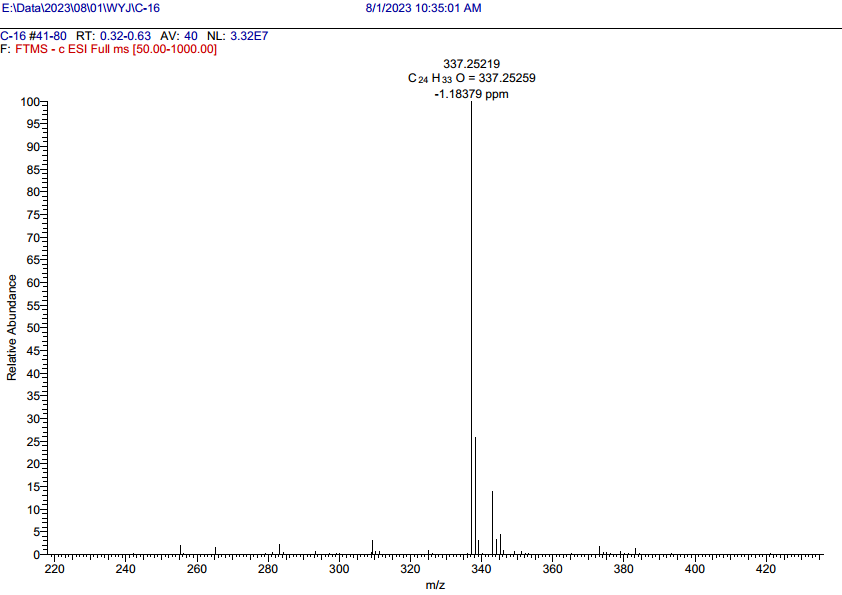
**

**
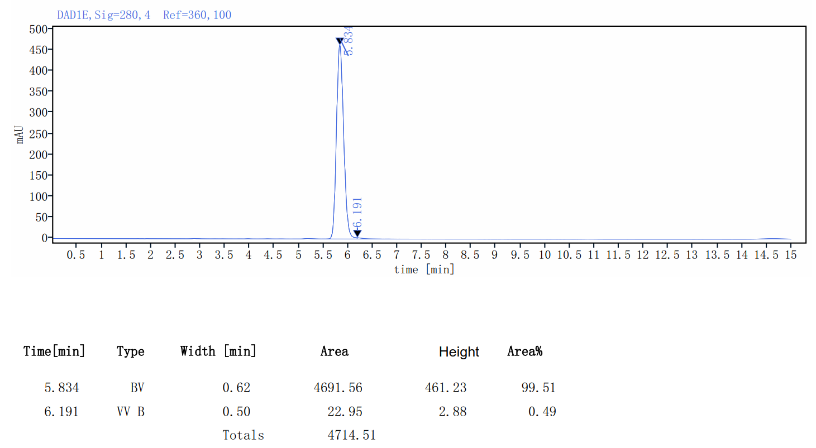
**

**Figure S26.** ^1^H NMR, ^13^C NMR, HRMS and HPLC data of compound **B3**.


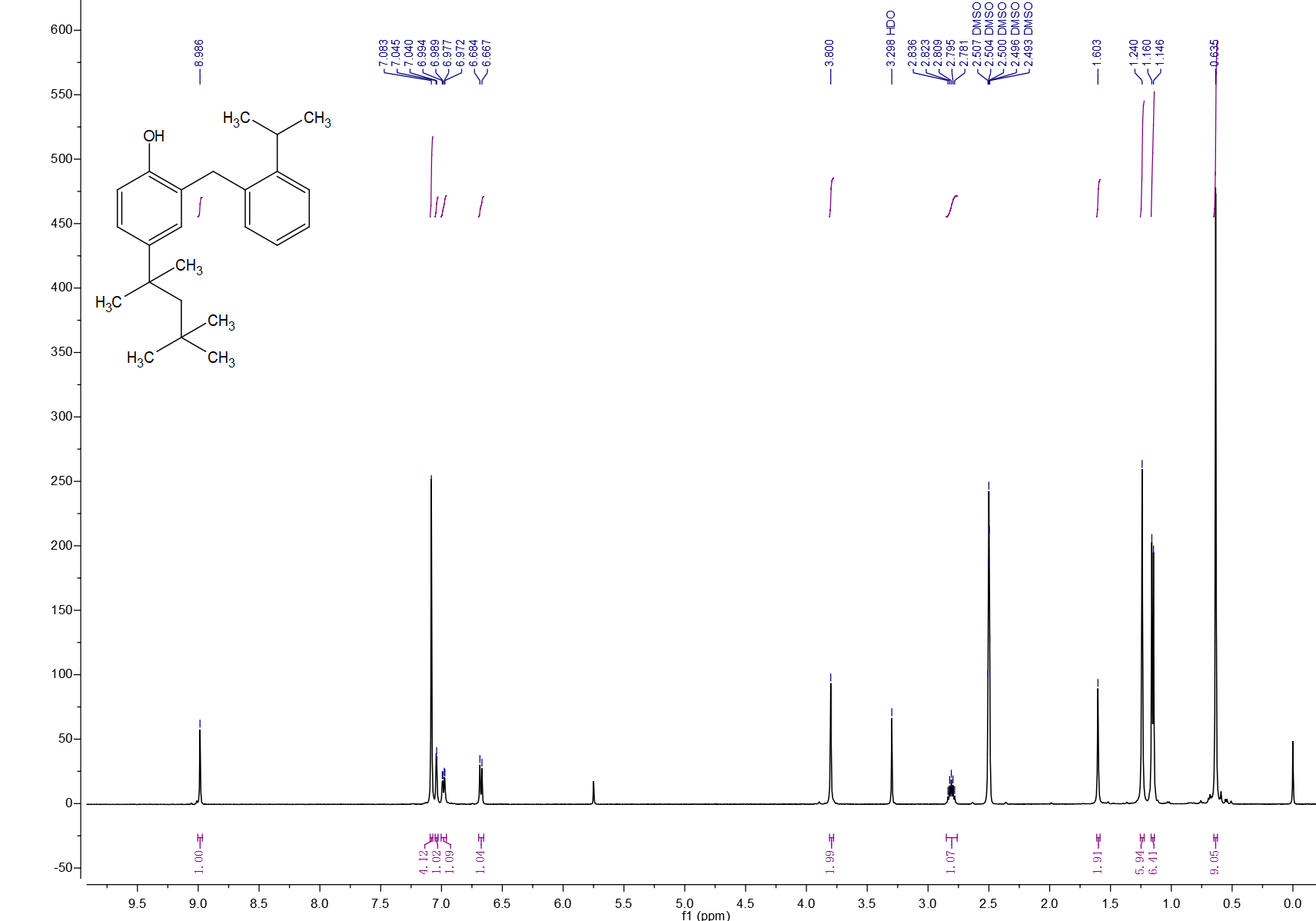


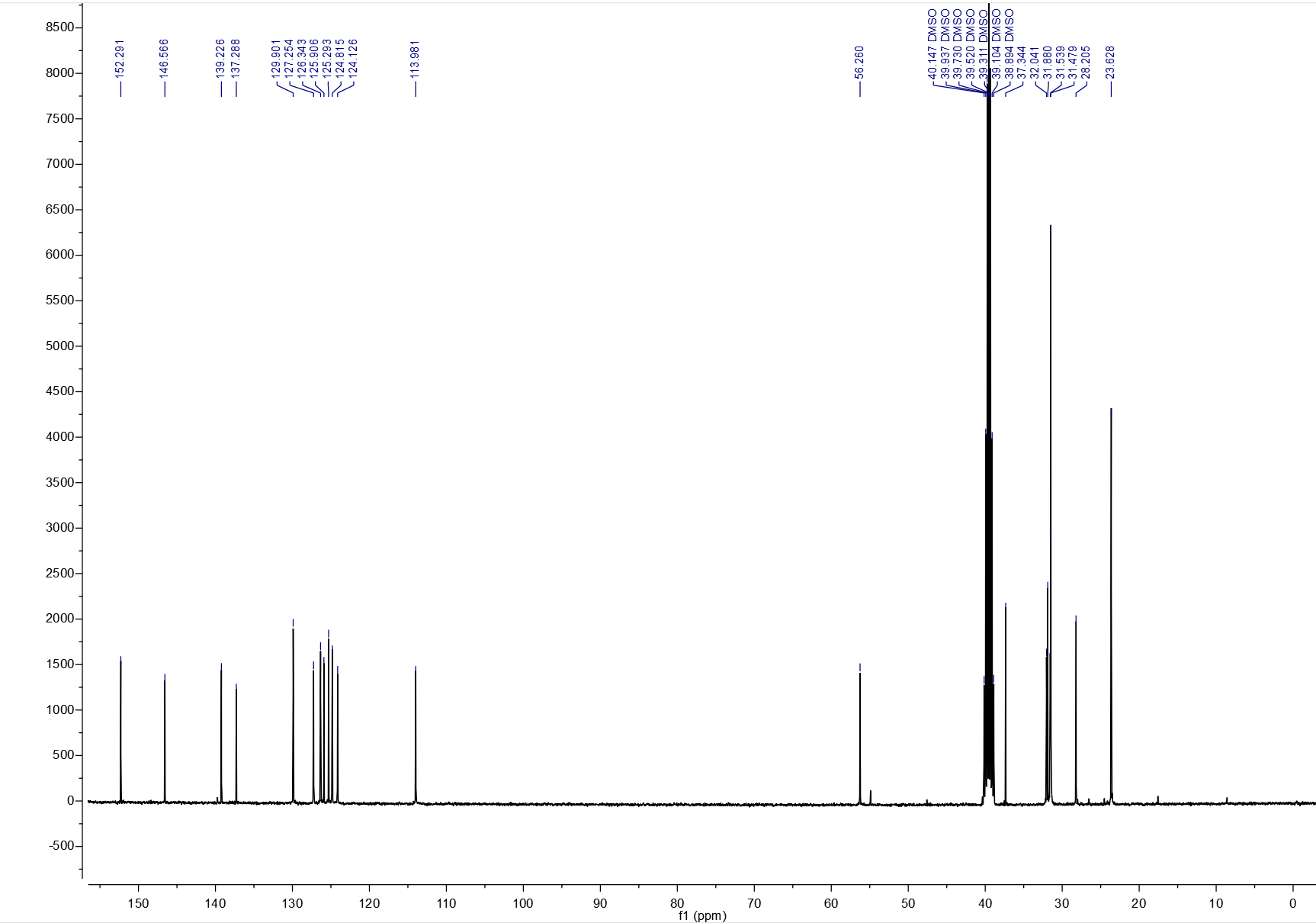


**
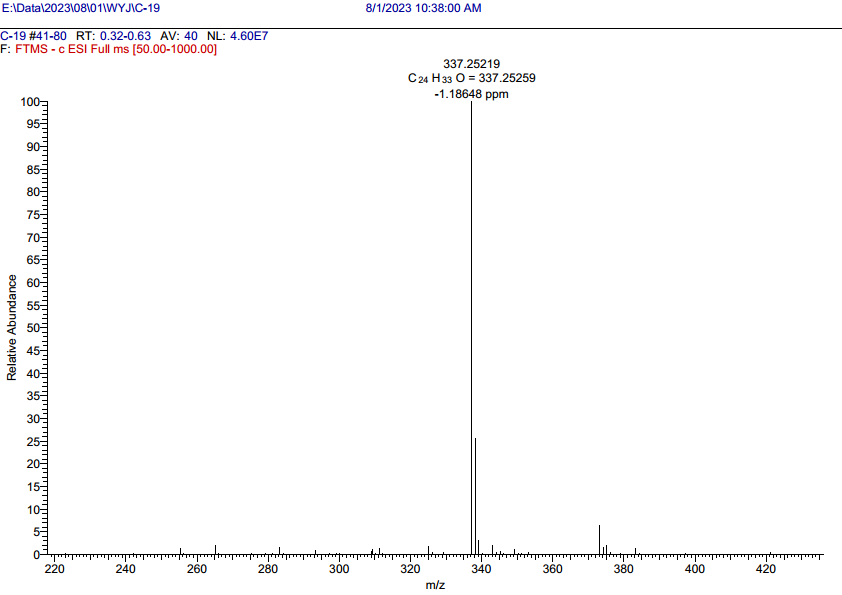
**

**
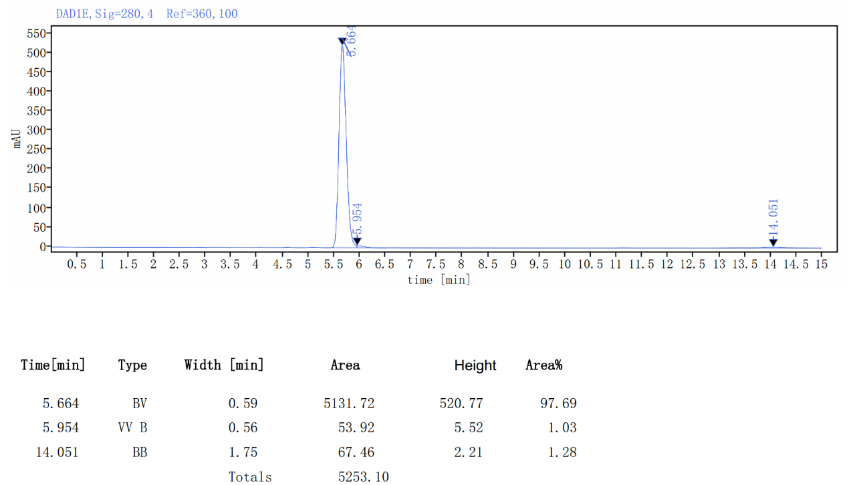
**

**Figure S27.** ^1^H NMR, ^13^C NMR, HRMS and HPLC data of compound **B4**.


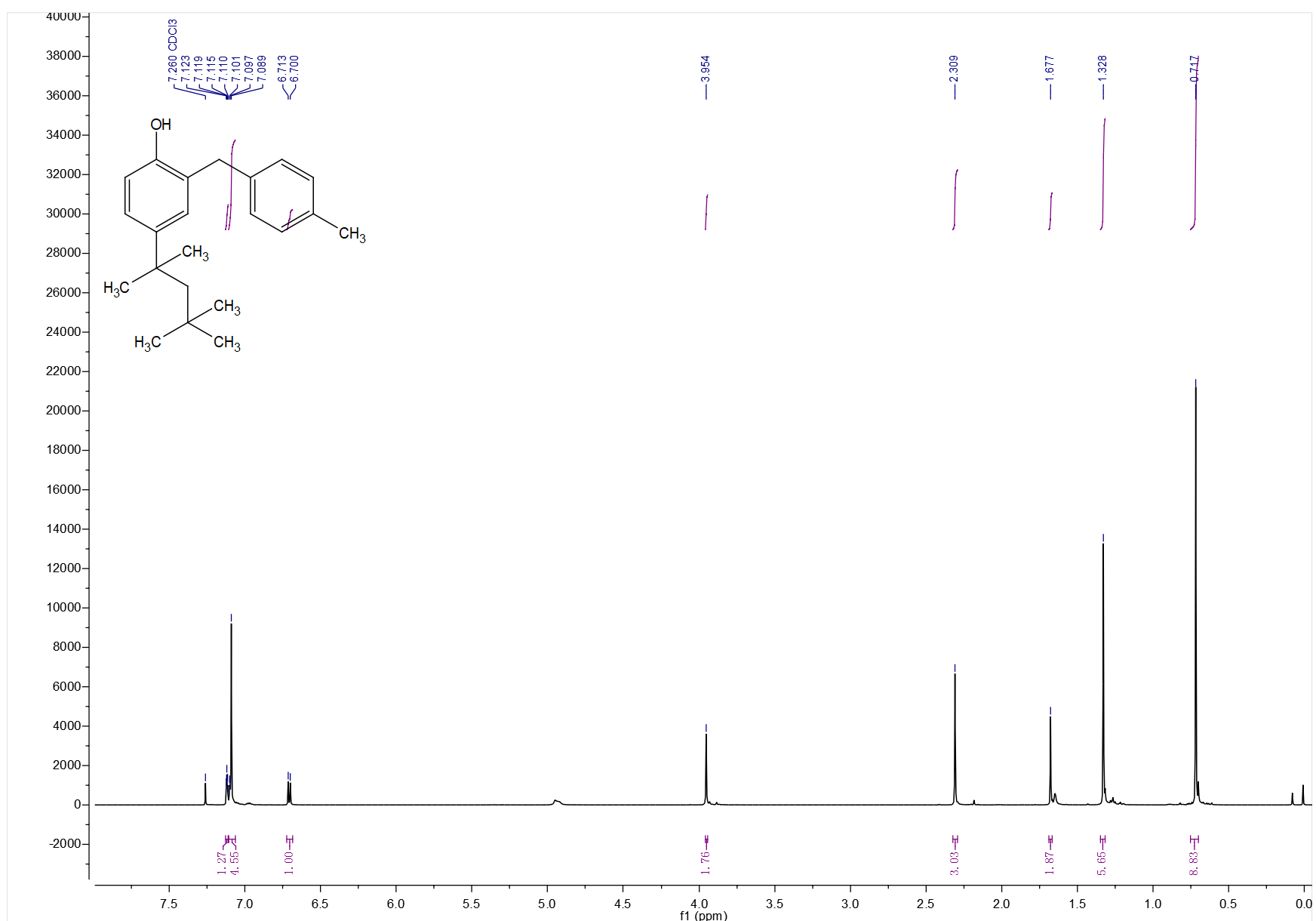


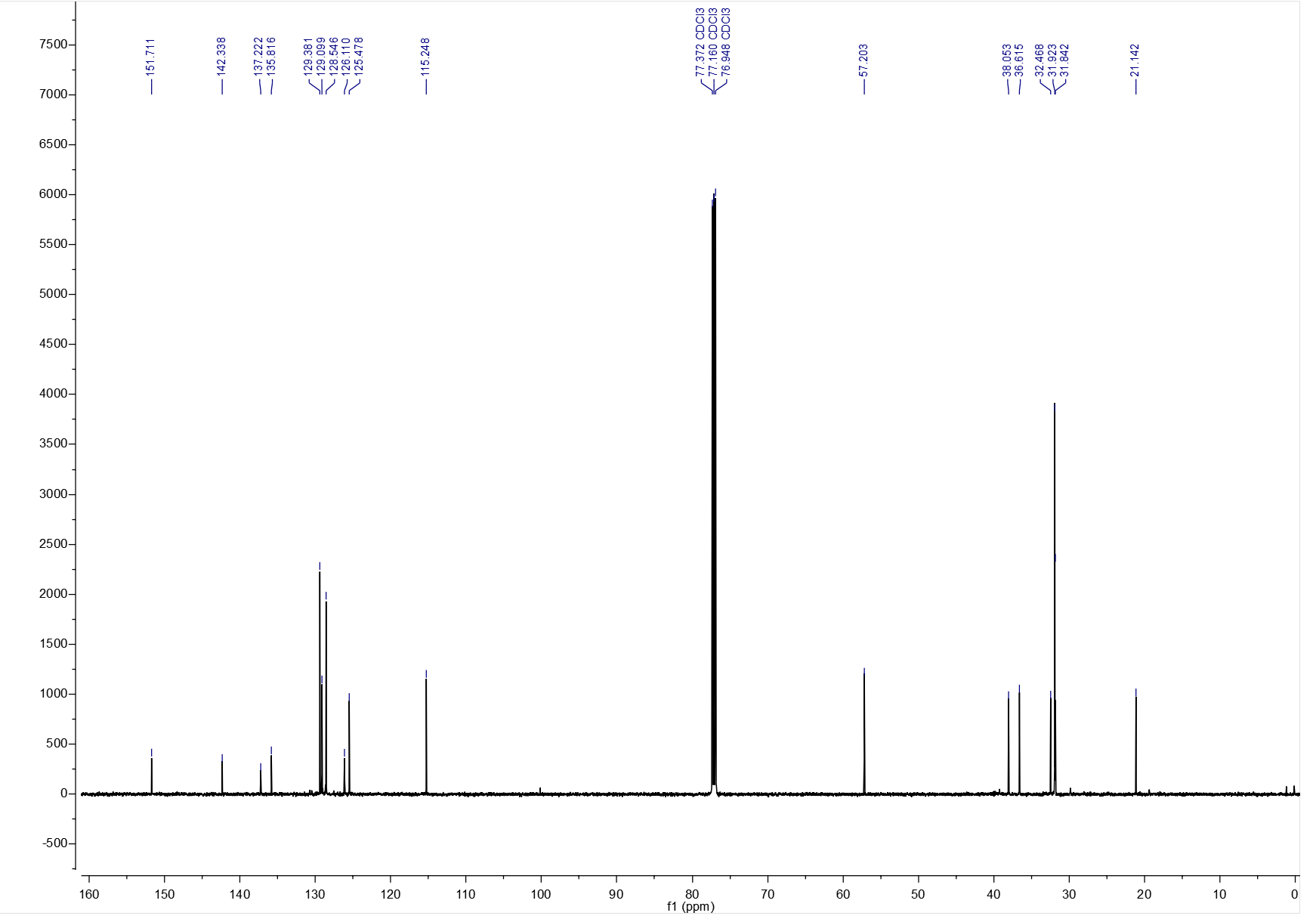


**
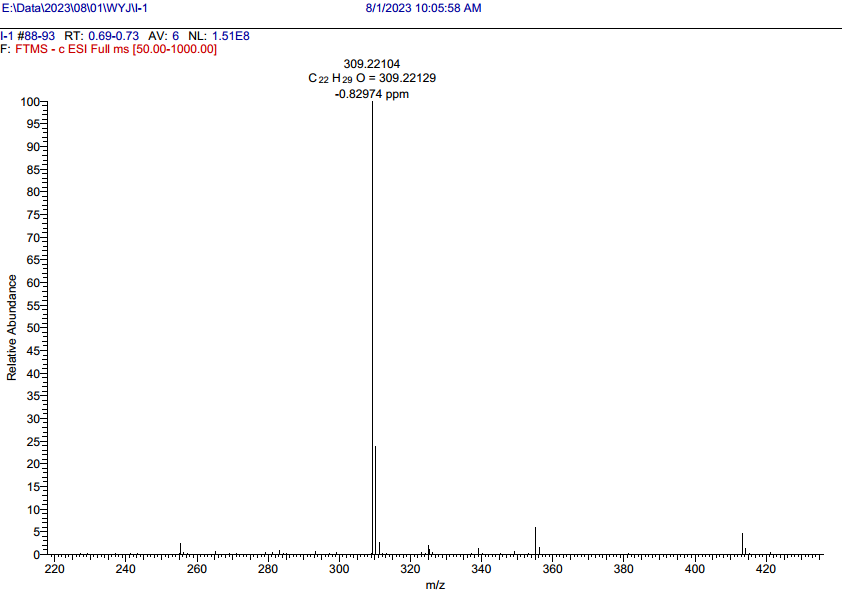
**

**
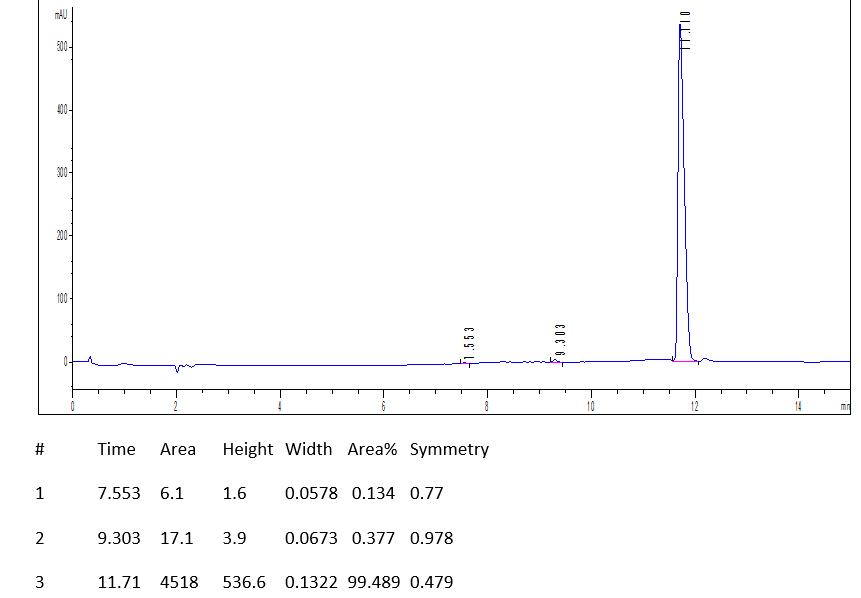
**

**Figure S28.** ^1^H NMR, ^13^C NMR, HRMS and HPLC data of compound **B5**.


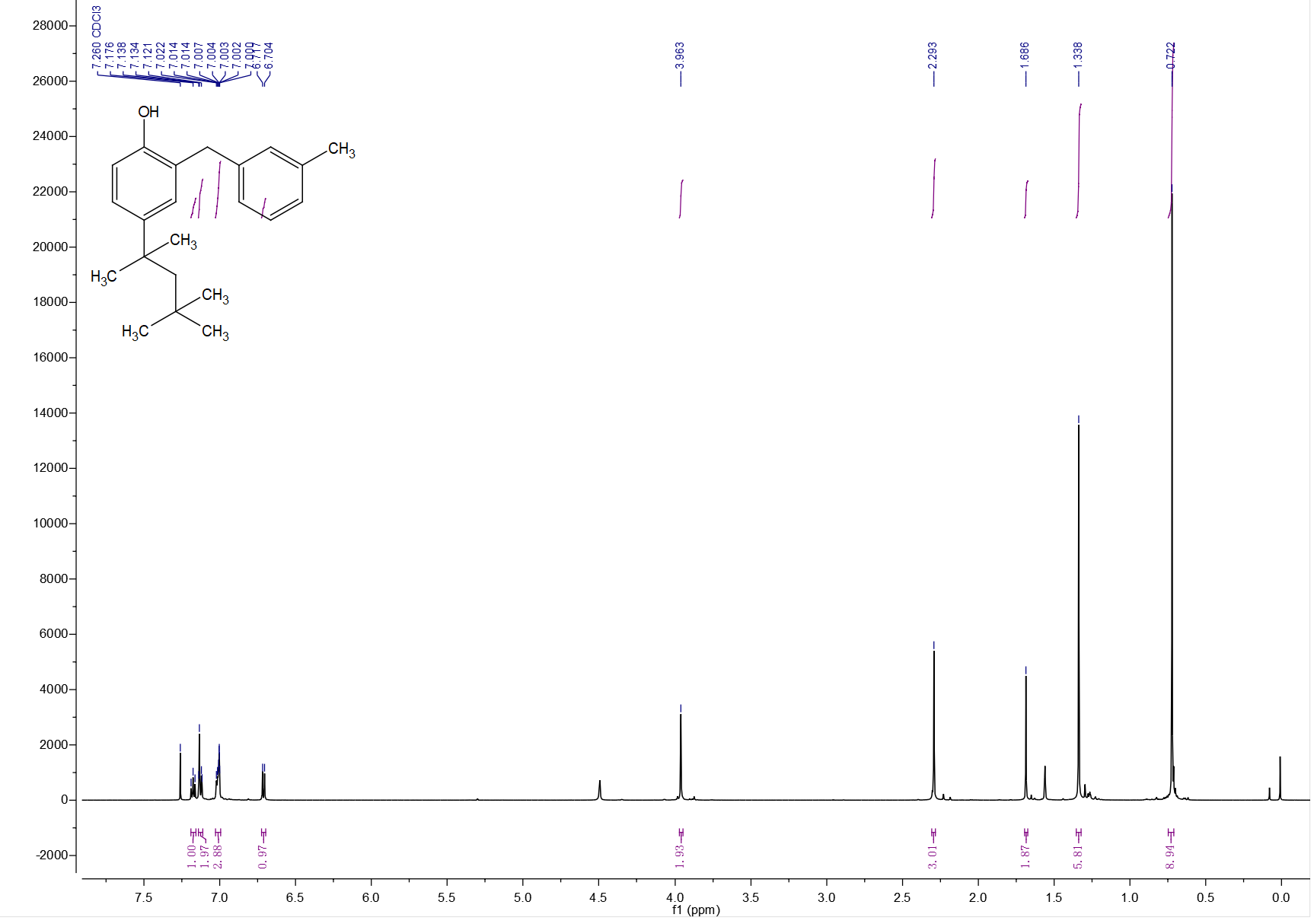


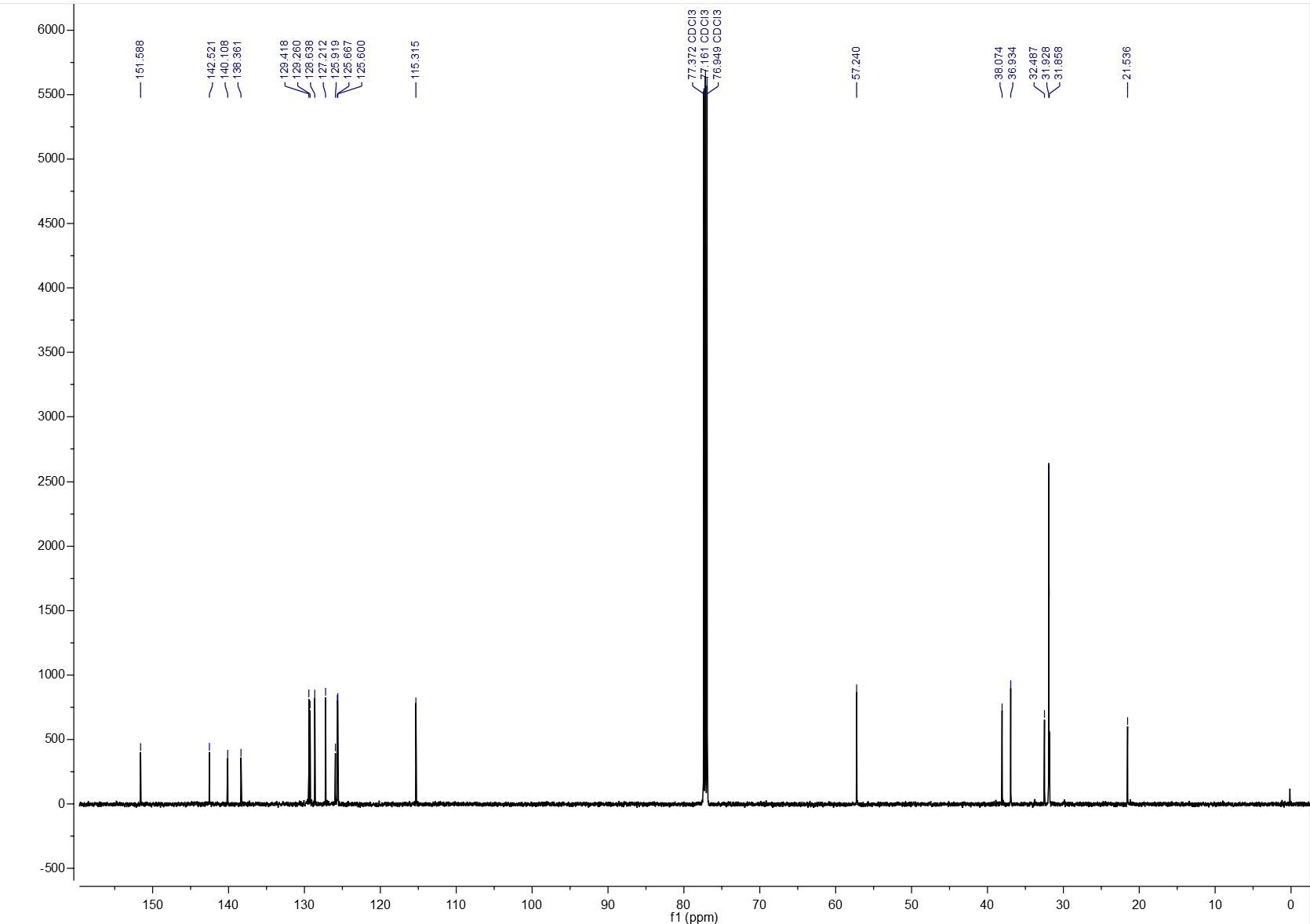


**
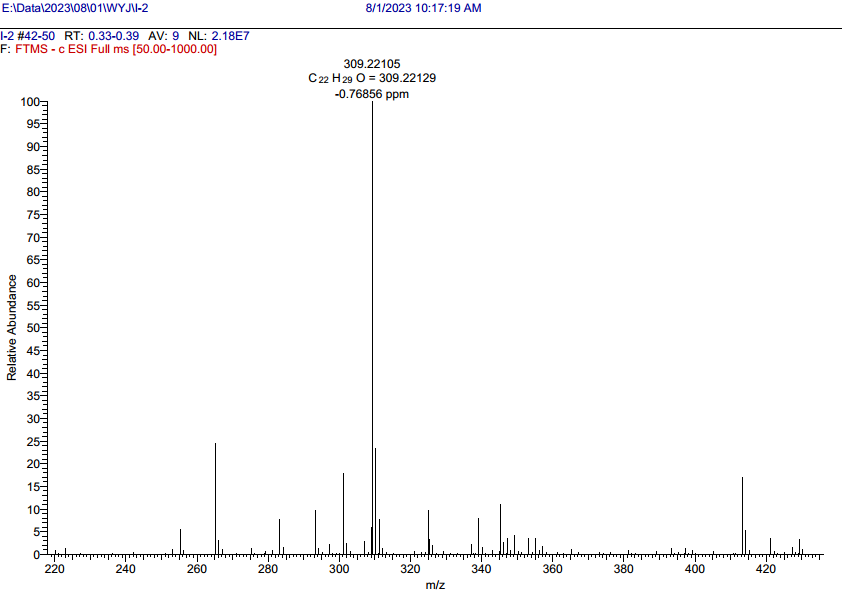
**

**
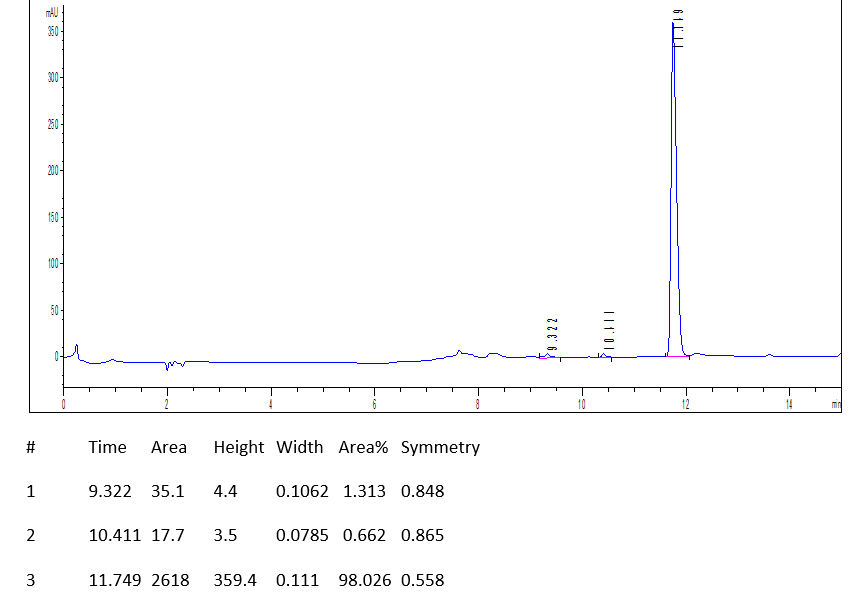
**

**Figure S29.** ^1^H NMR, ^13^C NMR, HRMS and HPLC data of compound **B6**.


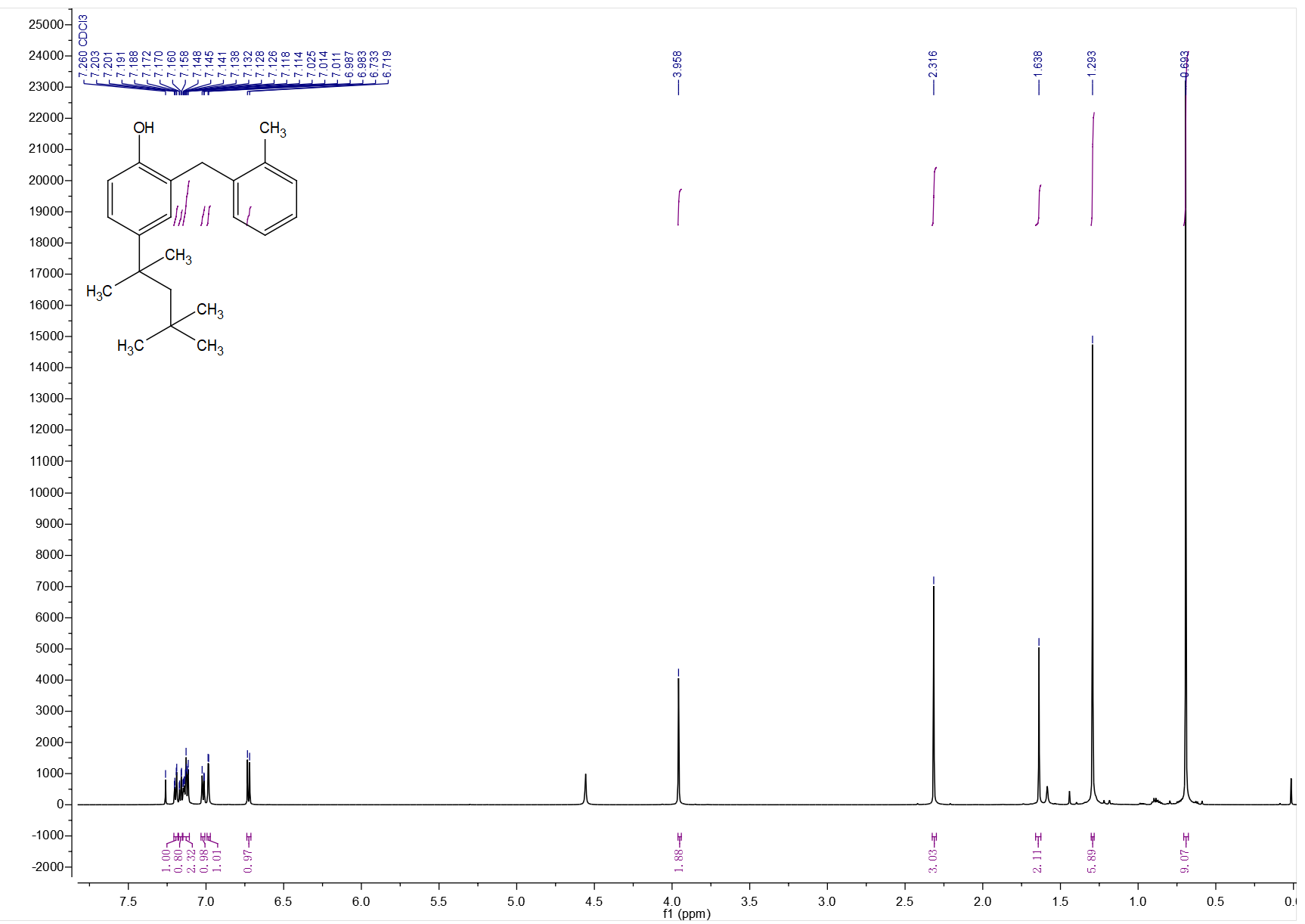


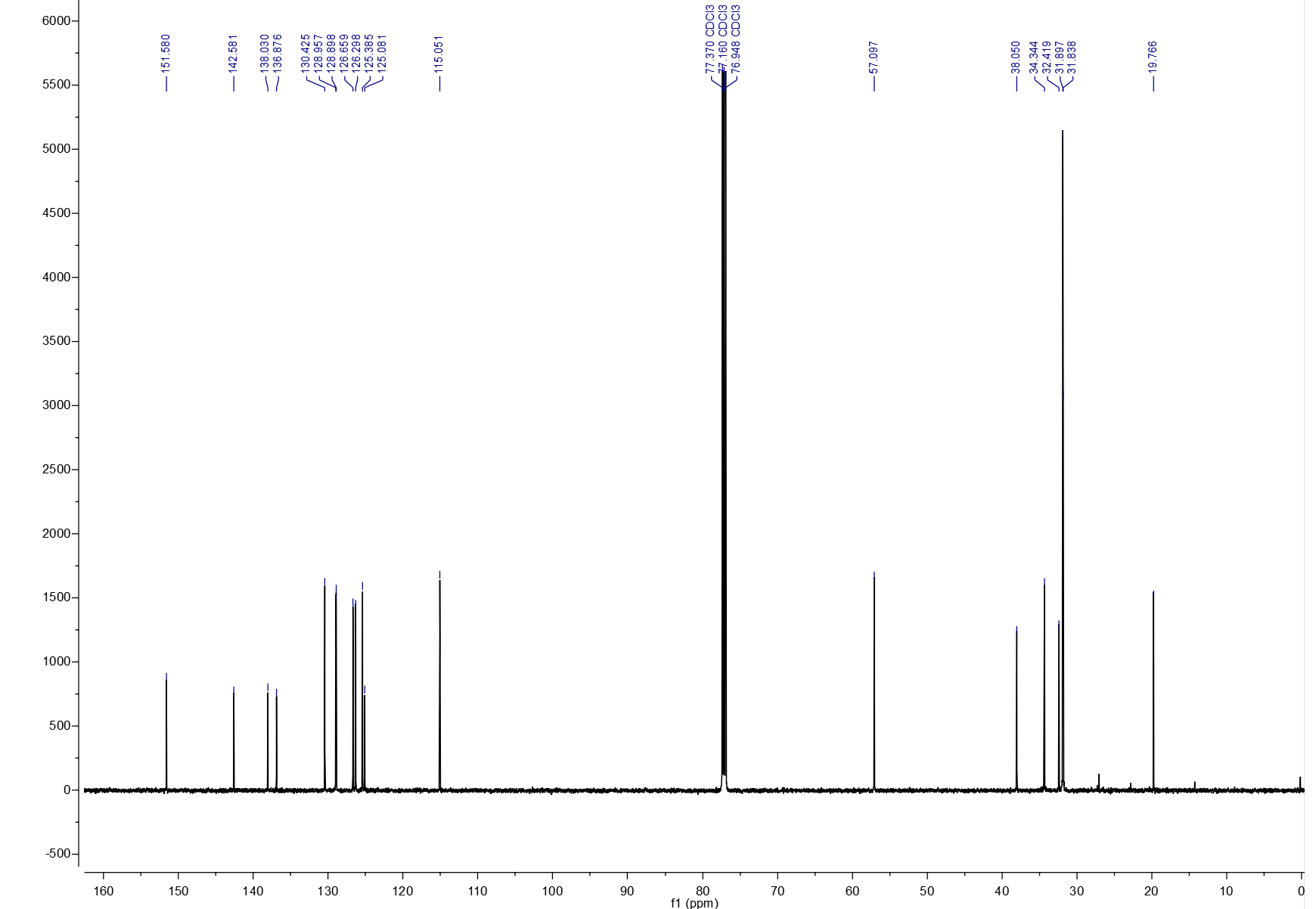


**
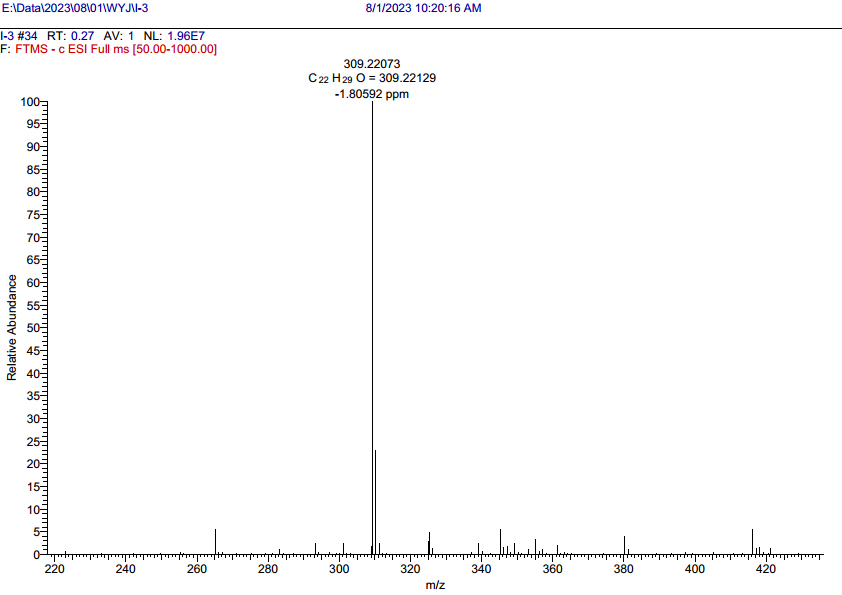
**

**
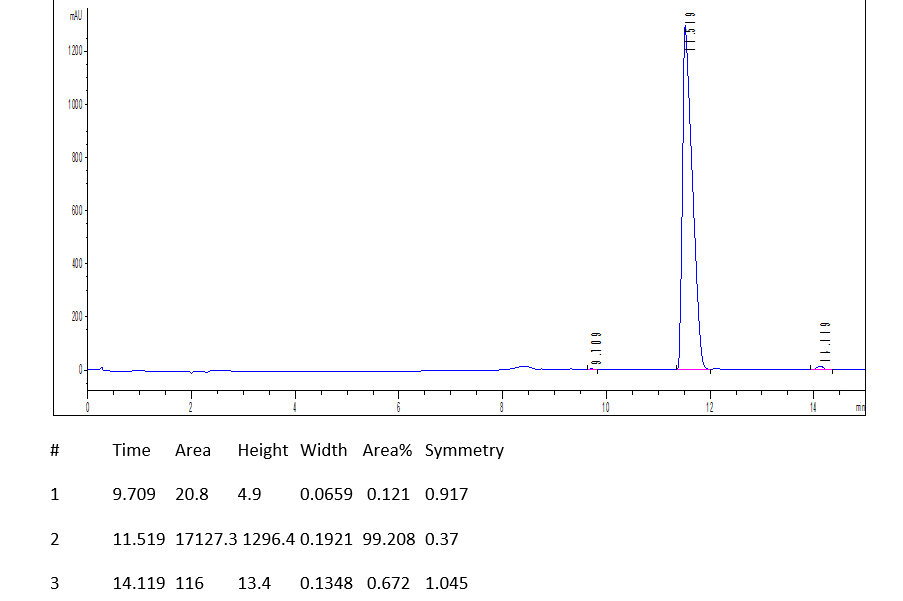
**

**Figure S30.** ^1^H NMR, ^13^C NMR, HRMS and HPLC data of compound **B7**.


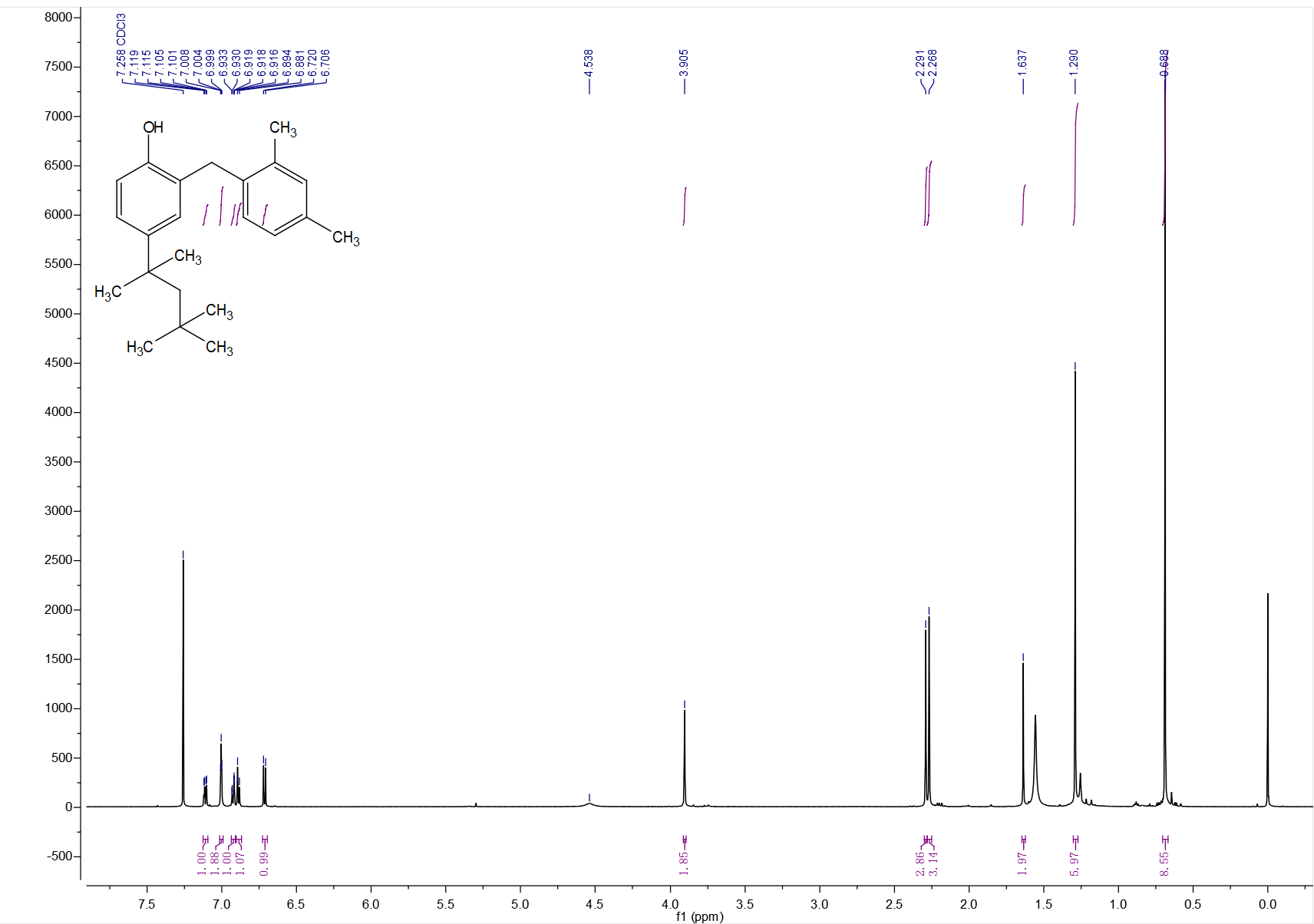


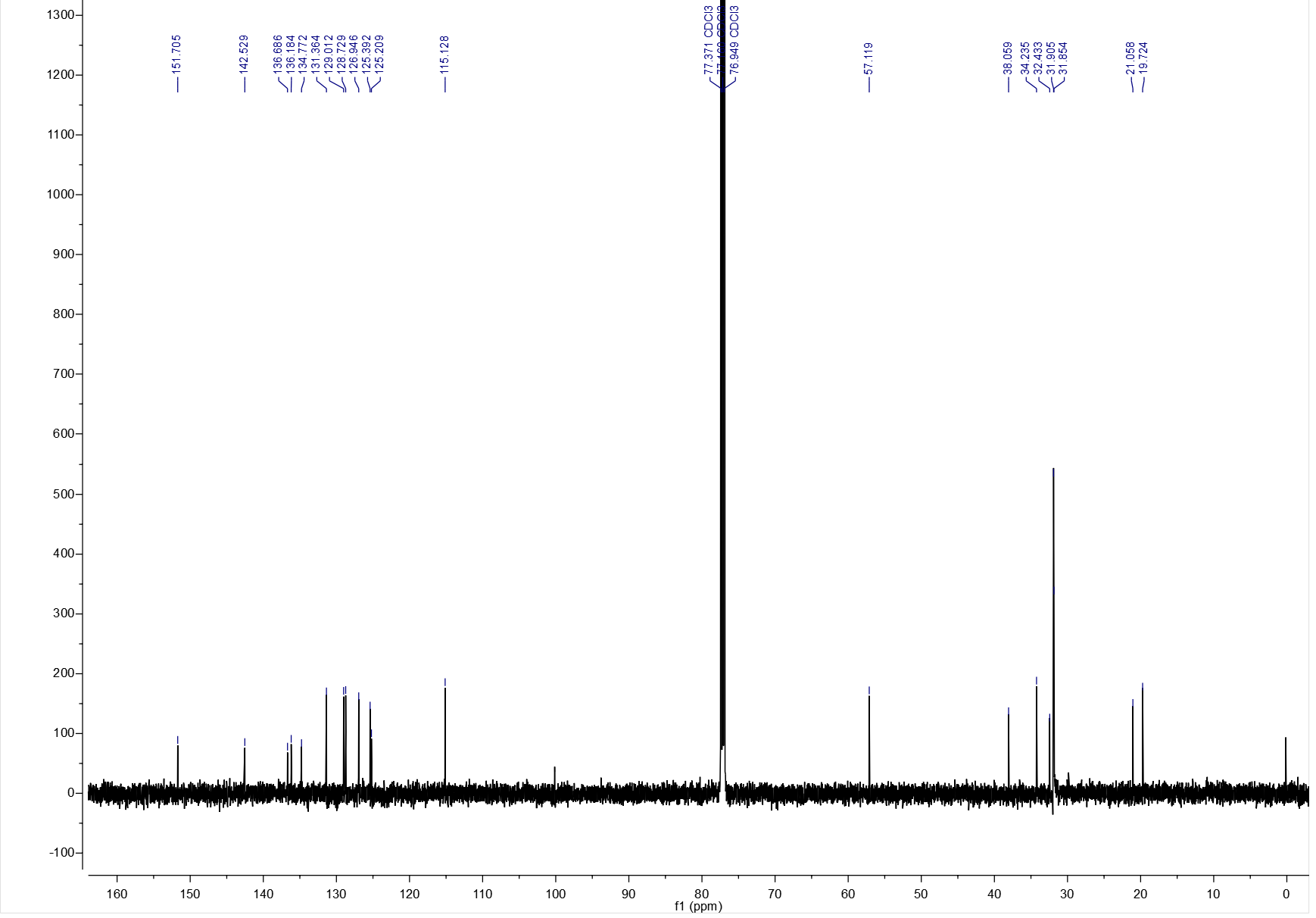


**
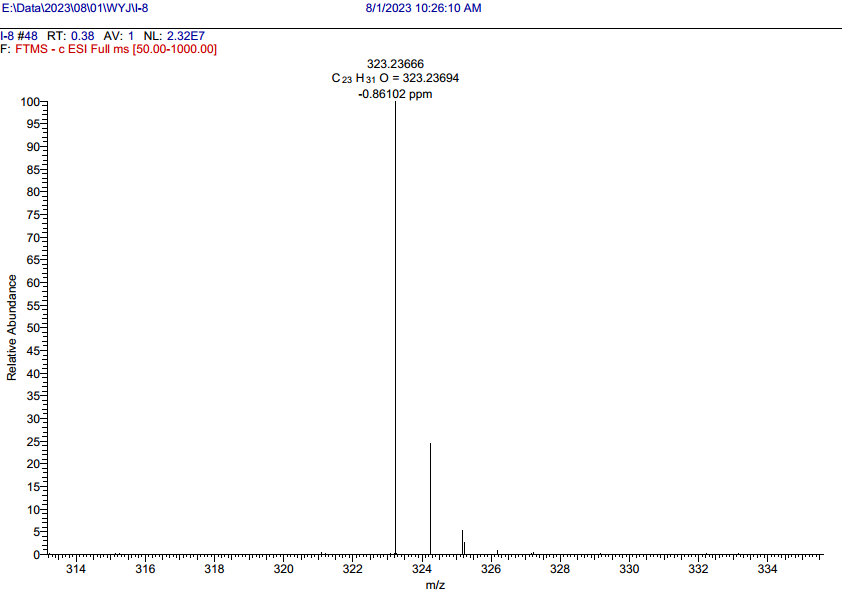
**

**
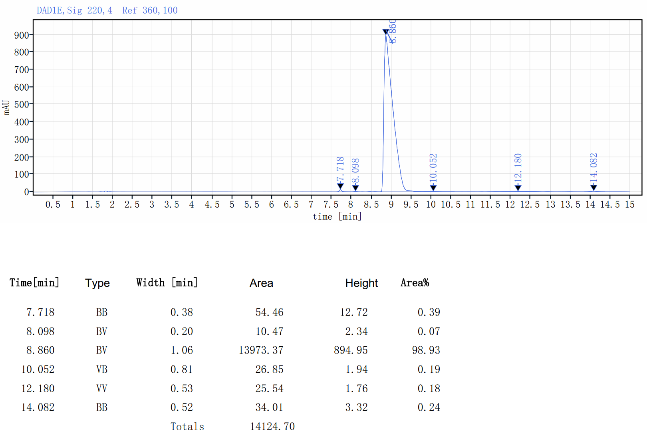
**

**Figure S31.** ^1^H NMR, ^13^C NMR, HRMS and HPLC data of compound **B8**.


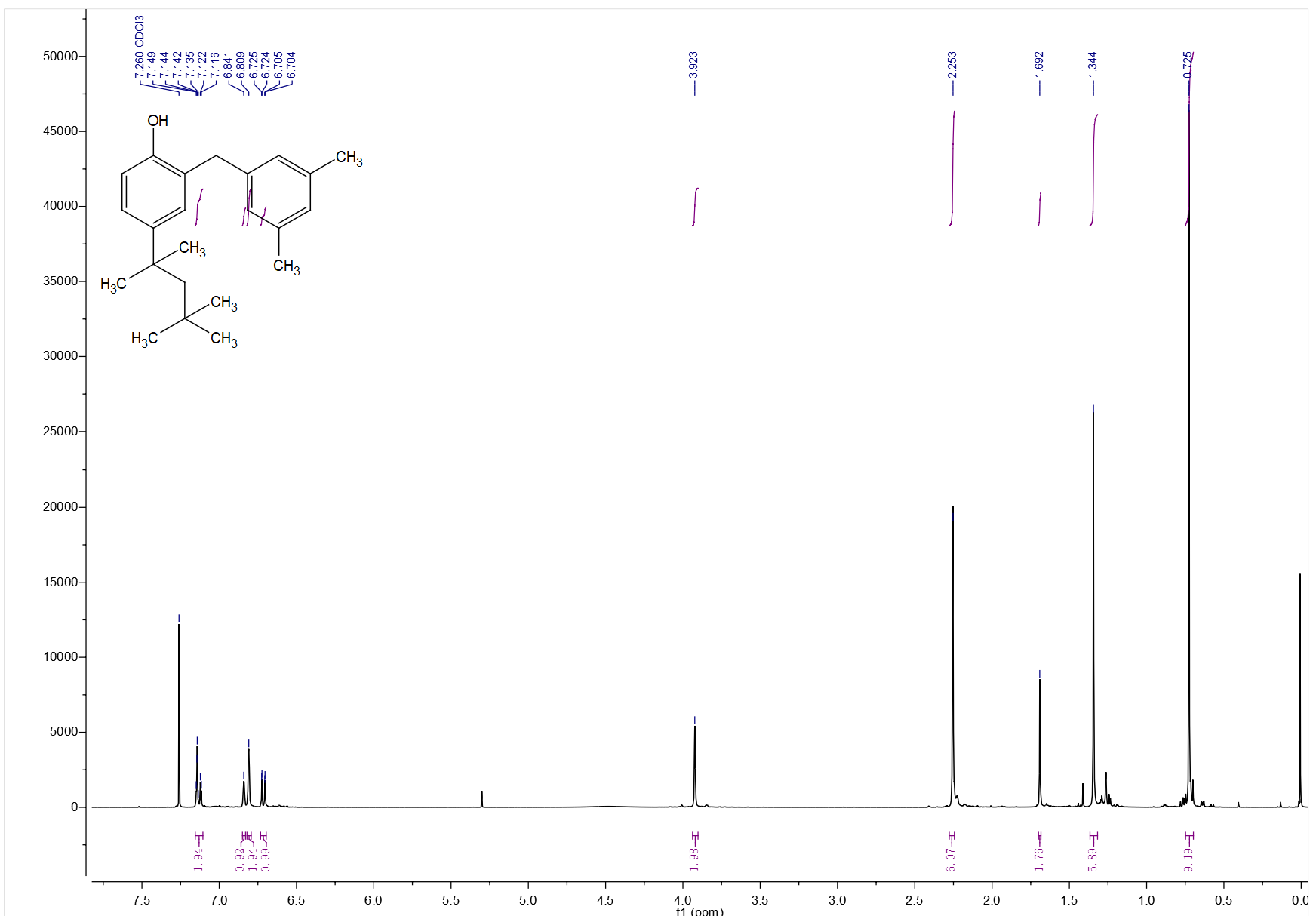


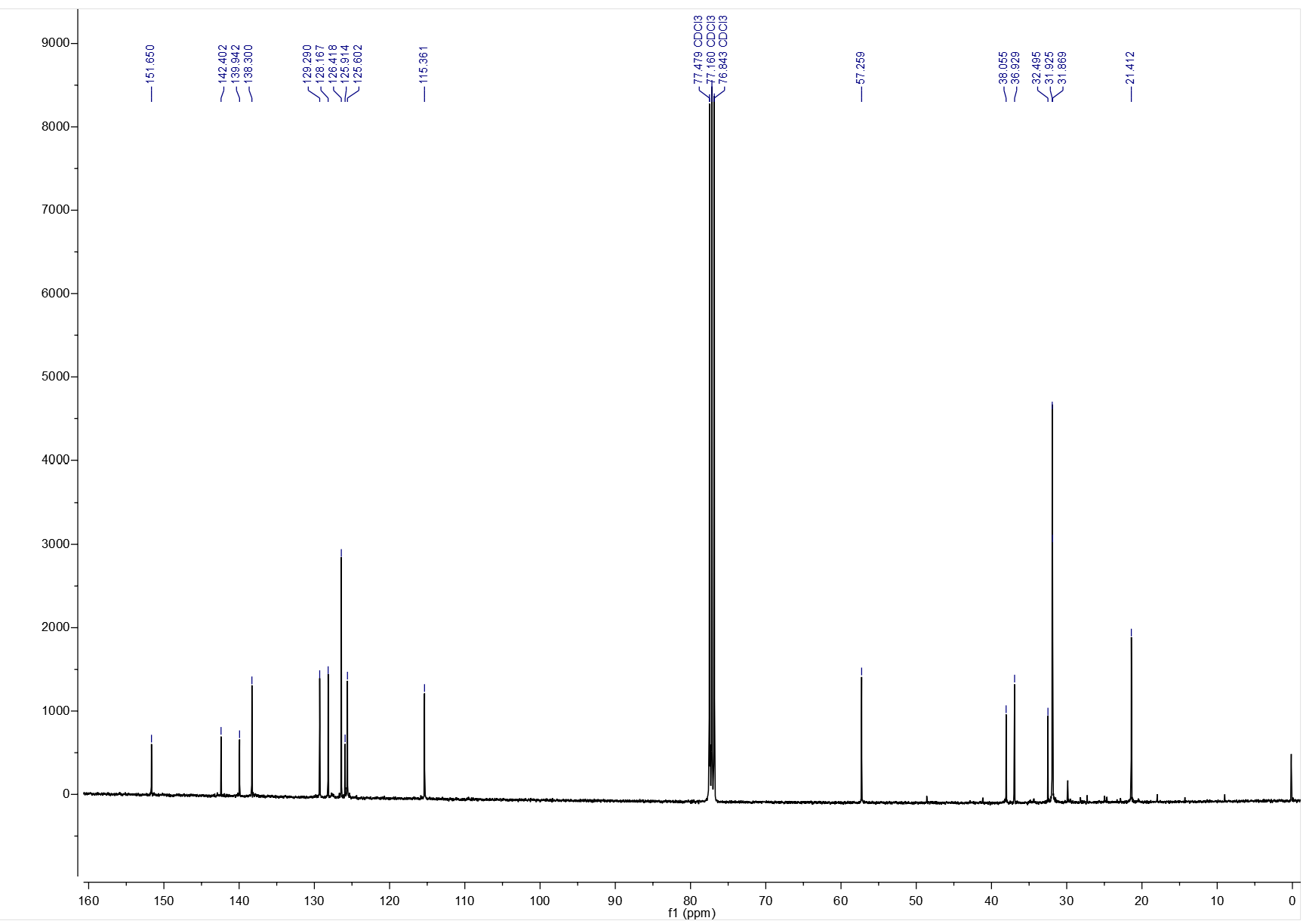


**
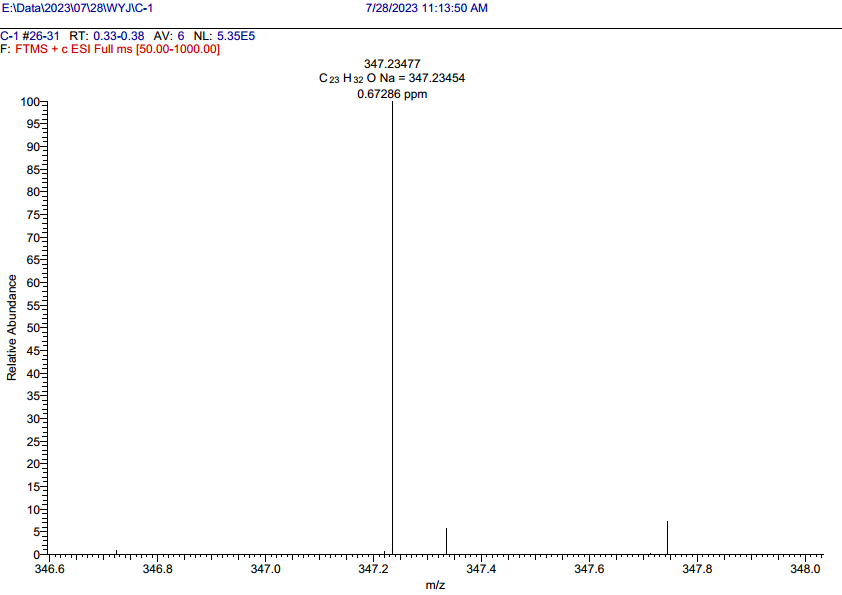
**

**
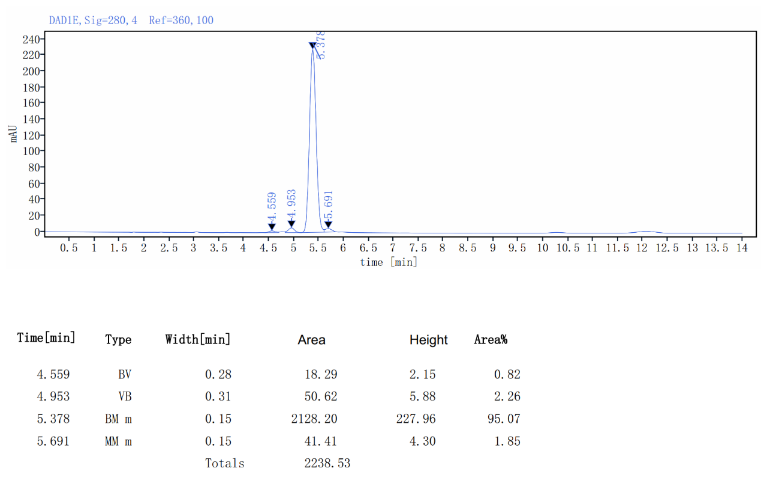
**

**Figure S32.** ^1^H NMR, ^13^C NMR, HRMS and HPLC data of compound **B9**.


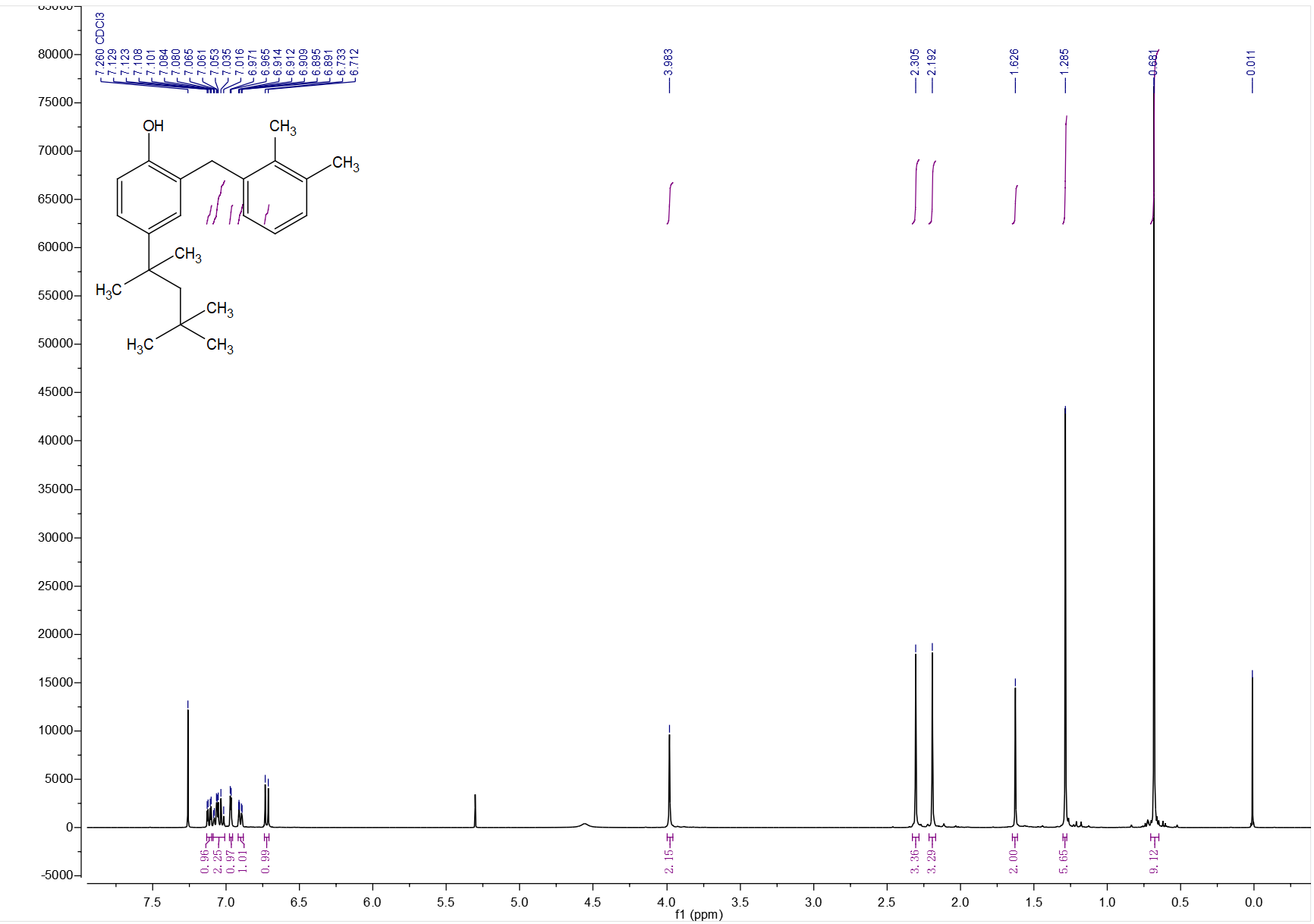


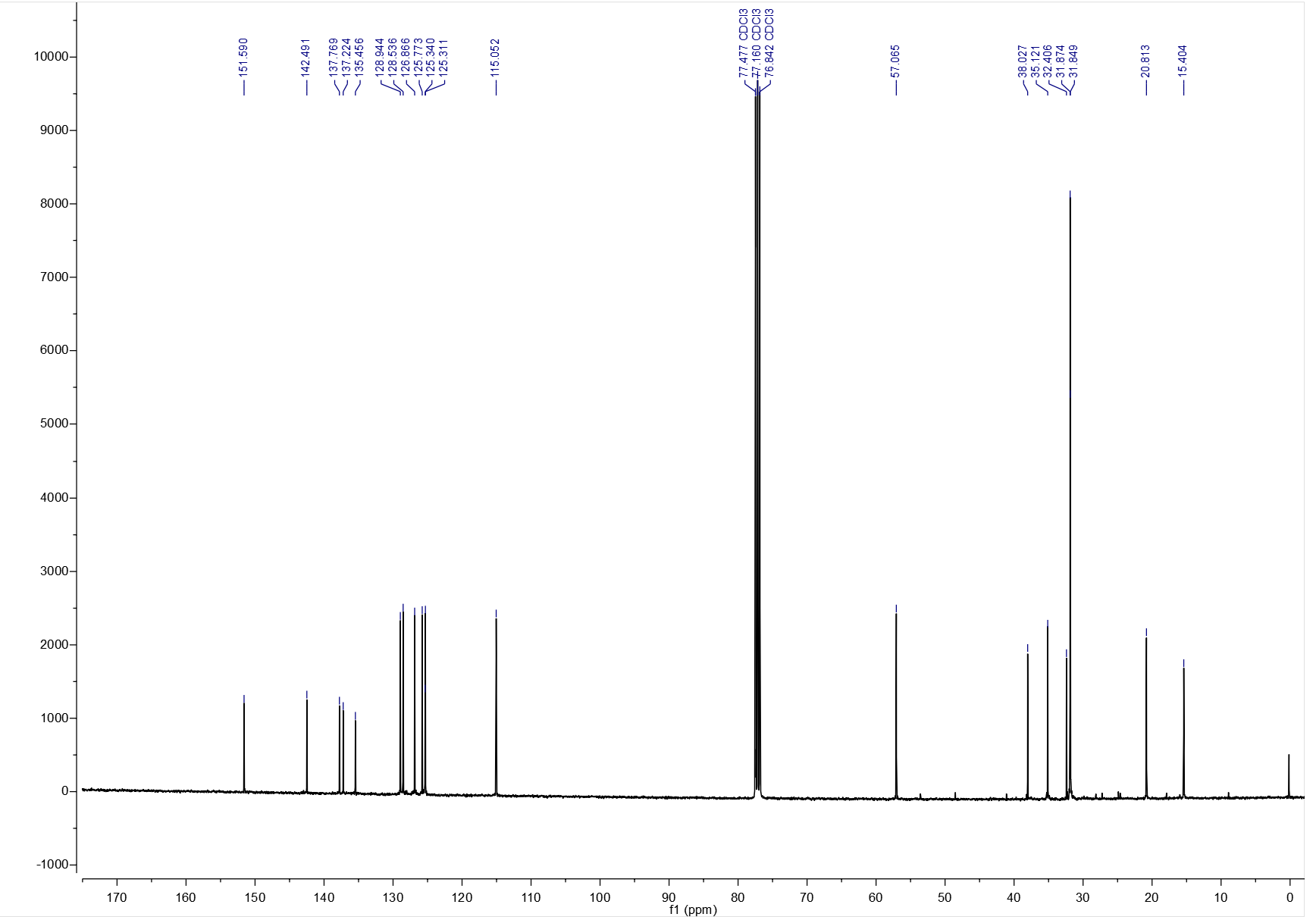


**
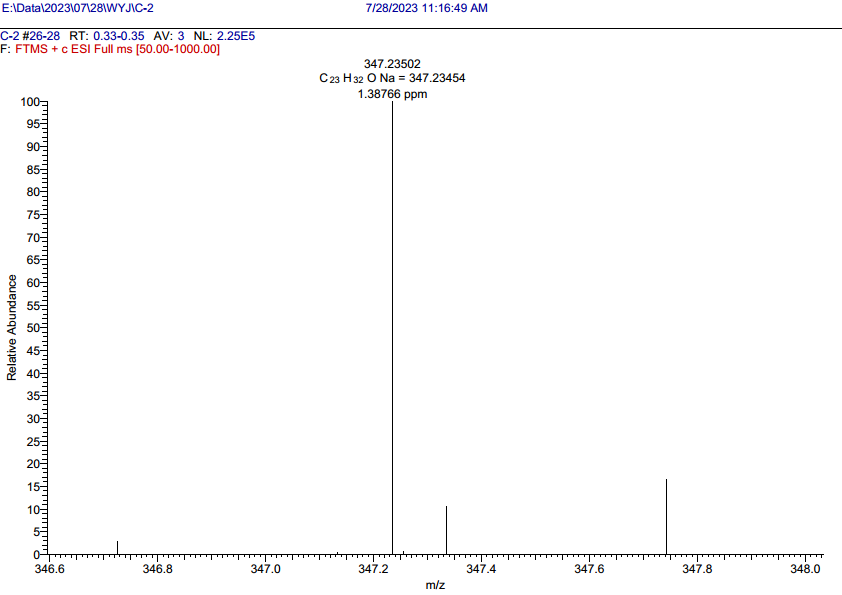
**

**
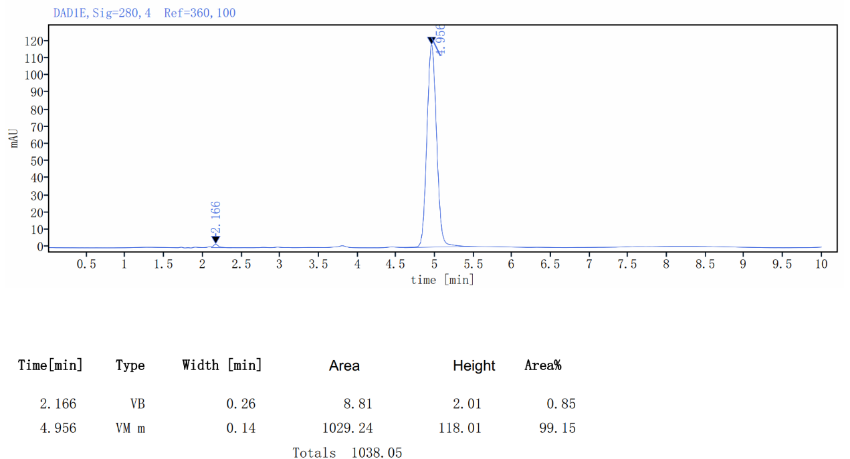
**

**Figure S33.** ^1^H NMR, ^13^C NMR, HRMS and HPLC data of compound **B10**.


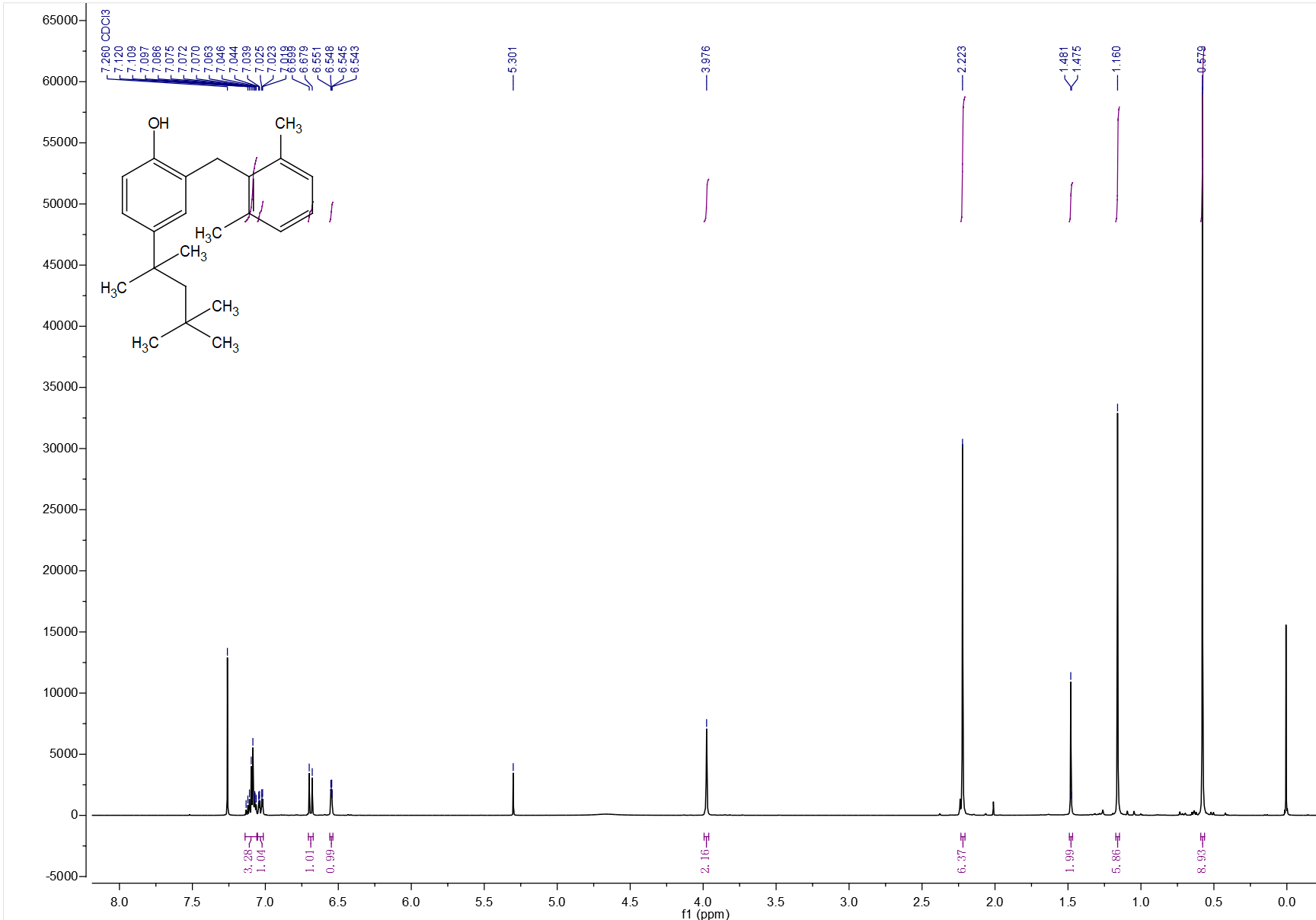


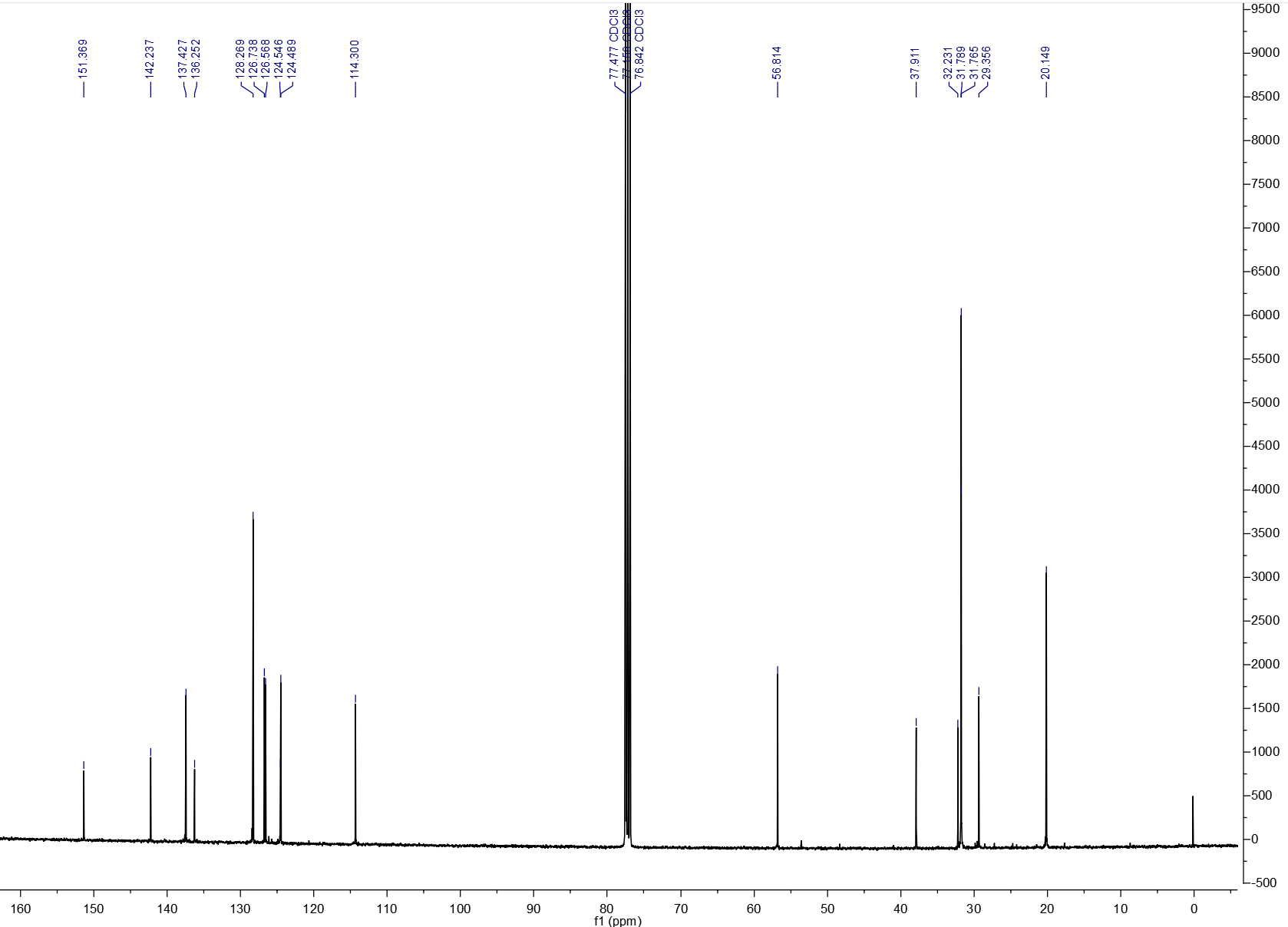


**
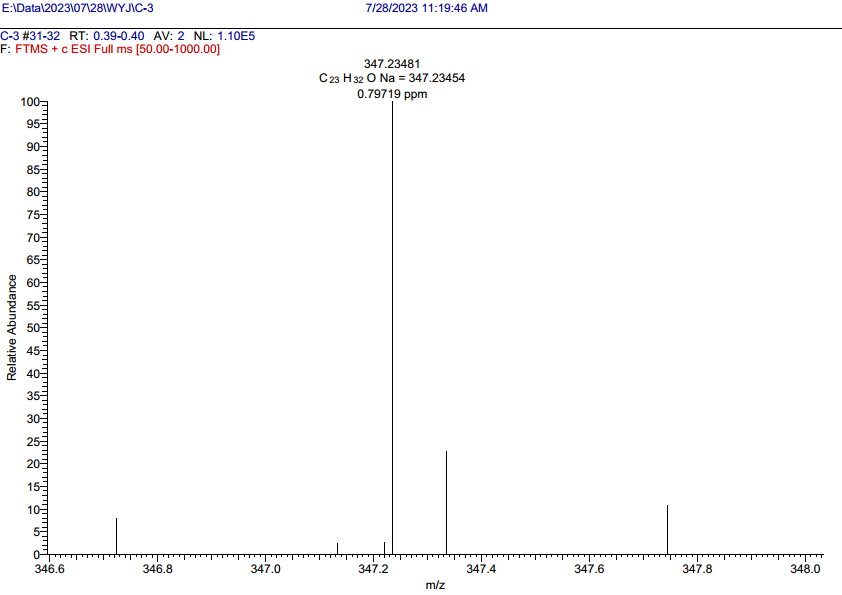
**

**Figure S34.** ^1^H NMR, ^13^C NMR, HRMS and HPLC data of compound **B11**.

**Figure S35.** ^1^H NMR, ^13^C NMR, HRMS and HPLC data of compound **B12**.

**Figure S36.** ^1^H NMR, ^13^C NMR, HRMS and HPLC data of compound **B13**.

**Figure S37.** ^1^H NMR and MS data of compound **5a**.

**Figure S38.** ^1^H NMR and MS data of compound **5b**.

**Figure S39.** ^1^H NMR and MS data of compound **5c**.

**Figure S40.** ^1^H NMR and MS data of compound **5d**.

**Figure S41.** ^1^H NMR, ^13^C NMR, HRMS and HPLC data of compound **C1**.

**Figure S42.** ^1^H NMR, ^13^C NMR, HRMS and HPLC data of compound **C2**.

**Figure S43.** ^1^H NMR, ^13^C NMR, HRMS and HPLC data of compound **C3**.

**Figure S44.** ^1^H NMR, ^13^C NMR, HRMS and HPLC data of compound **C4**.

**Figure S45.** ^1^H NMR and MS data of compound **6**.

**Figure S46.** ^1^H NMR and MS data of compound **7**.

**Figure S47.** ^1^H NMR, ^13^C NMR, HRMS and HPLC data of compound **D1**.

**Figure S48.** ^1^H NMR, ^13^C NMR, HRMS and HPLC data of compound **D2**.

**Figure S49.** ^1^H NMR, ^13^C NMR, HRMS and HPLC data of compound **D3**.

**Figure S50.** ^1^H NMR, ^13^C NMR, HRMS and HPLC data of compound **D4**.

**Figure S51.** ^1^H NMR, ^13^C NMR, HRMS and HPLC data of compound **D5**.
